# Dapagliflozin in heart failure with improved ejection fraction: a prespecified analysis of the DELIVER trial

**DOI:** 10.1038/s41591-022-02102-9

**Published:** 2022-12-15

**Authors:** Orly Vardeny, James C. Fang, Akshay S. Desai, Pardeep S. Jhund, Brian Claggett, Muthiah Vaduganathan, Rudolf A. de Boer, Adrian F. Hernandez, Carolyn S. P. Lam, Silvio E. Inzucchi, Felipe A. Martinez, Mikhail N. Kosiborod, David DeMets, Eileen O’Meara, Shelley Zieroth, Josep Comin-Colet, Jaroslaw Drozdz, Chern-En Chiang, Masafumi Kitakaze, Magnus Petersson, Daniel Lindholm, Anna Maria Langkilde, John J. V. McMurray, Scott D. Solomon

**Affiliations:** 1 Minneapolis VA Center for Care Delivery and Outcomes Research, University of Minnesota, Minneapolis, MN USA; 2 University of Utah Medical Center, Salt Lake City, UT USA; 3 Cardiovascular Division, Brigham and Women’s Hospital, Harvard Medical School, Boston, MA USA; 4 BHF Glasgow Cardiovascular Research Center, School of Cardiovascular and Metabolic Health, University of Glasgow, Glasgow, UK; 5 Department of Cardiology, Thoraxcenter, Erasmus Medical Center, Rotterdam, the Netherlands; 6 Duke University Medical Center, Durham, NC USA; 7 National Heart Centre Singapore and Duke–National University of Singapore, Singapore, Singapore; 8 Yale School of Medicine, New Haven, CT USA; 9 Universidad Nacional de Córdoba, Córdoba, Argentina; 10 Saint Luke’s Mid America Heart Institute and University of Missouri-Kansas City, Kansas City, MO USA; 11 University of Wisconsin, Madison, WI USA; 12 Montreal Heart Institute and Université de Montréal, Montreal, Quebec Canada; 13 St. Boniface Hospital & University of Manitoba, Winnipeg, Manitoba Canada; 14 Cardiology Department, Bellvitge University Hospital, Bio-Heart Bellvitge Institute for Biomedical Research, University of Barcelona, Hospitalet de Llobregat, Barcelona, Spain; 15 Department Cardiology, Medical University Lodz, Lodz, Poland; 16 General Clinical Research Center and Division of Cardiology, Taipei Veterans General Hospital and National Yang Ming Chiao Tung University, Taipei, Taiwan; 17 Kinshukai Hanwa Daini Senboku Hospital, Osaka, Japan; 18 Late-Stage Development, Cardiovascular, Renal, and Metabolism, BioPharmaceuticals R&D, AstraZeneca, Gothenburg, Sweden

**Keywords:** Outcomes research, Cardiovascular diseases

## Abstract

With modern treatments for heart failure with reduced ejection fraction (EF), indicative of impaired cardiac systolic function, patients may exhibit an increase in EF. Limited data are available regarding the clinical management of this growing population, categorized as heart failure with improved EF (HFimpEF), which has a high event rate and has been excluded from virtually all prior heart failure outcomes trials. In a prespecified analysis of the DELIVER trial (NCT03619213), of a total of 6,263 participants with symptomatic heart failure and a left ventricular EF >40%, 1,151 (18%) had HFimpEF, defined as patients whose EF improved from ≤40% to >40%. Participants were randomized to 10 mg dapagliflozin or placebo daily and the primary outcome of the trial was a composite of cardiovascular death or worsening heart failure (heart failure hospitalization or an urgent heart failure visit). Participants with HFimpEF had similar event rates to those with an EF consistently >40%. In participants with HFimpEF, dapagliflozin reduced the primary composite outcome (hazard ratio (HR) = 0.74, 95% confidence interval (CI) = 0.56–0.97), first worsening heart failure events (HR = 0.84, 95% CI = 0.61–1.14), cardiovascular death (HR = 0.62, 95% CI = 0.41–0.96) and total worsening heart failure events (rate ratio = 0.68, 95% CI = 0.50–0.94) to a similar extent as for individuals with an EF consistently >40%. These data suggest that patients with HFimpEF who are symptomatic may benefit from the addition of a sodium/glucose cotransporter 2 inhibitor to previously instituted guideline-directed medical therapy to further reduce morbidity and mortality.

## Abstract (web summary)

A prespecified analysis of the DELIVER clinical trial shows that the sodium/glucose cotransporter 2 inhibitor dapagliflozin benefits patients categorized as having heart failure with improved ejection fraction, a class of patients at high risk of cardiac events but for which limited treatment data are available.

## Main

Heart failure with improved ejection fraction (HFimpEF), formerly referred to as heart failure (HF) with recovered ejection fraction, has been defined as HF with previously reduced left ventricular EF (LVEF) of 40% or less and a subsequent measurement of LVEF that has increased to >40%, often as a result of guideline-directed medical therapy (GDMT)^1^. Although patients with HFimpEF may exhibit a better prognosis than patients with a persistently reduced EF (HFrEF), they still experience clinical events, including HF hospitalizations and cardiovascular mortality, as well as impaired quality of life^2–5^. The 2022 American Heart Association, American College of Cardiology, Heart Failure Society of America (AHA/ACC/HFSA) Guidelines for the Management of Heart Failure provide little guidance regarding the appropriate pharmacological treatment of this population but recommend that patients with HFimpEF should continue treatment with GDMT to avoid relapse and worsening of left ventricular function^6^. Whether initiation of specific new pharmacological therapies might improve clinical outcomes in these patients is unknown.

Sodium/glucose cotransporter 2 (SGLT2) inhibitors have been shown to reduce the risk of both cardiovascular death and hospitalization for HF in patients with HF, regardless of diabetes mellitus^7–11^. However, previous HF trials, including those testing an SGLT2 inhibitor, have explicitly excluded individuals with HFimpEF. The Dapagliflozin Evaluation to Improve the Lives of Patients With Preserved Ejection Fraction Heart Failure (DELIVER) trial demonstrated that the SGLT2 inhibitor dapagliflozin reduced the risk of cardiovascular death, HF hospitalization or urgent HF visits in patients with HF and an LVEF >40% (HR = 0.82, 95% CI = 0.73–0.92)^12^. DELIVER deliberately permitted enrollment of patients with previous LVEF ≤40%^13,14^ and thus provided a unique opportunity to examine the efficacy of dapagliflozin in this previously understudied group of patients with HF. The objective of this analysis was to determine the efficacy and safety of dapagliflozin in patients with HFimpEF. We hypothesized that patients with HFimpEF would benefit from dapagliflozin to a similar extent as patients with LVEF consistently over 40%, regardless of achieved EF.

## Results

### Patients by HFimpEF status

**Fig. 1:**
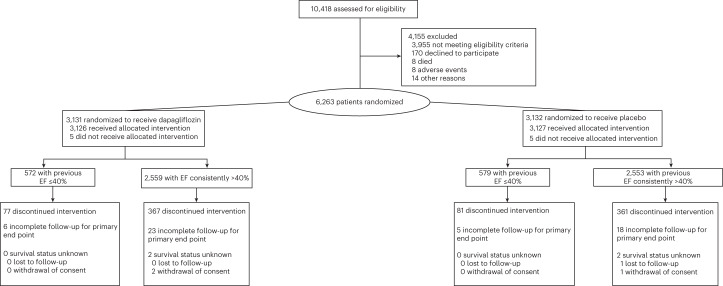
CONSORT diagram. Enrollment, randomization and follow-up of participants in those with HF with improved EF and those with HF with an EF consistently over 40%.

Between 1 September 2018 and 18 January 2021, 3,131 patients were assigned to dapagliflozin and 3,132 patients were assigned to placebo (Fig. 1). Of these, 1,151 participants (18%) had a history of previous LVEF of 40% or less (572 were assigned to dapagliflozin and 579 to placebo). Vital status was known in all patients with HF and improved EF at the end of the study; complete follow-up was available for the primary end point in all but six patients in the dapagliflozin group and five patients in the placebo group. Compared to those with LVEF consistently >40% (Table 1, left three columns), those with HFimpEF were younger, more likely to be male, less likely to be White and had a lower LVEF at baseline (50.5 ± 8.3% versus 55.0 ± 8.7%, *P* < 0.001). Baseline N-terminal (NT)-pro hormone BNP (NT-proBNP) levels were similar between groups. A higher percentage of participants with HFimpEF compared with those with LVEF consistently over 40% had a history of coronary artery disease, myocardial infarction and were more likely to be previously hospitalized for HF or had implantable cardioverter defibrillators (ICDs). Those with HFimpEF generally carried a diagnosis of HF for longer and were more likely to have New York Heart Association (NYHA) functional class II (versus III and IV) compared to those with LVEF consistently over 40%. Participants with HFimpEF, compared with those with LVEF consistently over 40%, were more likely to be treated at baseline with angiotensin-converting enzyme (ACE) inhibitor or angiotensin receptor neprilysin inhibitor, beta blockers or mineralocorticoid receptor antagonists. The proportion of patients who were on full guideline-directed medical therapy for HFrEF was higher in those with HFimpEF than in those with LVEF consistently over 40%.

**Table 1:** Baseline characteristics by HFimpEF status and treatment group in those with HFimpEF

	Patients with HFimpEF versus those with EF consistently over 40%			Treatment groups in patients with HFimpEF		
	HFimpEF	EF consistently >40%	*P*	Dapagliflozin	Placebo	*P*
	*n* = 1,151	*n* = 5,112				
Age (years)	70.1 ± 10.0	72.0 ± 9.4	<0.001	69.9 ± 10.3	70.3 ± 9.8	0.58
Male sex	774 (67.2%)	2,742 (53.6%)	<0.001	387 (67.7%)	387 (66.8%)	0.77
Race			<0.001			0.10
White	774 (67.2%)	3,665 (71.7%)		382 (66.8%)	392 (67.7%)	
Asian	290 (25.2%)	984 (19.2%)		140 (24.5%)	150 (25.9%)	
Black or African American	36 (3.1%)	123 (2.4%)		16 (2.8%)	20 (3.5%)	
American Indian or Alaska Native	21 (1.8%)	168 (3.3%)		12 (2.1%)	9 (1.6%)	
Other	30 (2.6%)	172 (3.4%)		22 (3.8%)	8 (1.4%)	
Geographical region			<0.001			0.64
Europe and Saudi Arabia	482 (41.9%)	2,523 (49.4%)		236 (41.3%)	246 (42.5%)	
Asia	284 (24.7%)	942 (18.4%)		136 (23.8%)	148 (25.6%)	
Latin America	198 (17.2%)	983 (19.2%)		106 (18.5%)	92 (15.9%)	
North America	187 (16.2%)	664 (13.0%)		94 (16.4%)	93 (16.1%)	
History of atrial fibrillation or flutter	593 (51.5%)	2,959 (57.9%)	<0.001	279 (48.8%)	314 (54.2%)	0.06
Type 2 diabetes mellitus	529 (46.0%)	2,277 (44.5%)	0.38	285 (49.8%)	244 (42.1%)	0.009
History of myocardial infarction	400 (34.8%)	1,239 (24.2%)	<0.001	197 (34.4%)	203 (35.1%)	0.83
History of HF hospitalization	560 (48.7%)	1,979 (38.7%)	<0.001	270 (47.2%)	290 (50.1%)	0.33
Any coronary artery disease	676 (58.7%)	2,488 (48.7%)	<0.001	338 (59.1%)	338 (58.4%)	0.81
Any atherosclerotic cardiovascular disease	729 (63.3%)	2,823 (55.2%)	<0.001	367 (64.2%)	362 (62.5%)	0.56
Current smoker	118 (10.3%)	366 (7.2%)		49 (8.6%)	69 (11.9%)	0.17
Baseline body mass index (kg m^−2^)	29.4 ± 6.0	29.9 ± 6.1	0.008	29.7 ± 6.2	29.2 ± 5.7	0.16
Time from diagnosis of HF to baseline			<0.001			0.62
0–3 months	61 (5.3%)	507 (9.9%)		31 (5.4%)	30 (5.2%)	
>3–6 months	70 (6.1%)	522 (10.2%)		36 (6.3%)	34 (5.9%)	
>6–12 months	114 (9.9%)	728 (14.3%)		63 (11.0%)	51 (8.8%)	
>1–2 years	149 (12.9%)	846 (16.6%)		80 (14.0%)	69 (11.9%)	
>2–5 years	350 (30.4%)	1,219 (23.9%)		168 (29.4%)	182 (31.4%)	
>5 years	407 (35.4%)	1,285 (25.2%)		194 (33.9%)	213 (36.8%)	
NYHA class at baseline			0.001			0.89
I	0 (0%)	1 (0%)				
II	918 (79.8%)	3,795 (74.2%)		453 (79.2%)	465 (80.3%)	
III	229 (19.9%)	1,302 (25.5%)		117 (20.5%)	112 (19.3%)	
IV	4 (0.3%)	14 (0.3%)		2 (0.3%)	2 (0.3%)	
Baseline LVEF (%)	50.5 ± 8.3	55.0 ± 8.7	<0.001	50.3 ± 7.9	50.8 ± 8.7	0.29
LVEF group			<0.001			0.47
≤40	1 (0.1%)	3 (0.1%)		1 (0.2%)	0 (0%)	
≥41–49	623 (54.1%)	1,489 (29.1%)		313 (54.7%)	310 (53.5%)	
50–59	328 (28.5%)	1,928 (37.7%)		167 (29.2%)	161 (27.8%)	
≥60	199 (17.3%)	1,692 (33.1%)		91 (15.9%)	108 (18.7%)	
Baseline NT-proBNP (pg ml^−1^)	1,009 (623–1,728)	1,012 (623–1,753)	0.96	1,010 (627–1,812)	1,007 (614–1,688)	0.40
Baseline ECG atrial fibrillation/flutter	424 (36.8%)	2,220 (43.4%)	<0.001	208 (36.4%)	216 (37.3%)	0.74
Baseline systolic blood pressure (mm Hg)	127.2 ± 16.6	128.5 ± 15.0	0.016	127.3 ± 16.8	127.2 ± 16.5	0.87
Baseline eGFR (ml min^−1^ per 1.73 m^2^)	61.9 ± 19.2	60.8 ± 19.1	0.10	61.9 ± 19.0	61.8 ± 19.3	0.90
Medications						
Loop diuretics	883 (76.8%)	3,928 (76.9%)	0.96	446 (78.0%)	437 (75.6%)	0.34
ACE inhibitor	458 (39.8%)	1,837 (35.9%)	0.014	224 (39.2%)	234 (40.5%)	0.65
ARB	337 (29.3%)	1,935 (37.9%)	<0.001	166 (29.0%)	171 (29.6%)	0.83
Neprilysin inhibitor/ARB (ARNI)	152 (13.2%)	149 (2.9%)	<0.001	73 (12.8%)	79 (13.7%)	0.65
Beta blocker	991 (86.2%)	4,186 (81.9%)	<0.001	486 (85.0%)	505 (87.4%)	0.24
Mineralocorticoid receptor antagonist	580 (50.4%)	2,087 (40.8%)	<0.001	290 (50.7%)	290 (50.2%)	0.86
ICD	59 (5.1%)	54 (1.1%)	<0.001	36 (6.3%)	23 (4.0%)	0.07
ACE inhibitor, ARB, ARNI and beta blocker	826 (72%)	3,233 (63%)	<0.001	399 (69.8%)	427 (73.9%)	0.12
ACE inhibitor, ARB, ARNI, beta blocker and mineralocorticoid receptor antagonist	425 (37%)	1,406 (28%)	<0.001	208 (36.4%)	217 (37.5%)	0.68

Among patients with HFimpEF, the groups assigned to dapagliflozin and placebo were well balanced (Table 1, right three columns). Among patients with HFimpEF, 77 patients discontinued study treatment and 6 had incomplete follow-up for the primary end point in the dapagliflozin group; 81 patients discontinued study treatment and 5 had incomplete follow-up for the primary end point in the placebo group. Vital status was known in all patients with HFimpEF in both treatment groups.

### Outcomes by HFimpEF status

The trial ended on reaching the prespecified number of events (at least 1,117). The rates of worsening HF or cardiovascular death were similar among those with HFimpEF and those with LVEF consistently over 40% (8.8 per 100 patient years versus 8.7 per 100 patient years, respectively) (Table 2). Results were consistent after adjustment for age, sex and geographical region (adjusted HR = 0.99, 95% CI = 0.85–1.15, *P* = 0.92). Rates of cardiovascular death, first HF events (HF hospitalizations or urgent HF visits), all-cause death and total HF events were also similar between those with HFimpEF and those with LVEF consistently over 40%.

**Table 2:** Primary and secondary outcomes according to HFimpEF status

	Previous EF ≤ 40%	Prior EF > 40%	*P*
Outcome	*n* = 1,151	*n* = 5,112	
Primary composite (cardiovascular death or worsening HF)	211 events	911 events	0.92
	(8.8 per 100 patient years)	(8.7 per 100 patient years)	
	HR = 0.99 (0.85–1.15)	(REF)	
Cardiovascular death	87 events	405 events	0.63
	(3.4 per 100 patient years)	(3.6 per 100 patient years)	
	HR = 0.94 (0.75, 1.19)	(REF)	
HF event	161 events	662 events	0.74
	(6.7 per 100 patient years)	(6.3 per 100 patient years)	
	HR = 1.03 (0.86–1.23)	(REF)	
HF hospitalization	144 events	603 events	0.94
	(6.0 per 100 patient years)	(5.7 per 100 patient years)	
	HR = 1.01 (0.84–1.21)	(REF)	
Urgent HF visit	34 events	104 events	0.10
	(1.3 per 100 patient years)	(0.9 per 100 patient years)	
	HR = 1.39 (0.94–2.06)	(REF)	
All-cause death	190 events	833 events	0.76
	(7.4 per 100 patient years)	(7.4 per 100 patient years)	
	HR = 1.03 (0.87–1.20)	(REF)	
Composite of cardiovascular death and recurrent HF events	351 events	1521 events	0.80
	(13.7 per 100 patient years)	(13.5 per 100 patient years)	
	RR = 0.98 (0.82–1.17)	(REF)	

**Fig. 2:**
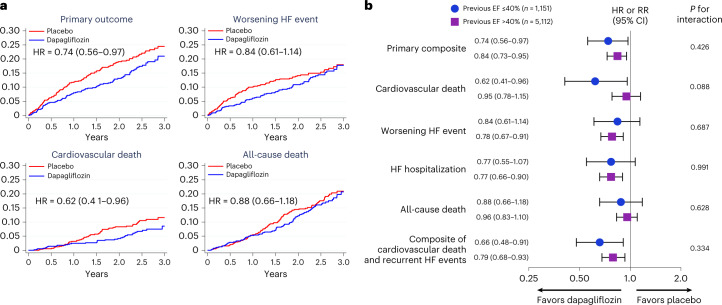
Primary and secondary end points. **a**, Incidence of the primary outcome (upper left), worsening HF (upper right), cardiovascular death (lower left) and all-cause death (lower right) by treatment assignment in patients with HF with improved EF. Participants randomized to dapagliflozin are indicated in blue and those randomized to placebo in red. Each of the graphs shows Kaplan–Meier curves with an HR and 95% CI estimated from a Cox’s model with two-sided *P* values. No adjustment for multiple comparisons was made. **b**, Primary and secondary end points by treatment assignment in patients with previous EF of 40% or less and in those with EF consistently over 40%. Estimates are HRs or RRs; 95% CIs were estimated from Cox models with two-sided *P* values and are displayed as error bars. The RR was calculated for the assessment of cardiovascular death and total HF events using the method of Lin et al.^23^. Interaction *P* values refer to the treatment by subgroup interaction and represent a two-sided *P* value for interaction from the Wald test of the Cox model.

**Fig. 3:**
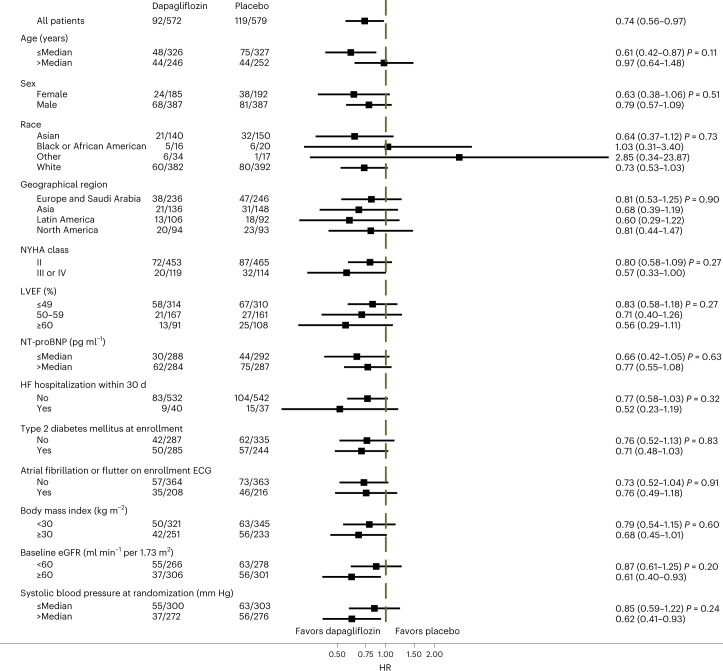
Primary end point in prespecified subgroups in patients with HF with improved EF. Estimates are HRs with the error bars representing the 95% CIs from the Cox model and a two-sided *P* value for interaction from the Wald test of the Cox model. No adjustment for multiple comparisons was made.

Dapagliflozin reduced the primary composite end point compared to placebo in participants with HFimpEF (HR = 0.74, 95% CI = 0.56–0.97) to a similar extent as patients with LVEF consistently over 40% (HR = 0.84, 95% CI 0.73–0.95; interaction *P* = 0.43) (Fig. 2a,b). Among those with HFimpEF, the benefit of dapagliflozin relative to placebo was consistent across prespecified subgroups, including LVEF at enrollment (LVEF ≤ 49%: HR = 0.84, 95% CI = 0.59–1.20; LVEF = 50–59%: HR = 0.73, 95% CI = 0.41–1.29; LVEF ≥ 60%: HR = 0.55, 95% CI = 0.28–1.07; interaction *P* = 0.24; Fig. 3). The effect of dapagliflozin on HF outcomes was also similar in those with HFimpEF (HR = 0.84, 95% CI = 0.61–1.14) and those with LVEF consistently over 40% (HR = 0.78, 95% CI = 0.67–0.91; interaction *P* = 0.69), as it was for cardiovascular death among individuals with HFimpEF (HR = 0.62, 95% CI = 0.41–0.96) and in those with LVEF consistently over 40% (HR = 0.95, 95% CI = 0.78–1.15; interaction *P* = 0.09). The composite of total HF events and cardiovascular events was also similarly reduced in patients with HFimpEF (rate ratio (RR) = 0.68, 95% CI = 0.50–0.94) as in patients with LVEF consistently over 40% (RR = 0.79, 95% CI = 0.67–0.93; interaction *P* = 0.43). These findings were similar irrespective of age (primary end point, interaction *P* = 0.43 for age as a continuous variable and interaction *P* = 0.28 for age ≥75 versus <75 years and for cardiovascular death, interaction *P* = 0.71 for age as a continuous variable and interaction *P* = 0.42 for age ≥75 versus <75 years).

### Symptom burden by HFimpEF status

At baseline, participants with HFimpEF had higher Cardiomyopathy Questionnaire (Kansas City) (KCCQ) total symptom scores compared to those with LVEF consistently over 40% (73.5 ± 21.3 versus 69.3 ± 22.3, *P* < 0.001). Overall, participants randomized to dapagliflozin experienced an increase in KCCQ summary scores compared to baseline relative to placebo, irrespective of HFimpEF status, although the magnitude of change in KCCQ was numerically smaller in those with HFimpEF compared to those with LVEF consistently over 40% (HFimpEF mean difference = +0.90, 95% CI = −1.32 to +3.11 versus without HFimpEF mean difference = +2.8, 95% CI = 1.70–3.90, interaction *P* = 0.14). Sensitivity analyses using KCCQ data from months 1, 4 and 8 via longitudinal mixed effects models with random patient intercepts and treatment, study visit and treatment–visit interaction terms as fixed effects produced similar results (HFimpEF mean difference = +0.80, 95% CI = −1.28 to +2.88 versus those with LVEF consistently over 40% (mean difference = +2.78, 95% CI = 1.75–3.81, interaction *P* = 0.10).

### Safety and tolerability by HFimpEF status

Frequencies of study medication discontinuation were similar between participants with HFimpEF compared to those with LVEF consistently over 40% (13.8 versus 14.3%, *P* = 0.65) regardless of treatment assignment (Table 3). Diabetic ketoacidosis and major hypoglycemia were infrequent and did not differ by HFimpEF status or treatment assignment. Serious adverse events or events leading to study drug discontinuation suggestive of volume depletion occurred in 18 participants in the HFimpEF group (10 (1.7%) in the dapagliflozin group and 8 (1.4%) in the placebo group) and in 56 individuals in those with LVEF consistently over 40% (32 (1.3%) in the dapagliflozin group and 24 (0.9%) in the placebo group).

**Table 3:** Safety outcomes by HFimpEF status

	Previous LVEF ≤ 40%, *n* = 1,151		Previous EF > 40%, *n* = 5,112	
Safety outcome	Dapa, *n* = 572	Placebo, *n* = 579	Dapa, *n* = 2,559	Placebo, *n* = 2,553
Discontinuation of study drug	77 (13%)	81 (14%)	367 (14%)	361 (14%)
Any serious adverse event	247 (43.2%)	273 (47.3%)	1,114 (43.6%)	1,150 (45.1%)
Any adverse event leading to discontinuation of IP	36 (6.3%)	38 (6.6%)	146 (5.7%)	143 (5.6%)
Any adverse event leading to interruption of IP	79 (13.8%)	92 (15.9%)	357 (14.0%)	402 (15.8%)
Any amputation	5 (0.9%)	7 (1.2%)	14 (0.5%)	18 (0.7%)
Any potential risk factor adverse event for amputation affecting lower limbs	38 (6.6%)	56 (9.7%)	150 (5.9%)	143 (5.6%)
Any definite or probable diabetic ketoacidosis	0 (0%)	0 (0%)	2 (0.1%)	0 (0%)
Any major hypoglycemic event	1 (0.2%)	1 (0.2%)	5 (0.2%)	6 (0.2%)
Any serious adverse event or adverse event leading to study drug discontinuation suggestive of volume depletion	10 (1.7%)	8 (1.4%)	32 (1.3%)	24 (0.9%)
Any renal serious adverse event or adverse event leading to study drug discontinuation	14 (2.4%)	16 (2.8%)	59 (2.3%)	63 (2.5%)

## Discussion

We found that in patients with HFimpEF enrolled in the DELIVER trial, dapagliflozin reduced the composite of cardiovascular death or worsening HF events, and other outcomes, to a similar extent as in those with LVEF consistently over 40%. The benefit in patients with HFimpEF did not differ by the achieved LVEF at baseline. Similarly, the rates of adverse events were comparable between groups. These data suggest that patients with HF who previously had reduced LVEF but whose LV function has improved above 40% yet remain symptomatic benefit from the addition of dapagliflozin.

Individuals with HFimpEF comprised 18% of the total patients in DELIVER, the only cohort of patients with HFimpEF enrolled in a randomized outcome trial of any therapeutic agent. Clinical guidelines have largely been limited on recommendations for this population because of the lack of high-quality evidence. This phenotype represents a distinct group, as evidenced by the striking differences in baseline characteristics in HFimpEF patients in DELIVER compared with those with an LVEF consistently over 40%. In particular, these patients had baseline characteristics that more resembled those of the HFrEF population from which they emerged, including younger age, male sex, longer duration of HF and the more frequent treatment with ACE inhibitors, ARBs, ARNI, beta blockers and ICDs. The lower burden of HF symptoms (albeit with similar natriuretic peptide levels) in this group may reflect the higher use of GDMT compared with those with an LVEF consistently >40%. Nevertheless, event rates in this group were similar to those with LVEF consistently >40%, reinforcing the notion that this phenotype does not signify ‘recovery’, or normalization of LV function, but that these patients are at substantial risk for adverse outcomes. The event rates of this cohort in DELIVER may have even been higher than reported in previous observational studies^3,15–17^, potentially in part because of the elevated natriuretic peptide levels and persistent symptoms required by DELIVER.

Data on the management of patients with HFimpEF are sparse. In the 51-patient, open-label randomized TRED-HF trial, withdrawal of GDMT in patients with asymptomatic dilated cardiomyopathy who had had a previously reduced LVEF, but whose LVEF increased to 50% or higher, resulted in deterioration of EF within 6 months in nearly half of the patients studied^6^. A recent scientific statement^2^ and the most recent AHA/ACC/HFSA HF guidelines recommended the continuation of GDMT in patients with HFimpEF as a consequence of these data^2^. Virtually all previous outcome trials in patients with HF and an LVEF > 40%, including those testing candesartan, spironolactone and sacubitril/valsartan and empagliflozin excluded patients with previous LVEF ≤40%^9,18–20^. Thus, these data from DELIVER are the first to inform initiation of GDMT in patients with HFimpEF. This is a growing population of patients with HF and it is estimated that between 10 and 40% of those with previous HFrEF have HFimpEF. Since patients with HFimpEF are more likely to be cared for by general practitioners once GDMT has been optimized and have more frequent encounters with these practitioners compared to cardiologists, these findings are applicable to the broader medical community.

We noted that patients with HFimpEF enrolled in DELIVER had a lower symptom burden (as indicated by higher baseline KCCQ total symptom score) than those with LVEF consistently over 40%. This may in part be due to symptomatic improvement before enrollment, potentially from GDMT. While there was no heterogeneity in the treatment response with respect to improvement in total symptom score in patients with HFimpEF compared with those without HFimpEF, the numerically lower magnitude of the benefit with dapagliflozin may reflect that these patients started with fewer symptoms and may have less potential for improvement.

While formal interaction testing did not show significant heterogeneity between the HFimpEF group or those with LVEF consistently over 40% for either the primary end point or cardiovascular death, the lower point estimate for cardiovascular death in the group with HFimpEF raise the possibility that the ability of dapagliflozin to modify disease progression may even be greater in the improved group than in those with LVEF consistently over 40%, as it may in patients with HFrEF.

This analysis has several limitations. Data on previous LVEF was investigator-reported and we did not collect the exact previous LVEF value; nevertheless, the baseline characteristics of the HFimpEF cohort were substantially different from those with LVEF consistently over 40%, signifying that this cohort represents a distinct phenotype. While the recent AHA/ACC/HFSA guidelines have designated HFimpEF as HF with LVEF ≤ 40%, with a subsequent measurement of LVEF that has increased to >40%; other definitions have varied. The 2021 European Society of Cardiology Guidelines for the diagnosis and treatment of acute and chronic heart failure define HFimpEF as patients with a history of LVEF ≤ 40% who present with a follow-up LVEF of ≥50%; the universal definition position paper characterizes HFimpEF as at least a 10-point increase in LVEF from a baseline that is ≤40%^21,22^. While we were not able to determine if patients had a 10-point increase, the magnitude of the benefit was consistent, if not better, in HFimpEF patients with an achieved LVEF of 50% or higher (primary end point HR = 0.63, 95% CI = 0.41–0.97; cardiovascular death HR = 0.38, 95% CI = 0.17–0.87). Similarly, the DELIVER trial did not collect data on the duration of medical or device therapies that may have allowed improvement in LVEF. Also, improvement in LVEF varies substantially by specific etiologies of HF, which were not captured as part of the trial. Previous trials in heart failure with mid-range EF and heart failure with preserved EF excluded patients with improved EF; the DAPA-HF study did not collect longitudinal measures of LVEF, thus precluding the ability to perform pooled analyses with other outcome studies. While this analysis was prespecified in the academic statistical analysis plan, it is one of many subgroups and DELIVER was not powered specifically to assess a treatment effect in this cohort. Nevertheless, given that we observed no heterogeneity in the treatment benefit by HFimpEF status, and that these patients have been excluded from nearly all other trials in this population, these data should inform clinical decision-making and forthcoming clinical practice guidelines on the contemporary management of patients with HFimpEF.

In summary, in patients enrolled in the DELIVER study who had HFimpEF, dapagliflozin compared with placebo reduced the composite of cardiovascular death or worsening HF, and other outcomes, to a similar extent as patients with LVEF consistently over 40%. Despite previous improvement in LVEF, patients with HFimpEF in DELIVER faced heightened risks of disease progression including worsening HF events and death, which were comparable to those who had LVEF consistently over 40%. These data serve as the largest randomized data of this population and suggest that patients with HFimpEF who are symptomatic may benefit from the addition of dapagliflozin.

## Methods

### Trial design and oversight

DELIVER was an international, prospective, randomized, double-blind, placebo-controlled trial conducted at 353 sites across 20 countries testing the efficacy and safety of dapagliflozin compared to placebo in patients with HF and mildly reduced, preserved or improved EF. The trial design and conduct of this trial was previously published elsewhere^13^. The protocol was approved by the local ethics committees at each participating site and each patient provided written informed consent in accordance with established guidelines. The trial was reviewed by an independent monitoring committee. See Supplementary Appendix for the listing of the sites and investigators.

### Trial patients

The study enrolled patients aged 40 or older with symptomatic HF (NYHA functional class II–IV) and a LVEF >40% (within 12 months of enrollment), elevated levels of natriuretic peptides (NT-proBNP of at least 300 pg ml^−1^ in those without atrial fibrillation or flutter, or at least 600 pg ml^−1^ in those in atrial fibrillation or flutter) and evidence of structural heart disease (left atrial enlargement or left ventricular hypertrophy). Patients with or without type 2 diabetes mellitus, either an outpatient or hospitalized for worsening HF were eligible for enrollment. Patients were also eligible if they previously had an LVEF ≤ 40% but had an LVEF > 40% on their qualifying echocardiogram (ECG) at enrollment. The main exclusion criteria were the use of SGLT2 inhibitors within 4 weeks of randomization, previous intolerance to SGLT2 inhibitors, type 1 diabetes mellitus, estimated glomerular filtration rate (eGFR) <25 ml per min per 1.73 m^2^ at screening and systolic blood pressure ≥160 mm Hg if not using 3 or more blood pressure-lowering medications or ≥180 mm Hg regardless of the number of antihypertensives. Patients were also excluded if they had diagnoses that could alternatively account for their HF symptoms (for example, anemia, primary pulmonary hypertension). Participants were randomized in a 1:1 fashion to dapagliflozin 10 mg or matched placebo daily, stratified by type 2 diabetes status.

### Baseline categorization of improved EF status

Identification of participants with HFimpEF was based on the case report form question: Does the patient have a medical history of symptomatic heart failure with reduced ejection fraction (LVEF ≤ 40%)? The timing of previous low EF assessment and severity of previous LV dysfunction were not collected. The most recent EF before randomization was also recorded and was required to fulfill the entry criteria.

### Randomization

Participants were centrally assigned to a randomized investigational product (IP) using an interactive voice/Web response system (IxRS). Randomization to IP was performed in balanced blocks to ensure approximate balance between the treatment groups (1:1). The blocks were not revealed to the investigators. Randomization was stratified in the IxRS system based on whether the patient was or was not known to have type 2 diabetes at the time of randomization (based on either an established diagnosis or glycated hemoglobin ≥6.5% at enrollment). Patients were randomized in a 1:1 fashion to dapagliflozin 10 mg or matching placebo once daily.

### Study interventions and procedures

After randomization, study visits occurred at approximately days 30, 120, 240, 360 and 480 after randomization and then every 120 d thereafter. Unscheduled visits could also be performed at the discretion of the investigator. Treatment adherence was assessed by asking patients to return all unused IPs and empty packages to the clinic at site visits.

### Outcomes

The primary outcome of the DELIVER trial was the composite of cardiovascular death or worsening HF (defined as either an unplanned hospitalization or urgent HF visit requiring intravenous therapy). Secondary outcomes included total number of HF events (for example, both first and recurrent hospitalizations for HF or urgent visits for HF) and cardiovascular death, quality of life assessed by the KCCQ total symptom score (range = 0–100; higher scores indicate fewer symptoms; ≥5-point individual change considered clinically meaningful), cardiovascular death and all-cause mortality. Clinical outcomes were adjudicated by an independent, blinded adjudication committee. The analysis of the HFimpEF subgroup was prespecified in the academic statistical analysis plan before database lock.

### Statistical analysis

Data from all randomized patients were included in this analysis (see protocol and statistical analysis plans in Supplementary Appendix). The trial was event-driven and the statistical assumptions underlying DELIVER have been published elsewhere^13^. Details of the DELIVER Statistical Analysis Plan (SAP) have been published previously. Primary and secondary time-to-event outcomes from the DELIVER SAP were compared between treatment arms using the intention-to-treat approach, utilizing data from all randomized patients according to their randomized assignment without censoring for discontinuation of study drug. The analysis of participants with HFimpEF was prespecified. Baseline characteristics in participants with HFimpEF and those with LVEF consistently over 40% were summarized as means and s.d., medians and interquartile ranges or percentages and compared by chi-squared test for categorical variables and Wilcoxon test and two-sample Student’s *t*-test for nonnormal and normally distributed continuous variables, respectively. Sex and race were determined by self-report. Time-to-event data for the primary outcome and secondary clinical outcomes according to HFimpEF status, regardless of treatment allocation, were evaluated using the Kaplan–Meier estimator and Cox proportional-hazards models, stratified by diabetes status at randomization. Analysis of total HF events and cardiovascular death was carried out with a semiparametric Cox model utilizing a robust variance estimator. Interactions between HFimpEF status and treatment were evaluated. In patients with HFimpEF, the treatment effect for the primary outcome was evaluated in prespecified subgroups. The composite of total (recurrent) HF events and cardiovascular death was assessed using the method by Lin et al.^23^. KCCQ change from baseline at month 8 was analyzed in patients with available paired data. Change in the KCCQ total symptom score was assessed using analysis of covariance adjusting for baseline measure and treatment. All analyses were performed in Stata v.17 (StataCorp).

### Reporting summary

Further information on research design is available in the Nature Portfolio Reporting Summary linked to this article.

## Online content

Any methods, additional references, Nature Portfolio reporting summaries, source data, extended data, supplementary information, acknowledgements, peer review information; details of author contributions and competing interests; and statements of data and code availability are available at 10.1038/s41591-022-02102-9.

## Supplementary information

Supplementary Information Supplementary Appendix containing the list of DELIVER committees, national lead investigators, all investigators and sites by country, DELIVER protocol, statistical analysis plan and academic statistical analysis plan.

Reporting Summary

## Data Availability

The data underlying the findings described in this manuscript may be obtained by following the AstraZeneca’s data sharing policy described at https://astrazenecagrouptrials.pharmacm.com/ST/Submission/Disclosure.
