# Author Correction: Dapagliflozin in heart failure with improved ejection fraction: a prespecified analysis of the DELIVER trial

**DOI:** 10.1038/s41591-023-02302-x

**Published:** 2023-03-17

**Authors:** Orly Vardeny, James C. Fang, Akshay S. Desai, Pardeep S. Jhund, Brian Claggett, Muthiah Vaduganathan, Rudolf A. de Boer, Adrian F. Hernandez, Carolyn S. P. Lam, Silvio E. Inzucchi, Felipe A. Martinez, Mikhail N. Kosiborod, David DeMets, Eileen O’Meara, Shelley Zieroth, Josep Comin-Colet, Jaroslaw Drozdz, Chern-En Chiang, Masafumi Kitakaze, Magnus Petersson, Daniel Lindholm, Anna Maria Langkilde, John J. V. McMurray, Scott D. Solomon

**Affiliations:** 1 Minneapolis VA Center for Care Delivery and Outcomes Research, University of Minnesota, Minneapolis, MN USA; 2 University of Utah Medical Center, Salt Lake City, UT USA; 3 Cardiovascular Division, Brigham and Women’s Hospital, Harvard Medical School, Boston, MA USA; 4 BHF Glasgow Cardiovascular Research Center, School of Cardiovascular and Metabolic Health, University of Glasgow, Glasgow, UK; 5 Department of Cardiology, Thoraxcenter, Erasmus Medical Center, Rotterdam, the Netherlands; 6 Duke University Medical Center, Durham, NC USA; 7 National Heart Centre Singapore and Duke–National University of Singapore, Singapore, Singapore; 8 Yale School of Medicine, New Haven, CT USA; 9 Universidad Nacional de Córdoba, Córdoba, Argentina; 10 Saint Luke’s Mid America Heart Institute and University of Missouri-Kansas City, Kansas City, MO USA; 11 University of Wisconsin, Madison, WI USA; 12 Montreal Heart Institute and Université de Montréal, Montreal, Quebec Canada; 13 St. Boniface Hospital & University of Manitoba, Winnipeg, Manitoba Canada; 14 Cardiology Department, Bellvitge University Hospital, Bio-Heart Bellvitge Institute for Biomedical Research, University of Barcelona, Hospitalet de Llobregat, Barcelona, Spain; 15 Department Cardiology, Medical University Lodz, Lodz, Poland; 16 General Clinical Research Center and Division of Cardiology, Taipei Veterans General Hospital and National Yang Ming Chiao Tung University, Taipei, Taiwan; 17 Kinshukai Hanwa Daini Senboku Hospital, Osaka, Japan; 18 Late-Stage Development, Cardiovascular, Renal, and Metabolism, BioPharmaceuticals R&D, AstraZeneca, Gothenburg, Sweden

**Keywords:** Outcomes research, Cardiovascular diseases

Correction to: *Nature Medicine* 10.1038/s41591-022-02102-9. Published online 15 December 2022.

In the version of this article initially published, there was an error in the abstract, where in the sentence now reading, in part, “In participants with HFimpEF… first worsening heart failure events (HR = 0.84),” the hazard ratio originally read “0.78,” while in the second paragraph of the “Outcomes by HFimpEF status” Results subsection, in the sentence now reading, in part, “The effect of dapagliflozin on HF outcomes was also similar in those with HFimpEF (HR = 0.84, 95% CI = 0.61–1.14) and those with LVEF consistently over 40% (HR = 0.78),” the hazard ratio values were mistakenly interchanged. The values have been updated in the HTML and PDF versions of the article.

