# Supplementary material for: Dapagliflozin in heart failure with improved ejection fraction: a prespecified analysis of the DELIVER trial
Source: Nat Med. 2022 Dec 15;28(12):2504–11. doi: 10.1038/s41591-022-02102-9 (PMC9800271; doi:10.1038/s41591-022-02102-9)
Supplement: Supplementary file 1 — Supplementary Appendix containing the list of DELIVER committees, national lead investigators, all investigators and sites by country, DELIVER protocol, statistical analysis plan and academic statistical analysis plan. [file 41591_2022_2102_MOESM1_ESM.pdf]

nature medicine

Article

https://doi.org/10.1038/s41591-022-02102-9

Dapagliflozin in heart failure with improved ejection fraction: a prespecified analysis of the DELIVER trial

In the format provided by the authors and unedited

nature medicine

Article

https://doi.org/10.1038/s41591-022-02102-9

Dapagliflozin in heart failure with improved ejection fraction: a prespecified analysis of the DELIVER trial

In the format provided by the authors and unedited

Supplemental Appendix

Table of Contents

DELIVER Committees 2

Steering Committee 2

Sponsor Leadership (AstraZeneca) 2

Data Safety Monitoring Committee 2

Clinical Events Committee 2

Diabetic Ketoacidosis Committee 3

National Lead Investigators 3

List of Investigators and Sites by Country 4

DELIVER Protocol, Statistical Analysis Plan, and Academic Statistical Analysis Plan 13

DELIVER Committees

Steering Committee

Scott D. Solomon, MD (Chair), Brigham and Women's Hospital
John J.V. McMurray, MD, (co-Chair) University of Glasgow
Rudolf A. de Boer, MD, University of Groningen
David DeMets, PHD, University of Wisconsin
Adrian F. Hernandez, MD, Duke University
Silvio E. Inzucchi, MD, Yale University
Mikhail N. Kosiborod, MD, Mid-America Heart Institute
Carolyn S.P. Lam, MD, Duke University National University of Singapore
Felipe Martinez, MD, University of Cordoba
Sanjiv J. Shah, MD, Northwestern University

Sponsor Leadership (AstraZeneca)

Anna Maria Langkilde, MD, PhD (BioPharmaceuticals R&D, AstraZeneca, Gothenburg, Sweden)
Magnus Petersson, MD, PhD (BioPharmaceuticals R&D, AstraZeneca, Gothenburg, Sweden)
Daniel Lindholm, MD, PhD (BioPharmaceuticals R&D, AstraZeneca, Gothenburg, Sweden)
Ulrica Wilderäng, PhD (BioPharmaceuticals R&D, AstraZeneca, Gothenburg, Sweden)
Olof Bengtsson, PhLic (BioPharmaceuticals R&D, AstraZeneca, Gothenburg, Sweden)
Ann Nilsson (BioPharmaceuticals R&D, AstraZeneca, Gothenburg, Sweden)
Barbara Kucharczuk-Filipek (BioPharmaceuticals R&D, AstraZeneca, Warzaw, Poland)

Data Safety Monitoring Committee

Marc Pfeffer,MD, PhD, Brigham and Women's Hospital (Chair)
Stuart Pocock,MD, London School of Hygeine and Tropical Medicine
Karl Swedberg,MD, University of Gothenburg
Jean L. Rouleau,MD, University of Montreal
Nish Chaturvedi,MD, University College London
Peter Ivanovich, MD (deceased), Northwestern University
Andrew Levey,MD, Tufts Medical Center

Clinical Events Committee

Akshay Desai,MD, Brigham and Women's Hospital (Chair)
Pardeep Jhund,MD, University of Glasgow (Co-Chair)
Peter Finn,MD, Brigham and Women's Hospital
Abdel Brahimi,MD, Brigham and Women's Hospital
Martina McGrath,MD, Brigham and Women's Hospital

Ebrahim Barkoudah,MD, Brigham and Women's Hospital
Finnian McCausland,MD, Brigham and Women's Hospital
Eugene Connolly,MD, Universtiy of Glasgow
Ninian Lang,MD, University of Glasgow
Mark Petrie,MD, University of Glasgow

Diabetic Ketoacidosis Committee

Christian Ruff, MD (Chair), Brigham and Women's Hospital
Sara Alexanian, MD, Boston Medical Center
Rajesh K. Garg, MD, University of Miami Miller School of Medicine
Marie E. McDonnell, MD, Brigham and Women's Hospital

National Lead Investigators

Adrian Hernandez, Duke University (Lead National Lead Investigator)

Argentina, Jorge Thierer , Centro de Educación Médica e Investigaciones Clínicas "Norberto Quirno" (CEMIC); Argentina
Belgium , Stefan P. Janssens, University Hospitals Leuven, Belgium
Brazil, Jose Francisco Kerr Saraiva, Cardiovascular Division, Instituto de Pesquisa Clínica de Campinas, Campinas SP, Brazil
Bulgaria, Tsvetana Katova, National Cardiology Hospital, Sofia, Bulgaria
Canada, Subodh Verma, St. Michael's Hospital, University of Toronto
Canada, Eileen O'Meara, Institut de Cardiologie de Montréal, Université de Montréal, Montréal
China, Yaling Han, Cardiovascular Research Institute, General Hospital of Northern Theater Command, Shenyang, China
Czech Rep, Jan Belohlavek, General University Hospital in Prague,
First Faculty of Medicine, Charles University
Hungary, Bela Merkely , Heart and Vascular Center, Semmelweis University, Budapest
Japan, Masafumi Kitakaze, Kinshukai Hanwa Daini Senboku Hospital
Osaka, Japan
Mexico, Marco Antonio Alcocer Gamba, Centro de Estudios Clínicos de Querétaro(CECLIQ), Querétaro, México.
Netherlands, C. Jan Willem Borleffs, HagaZiekenhuis, Department of Cardiology, Els Borst-Eilersplein 275, 2545 AA Den Haag, Netherlands
Peru, Jose Walter Cabrera Honorio, Clínica Vesalio, San Borja, Peru
Poland, Jaroslaw Drozdz, 2nd Department of Cardiology, Central University Hospital, Medical University of Lodz, Łódź, Poland.
Romania, Dan Dobreanu, University of Medicine Pharmacy Science and Technology "G.E. Palade" of Târgu Mures, , Târgu Mures, Romania
Russia, Sergey N. Tereschenko, Department of Myocardial Disease and Heart Failure, National Medical Research Center of Cardiology, Moscow, Russia

Saudi Arabia, Waleed Al Habeeb, Cardiac Sciences Department, King Saud University, Riyadh, Saudi Arabia.
Spain, Josep Comin-Colet, Cardiovascular Diseases Research Group, Bellvitge Biomedical Research Institute (IDIBELL), L'Hospitalet de Llobregat, Barcelona, Spain
Taiwan, Chern-En Chiang, General Clinical Research Center, Division of Cardiology, Taipei Veterans General Hospital and National Yang-Ming University, Taipei, Taiwan.
USA, Jim Fang, Division of Cardiovascular Medicine, University of Utah, Salt Lake City, Utah.
USA, Orly Vardeny, Minneapolis VA Center for Care Delivery and Outcomes Research, University of Minnesota, Minneapolis
Vietnam, Pham Nguyen Vinh, Tam Anh hospital, Vietnam, Tan Tao University, Vietnam

List of Investigators and Sites by Country

Argentina

Juan Loureyro, INSTITUTO CAICI SRL.
Diego Besada, Centro Medico Dra. De Salvo
Eduardo Perna, Instituto de Cardiología de Corrientes J.F.Cabral
Paula Perez Terns, Centro Médico Medeos
Daniela García Brasca, Hospital Italiano de Córdoba
Diego Aizenberg, Centro Médico Viamonte
Guillermo Mercau, Centro Modelo de Cardiologia
Andres Alvarisqueta, Centro de Investigaciones Medicas
Cesar Zaidman, CIPREC
Mariana Ruiz, Centro Médico Luquez
Oscar Montaña, Clínica Privada DIM
Viviana Arias, Sanatorio Junin
Diego Martinez, Fundación Rusculleda
Natalia Cluigt, Instituto de Investigaciones Clínicas Mar del Plata
Miguel Hominal, Centro de Investigaciones Clínicas del Litoral
Sonia Sassone, Centro Medico Dra Laura Maffei-Investigación Clínica Aplicad
Alberto Liberman, Instituto de Investigaciones Clínicas de Córdoba
Claudio Majul, CEMEDIC
Rubén García Durán, Instituto de Investigaciones Clinicas San Nicolas
Fredy Ferre Pacora, Centro Medico Colon

Belgium

Anne Pouleur, Cliniques Universitaires Saint-Luc
Stefan Janssens, UZ Leuven
Rachid Maamar, Centre Hospitalier Régional de Huy
Walter Smolders, Algemeen Ziekenhuis Klina
Philippe Vanduynhoven, Algemeen Stedelijk Ziekenhuis - Campus Aalst
David Derthoo, AZ Groeninge

Brazil

Adrian Paulo Moralles Kormann, MK Blumenau Pesquisa Clínica
Fabio Tuche, Instituto Brasil De Pesquisa Clinica S.A
Renner Lariucci, Dr. Consulta
Mauro Esteves Hernandes, Santa Casa de Misericordia of Votuporanga
Paulo Ernesto Leaes, Irmandade Santa Casa de Misericórdia de Porto Alegre
Dalton Bertolim Precoma, Sociedade Hopitalar Angelina Caron
Maria Helena Vidotti, Loema - Instituto de Pesquisa Clínica
Lidia Zytynski Moura, Núcleo de Pesquisa Clínica da Escola de Medicina da PUCPR
Jose Francisco Kerr Saraiva, Instituto de Pesquisa clinica de Campinas
Fernando Carvalho Neuenschwander, Instituto Orizonti
Adamastor Humberto Pereira, Hospital de Clinicas de Porto Alegre
Gilmar Reis, Cardioresearch Cardiologia Assistencial e de Pesquisa
Wladmir Faustino Saporito, Pesquisare Saude S/S Ltda

Bulgaria

Nikolai Runev, UMHAT Aleksandrovska EAD
Nikolay Iliev, MHAT Sveti Pantaleymon - Pleven OOD, Pleven
Yuliyana Ivanova, DCC "Focus 5 - LZIP EOOD
Atanas Mihov, MHAT "Sveta Ekaterina"
Maria Milanova, UMHATEM N. I. Pirogov
Tsvetelina Damyanova, MC Synexus Sofia EOOD
Vladimir Zhelev, MC Synexus Sofia EOOD, Branch Stara Zagora
Antoni Gogov, MHAT Haskovo AD
Dimitar Karageorgiev, MHAT Sveta Karidad EAD
Georgi Stanchev, MHAT Pazardzhik AD
Daniela Tsonkova-Dobreva, Medical centers Fi Health AD
Tsvetana Katova, MHAT National Cardiology Hospital EAD
Ralitsa Vatova, Medical Center - Zdrave1
Danail Avramov, MHAT National Cardiology Hospital EAD

Canada

Timothy Salter, LMC Manna Research Burlington North
Ying Tung Sia, CIUSSS de la Mauricie-et-du-Centre-du-Québec
Subodh Verma, North York Diagnostic and Cardiac Centre
Ram Vijayaraghavan, Heart Health Institute
Helene Mayrand, Cite de la Sante de Laval
Shelley Zieroth, Saint Boniface General Hospital
Dany El-Ghassan, CISSS de Lanaudiere - Hopital Pierre Le Gardeur
Andrew Yan, Unity Health Toronto - St. Michael's Hospital
Milan Gupta, Brampton Clinical Trials
Sewa Singal, Sewa Ram Singal Medicine Professional Corporation
Laurie Hill, Peel Research Associates
Yaariv Khaykin, Partners of Advanced Cardiac Evaluation (PACE)
Shamir Mehta, Hamilton Health Sciences General Site

Yves Pesant, Centre intégré de santé et de services sociaux des Laurentides
Louis Yao, The Office of Dr. Louis Yao
Luis Noronha, Diabetes Heart Research Centre
James Cha, Dr James Cha
Amritanshu Pandey, Cambridge Cardiac Care Centre
Richard Choi, Riverside Cardiology and Diagnostic Imaging
Mohan Babapulle, CPS Research
John Stewart, Medical Associates of Port Perry
Eileen O'Meara, Institut de Cardiologie de Montréal

China

Ye Gu, Puai Hospital of Wuhan
Lihong Lu, The Fujian Provincial Hospital
Xueya Guo, Lanzhou University Second Hospital
Yingzi Liang, Tianjin Medical University Affiliated 2nd Hospital
Jinqiu Liu, First Affiliated Hospital of Dalian Medical University
Yundai Chen, Chinese PLA general hospital
Jingfeng Wang, Sun Yat-sen Memorial Hospital, Sun Yat-Sen University
Zeqi Zheng, 1st Affiliated Hospital of Nanchang University
Wenxia Zong, Hubei Zhongshan Hospital
Yu Song, Teda International Cardiovascular Hospital
Xiaoli Zhang, Zhongda Hospital-Southeast University
Qiang Zhao, Ruijin Hospital, Shanghai Jiao Tong University, School of Medicine
Jun Zhang, Cangzhou central hospital
Hui Li, Daqing Oilfield General Hospital
Zuyi Yuan, The First Affiliated hospital of Xi'an Jiaotong University
Daying Wang, Shanghai Putuo District Center Hospital
Biao Xu, Nanjing Drum Tower Hospital
Jianzeng Dong, Beijing Anzhen Hospital, Capital Medical University
Mingzhi Long, The Second Affiliated Hospital of Nanjing Medical University
Ling Wu, General Hospital of Ningxia Medical University
Xin Qi, Tianjin People's hospital
Zhurong Luo, 900 Hospital of the Joint Logistics Team
Chunhua Ding, The Central Hospital of China Aerospace
Guohai Su, Jinan Central Hospital
Yong He, West China Hospital of Sichuan University
Xuelian Zhang, Jilin Province People's Hospital
Wenjun Huang, The Jiangxi Pingxiang People's Hospital
Feng Guo, Chinese PLA Joint Logistic support foce No.983 Hospital
Yimeng Zhou, Shanghai Yangpu District Central Hospital
Xuefeng Lin, The First Affiliated Hospital of Baotou Medical College, Inn
Dongfang W
ang, Chifeng Hospital
Ping Yang, China-Japan Union Hospital of Jilin University
Jiyan Chen, Guangdong Provincial People's Hospital
Lu Fu, 1st Affiliated Hospital of Haerbin Medical University

Yaling Han, The General Hospital of Shenyang Military Command

Czech Republic

Ondrej Jerabek, Kardiologicka ambulance s.r.o.
Jan Belohlavek, Vseobecna fakultni nemocnice, 3. interni klinika
Dusan Kucera, Pedicor Care s.r.o.
Jiri Parenica, Kardiologicka ambulance Brno s.r.o.
Jiri Krupicka, MEDICUS SERVICES Ltd.
Roman Kuchar, Lekarna Innera s.r.o.
Jan Matejka, Nemocnice Pardubickeho kraje a s
Jiri Vesely, Edumed s.r.o.
Petr Povolny, Cardiocentrum Kladno s.r.o.
Jiri Vesely, Edumed s.r.o.
Ladislav Busak, KardioBusak s.r.o.

Hungary

Ebrahim Noori, Fejér Megyei Szent György Egyetemi Oktató Kórház
Andrea Hornyik, CRU Hungary Kft
László Nagy, Csongrád-Csanád Megyei Dr Bugyi István Kórház
András Motyovszki, MOPA-MED Kft. Háziorvosi Rendelő
Anna Czigány, XIII kerületi Egészségügyi Szolgálat Közhasznú Nonprofit Kft
Csaba Király, Bács-Kiskun Megyei Kórház
Béla Merkely, Semmelweis Egyetem AOK
Csaba András Dézsi, Petz Aladár Egyetemi Oktató Kórház - Győr
Zsolt Zilahi, MediFarma-98 Kft.
Béla Benczur, Tolna Megyei Balassa János Kórház
Miklós Kajetán, Strázsahegy Medicina Háziorvosi Rendelő
Béla Herczeg, Jász-Nagykun-Szolnok Megyei Hetényi Géza Kórház-Rendelőintézet

Japan

Atsushi Kawasaki, National Hospital Organization Mie Chuo Medical Center
Takashi Takenaka, NHO Hokkaido Medical Center
Keiki Yoshida, Saga-Ken Medical Centre Koseikan
Shujiro Inoue, Iizuka Hospital
Daisuke Maebuchi, Shin-Kuki General Hospital
Akihito Sasaki, Nishiarai Heart Central Clinic
Kenjiro Kitasato, Chiba Tokushukai Hospital
Masatoshi Shimizu, National Hospital Organization Kobe Medical Center
Masato Ohnishi, NHO Higashi-Ohmi General Medical Center
Masaharu Akao, National Hospital Organization Kyoto Medical Center
Harukazu Iseki, Agricultural Cooperatives, Sagamihara Kyodo Hospital
Shinichiro Uchikawa, Japanese Red Cross Society Azumino Hospital
Yasuaki Koga, Oita Prefectural Hospital
Hiroyuki Takase, JA Shizuoka Koseiren Enshu Hospital
Chisato Izumi, National Cerebral and Cardiovascular Center Hospital

Takeshi Akasaka, Shonan Fujisawa Tokushukai Hospital
Shintaro Akashi, National Hospital Organization Hamada Medical Center
Arihiro Kiyosue, Tokyo Eki Center-Building Clinic
Takuro Takagi, Tokyo Shinagawa Hospital Medical Corp. Tokyokyojuno-kai
Satoru Sakagami, National Hospital Organization Kanazawa Medical Center
Mitsuhiro Shimomura, National Hospital Organization Ureshino Medical Center
Hiroshi Sugino, National Hospital Organization Kure Medical Center
Yoshiki Hata, Minamino Cardiovascular Hospital
Masanori Asakura, Hyogo College of Medicine Hospital
Shinichi Higashiue, Kishiwada Tokushukai Hospital
Tetsuo Sakai, Koukan Clinic
Noritaka Fujimoto, National Hospital Organization Beppu Medical Center
Junji Kanda, Asahi General Hospital
Koichiro Kuwahara, Shinshu University Hospital
Yusuke Katayama, National Hospital Organization Iwakuni Clinical Center
Wataru Takahashi, National Hospital Organization Shinshu Ueda Medical Center
Masaya Shinbo, Tokushukai Takasago Seibu Hospital
Kengo Tsukahara, Fujisawa City Hospital
Eijiro Hattori, JA Toride Medical Center
Yuichiro Takagi, Federation of National Public Service Takamatsu Hospital
Hideki Ueno, Medical corporation tokushukai, HAYAMA HEART CENTER
Ryosuke Kametani, Nagoya Tokusyukai General Hospital
Motoaki Higuchi, Mito Saiseikai General Hospital
Toru Kato, National Hospital Organization Tochigi Medical Center
Masataka Fukue, Japanese Red Cross Koga Hospital
Tsunekazu Kakuta, Tsuchiura Kyodo General Hospital
Nobuyoshi Higa, Tokushukai Medical Corporation Chubu Tokushukai Hosp
Tomohiko Teramoto, Ichinomiyanishi Hospital

Mexico

José Garza Ruíz, IMED Clin Trials
Víctor González López, Centro Especializado de Investigación Medical Group
Vicente Ruiz Ruiz, Clinstile S.A de C.V
Melchor alpizar salazar, Centro Esp en Diabetes y Obes y Prev de Enf CV´s
Edmundo Bayram Llamas, Hospital Cardiologica Aguascalientes
Guillermo Rivera Martinez, Centro Cardiológico Integral
Elias Garcia Cantu, CardioLink Clin Trials
Mauricio Zaragoza Pérez, ARKE Estudios Clínicos
Gabriel Ramos Lopez, Private Clinic Dr. Gabriel Ramos
Jorge Echeverri Rico, Sociedad de Metabolismo y Corazón, S.C.
Marco Alcocer Gamba, Centro de Estudios Clínicos de Queretaro S.C.
Guillermo Hernández Llamas, Estudios Clínicos de Vanguardia
Carlos Hernandez Herrera, Instituto Cardiovascular de Monclova, S de RL de CV

Netherlands

Rudolf de Boer, Universitair Medisch Centrum Groningen

Marco Van Gent, Albert Schweitzer Ziekenhuis
Reginald Groutars, Onze Lieve Vrouwe Gasthuis
Maria (Mireille) Emans, Ikazia Ziekenhuis
Cornelis (Ron) de Nooijer, Maxima Medisch Centrum
Ruud van de Wal, Bernhoven Ziekenhuis
Tjeerd Römer, Alrijne Ziekenhuis Leiderdorp
Jan Willem Borleffs, HAGA Ziekenhuis locatie Els Borst-Eilersplein
Bastiaan Kietselaer, Zuyderland Medisch Centrum - Heerlen
Pieter Hoogslag, Isala Diaconessenhuis
Ron Pisters, Rijnstate Ziekenhuis

Peru

Armando Godoy, Instituto Nacional del Corazon EsSalud
Luisa Cardoza, Centro de Investigación Clínica Belén
Helard Manrique, Centro de Expertos en Diabetes, Obesidad y Nutricion
Victor Sanchez, Hospital de Chancay
Hugo Arbanil, Hospital Nacional Dos de Mayo
Walter Cabrera, Clinica Vesalio
Angelica Valdivia, Hospital Militar Geriático
Roger Correa, Clinica Providencia
Henry Anchante, Hospital Nacional Cayetano Heredia
Aldo Rodriguez, Hospital Alberto Sabogal Sologuren - ESSALUD
Carlos Chavez, Hospital Daniel Alcides Carrion
Elizabeth Garrido Carrasco, Clinica El Golf
Victor Rodriguez, Hosp Central de la Fuerza Aerea de Peru

Poland

Krzysztof Cymerman, Indywidualna Specjalistyczna Praktyka Lekarska w Dz.Kard.
Michał Ploch, Optima Medycyna S.A.
Lidia Pawłowicz, Praktyka Lekarska Lidia Pawlowicz
Bernadeta Gogola-Migdał, Centrum Usług Medycznych MaxMed Marek Maciaszek
Romuald Korzeniak, MEDIKARD Specjalistyczna Przychodnia Lekarska
Stanisław Mazur, Centrum Medyczne Medyk
Jacek Lampart, Centrum Medyczne Pratia Katowice
Paweł Miękus, NZOZ „PRO CORDIS" Sopockie Centrum Badań Kardiologicznych
Adam Mądrzejewski, Centrum Medyczne Lukamed Joanna Łuka-Wendrowska
Sławomir Szynal, Sławomir Szynal Indywidualna Praktyka Lekarska
Robert Witek, Poradnia Chorób Metabolicznych w Wierzchoslawicach
Andrzej Szuba, 4 Wojskowy Szpital Kliniczny z Polikliniką SPZOZ
Grzegorz Drelich, CenterMed Lublin Sp. z o. o.
Jarosław Drożdż, SPZOZ Centralny szpital Kliniczny Uniwersytetu Medycznego w Łodzi
Jaroslaw Jurowiecki, Poradnia Kardiologiczna Jarosław Jurowiecki
Ryszard Ściborski, Zespół Opieki Zdrowotnej w Oławie SP ZOZ
Anna Lisowska, PANEKMED Indywidualna Praktyka Lekarska Bogusław Panek

Romania

Sirona Lupu, SC CARDIOMED SRL
Ella Pintilei, Consultmed SRL
Cristian Podoleanu, Cabinet Medical de Cardiologie si Medicina Interna
Constantin Militaru, Spitalul Clinic Judetean de Urgenta Craiova
Octavian ISTRATOAIE, Spitalul Clinic Judetean de Urgenta Craiova

Russian Federation

Dmitrii Pevzner, Russian Cardiology Research and Manufacturing Complex
Zhanna Kobalava, Moscow, City Hospital No. 64
Yury Didenko, St.Petersburg City Multiservice Hospital #2
Svetlana Boldueva, North-Western State Medical University n.a. I. I. Mechnikov
Elena Privalova, First MSMU n.a. I.M.Sechenov based on University clin hosp 1
Polina Ermakova, Ltd "Astarta"
Sergey Tereshchenko, Russian Cardiology Research and Manufacturing Complex
Oleg Averkov, City Clinical Hospital #15 n.a. Filatov
Natalya Krasnopeeva, Chelyabinsk Road Hospital of RRW
Sergei Aksentiev, Ryazan Regional Clinical Hospital
Alexey Maltcev, Consultative and Diagnostic Center 85
Tatiana Medina, PCC FGBUZ FMBA of Russia
Alexey Nesmachnyy, Meshalkin Research Institute of Blood Circulation Pathology
Nino Dzhaiani, Moscow, City Hospital # 68
Andrei Kazakov, Tver Regional Hospital

Saudi Arabia

Waleed Al-Habeeb, King Khalid University Hospital
Abdulmajid Mukhtar, King Abdulaziz Medical City
Ammar Chaudhary, King Faisal Specialist Hospital - Jeddah
Kamal AlGhalayini, King Abdulaziz University Hospital - Jeddah
Ali Youssef, Saud Al-Babtain Cardiac Center
Abdulrahman Al Qahtani, King Fahad Medical City

Spain

Rafael Jesús Hidalgo Urbano, Hospital Universitario Virgen Macarena
Nuria Farré López, Hospital del Mar
Josep Comín Colet, Hospital Universitàrio de Bellvitge
Alfonso Soto Gonzalez, Complejo Hospitalario Universitario A Coruña
Ramón Bover Freire, Hospital Clinico Universitario San Carlos
Manuel Beltrán Robles, Hospital Virgen del Camino
Luis Almenar Bonet, Hospital Universitari i Politecnic La Fe de Valencia
José Ramón González Juanatey, Complejo Hospitalario Universitario de Santiago (CHUS)
Javier Salvador de Juan Bagudá, Hospital Universitario 12 de Octubre
Enrique García del Río, Hospital Virgen de las Montañas

Julio Eduardo Núñez Villota, Hospital Clínico Universitario de Valencia

Taiwan

Kwo-Chang Ueng, Chung Shan Medical University Hospital
Tsung-Hsien Lin, Kaohsiung Medical University Hospital
Pao-Hsien Chu, Chang Gung Medical Foundation Linkou Branch
Chun-Yao Huang, Taipei Medical University Hospital
Hung-Yu Chang, Cheng Hsin General Hospital
Chern-En Chiang, Veteran General Hospital Taipei
Jin-Long Huang, Veterans General Hospital Taichung
Ming-En Liu, MacKay Memorial Hospital-HsinChu
Ting-Hsing Chao, National Cheng-Kung University Hospital
Wei-Hsiang Lin, Triple Service General Hospital
Tzung-Dau Wang, National Taiwan University Hospital
Kuan-Cheng Chang, China Medical University Hospital
Thung-Lip Lee, E-DA Hospital

United States of America

Julian Javier, Advanced Research for Health Improvement, LLC
Sheila Hegde, Brigham and Women's Hospital
David Murray, William S. Middleton Memorial Veterns Hospital
Rajat Barua, VA Medical Center - Kansas City
Parag Goyal, New York-Presbyterian/Weill Cornell Medical Center
Michael Pursley, Heart Group of the Eastern Shore PC
Preetham Jetty, Community Clinical Research Center
Raymond Little, North Houston Heart & Vascular Associates
Alexander Schabauer, Monument Health Rapid City Hospital, Inc
Ronald Fernando, VA Loma Linda Healthcare System Pulmonary
Todd Lewis, East Coast Institute for Research, LLC
Ravi Bhagwat, Cardiology Assoc of NW Indiana
Freny Mody, VA Greater Los Angeles Healthcare System/West LA VA Medical
Gaurav Choudhary, Providence VA Medical Center
Barry Borlaug, Mayo Clinic
Subash Bazaz, Virginia Heart
Christopher Brown, Mobile Heart Specialists PC
Henry Ooi, VA Tennessee Valley Healthcare System
Joseph Izzo, Erie County Medical Center Hospital
Joe Hargrove, Cardiology and Medicine Clinic
Renee Sangrigoli, Doylestown Health Physicians
Vishweshwar Ranga, Sierra Clinical Research
Cezar Staniloae, NYU Langone Medical Associates
Mark Napoli, Clinical Trials of America Inc
Peter Van Buren, University of Vermont Medical Center
Viorel Florea, Minneapolis VA Medical Center
Syed Ahmed, McLaren Regional Medical Center
Jorge Castriz, Great Falls Clinic, LLP

Nikhil Joshi, Sherman Clinical Research
Mark Fasulo, DiscoveResearch Inc
Leslie Forgosh, HealthEast St Josephs Hospital
Antwan Robinson, The Jackson Clinic
Matthew Janik, PMG Research of Wilmington
Ahmed Arif, Apex Medical Research of Flint PC
Christopher Bianco, West Virginia University School of Medicine
Mayank Kansal, Jesse Brown VA Medical Center
James Liu, OnSite Clinical Solutions LLC
Mohammad Tahir, Saint Louis Heart and Vascular
Modele Ogunniyi, Emory University
Diego Torres, Ormond Beach Clinical Research
Preethi William, Banner University of Arizona Medical Center
John Blair, The University of Chicago
Dinesh Gupta, Tennessee Center for Clinical Trials
Sharan Mahal, Advanced Heart Care, LLC
Shahab Ghafghazi, University of Louisville Hospital
Jagadeesh Ganji, Medication Management, LLC
Gregory Lewis, Massachusetts General Hospital
David Hotchkiss, Charlotte Cardiovascular Institute
Daniel Lee, Bay Regional Medical Center
Dwayne Schmidt, Hightower Clinical
Akbar Nikfarjam, Sanitas Research
Albert Ridgeway, Syed Research Consultants, LLC
John Agaiby, Clinical Investigation Specialists, Inc.
Norman Lepor, Westside Medical Associates of Los Angeles
Lauren Gilstrap Milley, Dartmouth Hitchcock Medical Center
Sheldon Litwin, Medical University of South Carolina IRB
Jay Amin, Clearwater Cardiovascular Interventional Consultants
Peter Fail, Cardiovascular Institute of the South
Thomas Baumgarten, Clinical Research Associates of Central PA LLC
Michael Nassif, St Luke's Hospital
Edward Portnay, Cardiology Associates of Fairfield County, P.C.
Jeffrey Whittle, Zablocki VA Medical Center
Lan Luo, Ocala Cardiovascular Research
Tariq Haddad, Virginia Heart
Ghiath Mikdadi, Heart Clinic of Hammond, LLC
Sumeet Mitter, Mount Sinai

Vietnam

Hai Nguyen, Gia Dinh People Hospital
Huan Do, HCMC Heart Institute
Hien Nguyen, Saint Paul Hospital
Dung Ho, Thong Nhat Hospital
Nga Vu, Hanoi Heart Hospital- Trần Hưng Đạo
Minh Ton, Tam Duc hospital
Hien Nguyen, Hanoi Heart Hospital- Trần Hưng Đạo

DELIVER Protocol, Statistical Analysis Plan, and Academic Statistical Analysis Plan

**Clinical Study Protocol**	
Drug Substance	Dapagliflozin
Study Code	D169CC00001
Version	1.0
Date	24th April 2018

An International, Double-blind, Randomised, Placebo-Controlled Phase III Study to Evaluate the Effect of Dapagliflozin on Reducing CV Death or Worsening Heart Failure in Patients with Heart Failure with Preserved Ejection Fraction (HFpEF)

DELIVER - Dapagliflozin Evaluation to Improve the LIVEs of Patients with PReserved Ejection Fraction Heart Failure

Sponsor: AstraZeneca AB, 151 85 Södertälje, Sweden

Regulatory Agency Identifying Number(s):

VERSION HISTORY

**Version 1.0, 24th April 2018**
Initial creation

This Clinical Study Protocol has been subject to a peer review according to AstraZeneca Standard procedures. The Clinical Study Protocol is publicly registered and the results are disclosed and/or published according to the AstraZeneca Global Policy on Bioethics and in compliance with prevailing laws and regulations.

TABLE OF CONTENTS

TITLE PAGE 1
VERSION HISTORY 2
TABLE OF CONTENTS 3
1. PROTOCOL SUMMARY 8
1.1 Schedule of Activities (SoA) 8
1.2 Synopsis 12
1.3 Schema 16
2. INTRODUCTION 17
2.1 Study rationale 17
2.2 Background 18
2.3 Benefit/risk assessment 18
2.3.1 Potential risks to patients 19
2.3.1.1 Protection against risks 19
2.3.2 Potential benefits to patients 20
2.3.3 Conclusion 20
3. OBJECTIVES AND ENDPOINTS 20
4. STUDY DESIGN 22
4.1 Overall design 22
4.2 Scientific rationale for study design 22
4.3 Justification for dose 24
4.4 End of study definition 25
5. STUDY POPULATION 25
5.1 Inclusion criteria 25
5.2 Exclusion criteria 26
5.3 Lifestyle restrictions (not applicable) 28
5.4 Screen failures 28
5.5 Procedures for handling of randomized not eligible patients 28
6. STUDY TREATMENTS 29
6.1 Treatments administered 29
6.2 Preparation/handling/storage/accountability 29
6.3 Measures to minimise bias: randomisation and blinding 30
6.3.1 Stratification and capping 30
6.3.1.1 Stratification 31

6.3.1.2 Capping 31

6.4 Treatment compliance 31

6.5 Concomitant therapy 31
6.5.1 Prohibited medication........ 31
6.5.2 Recording of concomitant treatment 32
6.5.3 Heart failure background standard of care 32
6.5.4 Anti-diabetes treatment 32
6.5.4.1 Background 32
6.5.4.2 Treatment of patients with established diagnosis of type 2 diabetes 32
6.5.5 Other concomitant treatment 32

6.6 Dose modification (not applicable) 32

6.7 Treatment after the end of the study 32

7. DISCONTINUATION OF TREATMENT AND SUBJECT WITHDRAWAL .. 33

7.1 Discontinuation of study treatment 33
7.1.1 Temporary discontinuation 33
7.1.1.1 Unexpected acute declines in eGFR 33
7.1.1.2 Volume depletion/hypotension 33
7.1.1.3 Patients at risk of volume depletion 34
7.1.2 Procedures for discontinuation of study treatment 34

7.2 Lost to follow-up 34

7.3 Withdrawal from the study 35

8. STUDY ASSESSMENTS AND PROCEDURES 35

8.1 Enrolment Period 36
8.1.1 Visit 1, Enrolment (Day -21 to Day -1) 36

8.2 Treatment period 37
8.2.1 Visit 2, Randomisation (Day 1) 37
8.2.2 Visit 3 (Day 30; ±7 days): 37
8.2.3 Visit 4 (Day 120 ±7 days): 38
8.2.4 Visit 5 (Day 240 ±7 days) 38
8.2.5 Visit 6 (Day 360 ±7 days) 39
8.2.6 Visit 7 and onwards (Day 480 and every 120 days ±14 days) 39
8.2.7 Premature Treatment Discontinuation Visit 40
8.2.8 Study Closure Visit 40
8.2.9 Unscheduled visits 41

8.3 Efficacy assessments 41
8.3.1 Efficacy event capture 41
8.3.2 Efficacy event adjudication 41
8.3.3 Clinical Outcome Assessments (COA) 42
8.3.3.1 KCCQ 42
8.3.3.2 Patient Global Impression of Severity (PGIS) 43
8.3.3.3 EuroQoL five-dimensional five-level questionnaire (EQ-5D-5L) 43

8.3.3.4 Administration of electronic PROs 43
8.4 Safety assessment 44
8.4.1 Physical examinations 44
8.4.2 Vital Signs 44
8.4.3 Electrocardiogram 44
8.4.4 Safety laboratory assessments 45
8.4.5 Other safety assessments (not applicable) 45
8.4.6 Other clinical assessments 45
8.4.6.1 Body weight and height 45
8.5 Collection of adverse events 45
8.5.1 Method of detecting AEs and SAEs 45
8.5.1.1 Adverse events (AEs) leading to amputation and potential risk factor AEs for amputations affecting lower limbs ("preceding events") 45
8.5.1.2 Capture of DKA events 46
8.5.1.3 DKA definition 46
8.5.1.4 Capture of cardiac ischaemic events and stroke 46
8.5.1.5 Capture of additional laboratory values 46
8.5.2 Time period and frequency for collecting AE and SAE information 46
8.5.3 Follow-up of AEs and SAEs 47
8.5.4 Adverse event data collection 47
8.5.5 Causality collection 48
8.5.6 Adverse events based on signs and symptoms 48
8.5.7 Adverse events based on examinations and tests 48
8.5.8 Disease-under study (DUS) (not applicable) 48
8.5.9 Disease progression (not applicable) 48
8.6 Safety reporting and medical management 48
8.6.1 Reporting of serious adverse events 48
8.6.1.1 Reporting of SAEs considered to be potential endpoints 49
8.6.2 Pregnancy 49
8.6.2.1 Maternal exposure 50
8.6.3 Overdose 50
8.7 Pharmacokinetics (not applicable) 50
8.8 Pharmacodynamics (not applicable) 50
8.9 Optional exploratory genetics 51
8.10 Biomarkers (not applicable) 51
8.11 Health Economics (not applicable) 51
9. STATISTICAL CONSIDERATIONS 51
9.1 Statistical hypotheses 51
9.2 Sample size determination 51
9.3 Populations for analyses 52
9.4 Statistical analyses 52

9.4.1 Efficacy analyses 53
9.4.1.1 Analysis of the primary variable 53
9.4.1.2 Analysis of the secondary variables 53
9.4.1.3 Subgroup analysis 53
9.4.1.4 Sensitivity analysis 54
9.4.2 Safety analyses 54
9.4.3 Methods for multiplicity control 54
9.5 Interim analyses 54
9.5.1 Data monitoring committee (DMC) 55
10. REFERENCES 56
11. SUPPORTING DOCUMENTATION AND OPERATIONAL CONSIDERATIONS 61

LIST OF TABLES

Table 1 Study of Assessments 9
Table 2 Objectives and Endpoints 13
Table 3 Study objectives 20
Table 4 Study Treatments 29
Table 5 Population for analysis 52

LIST OF FIGURES

Figure 1 Study design 16

LIST OF APPENDICES

Appendix A Regulatory, ethical and study oversight considerations 61
Appendix B Adverse event definitions and additional safety information 65
Appendix C Cardiovascular related events 69
Appendix D Genetics 72
Appendix E Handling of Human Biological Samples 76
Appendix G Actions required in cases of increases in liver biochemistry and evaluation of Hy’s Law (not applicable) 78

Appendix H Medical device incidents: definition and procedures for recording, evaluating, follow-up, and reporting (not applicable) 79
Appendix I Abbreviations 80
Appendix J New York Heart Association (NYHA) Functional Classification........... 82
Appendix K The KC Cardiomyopathy Questionnaire 83
Appendix L EQ-5D-5L Questionnaire 88
Appendix M Patient Global Impression of Severity for Heart Failure Symptoms 91

1. PROTOCOL SUMMARY

1.1 Schedule of Activities (SoA)

Table 1 Study of Assessments

**Visit**	**1 Enrolment**	**2 Randomisation**	**3**	**4**	**5**	**6**	**7 – onwards**	**Premature Treatment Discontinuation Visit**	**Study Closure Visit**	**For details see Section:**
**Day/Month**	**Day -21 to Day -1**	**Day 1**	**Day 30 (±7)**	**Day 120 (±7)**	**Day 240 (±7)**	**Day 360 (±7)**	**Day 480 - onwards (every 120 days ±14 days)**		**≤ 6 weeks after PACD**	
Informed consent	X[6]									5.1, A 3
Inclusion/exclusion criteria	X	X								5.1, 5.2
Demographics	X									5.15.1
Medical history	X	X								5.15.1
Concomitant medication		X	X	X	X	X	X	X	X	6.5
Cardiac and HF related procedures			X	X	X	X	X	X	X	8.5.1.4
Physical exam	X	X							X	8.4.1
Systolic and diastolic BP	X	X	X			X	X[3]	X	X	5.2, 8.4.2
Pulse	X	X	X			X	X[3]	X	X	5.2, 8.4.2
Weight	X					X	X[3]	X	X	8.4.6.1
Height	X									8.4.6.1
NYHA classification	X	X	X	X	X			X	X	5.1, Appendix J
12-lead ECG	X									8.4.3

**Visit**	**1 Enrolment**	**2 Randomisation**	**3**	**4**	**5**	**6**	**7 – onwards**	**Premature Treatment Discontinuation Visit**	**Study Closure Visit**	**For details see Section:**
**Day/Month**	**Day -21 to Day -1**	**Day 1**	**Day 30 (±7)**	**Day 120 (±7)**	**Day 240 (±7)**	**Day 360 (±7)**	**Day 480 - onwards (every 120 days ±14 days)**		**≤ 6 weeks after PACD**	
C-lab NT-proBNP	X									5.1
C-lab eGFR (creatinine)	X		X	X		X	X[3]			5.2, 8.4.4
C-lab HbA1c	X									6.3.1.1
Sample for genetic research, if applicable[5]		X								Appendix D
KCCQ		X[4]	X[4]	X[4]	X[4]			X[4]	X[4]	8.3.3.1
PGIS		X[4]	X[4]	X[4]	X[4]			X[4]	X[4]	8.3.3.2
EQ-5D-5L		X[4]			X[4]			X[4]	X[4]	8.3.3.3
Local pregnancy test (female patients with childbearing potential only)		X								5.1
Randomisation		X								8.2.1
Dispense investigational product (IP)		X		X	X	X	X			6
Collect unused IP; check IP compliance				X	X	X	X	X	X	6

Visit	1 Enrolment	2 Randomisation	3	4	5	6	7 – onwards	Premature Treatment Discontinuation Visit	Study Closure Visit	For details see Section:
Day/Month	Day -21 to Day -1	Day 1	Day 30 (±7)	Day 120 (±7)	Day 240 (±7)	Day 360 (±7)	Day 480 - onwards (every 120 days ±14 days)		≤ 6 weeks after PACD	
Efficacy events (death and worsening heart failure) [1]		X[1]	X	X	X	X	X	X	X	8.3
Safety events[2]	X	X	X	X	X	X	X	X	X	8.4

AEs Adverse events; DAEs Adverse events leading to discontinuation of investigational product; PACD Primary Analysis Censoring Date; SAEs Serious adverse events; C-lab Central laboratory

[1] Efficacy events are considered as endpoints from time of randomisation and throughout the study. Prior to randomisation, these events are considered as SAEs.

[2] SAEs will be recorded from the time of informed consent. DAEs and Amputations, adverse events (AEs) leading to amputation and potential risk factor AEs for amputations affecting lower limbs will be recorded from Visit 2 onwards.

[3] Assessments to be repeated every 12 months.

[4] Will be administered using a site-based electronic device. It is preferred that PRO questionnaires are completed prior to any other study procedures and before discussion of disease progression to avoid biasing the patient's responses to the questions

[5] Blood sample for future genetic research is optional. The genetic sampling is subject to separate consent by the patient.

[6] The Patient signs the ICF. Patients who agree to the optional sampling of blood for genetic research will provide their consent.

1.2 Synopsis

International coordinating Investigator

Scott D. Solomon, MD

The Edward D. Frohlich Distinguished Chair
Professor of Medicine
Harvard Medical School
Senior Physician
Brigham and Women´s Hospital
75 Francis Street
Boston, MA 02115
US

Protocol Title:

An International, Double-blind, Randomised Placebo-Controlled Phase III Study to Evaluate the Effect of Dapagliflozin on Reducing CV Death or Worsening Heart Failure in Patients with Heart Failure with Preserved Ejection Fraction (HFpEF).

Rationale:

The prevalence of chronic heart failure (HF) continues to increase globally, and the annual global economic burden (several hundred billion dollars in 2012) will increase as the population ages. Approximately half of all heart failure patients have heart failure with preserved ejection fraction (HFpEF) representing a particularly significant unmet need given that no approved pharmacotherapy exists specifically for this condition. Patients with HFpEF generally receive diuretic treatment for symptom relief, and should receive guideline recommended therapies for concomitant diseases such as hypertension. Recent data from cardiovascular (CV) outcome trials of SGLT2 inhibitors (empagliflozin and canagliflozin) and real world studies (including patients treated with dapagliflozin) indicate that treatment with SGLT2 inhibitors can reduce the risk of CV death and hospitalisation due to HF in patients with Type 2 Diabetes (T2D) overall and in patients with T2D and concomitant HF. Limitations associated with the randomised clinical trials as well as the observational studies are that only patients with T2D were studied, and that the proportion of patients with HFrEF and HFpEF, respectively, is unknown. This study will test the hypothesis that dapagliflozin will reduce the composite of CV death and HF events (hospitalisation for HF or urgent HF visit) in patients with HF and preserved systolic function (LVEF >40%), with or without T2D.

Table 2 Objectives and Endpoints

**Primary objective:**	**Endpoint/variable:**
To determine whether dapagliflozin is superior to placebo, when added to standard of care, in reducing the composite of CV death and HF events (hospitalisation for HF or urgent HF visit) in patients with HF and preserved systolic function.	Time to the first occurrence of any of the components of this composite:<br>1. CV death<br>2. Hospitalisation for HF<br>3. Urgent HF visit (e.g., emergency department or outpatient visit)
**Secondary objective:**	**Endpoint/variable:**
To determine whether dapagliflozin is superior to placebo in reducing the total number of recurrent HF hospitalisations and CV death	Total number of (first and recurrent) hospitalisations for HF and CV death
To determine whether dapagliflozin is superior to placebo in improving Patient Reported Outcomes measured by KCCQ	Change from baseline in the total symptom score (TSS) of the KCCQ at 8 months
To determine whether dapagliflozin is superior to placebo in reducing the proportion of patients with worsened NYHA class	Proportion of patients with worsened NYHA class from baseline to 8 months
To determine whether dapagliflozin is superior to placebo in reducing all-cause mortality	Time to the occurrence of death from any cause
**Safety objective:**	**Endpoint/variable:**
To evaluate the safety and tolerability of dapagliflozin compared to placebo in patients with HFpEF	Serious adverse events (SAEs), adverse events leading to treatment discontinuation (DAEs), amputations, adverse events (AEs) leading to amputation and potential risk factor AEs for amputations affecting lower limbs
**Exploratory Objective:**	**Endpoint/Variable:**
To determine whether dapagliflozin is superior to placebo in reducing all-cause hospitalisation	Time to the first occurrence of hospitalisation from any cause
To compare the effect of dapagliflozin versus placebo on health status assessed by EuroQol five-dimensional five-level questionnaire (EQ- 5D-5L) to support health economic analysis and health technology assessment	Changes in health status measured by EQ-5D-5L

To compare the effect of dapagliflozin versus placebo on health status assessed by Patient global impression of severity (PGIS) questionnaires	Changes in health status measured by PGIS
To determine whether dapagliflozin compared with placebo will have an effect on systolic BP	Change in systolic BP from baseline
To determine whether dapagliflozin compared with placebo will have an effect on body weight	Change in body weight from baseline
To determine whether dapagliflozin compared with placebo will have an effect on eGFR.	Change in eGFR from baseline
To explore whether dapagliflozin compared to placebo improves KCCQ summary scores, subscores of TSS (Symptom frequency and symptom burden) and domains	Change in Clinical summary score, TSS sub-scores, Overall summary score, QoL score
To collect and store blood samples for future exploratory genetic research	Not applicable. Results will be reported separately

BP Blood pressure; CV Cardiovascular; EQ-5D-5L EuroQol five-dimensional five-level questionnaire
HF Heart failure; HFrEF Heart failure with reduced ejection fraction; KCCQ Kansas City Cardiomyopathy Questionnaire NYHA New York Heart Association

Overall design:

This is an international, multicentre, parallel-group, event-driven, randomised, double-blind study in patients with HFpEF, evaluating the effect of dapagliflozin 10 mg versus placebo, given once daily in addition to background regional standard of care therapy, including treatments to control co-morbidities, in reducing the composite of CV death and heart failure events (hospitalisations for HF or urgent HF visits). Adult patients aged ≥40 years with HFpEF (LVEF >40% and evidence of structural heart disease) and New York Heart Association (NYHA) class II-IV who are eligible according to the inclusion/exclusion criteria will be randomised in a 1:1 ratio to receive either dapagliflozin 10 mg or placebo. Both out-patients and in-patients hospitalised for heart failure and off intravenous heart failure-therapy for 24 hours can be randomised. It is estimated that approximately 8000 patients at approximately 400-500 sites in 20-25 countries will need to be enrolled to reach the target of approximately 4700 randomised patients.

Study Period:

Estimated date of first patient enrolled: Q3 2018

Estimated date of last patient completed: Q3 2021

Number of randomised Subjects: approximately 4700 patients

Treatments and treatment duration:

Patients will be randomised in a 1:1 ratio to receive either dapagliflozin 10 mg or placebo once daily. The anticipated average treatment duration is 24 months (range 15 to 33 months).

Data Monitoring Committee:

An independent Data Monitoring Committee (DMC) will review accumulating trial data by treatment group in order to monitor patient safety and efficacy, ensure the validity and integrity of the trial, and make benefit-risk assessment.

Statistical methods

This study is event-driven. The primary objective of the study is to determine the superiority of dapagliflozin versus placebo, when added to standard of care, in reducing the composite of CV death and HF events (hospitalisation for HF or urgent HF visit). Assuming a true hazard ratio (HR) of 0.80 between dapagliflozin and placebo, using a two-sided alpha of 5%, 844 primary endpoint events will provide a statistical power of 90% for the test of the primary composite endpoint.

Approximately 4700 patients are estimated to provide the required number of primary events during an anticipated recruitment period of 18 months and a minimal follow-up period of 15 months (total study duration 33 months, average follow-up 24 months). Randomisation will be stratified by presence or absence of Type 2 Diabetes (T2D).

All patients who have been randomised to study treatment will be included in the Full Analysis Set (FAS) irrespective of their protocol adherence and continued participation in the study. The primary variable is the time to first event included in the primary composite endpoint. The primary analysis will be based on the intention to treat (ITT) principle using the FAS, including events occurring on or prior to the primary analysis censoring date (PACD), confirmed by adjudication.

In the analysis of the primary composite endpoint, treatments (dapagliflozin versus placebo) will be compared using a Cox proportional hazards model with a factor for treatment group, stratified by type 2 diabetes (T2D) status at randomisation. The p-value, hazard ratio and 95% confidence interval will be reported.

Interim analysis of superiority and futility is planned to be performed including approximately 67% of target number of adjudicated primary endpoints.

A closed testing procedure including a pre-specified hierarchical ordering of the primary and secondary endpoints will be utilized. No multiplicity control is placed on the exploratory endpoints.

1.3 Schema

The general study design is summarised in Figure 1.

Figure 1 Study design

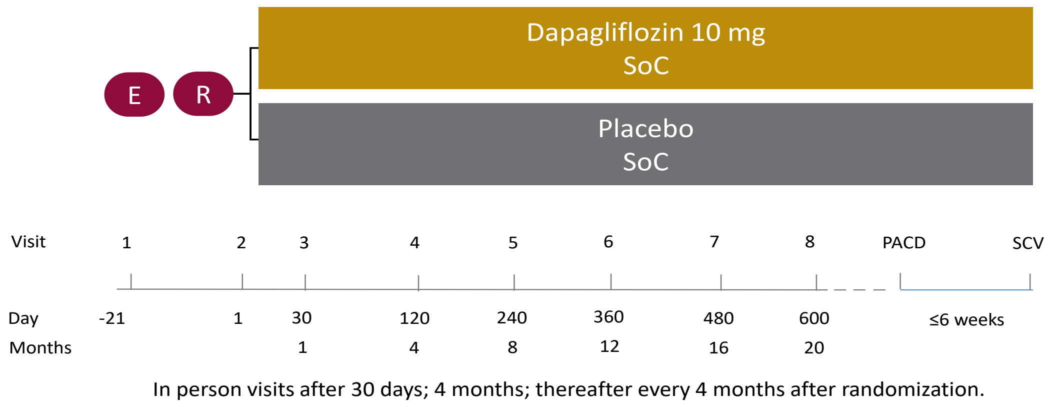

E=Enrolment; R=Randomization; SoC= Standard of Care; PACD=Primary Analysis Censoring Date; SCV=Study Closure Visit; FU=Follow Up

2. INTRODUCTION

2.1 Study rationale

The prevalence of chronic HF continues to increase globally. An estimated 38 million people are affected worldwide (Braunwald 2015), with over 1 million hospitalisations annually in both the United States and Europe (Ambrosy et al 2014). The annual global economic burden in 2012 was estimated to be $108 billion, (Cook et al 2014); this will increase dramatically as the population ages.

Heart failure is a complex syndrome caused by structural and/or functional abnormalities. It is characterised by dyspnoea, fatigue, and pulmonary congestion and/or peripheral oedema due to fluid retention. Patients with signs and symptoms of HF are categorised, based on measurement of left-ventricular ejection fraction (LVEF), as having HF with reduced LVEF (HFrEF) or HF with preserved LVEF (HFpEF).

Approximately half of all heart failure patients have HFpEF (Oktay et al 2013). Risk of death for HFpEF patients is high, with annualised mortality rate up to 15% in community settings (Lam et al 2011). In controlled clinical trials, patients with HFpEF tend to be older and have a higher prevalence of hypertension as compared to patients with HFrEF, although major clinical outcomes are similarly dominated by CV death and HF hospitalisation, the yearly event rates appear to be lower than in HFrEF (Solomon et al 2005). However, patients with HFpEF have a particularly significant unmet medical need given that outcome studies hitherto performed have not resulted in any approved pharmacotherapy specifically for this condition. Conversely, outcome studies have provided evidence for treatments for HFrEF that hence can improve symptoms and haemodynamics as well as reduce hospitalisations for heart failure and mortality. These treatments include diuretics, angiotensin converting enzyme inhibitors, angiotensin II receptor blockers, angiotensin II receptor blocker neprilysin inhibitors, mineralocorticoid receptor antagonists, and beta-blockers (Iwaz et al 2016).

Recent data from cardiovascular (CV) outcome trials of SGLT2 inhibitors (empagliflozin and canagliflozin) indicate that treatment with SGLT2 inhibitors can reduce the risk of CV death and hospitalisation due to HF in patients with T2D overall, and in patients with T2D and concomitant HF (Zinman et al 2015; Fitchett et al 2016; Neal et al 2017; Rådholm et al 2018).

Results from real-world observational studies are broadly consistent with the randomised clinical trials in supporting the benefits of SGLT2 inhibitors in reducing risk of HF hospitalisation and CV death. The CVD-REAL study, consisting of more than 300000 patients with T2D, both with and without established CV disease, across 6 countries found that patients treated with SGLT2 inhibitors compared to patients treated with other glucose lowering drugs was associated with a relative risk reduction in hospitalisation due to HF (39%), all-cause death (51%), and the composite of hospitalisation due to HF or CV death (46%) (Kosiborod et al 2017a).

Limitations associated with the randomised clinical trials as well as the observational studies are that only patients with T2D were studied, and that the proportion of patients with HFrEF and HFpEF, respectively, is unknown.

This study will test the hypothesis that dapagliflozin is superior to placebo in reducing the composite of CV death and HF events (hospitalisation for HF or urgent HF visit) in patients with HF and preserved systolic function (LVEF >40%), with or without T2D.

2.2 Background

Dapagliflozin is a potent, highly selective and orally active inhibitor of human renal SGLT2. A detailed description of the chemistry, pharmacology, efficacy, and safety of dapagliflozin is provided in the Investigator's Brochure. Supporting the hypothesis that dapagliflozin may reduce CV Death and HF events in HF patients, irrespective of diabetes status, are observations 'from the overall dapagliflozin clinical development programme. Dapagliflozin lowers HbA1c with a low risk of inducing hypoglycaemia. In addition, dapagliflozin treatment has also been shown to reduce weight and systolic blood pressure, and to have favourable effect on increased blood uric acid, albuminuria, and arterial elasticity, conditions which are associated with increased CV and renal risk (Shigiyama et al 2017). Dapagliflozin is believed be nephroprotective through non-glycaemic mechanisms (Wanner et al 2016).

The identified blood pressure lowering effects, may reduce the primary outcome in a study population with high prevalence of hypertension, similarly, the observed effects on body weight, may be beneficial to the large part of the study population with obesity. The findings from EMPA-REG, with a similar SGLT2 inhibitor compound, suggests that kidney function is preserved, or improved in this diabetic study population. Furthermore, HFpEF patients are characterized by fluid retention and a change in cardiac metabolism favouring glucose as substrate, both of which has been hypothesised to be positively impacted by SGLT2 inhibitor treatment. Moreover, arterial stiffness, and abnormal ventriculo-arterial coupling, are common in patients with HFpEF, and may be modified by SGLT2 inhibitor treatments.

The clinical studies in healthy subjects at high multiple doses also show that, due to the mechanism of action, dapagliflozin does not induce hypoglycemia in nondiabetic subjects; however, pharmacodynamic effects on glucose, sodium, and urinary volume are observed. Therefore, the changes in these diabetes-independent mechanisms and intrarenal physiology are expected to be similar regardless of underlying disease.

This study is an international, multicentre, parallel-group, event-driven, randomised, double-blind, placebo-controlled study in HFpEF patients, evaluating the effect of dapagliflozin 10 mg, given once daily in addition to background regional standard of care therapy, including treatments to control co-morbidities, in reducing the composite of CV death and heart failure events (hospitalisations for HF or urgent HF visits).

2.3 Benefit/risk assessment

Dapagliflozin has global marketing approval in 44 countries with the most recent estimate of cumulative post-marketing experience totalling over 1.6 million patient-years. Detailed information about the known and expected benefits and risks and reasonably expected adverse events of dapagliflozin appears in the Investigator's Brochure. The following is a summary of benefit-risk considerations relevant to the HFpEF target population.

2.3.1 Potential risks to patients

Dapagliflozin reduces blood volume and blood pressure from its diuretic effect, which could be a concern in patients with HFpEF, but also be important mechanisms of a potential treatment effect. However, in the dapagliflozin type 2 diabetes mellitus (T2D) program, the rate of events related to volume depletion and impaired renal function have been similar between dapagliflozin and placebo. Loop-diuretics are widely used in the target patient population and are also allowed in this study. A pooled analysis of patients with T2D and HF in the dapagliflozin development program, showed no increase of volume depletion events but increase in renal events, mainly creatinine increases, in patients treated with dapagliflozin (n=171) compared with placebo treated patients (n=149). About half of the patients were on loop diuretics (Kosiborod et al 2017b).

An increase in amputations, mostly affecting toes, was observed in a clinical trial (Neal et al 2017) with another SGLT2 inhibitor. There is no indication from the clinical development program that dapagliflozin is associated with an increased risk of amputation (see Section 8.5.1.1 for the detection and capture of amputation events).

Dapagliflozin has not been shown to induce hypoglycaemia in non-diabetes patients. In clinical pharmacology studies, healthy subjects have been treated with single oral doses up to 500 mg and multiple oral doses of 100 mg up to 14 days without any hypoglycaemic events.

There have been post-marketing reports of ketoacidosis, including diabetic ketoacidosis, in patients with T2D taking dapagliflozin and other SGLT2 inhibitors, although a causal relationship has not been established.

Patients treated with dapagliflozin who present with signs and symptoms consistent with ketoacidosis, including nausea, vomiting, abdominal pain, malaise, and shortness of breath, should be assessed for ketoacidosis, even if blood glucose levels are below 14 mmol/L (250 mg/dL). If ketoacidosis is suspected interruption of dapagliflozin treatment should be considered and the patient should be promptly evaluated.

Predisposing factors to ketoacidosis include a low beta-cell function reserve resulting from pancreatic disorders (e.g., T1D, history of pancreatitis, or pancreatic surgery), insulin dose reduction, reduced caloric intake, or increased insulin requirements due to infections, illness or surgery and alcohol abuse. Dapagliflozin should be used with caution in patients in these circumstances. Dapagliflozin is currently not indicated for the treatment of patients with T1D; these patients are excluded from this study.

2.3.1.1 Protection against risks

This study has been designed with appropriate measures in place to monitor and minimise any potential risks to participating patients. Data regarding amputations and adverse events potentially placing the patient at risk for a lower limb amputation will be collected (see Section 8.5.1.1). To ensure the safety of all patients participating in AstraZeneca sponsored studies, reviews of all safety information from all ongoing clinical dapagliflozin studies are conducted as they become available. In addition, an independent Data Monitoring Committee (DMC) will be responsible for safeguarding the interests of the patients by reviewing safety data throughout the study (see Section 9.5.1).

2.3.2 Potential benefits to patients

All patients in the study are expected to be optimally treated according to regional standard of care therapy, including treatments to control co-morbidities, and dapagliflozin or placebo will be administered on top of this treatment.

All patients participating in clinical trials irrespective of whether treated with active treatment or not, generally receive closer medical attention than those in ordinary clinical practice which may be to their advantage.

2.3.3 Conclusion

Considering the non-clinical and clinical experience with dapagliflozin and the precautions included in the study protocol, participation in this study should present a minimal and thus acceptable risk to eligible patients. Although hypothesis-generating data suggest beneficial effects of SGLT2 inhibitors in patients with T2D with heart failure, at the time of writing of this clinical study protocol, no available SGLT2 inhibitor has a treatment indication for patients with HFpEF. The proposed clinical study will test the hypothesis that dapagliflozin reduces the risk of CV death and HF events in patients with HFpEF, with or without T2D, in a rigorous fashion. The results could potentially offer substantial benefit to patients with HFpEF, a patient population with a large medical need for effective treatments.

3. OBJECTIVES AND ENDPOINTS

Table 3 Study objectives

**Primary objective:**	**Endpoint/variable:**
To determine whether dapagliflozin is superior to placebo, when added to standard of care, in reducing the composite of CV death and HF events (hospitalisation for HF or urgent HF visit) in patients with HF and preserved systolic function.	Time to the first occurrence of any of the components of this composite:<br>1. CV death<br>2. Hospitalisation for HF<br>3. Urgent HF visit (e.g., emergency department or outpatient visit)
**Secondary objective:**	**Endpoint/variable:**
To determine whether dapagliflozin is superior to placebo in reducing the total number of recurrent HF hospitalisations and CV death	Total number of (first and recurrent) hospitalisations for HF and CV death
To determine whether dapagliflozin is superior to placebo in improving Patient Reported Outcomes measured by KCCQ	Change from baseline in the total symptom score (TSS) of the KCCQ at 8 months
To determine whether dapagliflozin is superior to placebo in reducing the proportion of patients with worsened NYHA class	Proportion of patients with worsened NYHA class from baseline to 8 months

To determine whether dapagliflozin is superior to placebo in reducing all-cause mortality	Time to the occurrence of death from any cause
**Safety objective:**	**Endpoint/variable:**
To evaluate the safety and tolerability of dapagliflozin compared to placebo in patients with HFpEF	Serious adverse events (SAEs), adverse events leading to treatment discontinuation (DAEs), amputations, adverse events (AEs) leading to amputation and potential risk factor AEs for amputations affecting lower limbs
**Exploratory Objective:**	**Endpoint/Variable:**
To determine whether dapagliflozin is superior to placebo in reducing all-cause hospitalisation	Time to the first occurrence of hospitalisation from any cause
To compare the effect of dapagliflozin versus placebo on health status assessed by EuroQol five-dimensional five-level questionnaire (EQ- 5D-5L) to support health economic analysis and health technology assessment	Changes in health status measured by EQ-5D-5L
To compare the effect of dapagliflozin versus placebo on health status assessed by Patient global impression of severity (PGIS) questionnaires	Changes in health status measured by PGIS
To determine whether dapagliflozin compared with placebo will have an effect on systolic BP	Change in systolic BP from baseline
To determine whether dapagliflozin compared with placebo will have an effect on body weight	Change in body weight from baseline
To determine whether dapagliflozin compared with placebo will have an effect on eGFR	Change in eGFR from baseline
To explore whether dapagliflozin compared to placebo improves KCCQ summary scores, subscores of TSS (Symptom frequency and symptom burden) and domains	Change in Clinical summary score, TSS sub-scores, Overall summary score, QoL score
To collect and store blood samples for future exploratory genetic research	Not applicable. Results will be reported separately

BP Blood pressure; CV Cardiovascular; EQ-5D-5L EuroQol five-dimensional five-level questionnaire
HF Heart failure; HFrEF Heart failure with reduced ejection fraction; KCCQ Kansas City Cardiomyopathy Questionnaire NYHA New York Heart Association

4. STUDY DESIGN

4.1 Overall design

This is an international, multicentre, parallel-group, event-driven, randomised, double-blind, placebo-controlled study in HFpEF patients, evaluating the effect of dapagliflozin 10 mg versus placebo, given once daily in addition to background regional standard of care therapy, including treatments to control co-morbidities, in reducing the composite of CV death or heart failure events.

For an overview of the study design see Figure 1, Section 1.3. For details on treatments given during the study, see Section 6.1.

For details on what is included in the efficacy and safety endpoints, see Section 3 Objectives and Endpoints.

Adult patients with HFpEF (defined for the purposes of this study as LVEF >40% and evidence of structural heart disease) aged ≥40 years and with NYHA class II-IV who meet the inclusion criteria, and none of the exclusion criteria, will be randomised in a 1:1 ratio to receive either dapagliflozin 10 mg or placebo. Randomised treatment should be started as soon as possible and within 24 hours after randomisation. It is estimated that approximately 8000 patients at approximately 400-500 sites in 20-25 countries will be enrolled to reach the target of approximately 4700 randomised patients.

Study closure procedures will be initiated when the predetermined number of primary endpoints are predicted to have occurred (n=844), i.e. the Primary Analysis Censoring Date (PACD). Patients should be scheduled for a Study Closure Visit (SCV) within 6 weeks of the PACD. The anticipated total study duration is approximately 33 months dependent on randomisation rate and event rate. The study duration, and the number of patients, may be changed if the randomisation rate or the event rate is different than anticipated. The study may be terminated early if a clear harmful effect of the study treatment is detected during the DMC review, or due to DMC recommendations following pre-specified interim analyses (see Section 9.5).

Data on baseline characteristics, endpoints and AEs will be collected through a validated electronic data capture (EDC) system with electronic case report forms (eCRFs).

4.2 Scientific rationale for study design

This is a randomised, multi-centre, double-blind, parallel-group study. Randomisation and double-blinding will minimise potential bias. The target population includes adult (aged ≥ 40 years) male and female patients with HFpEF, which is defined in this study as individuals with an established diagnosis of heart failure and a LVEF >40% and structural heart disease who meet natriuretic peptide thresholds. The requirement of demonstrated structural heart disease (i.e. left ventricular

hypertrophy or left atrial enlargement[1]) and elevated natriuretic peptides aims to support the diagnosis of heart failure, since other common co-morbidities may cause overlapping symptoms. Most randomised patients will be out-patients. However, to address a specific need in a period with high risk for events, a proportion of patients will be enrolled and randomised during hospitalisation for heart failure or within 21 days of discharge from hospitalisation for heart failure (subacute subgroup).

The study population will include patients both with and without T2D, as the beneficial haemodynamic effects of dapagliflozin appear to be independent of the glycaemic effect, and can therefore be expected in both groups. Enrolment in the study may be capped based on the proportion of patients with/without T2D, in certain LVEF categories, in each NYHA class, with/without atrial fibrillation, randomised during or early after HF hospitalisation (subacute subgroup), and geographic region.

The control group will receive placebo; there are no approved pharmacological treatments for HFpEF that could be utilised as a comparator. All patients will be treated according to local guidelines on standard of care treatment for patients with HFpEF, focusing on treatment of HF symptoms (e.g. diuretics) and comorbidities (including treatment for high blood pressure, ischaemic heart disease, atrial fibrillation).

The study population will include patients with eGFR $\geq$ 25 ml/min/1.73m^2. Patients with reduced renal function have a clinical picture with increased intra-glomerular pressure, hypertension, proteinuria and fluid/sodium overload and SGLT2 inhibition can improve all these abnormalities through metabolic-independent mechanisms. Thus, patients with heart failure and reduced renal function could be expected to benefit from treatment with dapagliflozin.

The primary efficacy endpoints of the study are adjudicated CV death and HF events (hospitalisation for HF or urgent HF visit). The rationale for selecting CV death over all-cause death is the expectation that HF treatment will decrease CV death and not all potential causes of death (Zannad et al 2014). Heart failure events include both HF hospitalisations and unplanned HF visits requiring urgent treatment independently of whether the exacerbation of HF results in hospitalisation (according to CDISC definitions; Hicks et al 2014; Hicks et al. 2018 Hicks KA, Mahaffey KW, Mehran R, Nissen SE, et alCardiovascular and Stroke Endpoint Definitions for Clinical Trials. J Am Coll Cardiol 2018;71:1021–34 These are the same endpoint

[1] Left Atrial Enlargement defined by at least 1 of the following: LA width (diameter) $\geq$3.8 cm or LA length $\geq$5.0 cm or LA area $\geq$20 cm^2 or LA volume $\geq$55 mL or LA volume index $\geq$29 mL/m^2. Left Ventricular Hypertrophy defined by septal thickness or posterior wall thickness $\geq$1.1 cm

definitions currently employed in the Sponsor's ongoing HFrEF outcome study (Dapa-HF; Study D1699C00001).

The rationale for including outpatient HF events, in addition to hospital admissions, is that it is the occurrence of worsening of the patient's condition necessitating treatment, and not the place of treatment, that is important. As stated in EMA Guidance 2016, '…patient are often managed for episodes of transient decompensation or worsening HF in outpatient settings (eg, emergency departments, observation units, other outpatient settings). The capture of events of worsening HF without hospitalisation may be warranted as an additional endpoint.' Including only hospital admissions is likely to overlook a modest but significant proportion of episodes of worsening HF (Skali et al 2014, Okumura et al 2016, Greene at al 2000).

While CV death and HF hospitalisations are clearly important to patients and health-care systems, the impact of HF on patients' symptoms and physical/social functioning is also important. In order to evaluate the treatment effects on these aspects of the impact of HF, we will use the Kansas City Cardiomyopathy Questionnaire (KCCQ), a disease-specific patient reported outcomes (PRO) measure developed for patients with chronic HF. The KCCQ has shown to be a valid, reliable and responsive measure for patients with HF (Greene at al 2000, Spertus et al 2005).

4.3 Justification for dose

The 10 mg dose of dapagliflozin has a well-characterised efficacy and safety profile in the T2D clinical development program and is the recommended dose in the majority of countries worldwide.

From a pharmacokinetic perspective, the currently approved dapagliflozin dose of 10 mg once daily is appropriate for use in patients with HFpEF. Slightly higher systemic exposure to dapagliflozin is expected in HFpEF patients when symptomatic, based on the dual renal and hepatic metabolism of dapagliflozin and the lower perfusion of these organs in this patient group. However, the increase in systemic exposure of 10 mg dapagliflozin is not anticipated to warrant dose adjustment in HF patients. Moreover, the anticipated slightly higher systemic exposure to dapagliflozin is likely to be beneficial in HF patients, by compensating for the reduced renal perfusion and consequently lower renal glucose and sodium filtered loads in these patients. Doses lower than 10 mg are therefore unlikely to provide as much benefit to patients with HF as the 10-mg dose. Lastly, no changes in dose of concomitant medications in the HFpEF population are needed due to a lack of clinically meaningful drug-drug interactions for dapagliflozin with current medications used for treatment of patients with HFpEF, including standard of care medications used to control co-morbidities in this patient group.

In the dapagliflozin clinical program, there are no dose-related SAEs that preclude the use of 10 mg as a preferred dose. Additionally, in a post-hoc analysis of data from 320 patients with a documented history of HF and concomitant T2D in placebo-controlled clinical trials, dapagliflozin 10 mg was found to be well tolerated in this population (Kosiborod et al 2017b).

There are mechanistic reasons for choosing the 10-mg dose as well. One hypothesis of underlying pathophysiology in HFpEF is abnormal pressure coupling between the left ventricle and aorta, and drugs that reduce aortic stiffness may have beneficial effects in patients with HFpEF (Borlaug and

Paulus 2011). Studies examining the highest approved dose for empagliflozin have reported improvements in aortic elasticity (Chilton et al 2015,Cherney et al 2014); similar studies are ongoing with dapagliflozin. In a completed placebo-controlled study, treatment with dapagliflozin 10 mg resulted in improvements in parameters associated with arterial remodelling in addition to lowering blood pressure in patients with T2D (Ott et al 2017). This prior work suggests that selecting the 10-mg dose of dapagliflozin is reasonable from a mechanistic perspective to demonstrate a clinical effect.

4.4 End of study definition

The end of study is defined as the last expected visit/contact of the last subject undergoing the study.

The study may be terminated at individual study sites if the study procedures are not being performed according to GCP, or if no patients are recruited. Patients from terminated sites will have the opportunity to be transferred to another site to continue the study. AstraZeneca may also terminate the entire study prematurely if concerns for safety arise within this study or in any other study with dapagliflozin, or due to recommendation by the DMC. Regardless of the reason for termination, all data required by the protocol at the time of discontinuation of follow-up will be collected. In terminating the study, the Sponsor will ensure that adequate consideration is given to the protection of the patients' interests.

See Appendix A 6 for guidelines for the dissemination of study results.

5. STUDY POPULATION

Prospective approval of protocol deviations to recruitment and enrolment criteria, also known as protocol waivers or exemptions, is not permitted.

In this protocol, 'enrolled' patients are defined as those who sign the informed consent form (ICF) and received E-Code. The ICF process is described in Appendix A 3. 'Randomised' patients are defined as those who undergo randomisation and receive a randomisation code.

Patients are eligible to be randomised in the study only if all of the following inclusion criteria and none of the exclusion criteria apply. Enrolled patients who for any reason are not randomised are considered screen failures (see Section 5.4).

5.1 Inclusion criteria

Subjects are eligible to be randomised in the study only if all of the following inclusion criteria and none of the exclusion criteria apply:

1. Provision of signed informed consent prior to any study specific procedures.

2. Male or female patients age ≥40 years.

3. Documented diagnosis of symptomatic heart failure (NYHA class II-IV) at enrolment, and a medical history of typical symptoms/signs[2] of heart failure ≥6 weeks before enrolment with at least intermittent need for diuretic treatment.

4. Left Ventricular Ejection Fraction (LVEF) >40% and evidence of structural heart disease (i.e. left ventricular hypertrophy or left atrial enlargement[3]) documented by the most recent echocardiogram, and/or cardiac MR within the last 12 months prior to enrolment. For patients with prior acute cardiac events or procedures that may reduce LVEF, e.g. as defined in exclusion criterion 6, qualifying cardiac imaging assessment at least 12 weeks following the procedure/event is required.

5. NT-pro BNP ≥300 pg/ml at Visit 1 for patients without ongoing atrial fibrillation/flutter. If ongoing atrial fibrillation/flutter at Visit 1, NT-pro BNP must be ≥600 pg/mL.

6. Patients may be ambulatory, or hospitalized; patients must be off intravenous heart failure therapy (including diuretics) for at least 12 hours prior to enrolment and 24 hours prior to randomisation.

5.2 Exclusion criteria

1. Receiving therapy with an SGLT2 inhibitor within 4 weeks prior to randomisation or previous intolerance to an SGLT2 inhibitor

2. Type 1 diabetes mellitus (T1D)

3. eGFR <25 mL/min/1.73 m^2 (CKD-EPI formula) at Visit 1

[2] Typical symptoms associated with heart failure: breathlessness, orthopnoea, paroxysmal nocturnal dyspnoea, reduced exercise tolerance, fatigue, tiredness, increased time to recover after exercise, ankle swelling;
Signs associated with Heart Failure:
More specific: elevated jugular venous pressure, hepatojugular reflex, third heart sound (gallop rhythm), laterally displaced apical impulse
Less specific: weight gain (>2 kg/week), weight loss (in advanced HF), tissue wasting (cachexia), cardiac murmur, peripheral oedema (ankle, sacral, scrotal), pulmonary crepitations, reduced air entry and dullness to percussion at lung bases (pleural effusion), tachycardia, irregular pulse, tachypnoea, cheyne stokes respiration, hepatomegaly, ascites, cold extremities, oliguria, narrow pulse pressure

[3] Left Atrial Enlargement defined by at least 1 of the following: LA width (diameter) ≥3.8 cm or LA length ≥5.0 cm or LA area ≥20 cm^2 or LA volume ≥55 mL or LA volume index ≥29 mL/m^2. Left Ventricular Hypertrophy defined by septal thickness or posterior wall thickness ≥1.1 cm

4. Systolic blood pressure (BP) <95 mmHg on 2 consecutive measurements at 5-minute intervals, at Visit 1 or at Visit 2

5. Systolic BP≥160 mmHg if not on treatment with ≥3 blood pressure lowering medications or ≥180 mmHg irrespective of treatments, on 2 consecutive measurements at 5-minute intervals, at Visit 1 or at Visit 2.

6. MI, unstable angina, coronary revascularization (percutaneous coronary intervention (PCI) or coronary artery bypass grafting (CABG)), ablation of atrial flutter/fibrillation, valve repair/replacement within 12 weeks prior to enrolment. Before enrolment, these patients must have their qualifying echocardiography and/or cardiac MRI examination at least 12 weeks after the event.

7. Planned coronary revascularization, ablation of atrial flutter/fibrillation and valve repair/replacement.

8. Stroke or transient ischemic attack (TIA) within 12 weeks prior to enrolment

9. Probable alternative or concomitant diagnoses which in the opinion of the investigator could account for the patient's HF symptoms and signs (e.g. anaemia, hypothyroidism)

10. Body mass index >50 kg/m^2

11. Primary pulmonary hypertension, chronic pulmonary embolism, severe pulmonary disease including COPD (i.e., requiring home oxygen, chronic nebulizer therapy or chronic oral steroid therapy, or hospitalisation for exacerbation of COPD requiring ventilatory assist within 12 months prior to enrolment)

12. Previous cardiac transplantation, or complex congenital heart disease. Planned cardiac resynchronisation therapy.

13. HF due to any of the following: known infiltrative cardiomyopathy (e.g. amyloid, sarcoid, lymphoma, endomyocardial fibrosis), active myocarditis, constrictive pericarditis, cardiac tamponade, known genetic hypertrophic cardiomyopathy or obstructive hypertrophic cardiomyopathy, arrhythmogenic right ventricular cardiomyopathy/dysplasia (ARVC/D), or uncorrected primary valvular disease

14. A life expectancy of less than 2 years due to any non-cardiovascular condition, based on investigator's clinical judgement.

15. Inability of the patient, in the opinion of the investigator, to understand and/or comply with study medications, procedures and/or follow-up OR any conditions that, in the opinion of the investigator, may render the patient unable to complete the study

16. Active malignancy requiring treatment (with the exception of basal cell or squamous cell carcinomas of the skin).

17. Acute or chronic liver disease with severe impairment of liver function (e.g., ascites, oesophageal varices, coagulopathy)

18. Women of child-bearing potential (i.e. those who are not chemically or surgically sterilised or post-menopausal) not willing to use a medically accepted method of contraception considered reliable in the judgment of the investigator OR who have a positive pregnancy test at randomisation OR who are breast-feeding

19. Involvement in the planning and/or conduct of the study (applies to both AstraZeneca personnel and/or personnel at the study site)

20. Previous randomisation in the present study

21. Participation in another clinical study with an IP or device during the last month prior to enrolment

5.3 Lifestyle restrictions (not applicable)

5.4 Screen failures

Enrolled patients who are found not eligible (i.e. not meeting all the inclusion criteria or fulfilling any of the exclusion criteria) must not be randomised or initiated on treatment.

Screen failures are defined as patients who signed the informed consent form to participate in the study but are not subsequently randomised. A minimal set of screen failure information is required to ensure transparent reporting of screen failure patients to meet the Consolidated Standards of Reporting Trials (CONSORT) publishing requirements and to respond to queries from regulatory authorities. Minimal information includes demography, eligibility criteria (reason for screen failure), and any serious adverse event (SAE).

Screen failures may be re-enrolled one time during the study if the Investigator considers that the patient may be eligible for participation in this study at another time point. Re-enrolled patients should be assigned the same enrolment code as for the initial enrolment. All enrolment assessments and procedures, including signing the informed consent form, should be performed again.

5.5 Procedures for handling of randomized not eligible patients

If a patient is randomised and later found not eligible, the Investigator should immediately inform the AstraZeneca representative, who will report the protocol deviation to the AstraZeneca Study Physician.

Study treatment must be discontinued in all cases where continued treatment is deemed to pose a safety risk to the patient. Regardless of whether study treatment is discontinued or not, the patient should continue his/her participation in the study for follow-up of endpoints and other protocol-defined study procedures until the end of the study. Consistent with the intention-to-treat principle, all randomised patients are included in the efficacy analysis according to randomised treatment assignment. The AstraZeneca Study Physician must ensure that the protocol deviation and the rationale for the decision to discontinue or continue study treatment are appropriately documented.

6. STUDY TREATMENTS

Study treatment is defined as any investigational product(s) (including marketed product comparator and placebo) or medical device(s) intended to be administered to a study participant according to the study protocol. Study treatment in this study refers to dapagliflozin or matching placebo.

6.1 Treatments administered

Table 4 Study Treatments

	Dapagliflozin	Placebo
**Investigational Product name**	Dapagliflozin 10 mg	Matching placebo 10 mg
**Dosage formulation**	Green, diamond shaped, film coated tablets 10 mg	Green, diamond shaped, film coated tablets placebo
**Route of administration**	Oral	Oral
**Dosing instructions**	Once daily	Once daily
**Packaging and labelling**	Investigational Product will be provided in bottles. Each bottle will be labelled in accordance with Good Manufacturing Practice Annex 13 and per country regulatory requirements	Investigational Product will be provided in bottles. Each bottle will be labelled in accordance with Good Manufacturing Practice Annex 13 and per country regulatory requirements
**Provider**	AstraZeneca	AstraZeneca

The tablets contain lactose, in quantities not likely to cause discomfort in lactose-intolerant individuals.

6.2 Preparation/handling/storage/accountability

The investigator or designee must confirm appropriate temperature conditions have been maintained during transit for all study treatment received and any discrepancies are reported and resolved before use of the study treatment.

All investigational product (IP) should be kept in a secure place under appropriate storage conditions. The label on the IP bottle specifies the appropriate storage.

Only patients randomised in the study may receive IP and only authorised site staff may supply or administer IP. The administration of all investigational products should be recorded in the appropriate sections of the eCRF.

The Investigator is responsible for IP accountability, reconciliation, and record maintenance (i.e. receipt, reconciliation, and final disposition records).

The investigator will retain the returned IP until the AZ representative or delegate collects it, along with any IP not dispensed. The AZ representative or delegate is responsible for confirming the investigator or delegate has recorded the quantities of returned and unused tablets at a patient level before IP is destroyed. The AZ representative or delegate will advise on the appropriate method for destruction of unused IP.

6.3 Measures to minimise bias: randomisation and blinding

All patients will be centrally assigned to randomised IP using an interactive voice/web response system (IxRS). Randomisation to IP will be performed in balanced blocks to ensure approximate balance between the treatment groups (1:1). The randomisation codes will be computer generated and loaded into the IxRS database. Before the study is initiated, the telephone number and call-in directions for the IxRS and/or the log-in information and directions for the IxRS will be provided to each site.

If a randomised patient withdraws from the study, then his/her enrolment/randomisation code cannot be reused. Withdrawn randomised patients will be included in the intention to treat analysis.

The IxRS will provide the Investigator with the kit identification number to be allocated to the patient at each dispensing visit. At all visits where IP is dispensed, site personnel will do a kit verification in IxRS before providing the IP bottle to the patient. Routines for this will be described in the IxRS user manual that will be provided to each centre.

The blinding of treatment is ensured by using a double-blind technique. Individual treatment codes, indicating the randomised treatment for each patient, will be available to the investigator(s) or pharmacists from the IxRS. Instructions for code breaking/unblinding will be described in the IxRS user manual that will be provided to each site.

The randomisation code should not be broken except in medical emergencies when the appropriate management of the patient requires knowledge of the treatment randomisation. The Investigator is to document and report the action to AstraZeneca, without revealing the treatment given to the patient to the AstraZeneca staff.

AstraZeneca retains the right to break the code for SAEs that are unexpected and are suspected to be causally related to an investigational product and that potentially require expedited reporting to regulatory authorities. Randomisation codes will not be broken for the planned analyses of data until all decisions on the evaluability of the data from each individual patient have been made and documented.

6.3.1 Stratification and capping

The recruitment will be continuously monitored in order to achieve adequate proportions of patient sub-populations.

6.3.1.1 Stratification

Randomisation will be stratified in IxRS based on patients with and without T2D at the time of randomisation in order to ensure approximate balance between treatment groups within each sub-population. Stratification on T2D at the time of randomisation is based on:

- Established diagnosis of T2D

OR

- HbA1c ≥6.5% (48 mmol/mol) shown at central laboratory test at enrolment (Visit 1)

6.3.1.2 Capping

The intent is to enrol a typical cross-section of patients with HFpEF and to include representative proportions of diabetic and non-diabetic patients. The number of randomised patients with and without T2D will be monitored in order to ensure a minimum of 30% in each sub-population. Randomisation may be capped (i.e., no more patients can be randomised in a specific sub-population) if the pre-determined limit is reached.

Randomisation of patients based on geographic region will be monitored to ensure global representation. LVEF value, NYHA class, subacute subgroup (i.e. randomised in-hospital or within 21 days from discharge) and atrial fibrillation status at Visit 1 may be capped in IxRS to avoid over- or under-representation of these patient subgroups.

6.4 Treatment compliance

The administration of all IP should be recorded in the appropriate sections of the eCRF. Any change from the dosing schedule should be recorded in the eCRF.

Patients will be asked to return all unused IP and empty packages to the clinic at the site visit except Visit 3. At each visit, any patient found to be non-compliant will be counselled on the importance of taking their IP as prescribed. The investigator or delegate will enter the number of returned tablets in the eCRF.

The Investigational Product Storage Manager is responsible for managing IP from receipt by the study site until the destruction or return of all unused IP. The Investigator(s) is responsible for ensuring that the patient has returned all unused IP.

6.5 Concomitant therapy

All patients should be treated according to regional standard of care of HFpEF and existing comorbidities (including treatment of hypertension, ischemic heart disease, atrial fibrillation, diabetes, hyperlipidaemia). Background medications should be part of clinical practice and will not be provided by the Sponsor.

6.5.1 Prohibited medication

Concomitant treatment (i.e., treatment in combination with IP) with open label SGLT2 inhibitors e.g., dapagliflozin, empagliflozin, canagliflozin, ertugliflozin, tofogliflozin and luseogliflozin and

fix dose combinations containing these drugs should not be used. Also in situations when the patient is not on IP, treatment with open label SGLT2 inhibitors during the study could interfere with the interpretation of study results. If treatment with an SGLT2 inhibitor alone or in combination is deemed essential, IP must be discontinued before that treatment is started.

6.5.2 Recording of concomitant treatment

Recording of relevant concomitant medications in eCRF will be made according to the schedule of activities (Table 1). These include medications for cardiovascular conditions as well as diabetes mellitus.

6.5.3 Heart failure background standard of care

The patients should be on background standard of care therapies for patients with HFpEF according to local guidelines, including diuretics when needed to control symptoms and volume overload and adequate treatment of co-morbidities such as hypertension and ischaemic heart disease.

6.5.4 Anti-diabetes treatment

6.5.4.1 Background

More than 40% of patients with established HF are estimated to have T2D (Kristensen et al 2016). Therefore, it is expected that a large proportion of patients will have an established T2D diagnosis when included in this study and that some patients will develop T2D during the course of the study. Treatment of diabetes should follow established guidelines, such as according to glycaemic goals as recommended by the American Diabetes Association (ADA) and European Association for the Study of Diabetes (EASD) in their joint Position Statement (Inzucchi et al 2012, Inzucchi et al 2015).

6.5.4.2 Treatment of patients with established diagnosis of type 2 diabetes

Diabetes medications at baseline and during the study will be recorded in the eCRF. Patients with T2D at randomisation will continue their T2D treatment. SGLT2-inhibitors should be avoided (see Section 6.5.1). Patients treated with insulin or insulin secretagogues have a higher risk of experiencing hypoglycaemic events compared with those treated with other antidiabetic agents. If needed, T2D treatments may be adjusted at the discretion of the Investigator or diabetes health care provider.

6.5.5 Other concomitant treatment

Medications other than described above, which is considered necessary for the patient's safety and wellbeing, may be given at the discretion of the Investigator

6.6 Dose modification (not applicable)

6.7 Treatment after the end of the study

The patients will stop taking IP at the study closure visit (SCV). Remaining IP will be collected at that time. Post-study treatment will not be provided by the Sponsor. Patients should receive standard of care therapy after the SCV, at the discretion of the Investigator.

7. DISCONTINUATION OF TREATMENT AND SUBJECT WITHDRAWAL

7.1 Discontinuation of study treatment

Discontinuation from study treatment is NOT the same thing as a withdrawal from the study. If the patient temporarily or permanently discontinues IP, the patient should remain in the study and it is important that the scheduled study visits and data collection continue according to the study protocol until study closure.

- Patients may be discontinued from IP in the following situations:
- Contraindication to further dosing with IP, in the opinion of the Investigator, such as Adverse event or other safety reasons.
- Severe non-compliance with the study protocol.
- Diabetic ketoacidosis (DKA). Consider temporarily interrupting IP if DKA is suspected. If DKA is confirmed, IP should be discontinued permanently.
- Positive pregnancy test (discontinue IP and notify Sponsor representative).
- Patient decision. The patient is at any time free to discontinue treatment, without prejudice to further treatment.

See the Table 1 for data to be collected at the time of treatment discontinuation and follow-up and for any further evaluations that need to be completed.

7.1.1 Temporary discontinuation

Every attempt should be made to maintain patients on IP during the course of the study. If IP has been interrupted, it should be re-introduced as soon as, in the opinion of the Investigator, the patient's condition is stable.

7.1.1.1 Unexpected acute declines in eGFR

If an unexpected, acute decline in kidney function is observed, the patient should be evaluated and temporary interruption of IP should be considered. Volume depletion, hypotension, inter-current medical problems and concomitant drugs may cause increases in blood creatinine. Urinary tract infection and urinary obstruction should be considered (the latter especially in men). Several drugs may cause a decline in kidney function, especially non-steroidal anti-inflammatory drugs (NSAID) and certain antibiotics such as trimethoprim. If any drug is suspected of causing or contributing to worsening kidney function, their use should be re-considered.

7.1.1.2 Volume depletion/hypotension

Patients with clinically relevant symptoms/signs of suspected volume depletion and/or hypotension, should in addition to considering temporary interruption of IP have their regular medication reviewed, and consideration given to reducing the dose of, or stopping concomitant

medications, as assessed on an individual basis, including diuretics and drugs that lower blood pressure. The need for conventional diuretics (or the dose of diuretic used) should be re-evaluated in light of the patient's symptoms and signs.

7.1.1.3 Patients at risk of volume depletion

Temporary interruption of IP may be considered in patients thought to be at risk of volume depletion/hypotension, such as patients with an acute medical illness potentially causing volume depletion because of inadequate fluid intake or fluid/blood loss (e.g. gastroenteritis, gastrointestinal haemorrhage), or those undergoing major surgery.

7.1.2 Procedures for discontinuation of study treatment

Investigators should instruct their patients to contact the site before or at the time IP is stopped. A patient that decides to discontinue IP will always be asked about the reason(s) and the presence of any AEs. Generally, AEs, SAEs, and potential endpoint events should not lead to IP discontinuation, unless there is a clear clinical rationale to do so.

The date of last intake of IP should be documented in the eCRF. All IP should be returned by the patient at their next on-site study visit or unscheduled visit. Patients permanently discontinuing IP should be given locally available standard of care therapy, at the discretion of the Investigator.

Discontinuation of IP, for any reason, does not impact on the patient´s participation in the study. The patient should continue attending subsequent study visits and data collection should continue according to the study protocol. If the patient does not agree to continue in-person study visits, a modified follow-up must be arranged to ensure the collection of endpoints and safety information. This could be a telephone contact with the patient, a contact with a relative or treating physician, or information from medical records. The approach taken should be recorded in the medical records. A patient that agrees to modified follow-up is not considered to have withdrawn from the study.

Restart of randomised IP is always encouraged. Even if a premature treatment discontinuation visit (PTDV) was completed due to discontinuation of IP, this should not prevent the patient to return to randomised IP if deemed appropriate.

7.2 Lost to follow-up

A patient will be considered potentially lost to follow-up if he or she fails to return for scheduled visits and it is not possible for the site to get contact with the patient. To optimise the chance of getting in contact with the patient during the study, Investigators should record as much contact information as possible at the start of the study including home phone, mobile phone, holiday home phone, family member phone numbers, email address, and social media contact details.

The following actions must be taken if a patient fails to return to the clinic for a required study visit:

- The site must attempt to contact the patient and reschedule the missed visit as soon as possible and counsel the patient on the importance of maintaining the assigned visit schedule.

- Before a patient is deemed potentially lost to follow up, the Investigator or designee must make every effort to regain contact with the patient or next of kin by, e.g. repeat telephone calls, certified letter to the patient´s last known mailing address or local equivalent methods. These contact attempts should be documented in the patient's medical record.

- Efforts to reach the patient should continue until the end of the study. Information regarding vital status should always be collected if possible.

7.3 Withdrawal from the study

Patients are free to withdraw from the study at any time (IP and assessments), without prejudice to further treatment. Withdrawal of consent should only occur if the patient has received appropriate information about and does not agree to any kind of further assessments or contact, including modified follow up options (see Section 7.1.2). Discontinuation of IP in itself is not considered withdrawal of consent.

Withdrawal of consent must be ascertained and documented in writing by the Investigator who must inform the AZ representative and document the withdrawal of consent in the eCRF and medical records.

A patient who withdraws from the study will always be asked about the reason(s) and the presence of any AE. The Investigator will follow up AEs reported outside of the clinical study.

If a patient withdraws from participation in the study, then his/her enrolment and randomisation codes cannot be reused. Withdrawn patients will not be replaced. If the patient withdraws consent for disclosure of future information, the sponsor may retain and continue to use any data collected before such a withdrawal of consent.

To ensure validity of study data, it is very important to collect as much data as possible throughout the study and especially vital status (dead or alive) at study closure (also for patients who have withdrawn their informed consent). The Investigator will therefore attempt to collect information on all patients' vital status from publicly available sources at study closure, even if informed consent has been withdrawn, in compliance with local privacy laws/practices.

8. STUDY ASSESSMENTS AND PROCEDURES

Study procedures and their timing are summarised in the SoA (see Section 1.1).

An Electronic Data Capture (EDC) system will be used for data collection and query handling. The Investigator will ensure that data are recorded in the eCRFs as specified in the study protocol and in accordance with the eCRF instructions provided.

The Investigator ensures the accuracy, completeness, legibility, and timeliness of the data recorded and of the provision of answers to data queries according to the Clinical Study Agreement. The Investigator will sign the completed eCRFs. A copy of the completed eCRFs will be archived at the study site.

All screening evaluations must be completed and reviewed to confirm that potential patients meet all eligibility criteria. The Investigator will maintain a screening log to record details of all patients screened and to confirm eligibility or record reasons for screening failure, as applicable.

Procedures conducted as part of the patient's routine clinical management (e.g. LVEF assessment) and obtained before signing of the ICF may be utilised for screening or baseline purposes provided the procedures met the protocol-specified criteria.

Adherence to the study design requirements, including those specified in the SoA, is essential and required for study conduct.

8.1 Enrolment Period

8.1.1 Visit 1, Enrolment (Day -21 to Day -1)

Enrolment of hospitalized patients is allowed.

At enrolment the following assessments and procedures will be completed:

- The patient signs the ICF
  - Patients who agree to the optional sampling of blood for genetic research will provide their consent
- The investigator reviews the inclusion and exclusion criteria
- The patient will be enrolled and assigned an E-code in IxRS assuming inclusion/exclusion criteria are met
- Demography and relevant medical history (including prior cardiac imaging assessments) will be recorded
- A physical examination will be conducted
- NYHA Functional Classification will be evaluated and recorded
- 12-lead ECG will be recorded
- Vital signs (BP, pulse), height and weight will be assessed and recorded
- Blood samples will be taken for NT-proBNP, creatinine (for calculation of eGFR) and HbA1c assessment (central laboratory)

8.2 Treatment period

8.2.1 Visit 2, Randomisation (Day 1)

Prior to Visit 2, the investigator will assess eligibility based on the central laboratory assessments from Visit 1. Patients not eligible will be considered screen failures and should not continue to Visit 2.

Randomisation of hospitalized patients is allowed.

At randomisation, the following assessments and procedures will be completed:

- Medical history (including cardiac imaging assessments) will be re-assessed
- A physical examination will be conducted
- A pregnancy test for women of child-bearing potential will be done locally with a dipstick provided by central laboratory with result recorded in the medical record
- Vital signs (BP, pulse) will be assessed and recorded
- NYHA Functional Classification will be evaluated and recorded
- The investigator will re-assess the inclusion and exclusion criteria
- KCCQ, PGIS and EQ-5D-5L questionnaires will be completed
- Review of concomitant medication and recording of relevant medications
- If the patient has experienced any SAEs since last visit, this will be recorded in the eCRF
- Randomisation 1:1 ratio to IP (either dapagliflozin at 10 mg or placebo) will be done in IxRS
- IP will be dispensed via IxRS to the patient. The patient will be instructed to take the IP in accordance with protocol without interruptions, and to bring all dispensed bottles to all study visits
- Patients who have consented to sampling for genetic research, will provide a blood sample

8.2.2 Visit 3 (Day 30; ±7 days):

At Visit 3, the following assessments and procedures will be conducted:

- KCCQ and PGIS questionnaires will be completed
- NYHA Functional Classification will be evaluated and recorded

- Vital signs (BP, pulse) will be assessed and recorded
- Review and updating of concomitant medication and recording of relevant medications
- Review and recording of any cardiac and HF related procedures
- Review of potential efficacy and safety events.
- If the patient has experienced any potential endpoints, SAEs, DAEs and/or amputations, adverse events (AEs) leading to amputation and potential risk factor AEs for amputations affecting lower limbs since the last visit, this will be recorded in the eCRF
- Blood samples will be taken for creatinine (for calculation of eGFR) assessment (central laboratory)

8.2.3 Visit 4 (Day 120 ±7 days):

At Visit 4, the following assessments and procedures will be conducted:

- KCCQ and PGIS questionnaires will be completed
- NYHA Functional Classification will be evaluated and recorded
- Review and updating of concomitant medication and recording of relevant medications
- Review and recording of any cardiac and HF related procedures
- Review of potential efficacy and safety events.
- If the patient has experienced any potential endpoints, SAEs, DAEs and/or amputations, adverse events (AEs) leading to amputation and potential risk factor AEs for amputations affecting lower limbs since the last visit, this will be recorded in the eCRF.
- Blood samples will be taken for creatinine (for calculation of eGFR) assessment (central laboratory)
- IP will be dispensed via IxRS to the patient. Drug accountability of the returned IP will be checked. The patient will be instructed to take the IP in accordance with protocol and without interruptions

8.2.4 Visit 5 (Day 240 ±7 days)

At Visit 5, the following assessments and procedures will be conducted:

- KCCQ, PGIS and EQ-5D-5L questionnaires will be completed
- NYHA Functional Classification will be evaluated and recorded
- Review and updating of concomitant medication and recording of relevant medications

- Review and recording of any cardiac and HF related procedures
- Review of potential efficacy and safety events
- If the patient has experienced any potential endpoints, SAEs, DAEs and/or amputations, adverse events (AEs) leading to amputation and potential risk factor AEs for amputations affecting lower limbs since the last visit, this will be recorded in the eCRF.
- IP will be dispensed via IxRS to the patient. Drug accountability of the returned IP will be checked. The patient will be instructed to take the IP in accordance with protocol and without interruptions.

8.2.5 Visit 6 (Day 360 ±7 days)

At Visit 6, the following assessments and procedures will be conducted:

- Vital signs (BP, pulse), and weight will be assessed and recorded
- Review and updating of concomitant medication and recording of relevant medications
- Review and recording of any cardiac and HF related procedures
- Review of potential efficacy and safety events.
- If the patient has experienced any potential endpoints, SAEs, DAEs and/or amputations, adverse events (AEs) leading to amputation and potential risk factor AEs for amputations affecting lower limbs since the last visit, this will be recorded in the eCRF.
- IP will be dispensed via IxRS to the patient. Drug accountability of the returned IP will be checked. The patient will be instructed to take the IP in accordance with protocol and without interruptions.
- Blood samples will be taken for creatinine (for calculation of eGFR) assessment (central laboratory)

8.2.6 Visit 7 and onwards (Day 480 and every 120 days ±14 days)

At visit 7 and subsequent visits, the following assessments and procedures will be conducted:

- Vital signs (BP, pulse), and weight will be assessed and recorded every 12 months
- Review and updating of concomitant medication and recording of relevant medications
- Review and recording of any cardiac and HF related procedures
- Review of potential efficacy and safety events.

- If the patient has experienced any potential endpoints, SAEs, DAEs and/or amputations, adverse events (AEs) leading to amputation and potential risk factor AEs for amputations affecting lower limbs since the last visit, this will be recorded in the eCRF.

- IP will be dispensed via IxRS to the patient. Drug accountability of the returned IP will be checked. The patient will be instructed to take the IP in accordance with protocol and without interruptions.

- Blood samples will be taken for creatinine (for calculation of eGFR) assessment (central laboratory) every 12 months

8.2.7 Premature Treatment Discontinuation Visit

Patients who prematurely and permanently discontinue treatment with IP should return for a premature treatment discontinuation visit (PTDV), which will be done as soon as possible after last dose of IP. The following assessments and procedures will be conducted:

- KCCQ, PGIS and EQ-5D-5L questionnaire will be completed

- NYHA Functional Classification will be evaluated

- Vital signs (BP, pulse) and weight will be assessed and recorded

- Review and updating of concomitant medication and recording of relevant medications

- Review and recording of any cardiac and HF related procedures

- Review of potential efficacy and safety events

- If the patient has experienced any potential endpoints, SAEs, DAEs and/or amputations, adverse events (AEs) leading to amputation and potential risk factor AEs for amputations affecting lower limbs since the last visit, this will be recorded in the eCRF

- Drug accountability of the returned IP will be checked

Patients who discontinue treatment prematurely should attend all study visits according to plan, including the study closure visit (SCV). Patients may re-start treatments if assessed as appropriate by the Investigator. For further details regarding discontinuations from IP, please see Section 7.1.

8.2.8 Study Closure Visit

A primary analysis censoring date (PACD) will be declared based on the rate of accrued endpoints. A study closure visit (SCV) will be scheduled within 6 weeks of the PACD. All patients (including any patients who have discontinued treatment with IP) should return for this visit.

The patient will stop taking IP at the SCV. Remaining IP will be collected at that time and drug accountability will be checked. The following assessments and procedures will be conducted:

- KCCQ, PGIS and EQ-5D-5L questionnaire will be completed
- NYHA Functional Classification will be evaluated
- A physical examination will be conducted
- Vital signs (BP, pulse) and weight will be assessed and recorded.
- Review and updating of concomitant medication and recording of relevant medications
- Review and recording of any cardiac and HF related procedures
- Review of potential efficacy and safety events
- If the patient has experienced any potential endpoints, SAEs, DAEs and/or amputations, adverse events (AEs) leading to amputation and potential risk factor AEs for amputations affecting lower limbs since the last visit, this will be recorded in the eCRF
- Drug accountability of the returned IP will be checked

8.2.9 Unscheduled visits

An unscheduled on-site or telephone visit may occur in-between scheduled on-site visits (for example assessment of potential endpoint events or safety events).

8.3 Efficacy assessments

8.3.1 Efficacy event capture

Efficacy events (i.e. death, hospitalisation or urgent visits for HF) will be collected by site personnel according to the study visit schedule. All potential efficacy events should be recorded as an AE and on additional event modules in the eCRF. If the potential efficacy event fulfils SAE criteria (see Appendix B 2) the site is to record and report these events to the sponsor or designee within timelines described in Section 8.6.

NYHA classification will be done by the Investigators and recorded in the eCRF. PROs will be collected for all patients throughout the study period via a hand-held electronic device. All-cause hospitalisations will be derived from SAE reports.

8.3.2 Efficacy event adjudication

A Clinical Events Adjudication (CEA) Committee will be established for this trial and adjudicate primary efficacy events in accordance with adjudication criteria detailed in the CEA charter.

Events to be adjudicated include components of the primary efficacy endpoint: deaths, hospitalisation for HF, and urgent HF visits. All deaths will be adjudicated to determine if they are CV or non-CV deaths. All adjudication will be done on an ongoing basis throughout the trial.

8.3.3 Clinical Outcome Assessments (COA)

A COA is any assessment that may be influenced by human choices, judgement, or motivation and may support either direct or indirect evidence of treatment benefit. Patient Reported Outcomes (PROs) is one of the types of COAs. A PRO is any report of the status of a patient's health condition that comes directly from the patient, without interpretation of anyone else. PROs have become a significant endpoint when evaluating benefit/risk of treatments in clinical trials. The following PROs will be collected: KCCQ, PGIS and EQ-5D-5L (see Appendix J, Appendix L, Appendix M).

PROs will be collected for all patients throughout the study period via a hand-held electronic device. See study of assessment (See Table 1) for the timing of collection. The ePRO devices should be administered prior to first dose at visit 2/randomisation. Site staff should stress that the information is confidential.

8.3.3.1 KCCQ

The Kansas City Cardiomyopathy Questionnaire (KCCQ) is a 23-item, self-administered disease specific instrument and has shown to be a valid, reliable and responsive measure for patients with HF (Greene at al 2000, Spertus et al 2005). The KCCQ was developed to independently measure the patient's perception of their health status, which includes heart failure-related symptoms (frequency, severity and recent change), impact on physical and social function, self-efficacy and knowledge, and how their heart failure impacts their quality of life (QOL). Scores are transformed to a range of 0-100. Higher scores represent a better outcome.

The KCCQ tool quantifies the following six (6) distinct domains and two (2) summary scores:

- KCCQ Symptom Domain quantifies the frequency and burden of clinical symptoms in heart failure, including fatigue, shortness of breath, paroxysmal nocturnal dyspnea and patients' edema/swelling. An overall symptom score is generally used in analyses; subscale scores for both frequency and severity are also available. The total symptom Score incorporates the symptom domains into a single score

- KCCQ Physical Function Domain measures the limitations patients experience, due to their heart failure symptoms, in performing routine activities. Activities are common, gender-neutral, and generalizable across cultures, while also capturing a range of exertional requirements

- KCCQ Quality of Life Domain is designed to reflect patients' assessment of their quality of life, given the current status of their heart failure

- KCCQ Social Limitation Domain quantifies the extent to which heart failure symptoms impair patients' ability to interact in a number of gender-neutral social activities

- KCCQ Self-efficacy Domain quantifies patients' perceptions of how to prevent heart failure exacerbations and manage complications when they arise. This scale is not included in the summary scores

- KCCQ Symptom Stability Domain measures recent changes in patients' symptoms; their shortness of breath, fatigue or swelling. It compares patients frequency of heart failure symptoms at the time of completing the KCCQ with their frequency 2 weeks ago. As a measure of change, it is most interpretable as a baseline assessment of the stability of patients' symptoms at the start of a study and shortly thereafter, as a measure of the acute response to treatment. This domain is not included in the summary scores.

- Clinical Summary Score includes total symptom and physical function scores to correspond with NYHA Classification

- Overall Summary Score includes the total symptom, physical function, social limitations and quality of life scores

8.3.3.2 Patient Global Impression of Severity (PGIS)

The PGIS item is included to assess how a patient perceives his/her overall current severity of heart failure symptoms. Patients will choose from response options from "no symptoms" to "very severe"

8.3.3.3 EuroQoL five-dimensional five-level questionnaire (EQ-5D-5L)

The EQ-5D-5L is a self-reported questionnaire that is used to derive a standardized measure of health status, also referred to as a utility score. EQ-5D-5L utility scores are widely accepted by reimbursement authorities and will be used to support health economic evaluations.

8.3.3.4 Administration of electronic PROs

Each site must allocate the responsibility for the administration of the ePROs to a specific individual and, if possible, assign a backup person to cover if that individual is absent. A key aspect of study success is to have high PRO compliance. Therefore, it is essential to follow SoA and that sites make sure the device is charged and fully functional at all times in order to minimize missing data.

It is important that the site staff explains the value and relevance of PRO data: to hear directly from patients how they feel. The following best practice guidelines should be followed:

- Patient must not receive help from relatives, friends, or site personnel to answer or clarify the PRO questionnaires in order to avoid bias. If a patient uses visual aids (e.g., spectacles or contact lenses) for reading and does not have them at hand, the patient will be exempted from completing the PROs questionnaires on that visit

- Before any other study procedures are conducted at a given visit (except the Visit 2: eligibility confirmation before the KCCQ)

- Before being seen by the investigator

- PRO questionnaires must be completed by the patient in private

- The appointed site personnel should also stress that the information is confidential. Therefore, if the patient has any medical problems, he or she should discuss them with the doctor or research nurse separately from the ePRO assessment

- The appointed site personnel must show patients how to use the ePRO device, in accordance with the instructions provided

- The appointed site personnel should remind patients that there are no right or wrong answers, and the patient should be given sufficient time to complete the PRO questionnaires at his/her own speed

If the patient is unable to read the questionnaire (e.g., is blind or illiterate), the patient will be exempted from completing the PRO questionnaires and may still participate in the study. Patients exempted in this regard should be flagged appropriately by the site personnel.

8.4 Safety assessment

Planned time points for all safety assessments are provided in the schedule of activities (Table 1).

8.4.1 Physical examinations

A physical examination will be performed at the time-points specified in Table 1 and include an assessment of the following: general appearance, respiratory and cardiovascular systems (including oedema) and abdomen.

The assessment dates will be recorded in the eCRF.

8.4.2 Vital Signs

- Pulse and BP will be measured twice at all applicable visits, and all measurements will be recorded in the eCRF.

- The measurements should be done before any blood sampling. The measurements will be assessed in a sitting position with a completely automated device. Manual techniques will be used only if an automated device is not available.

- The measurements should be preceded by at least 5 minutes of rest for the subject in a quiet setting without distractions (e.g., television, cell phones).

8.4.3 Electrocardiogram

A 12-lead ECG (standard ECG with a paper speed of 25-50 mm/second covering at least 6 sequential beats) will be recorded at baseline (Visit 1) after the patient has been lying down to rest for at least 5 minutes, to confirm presence or absence of atrial fibrillation/flutter at enrolment. t rhythm will be reported in the eCRF. The baseline ECG should be stored and be made available upon request for adjudication purposes.

8.4.4 Safety laboratory assessments

Serum creatinine will be collected for calculation of eGFR using CKD-EPI equation (Levey at al 2009).

8.4.5 Other safety assessments (not applicable)

8.4.6 Other clinical assessments

8.4.6.1 Body weight and height

The patient's body weight will be measured with light clothing and no shoes. If the patient has a prosthetic limb, this should be consistently worn during all weight measurements. The patient's height will be measured at Visit 1, with no shoes. The weight and height will be recorded in the eCRF.

8.5 Collection of adverse events

The Principal Investigator is responsible for ensuring that all staff involved in the study are familiar with the content of this section.

The definitions of an AE or SAE can be found in Appendix B.

AE will be reported by the patient (or, when appropriate, by a caregiver, surrogate, or the patient's legally authorised representative).

The Investigator and any designees are responsible for detecting, documenting, and recording events that meet the definition of a SAEs and DAEs, amputation and events potentially placing the patient at risk for a lower limb amputation (preceding events). For information on how to follow-up AEs see Section 8.5.3.

8.5.1 Method of detecting AEs and SAEs

Care will be taken not to introduce bias when detecting SAEs or DAEs. Open-ended and non-leading verbal questioning of the patient is the preferred method to inquire about AE occurrences.

Safety information on SAEs and DAEs, amputations, adverse events (AEs) leading to amputation and potential risk factor AEs for amputations affecting lower limbs will be collected and entered into eCRFs by site personnel according to the study visit schedule.

If the potential efficacy event fulfils SAE criteria (see Appendix B 2) the site is to record and report these events to the Sponsor or designee within timelines described in Section 8.6.1.

8.5.1.1 Adverse events (AEs) leading to amputation and potential risk factor AEs for amputations affecting lower limbs ("preceding events")

To ensure that data on amputations is systematically collected, amputations and underlying conditions relevant to amputation will be recorded on a specific eCRF page. The adverse event leading to amputation should be recorded in the eCRF as AE/SAE.

In addition to amputation, non-serious and serious events potentially placing the patient at risk for a lower limb amputation (“preceding events”) should also be recorded in the eCRF as AE/SAE whether or not an amputation has taken place. The lower limb amputation “preceding events” of interest include diabetic foot related conditions, vascular, volume depletion, wounds/injury/trauma, infection and neuropathy. If any of these or other potentially relevant events have occurred, relevant information must be provided (this will be collected on a dedicated eCRF page - for details see eCRF instruction)”.

8.5.1.2 Capture of DKA events

For SAEs or DAEs reported by the Investigator as Diabetic Ketoacidosis (DKA - see definition below) additional information will be recorded on specific eCRF pages in addition to the AE/SAE form.

8.5.1.3 DKA definition

A diagnosis of Diabetic Ketoacidosis should only be made in a clinical setting consistent with DKA (based on patient history, symptoms, and physical exam) and in the absence of more likely alternative diagnoses and causes of acidosis (such as lactic acidosis). The following biochemical data should support diagnosis:

- Ketonaemia ≥3.0 mmol/L and/or significant ketonuria (more than 2+ on standard urine sticks)
- At least one of the following criteria suggesting high anion gap metabolic acidosis:
  - Arterial or Venous pH ≤7.3
  - Serum bicarbonate ≤18 mEq/L
  - Anion gap [Na – (Cl + HCO3)] >10

8.5.1.4 Capture of cardiac ischaemic events and stroke

For myocardial infarctions, unstable angina and stroke additional information will be recorded on specific eCRF pages in addition to the AE/SAE form.

The diagnosis of stroke, MI and unstable angina should be made according to standard clinical practice and align with the definition for stroke in the standardised definitions for endpoints (Hicks et al. 2018
Hicks KA, Mahaffey KW, Mehran R, Nissen SE, et alCardiovascular and Stroke Endpoint Definitions for Clinical Trials. J Am Coll Cardiol 2018;71:1021–34) described in Appendix C.

8.5.1.5 Capture of additional laboratory values

Any additional safety laboratory assessments during the study period, including creatinine, will be obtained per the Investigator’s medical judgment in the course of standard care using local laboratories. Laboratory values would be recorded only on SAE eCRFs as part of narrative information, per the Investigator’s judgment.

8.5.2 Time period and frequency for collecting AE and SAE information

Non- serious adverse events as defined per protocol will be collected from randomisation (Visit 2), throughout the treatment period until and including the patient’s last visit (the study closure

visit). Serious adverse events are recorded from the time of signing of informed consent form throughout the treatment period until and including the patient's last visit.

All SAEs will be recorded and reported to the sponsor or designee within 24 hours, as indicated in Appendix B. The Investigator will submit any updated SAE data to the sponsor within 24 hours of it being available.

The method of recording, evaluating, and assessing causality of AE and SAE and the procedures for completing and transmitting SAE reports are provided in Appendix BAppendix B.

8.5.3 Follow-up of AEs and SAEs

After the initial AE/SAE report, the Investigator is required to proactively follow each patient at subsequent visits/contacts. All SAE and events of amputation and potential preceding events will be followed until resolution, stabilization, the event is otherwise explained, or the patient is lost to follow-up.

Any AEs that are unresolved at the patient's last visit in the study are followed up by the Investigator for as long as medically indicated, but without further recording in the CRF. AstraZeneca retains the right to request additional information for any patient with ongoing AE(s)/SAE(s) at the end of the study, if judged necessary.

8.5.4 Adverse event data collection

The following variables will be collect for each AE;

- AE (verbatim)
- The date when the AE started and stopped
- Maximum intensity (mild/moderate/severe)
- Whether the AE is serious or not
- Investigator causality rating against the Investigational Product(s) (yes or no)
- Action taken with regard to IP
- Outcome

In addition, the following variables will be collected for SAEs:

- Date AE met criteria for serious AE
- Date Investigator became aware of serious AE
- AE is serious due to
- Date of hospitalisation
- Date of discharge
- Probable cause of death

- Date of death
- Autopsy performed
- Causality assessment in relation to Study procedure(s) and/or other medication
- Description of AE

8.5.5 Causality collection

The Investigator will assess causal relationship between the IP and each AE and answer 'yes' or 'no' to the question 'Do you consider that there is a reasonable possibility that the event may have been caused by the investigational product?'

For SAEs, causal relationship will also be assessed for other medication and study procedures. Note that for SAEs that could be associated with any study procedure the causal relationship is implied as 'yes'.

A guide to the interpretation of the causality question is found in Appendix B to the Clinical Study Protocol.

8.5.6 Adverse events based on signs and symptoms

All AEs spontaneously reported by the patient or reported in response to the open question from the study personnel: 'Have you had any health problems since the previous visit/you were last asked?' or revealed by observation will be collected and recorded in the eCRF if they fulfil the criteria specified in Section 8.5.2. When collecting AEs, the recording of diagnoses is preferred (when possible) to recording a list of signs and symptoms. However, if a diagnosis is known and there are other signs or symptoms that are not generally part of the diagnosis, the diagnosis and each sign or symptom will be recorded separately.

8.5.7 Adverse events based on examinations and tests

The results from the Clinical Study Protocol mandated vital signs and laboratory values will be summarised in the clinical study report. Deterioration as compared with baseline in protocol-mandated vital signs should therefore only be reported as AEs if they fulfil any of the SAE criteria or are the reason for discontinuation of treatment with the IP. If deterioration in a vital sign is associated with clinical signs and symptoms, the sign or symptom will be reported as an AE if they fulfil any of the SAE criteria or are the reason for discontinuation of treatment of IP, and the associated vital sign will be considered as additional information.

8.5.8 Disease-under study (DUS) (not applicable)

8.5.9 Disease progression (not applicable)

8.6 Safety reporting and medical management

8.6.1 Reporting of serious adverse events

All SAEs have to be reported, whether or not considered causally related to the investigational product, or to the study procedure(s). All SAEs will be recorded in the eCRF.

If any SAE occurs in the course of the study, then Investigators or other site personnel inform the appropriate AstraZeneca representatives within one day i.e., immediately but **no later than 24 hours** of when he or she becomes aware of it.

The designated AstraZeneca representative works with the Investigator to ensure that all the necessary information is provided to the AstraZeneca Patient Safety data entry site **within 1 calendar day** of initial receipt for fatal and life-threatening events **and within 5 calendar days** of initial receipt for all other SAEs.

For fatal or life-threatening adverse events where important or relevant information is missing, active follow-up is undertaken immediately. Investigators or other site personnel inform AstraZeneca representatives of any follow-up information on a previously reported SAE within one calendar day i.e., immediately but **no later than 24 hours** of when he or she becomes aware of it.

Once the Investigators or other site personnel indicate an AE is serious in the EDC system, an automated email alert is sent to the designated AstraZeneca representative.

If the EDC system is not available, then the Investigator or other study site staff reports a SAE to the appropriate AstraZeneca representative by telephone.

The AstraZeneca representative will advise the Investigator/study site staff how to proceed.

Investigators or other site personnel send relevant CRF modules by fax to the designated AstraZeneca representative.

For further guidance on the definition of a SAE, see Appendix B of the Clinical Study Protocol.

8.6.1.1 Reporting of SAEs considered to be potential endpoints

In order to avoid unnecessary unblinding of efficacy endpoint events, certain SAEs which are also potential endpoints (i.e., fatal AEs and HF events) will not be reported to health authorities. Clinical data for the above mentioned events will be recorded as AEs/SAEs as well as on separate event forms in the eCRF. Recording of a suspected endpoint should be done within the same timeframes as defined for SAEs (see Section 8.6.1).

In addition, fatal AEs and potential HF endpoints will be centrally adjudicated by an independent CEA committee (see Section 8.3.1 and 8.3.2). If adjudication confirms the endpoint, the SAE will not be reported to health authorities. However, if it is determined by the CEA committee that a potential endpoint does not meet the endpoint criteria, the event will be reported (according to the timelines specified in Section 8.6.1) to AZ patient safety data entry site and if applicable to the health authorities (note that the clock starts when the adjudication results are available).

8.6.2 Pregnancy

All pregnancies and outcomes of pregnancy should be reported to AstraZeneca except if the pregnancy is discovered before the study patient has received any IP. If a pregnancy is reported, the Investigator should inform the sponsor within 24 hours of learning of the pregnancy. Abnormal

pregnancy outcomes (e.g. spontaneous abortion, foetal death, stillbirth, congenital anomalies, ectopic pregnancy) are considered SAEs.

8.6.2.1 Maternal exposure

Women of childbearing potential who are not using contraception as defined in Section 5.2; exclusion criterion number 18 are not allowed to be included in this study. Should a pregnancy still occur, the investigational product should be discontinued immediately and the pregnancy reported to AstraZeneca.

Dapagliflozin must not be used in the second and third trimesters of pregnancy. In the time period corresponding to second and third trimester of pregnancy with respect to human renal maturation, maternal exposure to dapagliflozin in rat studies was associated with increased incidence and/or severity of renal pelvic and tubular dilatations in progeny.

There are no adequate and well-controlled studies of dapagliflozin in pregnant women. When pregnancy is detected, investigational product(s) should be discontinued.

8.6.3 Overdose

Dapagliflozin has been well tolerated at doses of up to 500 mg/day in single dose testing in healthy volunteers and up to 100 mg/day in repeat dose testing for 14 days in healthy volunteers and patients with T2D. Suspected single intake of more than 50 tablets of 10 mg dapagliflozin tablets or repeated intake of more than 10 tablets of 10 mg dapagliflozin tablets should be reported on the eCRF overdose module. If an overdose is suspected, monitoring of vital functions as well as treatment should be performed as appropriate.

For further information regarding overdose, refer to the IB.

- An overdose without associated symptoms is only recorded on the Overdose eCRF module
- An overdose with associated AEs is recorded as the AE diagnosis/symptoms on the relevant AE modules in the eCRF and on the Overdose eCRF module

If an overdose on an AstraZeneca IP occurs in the course of the study, then the investigator or other site personnel inform appropriate AstraZeneca representatives immediately, or no later than 24 hours of when he or she becomes aware of it.

The designated AstraZeneca representative works with the investigator to ensure that all relevant information is provided to the AstraZeneca Patient Safety data entry site.

For overdoses associated with a SAE, the standard reporting timelines apply, see Section 8.5.2. For other overdoses, reporting must occur within 30 days.

8.7 Pharmacokinetics (not applicable)

8.8 Pharmacodynamics (not applicable)

8.9 Optional exploratory genetics

Approximately 6 mL blood sample for DNA isolation will be collected from subjects who have consented to participate in the genetic analysis component of the study. Participation is optional. Subjects who do not wish to participate in the genetic research may still participate in the study.

See Appendix D for Information regarding genetic research. Details on processes for collection and shipment and destruction of these samples can be found in Appendix D.

8.10 Biomarkers (not applicable)

8.11 Health Economics (not applicable)

9. STATISTICAL CONSIDERATIONS

9.1 Statistical hypotheses

For the primary and secondary endpoints, the following hypothesis will be tested at the 4.980 % 2-sided level:

H0: HR [dapagliflozin:placebo] =1

versus

H1: HR [dapagliflozin:placebo] ≠1

9.2 Sample size determination

The primary objective of the study is to determine the superiority of dapagliflozin versus placebo added to standard of care in reducing the composite of CV death and heart failure events (hospitalisation for HF or urgent HF visit). Assuming a true hazard ratio (HR) of 0.80 between dapagliflozin and placebo, using a two-sided alpha of 5%, 844 primary endpoint events will provide a statistical power of 90% for the test of the primary composite endpoint. This is based on an overall 1:1 allocation between dapagliflozin and placebo.

The HR was chosen as a conservative assumption based on the observed HR 0.72 (95% confidence interval 0.50-1.04) for the composite of HF hospitalisation and CV death in patients with HF at baseline in the EMPA-REG OUTCOME trial (Fitchett et al 2016) and HR 0.61 (0.46-0.80) for patients with history of HF in the CANVAS program (Rådholm et al 2018) considering that these were post-hoc analyses in subgroups with limited documentation of baseline HF diagnosis, not characterised by ejection fraction.

The event rate assumptions are based on sub analyses of the TOPCAT and I-PRESERVE studies by geographic region, NT-proBNP levels, prior hospitalisation for HF, and T2D status (Pfeffer et al 2015, Kristensen et al 2015 Kristensen et al 2017). The sample size calculation builds on the assumption of an annual event rate of 9% in the placebo group for the majority of prevalent HFpEF patients, importantly all with NT-proBNP ≥300 pg/ml by inclusion criterion. Additionally, a subgroup of patients due to be discharged or recently discharged from a HF hospitalisation (here

denoted 'subacute' patients) with a higher event rate is planned to be included. Assuming 20% of patients from the sub-acute category with an annual event rate of 24% during the first year and 9% thereafter for the remainder of the study, (corresponding to an annualised rate of approximately 17% for sub-acute patients), approximately 4700 patients are estimated to provide the required number of 844 patients with a primary event during an anticipated recruitment period of 18 months and a minimal follow-up period of 15 months (total study duration 33 months, average follow-up 24 months). The study is event driven and the number of patients or duration may change if the event rate is lower than anticipated.

In addition, the expected number of patients who will be lost to follow-up is expected to be small; hence, these are not considered in the determination of the sample size.

9.3 Populations for analyses

For purposes of analysis, the following populations are defined:

Table 5 Population for analysis

Population	Description
Enrolled	All patients who sign the ICF
Full Analysis Set (FAS)	All patients who have been randomised to study treatment, irrespective of their protocol adherence and continued participation in the study.<br>Patients will be analysed according to their randomised investigational product assignment, irrespective of the treatment actually received. The FAS will be considered the primary analysis set for the intention to treat analysis of primary and secondary variables.
Safety analysis set	All patients randomly assigned to Study treatment and who take at least 1 dose of investigational product. Patients will be analysed according to the treatment actually received. The Safety analysis set will be considered the primary analysis set for all safety variables

9.4 Statistical analyses

All personnel involved with the analysis of the study will remain blinded until database lock and Clinical Study Protocol deviations identified.

Analyses will be performed by AstraZeneca or its representatives.

A comprehensive statistical analysis plan (SAP) will be developed prior to first patient randomised and any subsequent amendments will be documented, with final amendments finalised before database lock. This section is a summary of the planned statistical analyses of the primary and secondary endpoints. Any deviations from this plan will be reported in the clinical study report.

9.4.1 Efficacy analyses

9.4.1.1 Analysis of the primary variable

The primary variable is the time from randomisation to first event included in the primary composite endpoint. The primary analysis will be based on the ITT principle using the FAS, including events occurring on or prior to the PACD, adjudicated by the CEA committee.

In the analysis of the primary composite endpoint, treatments (dapagliflozin versus placebo) will be compared using a Cox proportional hazards model with a factor for treatment group, stratified by T2D status at randomisation. The p-value, HR and 95% confidence interval will be reported.

The contribution of each component of the primary composite endpoint to the overall treatment effect will be examined. Methods similar to those described for the primary analysis will be used to separately analyse the time from randomisation to the first occurrence of each component of the primary composite endpoint. HR and 95% confidence intervals will be reported.

Kaplan-Meier estimates of the cumulative incidence to the first occurrence of any event in the primary endpoint will be calculated and plotted, for overall analysis and for the individual components.

9.4.1.2 Analysis of the secondary variables

The outcome of all HF hospitalisations (first and recurring) and CV death will be analysed by the semi-parametric proportional rates model (Lin et al 2000) to test the treatment effect and to quantify the treatment difference. The rate ratio and its 95% confidence interval and corresponding two-sided p-value will be presented.

The proportion of patients with worsening NYHA classification from baseline to 8 months will be analysed by a logistic regression with treatment group, baseline NYHA and T2D at randomisation as factors. The odds ratio between treatment groups, its 95% confidence interval and corresponding two-sided p-value will be presented.

The analysis of change from baseline for KCCQ total symptom score at 8 months will be further detailed in the statistical analysis plan, e.g. with consideration of handling of patients who die. In addition to the secondary endpoint, total symptom score, the overall summary score, clinical summary score and domain scores will be analysed. A responder analysis will also be performed (more details presented in the SAP).

The analysis of time from randomisation to all-cause mortality will be analysed in the similar manner as the primary variable.

9.4.1.3 Subgroup analysis

Subgroup variables for the primary efficacy endpoint include demography, baseline disease characteristics, baseline concomitant medications and others. Cox proportional hazard model stratified for T2D with factors for treatment group, the subgroup variable and the interaction between treatment and subgroup will be used to examine treatment effects within relevant subgroups separately. A test of interaction between randomised treatment group and the subgroup

variable will be performed in each Cox model. The p-values for the subgroup analyses will not be adjusted for multiple comparisons as the tests are exploratory and will be interpreted descriptively. Treatment differences with 95% confidence intervals will be reported for each subgroup. HRs and CIs for overall analysis and subgroups will be presented with forest plots as well. Further details of the subgroup analysis, including the list of subgroup variables, will be provided in the SAP.

9.4.1.4 Sensitivity analysis

Details of the sensitivity analysis for the primary and secondary endpoints will be provided in the SAP.

9.4.2 Safety analyses

All safety analyses will be performed on the Safety analysis set. The number and percent of patients with SAEs, DAEs, amputations, and potential preceding events for lower limb amputations will be summarised by treatment group, and by system organ class and preferred term.

For safety analyses, summaries will be provided using both on treatment observations and using all observations regardless of whether patients are on or off study treatment.

9.4.3 Methods for multiplicity control

A closed testing procedure including a pre-specified hierarchical ordering of the primary and secondary endpoints will be utilised. The Type I error will be controlled at an overall two-sided 5% level for multiplicity across primary and secondary endpoints and in consideration of the planned interim analysis. With one interim analysis at 67%of events (see Section 9.5) the two-sided significance level in final analysis, α, will be 4.980%. Statistical significance will be assessed in the pre-specified order of the endpoints as specified in Section 3. If the primary endpoint is significant at level α, then the first secondary endpoint, recurrent HF hospitalisations and CV Death, will be tested at level α. If the first secondary endpoint is significant, then the α will be split between KCCQ total symptom score and NYHA class. If one of them is significant at level α/2, then the other can be tested at level α. If both KCCQ and NYHA class reach statistical significance, then all-cause mortality will be tested at significance level α.

9.5 Interim analyses

An interim analysis is planned to be performed including approximately 67% of the target number of patients with adjudicated primary endpoint events. There will in principle be one planned interim analysis for efficacy, with the possibility of the DMC to conduct subsequent interim analysis if they deem necessary. The significance level for final analysis will be determined by the Haybittle-Peto function based on the actual number of interim analyses. The interim analysis will assess superiority of dapagliflozin to placebo. The interim analysis will have a nominal two-sided alpha level of 0.2%. At the interim analysis, the primary composite endpoint will be tested first at the specified alpha level. If superiority is achieved for the primary endpoint, then the superiority of dapagliflozin to placebo on CV deaths will be tested at a two-sided level of 0.2%. If CV death is significant, then an action is triggered whereby the DMC will evaluate the totality of the efficacy data and safety data, to determine if benefit is unequivocal and overwhelming such that the DMC recommends ending the study.

A futility analysis is planned to be performed at the same time as the planned interim analysis. The study may be stopped for futility if the observed HR is > 0.946, corresponding to a predictive power of 5%. If the futility criterion of the primary endpoint is met, then DMC will evaluate the totality of data, including potential benefits on patient reported outcomes to consider recommending ending the study for futility.

9.5.1 Data monitoring committee (DMC)

An independent data monitoring committee (DMC) will be appointed and will report to the Executive Committee. The DMC will be responsible for safeguarding the interests of the patients in the outcome study by assessing the safety of the intervention during the study. The DMC will have access to the individual treatment codes and be able to merge these with the collected study data while the study is ongoing. A charter will be prepared to detail precise roles and responsibilities and procedures of the DMC.

10. REFERENCES

Ambrosy et al 2014
Ambrosy AP, Gheorghiade M, Chioncel O, Mentz RJ, Butler J. Global perspectives in hospitalised heart failure: regional and ethnic variation in patient characteristics, management, and outcomes. Curr Heart Fail Rep 2014 Dec;11(4):416-27.

Borlaug and Paulus 2011
Borlaug BA, Paulus WJ. Heart failure with preserved ejection fraction: pathophysiology, diagnosis, and treatment. Eur Heart J 2011;32:670-9.

Braunwald 2015
Braunwald E. The war against heart failure: the Lancet lecture. Lancet 2015;385:812-24.

Butler et al 2014
Butler J, Fonarow GC, Zile MR, Lam CS, Roessig L, Schelbert EB, et al. Developing therapies for heart failure with preserved ejection fraction: current state and future directions. JACC Heart Fail 2014 Apr;2(2):97-112.

Cherney et al 2014
Cherney DZ, Perkins BA, Soleymanlou N, Har R, Fagan N, Johansen OE, et al. The effect of the empagliflozin on arterial stiffness and heart rate variability in subjects with uncomplicated type 1 diabetes mellitus. Cardiovasc Diabetol 2014;13:28.

Chilton et al 2015
Chilton R, Tikkanen I, Cannon CP, Crowe S, Woerle HJ, Broedl UC, et al. Effects of empagliflozin on blood pressure and markers of arterial stiffness and vascular resistance in patients with type 2 diabetes. Diabetes Obes Metabo 2015;17:1180-93.

Cook et al 2014
Cook C, Cole G, Asaria P, Jabbour R, Francis DP. The annual global economic burden of heart failure. Int J Cardiol 2014 Feb 15;171(3):368-76.

EMA Guidance 2016
EMA 2016; Guideline on clinical investigation of medicinal products for the treatment of chronic heart failure (draft), Committee for Medicinal Products for Human Use (CHMP). EMA/CHMP/392958/2015.

FDA Draft Guidance for Industry 2016
Food and Drug Administration. Use of real-world evidence to support regulatory decision-making for medical devices. Draft Guidance for Industry and Food and Drug Administration Staff. 16 September 2016.

Fitchett et al 2016
Fitchett D, Zinman B, Wanner C, Lachin JM, Hantel S, Salsali A, et al. Heart failure outcomes with empagliflozin in patients with type 2 diabetes at high cardiovascular risk: results of the EMPA-REG OUTCOME® trial. Eur Heart J 2016;14;37(19):1526-34.

Greene at al 2000
Greene SJ, Mentz RJ, Felker GM. Outpatient Worsening Heart Failure as a Target for Therapy: A Review. JAMA Cardiol. 2018 Jan 31. doi:10.1001/jamacardio.2017.5250. [Epub ahead of print] PubMed PMID: 29387880.

Hicks et al 2014
Hicks KA, Hung HMJ, Mahaffey KW, Mehran R, Nissen SE et al, on behalf of the Standardized Data Collection for Cardiovascular Trials Initiative. Standardized Definitions for Cardiovascular and Stroke Endpoint Events in Clinical Trials. Draft Definitions for CDISC August 20, 2014.

Hicks et al. 2018
Hicks KA, Mahaffey KW, Mehran R, Nissen SE, et alCardiovascular and Stroke Endpoint Definitions for Clinical Trials. J Am Coll Cardiol 2018;71:1021–34

Inzucchi et al 2012
Inzucchi SE, Bergenstal RM, Buse J.B, Diamant M, Ferrannini E, Nauck M et al. Management of hyperglycaemia in type 2 diabetes: a patient-centered approach. Position statementof the American Diabetes Association (ADA) and the European Association for the Study of Diabetes (EASD). Diabetologia.

Inzucchi et al 2015
Inzucchi Silvio E Bergenstal Richard M, Buse John B, Diamant Michaela, Ferrannini Ele, Nauck Michael, Peters Anne L, Tsapas Apostolos, Wender Richard,and Matthews David R. Management of Hyperglycemia in Type 2 Diabetes, 2015: A Patient-Centered Approach. Update to a Position Statement of the American Diabetes Association and the European Association for the Study of Diabetes. Diabetes Care 2015;38:140–149

Iwaz et al 2016
Iwaz JA, Le E, Aramin H, Romero D, Iqbal N, Kawahara M, et al. New targets in the drug treatment of heart failure. Drugs 2016;79:187-201.

Kosiborod et al 2016
Kosiborod M, Xu J, Sjostrand M, Sjostrom CD Safety and Efficacy of Dapagliflozin in Combination with Potassium-sparing Agents. Abstr, 1094-P ADA, June 2016, New Orleans

Kosiborod et al 2017a
Kosiborod M, Cavender MA, Fu AZ, Wilding JP, Khunti K, Holl RW, et al, on behalf of the CVD-REAL Investigators and Study Group. Lower risk of heart failure and death in patients initiated on SGLT-2 inhibitors versus other glucose-lowering drugs: the CVD-REAL study. Circulation 2017 May 18. pii: CIRCULATIONAHA.117.029190. doi: 10.1161/CIRCULATIONAHA.117.029190. [Epub ahead of print]

Kosiborod et al 2017b
Kosiborod M, Gause-Nilsson I, J Xu J, Sonesson C, Johnsson E. Efficacy and safety of dapagliflozin in patients with type 2 diabetes mellitus and concomitant heart failure. J Diabetes Complications 2017;31(7):1215-21.

Kristensen et al 2015
Kristensen SL, Jhund PS, Køber L, McKelvie RS, Zile MR, Anand IS, et al. Relative importance of history of heart failure hospitalisation and N-terminal Pro–B-type natriuretic peptide level as predictors of outcomes in patients With heart failure and preserved ejection fraction. JACC Heart Fail 2015 Jun;3(6):478-86.

Kristensen et al 2016
Kristensen SL, Preiss D, Jhund PS, Squire I, Cardoso JS et al. Risk related to pre–diabetes mellitus and diabetes mellitus in heart failure with reduced ejection fraction insights from prospective comparison of ARNI with ACEI to determine impact on global mortality and morbidity in heart failure trial. Circ Heart Fail. 2016;9:e002560.

Kristensen et al 2017
Kristensen SL, Mogensen UM, Jhund PS, Petrie MC, Preiss D1, Win S, et al. Clinical and echocardiographic characteristics and cardiovascular outcomes according to diabetes status in patients with heart failure and preserved ejection fraction: a report from the I-Preserve Trial (Irbesartan in Heart Failure With Preserved Ejection Fraction). Circulation. 201721;135(8):724-35.

Lam et al 2011
Lam CSP, Donal E, Kraigher-Krainer E, Vasan RS. Epidemiology and clinical course of heart failure with preserved ejection fraction. European Journal of Heart Failure 2011; 13: 18–28.

Levey at al 2009
Levey AS, Stevens LA, Schmid CH, Zhang YL, Castro AF 3rd, Feldman HI et al. A new equation to estimate glomerular filtration rate. Ann Intern Med. 2009 May 5; 150(9):604-12.

Lin et al 2000
Lin DY, Wei LJ, Yang I, Ying Z. Semiparametric regression for the mean and rate functions of recurrent events. Journal of the Royal Statistical Society: Series B (Statistical Methodology) 2000; 62(4):711–730.

Neal et al 2017
Neal B, Perkovic V, Mahaffey KW, de Zeeuw D, Fulcher G, Erondu N, et al, for the CANVAS Program Collaborative Group. Canagliflozin and cardiovascular and renal events in type 2 diabetes. 2017; 377: 644-657.

Oktay et al 2013
Oktay AA, Rich JD, Shah SJ. The emerging epidemic of heart failure with preserved ejection fraction. Curr Heart Fail Rep 2013;10(4):401-10.

Okumura et al 2016
Okumura N, Jhund PS, Gong J, Lefkowitz MP, Rizkala AR, Rouleau JL, Shi VC, Swedberg K, Zile MR, Solomon SD, Packer M, McMurray JJ; PARADIGM-HF Investigators and Committees*. Importance of Clinical Worsening of Heart Failure Treated in the Outpatient Setting: Evidence From the Prospective Comparison of ARNI With ACEI to Determine Impact on Global Mortality and Morbidity in Heart Failure Trial

(PARADIGM-HF). Circulation. 2016 Jun 7;133(23):2254-62. doi: 10.1161/CIRCULATIONAHA.115.020729. Epub 2016 Apr 20. PubMed PMID: 27143684.Oktay AA, Rich JD, Shah SJ. The emerging epidemic of heart failure with preserved ejection fraction. Curr Heart Fail Rep 2013;10(4):401-10.

Ott et al 2017
Ott C, Jumar A, Striepe K, Friedrich S, Karg MV, Bramlage P, et al. A randomised study of the impact of the SGLT2 inhibitor dapagliflozin on microvascular and macrovascular circulation. Cardiovasc Diabetol 2017;16:26.

Pfeffer et al 2015
Pfeffer MA, Claggett B, Assmann SF, Boineau R, Anand IS, Clausell N, et al. Regional Variation in Patients and Outcomes in the Treatment of Preserved Cardiac Function Heart Failure With an Aldosterone Antagonist (TOPCAT) Trial. Circulation. 2015;131:34-42.

Rådholm et al 2018
Rådholm K, Figtree, Perkovic V, Solomon SD, Mahaffey KW, et al. Canagliflozin and Heart Failure in Type 2 Diabetes Mellitus: Results From the CANVAS Program (Canagliflozin Cardiovascular Assessment Study). Circulation. 2018;137
https://doi.org/10.1161/CIRCULATIONAHA.118.034222

Shigiyama et al 2017
Shigiyama F, Kumashiro N, Miyagi M, Ikehara K, Kanda E, Uchino H, et al. Effectiveness of dapagliflozin on vascular endothelial function and glycemic control in patients with early-stage type 2 diabetes mellitus: DEFENCE study. Cardiovasc Diabetol 2017;16:84. DOI 10.1186/s12933-017-0564-0.

Skali et al 2014
Skali H, Dwyer EM, Goldstein R, Haigney M, Krone R, Kukin M, Lichstein E, McNitt S, Moss AJ, Pfeffer MA, Solomon SD. Prognosis and response to therapy of first inpatient and outpatient heart failure event in a heart failure clinical trial: MADIT-CRT. Eur J Heart Fail. 2014 May;16(5):560-5. doi: 10.1002/ejhf.71. Epub 2014 Feb 27. PubMed PMID: 24578164.

Solomon et al 2005
Solomon SD, Anavekar N, Skali H, McMurray JJ, Swedberg K, Yusuf S, Granger CB, Michelson EL, Wang D, Pocock S, Pfeffer MA; Candesartan in Heart Failure Reduction in Mortality (CHARM) Investigators. Influence of ejection fraction on cardiovascular outcomes in a broad spectrum of heart failure patients. Circulation 2005 Dec 13;112(24):3738-44

Spertus et al 2005
Spertus J Am Heart J 2005. Monitoring clinical changes in patients with heart failure: A comparison of methods. Multicenter Study

Thygesen et al 2012
Thygesen Kristian, Alpert Joseph S., Jaffe Allan S., Simoons Maarten L, Chaitman ,White Harvey D. Writing Group on behalf of the Joint ESC/ACCF/AHA/WHF Task Force for the Universal

Definition of Myocardial Infarction. Third Universal Definition of Myocardial Infarction. Global Heart Vol: 7 (4), 2012 Page: 275-295

Wanner et al 2016
Wanner Christoph; Inzucchi, Silvio E.; Lachin, John M; Fitchett, David; von Eynatten, Maximilian. Cardio metabolic Risk Factor Changes Observed in Diabetes Prevention Programs in US Settings: A Systematic Review and Meta-analysis. New England Journal of Medicine Vol: 375 (4) 2016 Page: 323 - 334

Zannad et al 2014
Zannad F Garcia AA, Anker SD Armstrong PW Calvo G et al. Clinical outcome endpoints in heart failure trials: a European Society of Cardiology Heart Failure Association consensus document. Eur J Heart Failure 2013;15:1082–94.

Zinman et al 2015
Zinman B, Wanner C, Lachin JM, Fitchett D, Bluhmki E et al. Empagliflozin, cardiovascular outcomes, and mortality in type 2 diabetes. N Engl J Med 2015; 373:2117-2128.

11. SUPPORTING DOCUMENTATION AND OPERATIONAL CONSIDERATIONS

Appendix A Regulatory, ethical and study oversight considerations

A 1 Regulatory and ethical considerations

This study will be conducted in accordance with the protocol and with the following:

- Consensus ethical principles derived from international guidelines including the Declaration of Helsinki and Council for International Organizations of Medical Sciences (CIOMS) International Ethical Guidelines
- Applicable ICH Good Clinical Practice (GCP) Guidelines
- Applicable laws and regulations

The protocol, protocol amendments, ICF, Investigator Brochure, and other relevant documents (e.g. advertisements) must be submitted to an IRB/IEC by the investigator and reviewed and approved by the IRB/IEC before the study is initiated.

Any amendments to the protocol will require IRB/IEC approval before implementation of changes made to the study design, except for changes necessary to eliminate an immediate hazard to study subjects.

The investigator will be responsible for the following:

- Providing written summaries of the status of the study to the IRB/IEC annually or more frequently in accordance with the requirements, policies, and procedures established by the IRB/IEC
- Notifying the IRB/IEC of SAEs or other significant safety findings as required by IRB/IEC procedures
- Providing oversight of the conduct of the study at the site and adherence to requirements of 21 CFR, ICH guidelines, the IRB/IEC, European regulation 536/2014 for clinical studies (if applicable), and all other applicable local regulations

The study will be performed in accordance with the AstraZeneca policy on Bioethics and Human Biological Samples.

A 2 Financial disclosure

Investigators and sub-investigators will provide the sponsor with sufficient, accurate financial information as requested to allow the sponsor to submit complete and accurate financial certification or disclosure statements to the appropriate regulatory authorities. Investigators are responsible for providing information on financial interests during the course of the study and for 1 year after completion of the study.

A 3 Informed consent process

The Investigator or his/her representative will explain the nature of the study to the subject or his/her legally authorised representative and answer all questions regarding the study.

Subjects must be informed that their participation is voluntary. Subjects or their legally authorised representative will be required to sign a statement of informed consent that meets the requirements of 21 CFR 50, local regulations, ICH guidelines, Health Insurance Portability and Accountability Act (HIPAA) requirements, where applicable, and the IRB/IEC or study centre.

The medical record must include a statement that written informed consent was obtained before the subject was enrolled in the study and the date the written consent was obtained. The authorised person obtaining the informed consent must also sign the ICF.
Subjects must be re-consented to the most current version of the ICF(s) during their participation in the study.

A copy of the ICF(s) must be provided to the subject or the subject's legally authorised representative.

A subject who is rescreened is not required to sign another ICF.

A 4 Data protection

Each subject will be assigned a unique identifier by the sponsor. Any subject records or data sets transferred to the sponsor will contain only the identifier; subject names or any information which would make the subject identifiable will not be transferred.

The subject must be informed that his/her personal study-related data will be used by the sponsor in accordance with local data protection law. The level of disclosure must also be explained to the subject.

The subject must be informed that his/her medical records may be examined by Clinical Quality Assurance auditors or other authorised personnel appointed by the sponsor, by appropriate IRB/IEC members, and by inspectors from regulatory authorities.

A 5 Committees structure

Executive Committee

Together with AZ, the Executive Committee will be responsible for the final overall study design, including the development of the study protocol and eCRF, supervision of the study conduct and progress, development of any protocol amendments needed during the study, liaison with the CEA committee and DMC and DKA committee as needed, development of the statistical analysis plan, interpretation of the final data and reporting (presentations at international congresses and publications in peer reviewed journals) of the study.
The Executive Committee will make recommendations to AstraZeneca with regards to early stopping or modifications of the study based on the information received from the DMC. The

Executive Committee will be comprised of designated international academic leaders and nonvoting members of the Sponsor, and will operate under an Executive Committee charter.

National Lead Investigator (NLI) Committee

The National Lead Investigator (NLI) Committee will be comprised of NLIs from each country where the study is conducted and supervised by the Executive Committee. Members of the committee will be responsible for providing clinical guidance on study implementation, recruitment and study conduct in their respective country.

Data Monitoring Committee (DMC)

An independent DMC will be appointed and will report to the Executive Committee. The DMC will be responsible for safeguarding the interests of the patients in the outcome study by assessing the safety of the intervention during the study, and for reviewing the overall conduct of the study. The DMC will have access to the individual treatment codes and be able to merge these with the collected study data while the study is ongoing. A DMC charter will be prepared to detail precise roles and responsibilities and procedures to ensure maintenance of the blinding and integrity of the study in the review of accumulating data and interactions with the Executive Committee.

Clinical Event Adjudication (CEA) Committee

The role of the CEA committee is to independently review, interpret and adjudicate potential endpoints that are experienced by the patients. Endpoints will be identified preliminary by the investigators, and also by AZ personnel or in the CEA process as specified in the CEA charter. The CEA committee members will not have access to individual treatment codes for any patient or clinical efficacy endpoint and safety event. The precise responsibilities and procedures applicable for CEA will be detailed in the CEA charter.

A 6 Dissemination of clinical study data

A description of this clinical trial will be available on http://astrazenecaclinicaltrials.com and http://www.clinicaltrials.gov as will the summary of the main study results when they are available. The clinical trial and/or summary of main study results may also be available on other websites according to the regulations of the countries in which the main study is conducted.

A 7 Data quality assurance

All subject data relating to the study will be recorded on printed or electronic CRF unless transmitted to the sponsor or designee electronically (e.g. laboratory data). The Investigator is responsible for verifying that data entries are accurate and correct by physically or electronically signing the CRF.

The Investigator must maintain accurate documentation (source data) that supports the information entered in the CRF.

The Investigator must permit study-related monitoring, audits, IRB/IEC review, and regulatory agency inspections and provide direct access to source data documents.

The sponsor or designee is responsible for the data management of this study including quality checking of the data.

Study monitors will perform ongoing source data verification to confirm that data entered into the CRF by authorised site personnel are accurate, complete, and verifiable from source documents; that the safety and rights of subjects are being protected; and that the study is being conducted in accordance with the currently approved protocol and any other study agreements, ICH GCP, and all applicable regulatory requirements.

Records and documents, including signed ICFs, pertaining to the conduct of this study must be retained by the Investigator for 15 years after study completion unless local regulations or institutional policies require a longer retention period. No records may be destroyed during the retention period without the written approval of the sponsor. No records may be transferred to another location or party without written notification to the sponsor.

A 8 Source documents

Source documents provide evidence for the existence of the subject and substantiate the integrity of the data collected. Source documents are filed at the Investigator's site.

Data reported on the CRF that are transcribed from source documents must be consistent with the source documents or the discrepancies must be explained. The Investigator may need to request previous medical records or transfer records, depending on the study. Also, current medical records must be available.

A 9 Publication policy

The results of this study may be published or presented at scientific meetings. If this is foreseen, the investigator agrees to submit all manuscripts or abstracts to the sponsor before submission. This allows the sponsor to protect proprietary information and to provide comments.

The sponsor will comply with the requirements for publication of study results. In accordance with standard editorial and ethical practice, the sponsor will generally support publication of multicentre studies only in their entirety and not as individual site data. In this case, a coordinating investigator will be designated by mutual agreement.

Authorship will be determined by mutual agreement and in line with International Committee of Medical Journal Editors authorship requirements.

Appendix B Adverse event definitions and additional safety information

B 1 Definition of adverse events

An adverse event is the development of any untoward medical occurrence in a subject or clinical study subject administered a medicinal product and which does not necessarily have a causal relationship with this treatment. An AE can therefore be any unfavourable and unintended sign (e.g. an abnormal laboratory finding), symptom (for example nausea, chest pain), or disease temporally associated with the use of a medicinal product, whether or not considered related to the medicinal product.

The term AE is used to include both serious and non-serious AEs and can include a deterioration of a pre-existing medical occurrence. An AE may occur at any time, including run-in or washout periods, even if no Study treatment has been administered.

B 2 Definitions of serious adverse event

A serious adverse event is an AE occurring during any study phase (i.e., run-in, treatment, washout, follow-up), that fulfils one or more of the following criteria:

- Results in death
- Is immediately life-threatening
- Requires in-subject hospitalisation or prolongation of existing hospitalisation
- Results in persistent or significant disability or incapacity
- Is a congenital abnormality or birth defect
- Is an important medical event that may jeopardise the subject or may require medical treatment to prevent one of the outcomes listed above.

B 3 Life threatening

'Life-threatening' means that the subject was at immediate risk of death from the AE as it occurred or it is suspected that use or continued use of the product would result in the subject's death. 'Life-threatening' does not mean that had an AE occurred in a more severe form it might have caused death (e.g., hepatitis that resolved without hepatic failure).

B 4 Hospitalisation

Outpatient treatment in an emergency room is not in itself a serious AE, although the reasons for it may be (e.g., bronchospasm, laryngeal oedema). Hospital admissions and/or surgical operations planned before or during a study are not considered AEs if the illness or disease existed before the subject was enrolled in the study, provided that it did not deteriorate in an unexpected way during the study.

B 5 Important medical event or medical treatment

Medical and scientific judgement should be exercised in deciding whether a case is serious in situations where important medical events may not be immediately life threatening or result in death, hospitalisation, disability or incapacity but may jeopardize the subject or may require medical treatment to prevent one or more outcomes listed in the definition of serious. These should usually be considered as serious.

Simply stopping the suspect drug does not mean that it is an important medical event; medical judgement must be used.

- Angioedema not severe enough to require intubation but requiring iv hydrocortisone treatment
- Hepatotoxicity caused by paracetamol (acetaminophen) overdose requiring treatment with N-acetylcysteine
- Intensive treatment in an emergency room or at home for allergic bronchospasm
- Blood dyscrasias (e.g. neutropenia or anaemia requiring blood transfusion, etc.) or convulsions that do not result in hospitalisation
- Development of drug dependency or drug abuse

B 6 Intensity rating scale:

1. mild (awareness of sign or symptom, but easily tolerated)
2. moderate (discomfort sufficient to cause interference with normal activities)
3. severe (incapacitating, with inability to perform normal activities)

It is important to distinguish between serious and severe AEs. Severity is a measure of intensity whereas seriousness is defined by the criteria in Appendix B 2. An AE of severe intensity need not necessarily be considered serious. For example, nausea that persists for several hours may be considered severe nausea, but not a SAE unless it meets the criteria shown in Appendix B 2. On the other hand, a stroke that results in only a limited degree of disability may be considered a mild stroke but would be a SAE when it satisfies the criteria shown in Appendix B 2.

B 7 A Guide to Interpreting the Causality Question

When assessing causality consider the following factors when deciding if there is a 'reasonable possibility' that an AE may have been caused by the drug.

- Time Course. Exposure to suspect drug. Has the subject actually received the suspect drug? Did the AE occur in a reasonable temporal relationship to the administration of the suspect drug?

- Consistency with known drug profile. Was the AE consistent with the previous knowledge of the suspect drug (pharmacology and toxicology) or drugs of the same pharmacological class? Or could the AE be anticipated from its pharmacological properties?

- De-challenge experience. Did the AE resolve or improve on stopping or reducing the dose of the suspect drug?

- No alternative cause. The AE cannot be reasonably explained by another aetiology such as the underlying disease, other drugs, other host or environmental factors.

- Re-challenge experience. Did the AE reoccur if the suspected drug was reintroduced after having been stopped? AstraZeneca would not normally recommend or support a re-challenge.

- Laboratory tests. A specific laboratory investigation (if performed) has confirmed the relationship.

In difficult cases, other factors could be considered such as:

- Is this a recognized feature of overdose of the drug?

- Is there a known mechanism?

Causality of ‘related’ is made if following a review of the relevant data, there is evidence for a ‘reasonable possibility’ of a causal relationship for the individual case. The expression ‘reasonable possibility’ of a causal relationship is meant to convey, in general, that there are facts (evidence) or arguments to suggest a causal relationship.

The causality assessment is performed based on the available data including enough information to make an informed judgment. With limited or insufficient information in the case, it is likely that the event(s) will be assessed as ‘not related’.

Causal relationship in cases where the disease under study has deteriorated due to lack of effect should be classified as no reasonable possibility.

B 8 Medication Error

For the purposes of this clinical study a medication error is an unintended failure or mistake in the treatment process for an AstraZeneca investigational product that either causes harm to the participant or has the potential to cause harm to the participant.

A medication error is not lack of efficacy of the drug, but rather a human or process related failure while the drug is in control of the study site staff or participant.

Medication error includes situations where an error:

- Occurred
- Was identified and intercepted before the participant received the drug
- Did not occur, but circumstances were recognized that could have led to an error

Examples of events to be reported in clinical studies as medication errors:

- Drug name confusion
- Dispensing error e.g. medication prepared incorrectly, even if it was not actually given to the participant
- Drug not administered as indicated, for example, wrong route or wrong site of administration
- Drug not taken as indicated e.g. tablet dissolved in water when it should be taken as a solid tablet
- Drug not stored as instructed e.g. kept in the fridge when it should be at room temperature
- Wrong participant received the medication (excluding IxRS errors)
- Wrong drug administered to participant (excluding IxRS errors)

Examples of events that **do not** require reporting as medication errors in clinical studies:

- Errors related to or resulting from IxRS - including those which lead to one of the above listed events that would otherwise have been a medication error
- Participant accidentally missed drug dose(s) e.g. forgot to take medication
- Accidental overdose (will be captured as an overdose)
- Participant failed to return unused medication or empty packaging
- Errors related to background and rescue medication, or standard of care medication in open label studies, even if an AZ product

Medication errors are not regarded as AEs but AEs may occur as a consequence of the medication error.

Appendix C Cardiovascular related events

C 1 Myocardial Infractions (MI)

MIs are not endpoints in this study but unstable angina and myocardial infarction should be recorded as SAEs if serious criteria are met and additional information be collected in specific eCRF. The diagnoses of unstable angina and MI should adhere to the standardised definitions for endpoints (Hicks et al 2018) described in Appendix C 2

C 2 Diagnosis of MI and Unstable Angina

Myocardial infarction (MI)

The diagnosis of an MI should be made according to standard clinical practice but is expected to align with the criteria from Third Universal Definition of MI, i.e. detection of a rise and/or fall of cardiac biomarkers such as troponin and at least one of the following: typical clinical symptoms, ischaemic ECG findings, imaging evidence of myocardial injury, or detection of an intracoronary thrombus by angiography or autopsy (Thygesen et al 2012).

The diagnosis should be made by, or in consultation with, a cardiologist. The findings supporting the diagnosis should be documented in the description of the SAE in the eCRF.

Unstable Angina (UA)

Unstable Angina (UA) is not an endpoint in this study but should be recorded as SAEs (and DAEs when appropriate). The diagnosis of an UA should be made according to standard clinical practice but is expected to align with the following definition:

The diagnosis of unstable angina will require ischemic chest pain (or equivalent) at rest ≥10 minutes in duration considered to be myocardial ischemia upon final diagnosis and prompting hospitalisation within 24 hours of the most recent symptoms, and without elevation in cardiac biomarkers of necrosis, and the presence of objective evidence of ischemia as defined by at least 1 of the following criteria:

1. New or worsening ST or T wave changes in ≥2 anatomically contiguous leads on a resting ECG (in the absence of LVH and LBBB):

a) transient (<20 minutes) ST elevation at the J point ≥ 0.2 mV in men (> 0.25 mV in men < 40 years old) or ≥ 0.15 mV in women in leads V2-V3 and/or ≥ 0.1 mV in other leads, or

b) horizontal or down-sloping ST depression ≥ 0.10 mV, or

c) T-wave inversion ≥ 0.2 mV

2. Definite evidence of myocardial ischemia on myocardial scintigraphy (clear reversible perfusion defect), stress echocardiography (reversible wall motion abnormality), or MRI (myocardial perfusion deficit under pharmacologic stress) that is believed to be responsible for the myocardial ischemic symptoms/signs.

3. Angiographic evidence of ≥ 70% lesion and/or thrombus in an epicardial coronary artery that is believed to be responsible for the myocardial ischemic symptoms/signs.

C 3 Stroke

Stroke is not an endpoint in this study but should be recorded as SAEs if serious criteria are met, with additional information e.g. classification of stroke type (ischaemic, haemorrhagic, or undetermined) collected in a specific eCRF.

The diagnosis of stroke should be made according to standard clinical practice and align with the definition for stroke in the standardized definitions for endpoints (Hicks et al. 2018 Hicks KA, Mahaffey KW, Mehran R, Nissen SE, et alCardiovascular and Stroke Endpoint Definitions for Clinical Trials. J Am Coll Cardiol 2018;71:1021–34) described in Appendix C 4 and be differentiated vs Transient Ischaemic Attack (TIA).

C 4 Definition of Stroke and Transient Ischemic Attack

The distinction between an Ischemic Stroke and a Transient Ischemic Attack is the presence of infarction. Persistence of symptoms ≥24 hours or until death[3] is an acceptable indicator of acute infarction in the absence of imaging evidence of infarction.

Transient Ischemic Attack
Transient ischemic attack (TIA) is defined as a transient episode of focal neurological dysfunction caused by brain, spinal cord, or retinal ischemia, *without* acute infarction.

Stroke
Stroke is defined as an acute episode of focal or global neurological dysfunction caused by brain, spinal cord, or retinal vascular injury as a result of haemorrhage or infarction.

Classification:

A. Ischemic Stroke

Ischemic stroke is defined as an acute episode of focal cerebral, spinal, or retinal dysfunction caused by infarction of central nervous system tissue.
Haemorrhage may be a consequence of ischemic stroke. In this situation, the stroke is an ischemic stroke with haemorrhagic transformation and not a haemorrhagic stroke.

B. Haemorrhagic Stroke

Haemorrhagic stroke is defined as an acute episode of focal or global cerebral or spinal dysfunction caused by non-traumatic intraparenchymal, intraventricular, or subarachnoid haemorrhage. NOTE: Subdural hematomas are intracranial haemorrhagic events and not strokes.

C. Undetermined Stroke

Undetermined stroke is defined as an acute episode of focal or global neurological dysfunction caused by presumed brain, spinal cord, or retinal vascular injury as a result of haemorrhage or infarction but with insufficient information to allow categorization as either ischemic or haemorrhagic.

References:

Hicks KA et al. 2017 Cardiovascular and Stroke Endpoint Definitions for Clinical Trials. J Am Coll Cardiol 2018;71:1021–34 https://doi.org/10.1016/j.jacc.2017.12.048

Draft Definitions for CDISC August 20, 2014

Sacco RL, Kasner SE, Broderick JP, Caplan LR, Connors JJ, Culebras A, Elkind MSV, George MG, Hamdan AD, Higashida RT, Hoh BL, Janis LS, Kase CS, Kleindorfer DO, Lee J-M, Moseley ME, Peterson ED, Turan TN, Valderrama AL, Vinters HV; on behalf of the American Heart Association Stroke Council, Council on Cardiovascular Surgery and Anesthesia, Council on Cardiovascular Radiology and Intervention, Council on Cardiovascular and Stroke Nursing, Council on Epidemiology and Prevention, Council on Peripheral Vascular Disease, and Council on Nutrition, Physical Activity and Metabolism. An updated definition of stroke for the 21st century: a statement for healthcare professionals from the American Heart Association/American Stroke Association. Stroke. 2013;44:2064-2089.

Appendix D Genetics

D 1 Use/analysis of DNA

Genetic variation may impact a subject's response to therapy, susceptibility to, and severity and progression of disease. Variable response to therapy may be due to genetic determinants that impact drug absorption, distribution, metabolism, and excretion; mechanism of action of the drug; disease aetiology; and/or molecular subtype of the disease being treated. Therefore, where local regulations and IRB/IEC allow, a blood sample will be collected for DNA analysis from consenting subjects.

AstraZeneca intends to collect and store DNA for genetic research to explore how genetic variations may affect clinical parameters, risk and prognosis of diseases, and the response to medications. Genetic research may lead to better understanding of diseases, better diagnosis of diseases or other improvements in health care and to the discovery of new diagnostics, treatments or medications.

In addition, collection of DNA samples from populations with well described clinical characteristics may lead to improvements in the design and interpretation of clinical trials and, possibly, to genetically guided treatment strategies.

Genetic research may consist of the analysis of the structure of the subject´s DNA, i.e. the entire genome.

The results of genetic analyses may be reported in the clinical study report (CSR) or in a separate study summary.

The sponsor will store the DNA samples in a secure storage space with adequate measures to protect confidentiality.

The samples will be retained while research on heart failure with preserved ejection fraction continues but no longer than 15 years or other period as per local requirements.

D 2 Genetic research plan and procedures

Selection of genetic research population

Study selection record

All subjects will be asked to participate in this genetic research. Participation is voluntary and if a subject decline to participate there will be no penalty or loss of benefit. The subject will not be excluded from any aspect of the main study.

Inclusion criteria

- For inclusion in this genetic research, subjects must fulfil all of the inclusion criteria described in the main body of the Clinical Study Protocol **and** Provide informed consent for the genetic sampling and analyses.

Exclusion criteria

Exclusion from this genetic research may be for any of the exclusion criteria specified in the main study or any of the following:

- Previous allogeneic bone marrow transplant
- Non-leukocyte depleted whole blood transfusion in 120 days of genetic sample collection

Withdrawal of consent for genetic research:
Subjects may withdraw from this genetic research at any time, independent of any decision concerning participation in other aspects of the main study. Voluntary withdrawal will not prejudice further treatment. Procedures for withdrawal are outlined in Section **Error! Reference source not found.** of the main Clinical Study Protocol.

Collection of samples for genetic research
The blood sample for genetic research will be obtained from the subjects at Visit 2. Although DNA is stable, early sample collection is preferred to avoid introducing bias through excluding subjects who may withdraw due to an adverse event (AE), such subjects would be important to include in any genetic analysis. If for any reason the sample is not drawn at Visit 2, it may be taken at any visit until the last study visit. Only one sample should be collected per subject for genetics during the study. Samples will be collected, labelled, stored, and shipped as detailed in the Laboratory Manual.

Coding and storage of DNA samples
The processes adopted for the coding and storage of samples for genetic analysis are important to maintain subject confidentiality. Samples will be stored for a maximum of 15 years, from the date of last subject last visit, after which they will be destroyed. DNA is a finite resource that is used up during analyses. Samples will be stored and used until no further analyses are possible or the maximum storage time has been reached.

An additional second code will be assigned to the blood sample either before or at the time of DNA extraction replacing the information on the sample tube. Thereafter, the sample will be identifiable only by the second, unique number. This number is used to identify the sample and corresponding data at the AstraZeneca genetics laboratories, or at the designated organisation. No personal details identifying the individual will be available to any person (AstraZeneca employee or designated organisations working with the DNA).

The link between the subject enrolment/randomisation code and the second number will be maintained and stored in a secure environment, with restricted access at AstraZeneca or designated organisations. The link will be used to identify the relevant DNA samples for analysis, facilitate correlation of genotypic results with clinical data, allow regulatory audit, and permit tracing of samples for destruction in the case of withdrawal of consent.

Ethical and regulatory requirements
The principles for ethical and regulatory requirements for the study, including this genetics research component, are outlined in Appendix A.

Informed consent

The genetic component of this study is optional and the subject may participate in other components of the main study without participating in the genetic component. To participate in the genetic component of the study the subject must sign and date both the consent form for the main study and the genetic component of the study. Copies of both signed and dated consent forms must be given to the subject and the original filed at the study centre. The Principal Investigator(s) is responsible for ensuring that consent is given freely and that the subject understands that they may freely withdrawal from the genetic aspect of the study at any time.

Subject data protection

AstraZeneca will not provide individual genotype results to subjects, any insurance company, any employer, their family members, general physician unless required to do so by law.

Extra precautions are taken to preserve confidentiality and prevent genetic data being linked to the identity of the subject. In exceptional circumstances, however, certain individuals might see both the genetic data and the personal identifiers of a subject. For example, in the case of a medical emergency, an AstraZeneca Physician or an investigator might know a subject's identity and also have access to his or her genetic data. In addition, Regulatory authorities may require access to the relevant files, though the subject's medical information and the genetic files would remain physically separate.

Data management

Any genotype data generated in this study will be stored at a secure system at AstraZeneca and/or designated organizations to analyse the samples.

AstraZeneca and its designated organisations may share summary results (such as genetic differences from groups of individuals with a disease) from this genetic research with other researchers, such as hospitals, academic organisations or health insurance companies. This can be done by placing the results in scientific databases, where they can be combined with the results of similar studies to learn even more about health and disease. The researchers can only use this information for health-related research purposes. Researchers may see summary results but they will not be able to see individual subject data or any personal identifiers.

Some or all of the clinical datasets from the main study may be merged with the genetic data in a suitable secure environment separate from the clinical database.

Statistical methods and determination of sample size

The number of subjects that will agree to participate in the genetic research is unknown. It is therefore not possible to establish whether sufficient data will be collected to allow a formal

statistical evaluation or whether only descriptive statistics will be generated. A Statistical Analysis Plan may be prepared where appropriate.

Appendix E Handling of Human Biological Samples

E 1 Chain of custody of biological samples

A full chain of custody is maintained for all samples throughout their lifecycle.

The Investigator at each centre keeps full traceability of collected biological samples from the subjects while in storage at the centre until shipment or disposal (where appropriate).

The sample receiver keeps full traceability of the samples while in storage and during use until used or disposed of or until further shipment and keeps documentation of receipt of arrival.

AstraZeneca will keep oversight of the entire life cycle through internal procedures, monitoring of study sites, auditing or process checks, and contractual requirements of external laboratory providers

Samples retained for further use will be stored in the AZ-assigned biobanks and will be registered by the AstraZeneca Biobank Team during the entire life cycle.

If required, AstraZeneca will ensure that remaining biological samples are returned to the site according to local regulations or at the end of the retention period, whichever is the sooner.

E 2 Withdrawal of Informed Consent for donated biological samples

If a subject withdraws consent to the use of donated biological samples, the samples will be disposed of/destroyed, and the action documented. If samples are already analysed, AstraZeneca is not obliged to destroy the results of this research.

As collection of the biological sample(s) is an integral part of the study, then the subject is withdrawn from further study participation.

The Investigator:

- Ensures subjects' withdrawal of informed consent to the use of donated samples is notified immediately to AstraZeneca
- Ensures that biological samples from that subject, if stored at the study site, are immediately identified, disposed of /destroyed, and the action documented
- Ensures the organization(s) holding the samples is/are informed about the withdrawn consent immediately and that samples are disposed of/destroyed, the action documented and the signed document returned to the study site
- Ensures that the subject and AstraZeneca are informed about the sample disposal.

AstraZeneca ensures the organizations holding the samples is/are informed about the withdrawn consent immediately and that samples are disposed of/destroyed and the action documented and returned to the study site.

E 3 International Airline Transportation Association (IATA) 6.2 Guidance Document

LABELLING AND SHIPMENT OF BIOHAZARD SAMPLES

International Airline Transportation Association (IATA) classifies biohazardous agents into 3 categories (http://www.iata.org/whatwedo/cargo/dangerous_goods/infectious_substances.htm). For transport purposes the classification of infectious substances according to risk groups was removed from the Dangerous Goods Regulations in the 46th edition (2005). Infectious substances are now classified either as Category A, Category B or Exempt. There is no direct relationship between Risk Groups and Categories A and B.

Category A Infectious Substances are infectious substances in a form that, when exposure to it occurs, is capable of causing permanent disability, life-threatening or fatal disease in otherwise healthy humans or animals. Category A pathogens are e.g., Ebola, Lassa fever virus:

- Are to be packed and shipped in accordance with IATA Instruction 602.

Category B Infectious Substances are infectious Substances that do not meet the criteria for inclusion in Category A. Category B pathogens are e.g., Hepatitis A, B, C, D, and E viruses, Human immunodeficiency virus types 1 and 2. They are assigned the following UN number and proper shipping name:

- UN 3373 – Biological Substance, Category B
- Are to be packed in accordance with UN3373 and IATA 650

Exempt - all other materials with minimal risk of containing pathogens

- Clinical trial samples will fall into Category B or exempt under IATA regulations
- Clinical trial samples will routinely be packed and transported at ambient
- Temperature in IATA 650 compliant packaging (http://www.iata.org/whatwedo/cargo/dangerous_goods/infectious_substances.htm)
- **Biological samples transported in dry ice require additional dangerous goods specification for the dry-ice content**
- IATA compliant courier and packaging materials should be used for packing and transportation and packing should be done by an IATA certified person, as applicable
- Samples routinely transported by road or rail are subject to local regulations which require that they are also packed and transported in a safe and appropriate way to contain any risk of infection or contamination by using approved couriers and packaging/containment materials at all times. The IATA 650 biological sample containment standards are encouraged wherever possible when road or rail transport is used.

Appendix G Actions required in cases of increases in liver biochemistry and evaluation of Hy's Law (not applicable)

Appendix H Medical device incidents: definition and procedures for recording, evaluating, follow-up, and reporting (not applicable)

Appendix I Abbreviations

Abbreviation or special term	Explanation
AE	Adverse Event
BP	Blood Pressure
CEA	Clinical Event Adjudication
COPD	Chronic Obstructive Pulmonary Disease
CSA	Clinical study Agreement
CV	Cardiovascular
DAE	Adverse Event leading to discontinuation of investigational product
DKA	Diabetic Ketoacidosis
DMC	Data Monitoring Committee
eCRF	Electronic Case Report Form
EC	Ethics Committee, synonymous to Institutional Review Board (IRB) and Independent Ethics Committee (IEC)
ECG	Electrocardiogram
EDC	Electronic Data Capture
EHRs	Electronic Health Records
FAS	Full Analysis Set
GCP	Good Clinical Practice
HF	Heart Failure
HFpEF	Heart Failure with Preserved Ejection Fraction
HR	Hazard Ratio
ICF	Informed Consent Form
ICH	International Conference on Harmonisation
International Co-ordinating Investigator	If a study is conducted in several countries the International Co-ordinating Investigator is the Investigator co-ordinating the Investigators and/or activities internationally.
IxRS	Interactive Voice/Web Response System
KCCQ	Kansas City Cardiomyopathy Questionnaire
LAE	Left Atrial Enlargement
LSLV	Last Subject Last Visit
LVEF	Left Ventricular Ejection Fraction
MI	Myocardial Infarction
NYHA	New York Heart Association

Abbreviation or special term	Explanation
PACD	Primary Analysis Censoring Date
PTDV	Premature Treatment Discontinuation Visit
PI	Principal Investigator
SAE	Serious Adverse Event
SAP	Statistical Analysis Plan
SCV	Study Closure Visit
SoA	Schedule of Activities
T2D	Type 2 Diabetes Mellitus

Appendix J New York Heart Association (NYHA) Functional Classification

Class	Patient symptoms
I	No limitation of physical activity. Ordinary physical activity does not cause undue fatigue, palpitation, dyspnoea (shortness of breath).
II	Slight limitation of physical activity. Comfortable at rest. Ordinary physical activity results in fatigue, palpitation, dyspnea (shortness of breath).
III	Marked limitation of physical activity. Comfortable at rest. Less than ordinary activity causes fatigue, palpitation, or dyspnea.
IV	Unable to carry on any physical activity without discomfort. Symptoms of heart failure at rest. If any physical activity is undertaken, discomfort increases.

Appendix K The KC Cardiomyopathy Questionnaire

The KC Cardiomyopathy Questionnaire

The following questions refer to your heart failure and how it may affect your life. Please read and complete the following questions. There are no right or wrong answers. Please mark the answer that best applies to you.

1. **Heart failure** affects different people in different ways. Some feel shortness of breath while others feel fatigue. Please indicate how much you are limited by **heart failure** (shortness of breath or fatigue) in your ability to do the following activities over the past 2 weeks.

Place an **X** in one box on each line

Activity	Extremely Limited	Quite a bit Limited	Moderately Limited	Slightly Limited	Not at all Limited	Limited for other reasons or did not do the activity
Dressing yourself	❑	❑	❑	❑	❑	❑
Showering/Bathing	❑	❑	❑	❑	❑	❑
Walking 1 block on level ground	❑	❑	❑	❑	❑	❑
Doing yardwork, housework or carrying groceries	❑	❑	❑	❑	❑	❑
Climbing a flight of stairs without stopping	❑	❑	❑	❑	❑	❑
Hurrying or jogging (as if to catch a bus)	❑	❑	❑	❑	❑	❑

2. <u>Compared with 2 weeks ago</u>, have your symptoms of **heart failure** (shortness of breath, fatigue, or ankle swelling) changed?

My symptoms of **heart failure** have become…

**Much worse**	**Slightly worse**	**Not changed**	**Slightly better**	**Much better**	**I've had no symptoms over the last 2 weeks**
❑	❑	❑	❑	❑	❑

3. Over the <u>past 2 weeks</u>, how many times did you have **swelling** in your feet, ankles or legs when you woke up in the morning?

**Every morning**	**3 or more times a week, but not every day**	**1-2 times a week**	**Less than once a week**	**Never over the past 2 weeks**
❑	❑	❑	❑	❑

4. Over the <u>past 2 weeks</u>, how much has **swelling** in your feet, ankles or legs bothered you?

It has been ...

Extremely **bothersome**	Quite a bit **bothersome**	Moderately **bothersome**	Slightly **bothersome**	Not at all **bothersome**	I've had **no swelling**
❑	❑	❑	❑	❑	❑

5. Over <u>the past 2 weeks</u>, on average, how many times has **fatigue** limited your ability to do what you want?

All of the time	Several times per day	At least once a day	3 or more times per week but not every day	1-2 times per week	Less than once a week	**Never over the past 2 weeks**
❑	❑	❑	❑	❑	❑	❑

6. Over the past 2 weeks, how much has your **fatigue** bothered you?

It has been ...

Extremely **bothersome**	Quite a bit **bothersome**	Moderately **bothersome**	Slightly **bothersome**	Not at all **bothersome**	I've had **no fatigue**
❑	❑	❑	❑	❑	❑

7. Over the past 2 weeks, on average, how many times has **shortness of breath** limited your ability to do what you wanted?

**All of the time**	**Several times per day**	**At least once a day**	**3 or more times per week but not every day**	**1-2 times per week**	**Less than once a week**	Never over the past 2 weeks
❑	❑	❑	❑	❑	❑	❑

8. Over the past 2 weeks, how much has your **shortness of breath** bothered you?

It has been ...

Extremely **bothersome**	Quite a bit **bothersome**	Moderately **bothersome**	Slightly **bothersome**	Not at all **bothersome**	I've had **no shortness of breath**
❑	❑	❑	❑	❑	❑

9. Over the past 2 weeks, on average, how many times have you been forced to sleep sitting up in a chair or with at least 3 pillows to prop you up because of **shortness of breath**?

**Every night**	**3 or more times per week, but not every day**	**1-2 times a week**	**Less than once a week**	Never over the past 2 weeks
❑	❑	❑	❑	❑

10. Heart failure symptoms can worsen for a number of reasons. How sure are you that you know what to do, or whom to call, if your **heart failure** gets worse?

Not at all **sure**	**Not very** sure	Somewhat **sure**	**Mostly** sure	**Completely** sure
❑	❑	❑	❑	❑

11. How well do you understand what things you are able to do to keep your **heart failure** symptoms from getting worse? (for example, weighing yourself, eating a low salt diet etc.)

**Do not understand at all**	Do not understand very well	**Somewhat understand**	Mostly understand	Completely understand
❑	❑	❑	❑	❑

12. Over the past 2 weeks, how much has your **heart failure** limited your enjoyment of life?

**It has** extremely **limited my enjoyment of life**	It has limited my enjoyment of life **quite a bit**	**It has** moderately **limited my enjoyment of life**	It has **slightly** limited my enjoyment of life	It has **not limited** my enjoyment of life at all
❑	❑	❑	❑	❑

13. If you had to spend the rest of your life with your **heart failure** the way it is right now, how would you feel about this?

**Not at all satisfied**	Mostly dissatisfied	**Somewhat satisfied**	Mostly satisfied	Completely satisfied
❑	❑	❑	❑	❑

14. Over the past 2 weeks, how often have you felt discouraged or down in the dumps because of your heart failure?

I felt that way **all of the time**	I felt that way **most of the time**	I **occasionally** felt that way	I **rarely** felt that way	I **never** felt that way
❑	❑	❑	❑	❑

15. How much does your heart failure affect your lifestyle? Please indicate how your heart failure may have limited your participation in the following activities over the past 2 weeks.

Please place an **X** in one box on each line

**Activity**	**Severely limited**	**Limited quite a bit**	**Moderately limited**	**Slightly limited**	**Did not limit at all**	**Does not apply or did not do for other reasons**
**Hobbies, recreational activities**	❑	❑	❑	❑	❑	❑
**Working or doing household chores**	❑	❑	❑	❑	❑	❑
**Visiting family or friends out of your home**	❑	❑	❑	❑	❑	❑
**Intimate relationships with loved ones**	❑	❑	❑	❑	❑	❑

Copyright © 1992 –2005 John Spertus, MD, MPH Original US English

Appendix L EQ-5D-5L Questionnaire

Health Questionnaire

Under each heading, please check the ONE box that best describes your health TODAY

MOBILITY

I have no problems walking ☐

I have slight problems walking ☐

I have moderate problems walking ☐

I have severe problems walking ☐

I am unable to walk ☐

SELF-CARE

I have no problems washing or dressing myself ☐

I have slight problems washing or dressing myself ☐

I have moderate problems washing or dressing myself ☐

I have severe problems washing or dressing myself ☐

I am unable to wash or dress myself ☐

USUAL ACTIVITIES *(e.g. work, study, housework, family or leisure activities)*

I have no problems doing my usual activities ❑

I have slight problems doing my usual activities ❑

I have moderate problems doing my usual activities ❑

I have severe problems doing my usual activities ❑

I am unable to do my usual activities ❑

PAIN / DISCOMFORT

I have no pain or discomfort ❑

I have slight pain or discomfort ❑

I have moderate pain or discomfort ❑

I have severe pain or discomfort ❑

I have extreme pain or discomfort ❑

ANXIETY / DEPRESSION

I am not anxious or depressed ❑

I am slightly anxious or depressed ❑

I am moderately anxious or depressed ❑

I am severely anxious or depressed ❑

I am extremely anxious or depressed ❑

The best health you can imagine

- We would like to know how good or bad your health is TODAY.
- This scale is numbered from 0 to 100.
- 100 means the <u>best</u> health you can imagine.
  0 means the <u>worst</u> health you can imagine.
- Mark an X on the scale to indicate how your health is TODAY.
- Now, please write the number you marked on the scale in the box below.

YOUR HEALTH TODAY = ☐

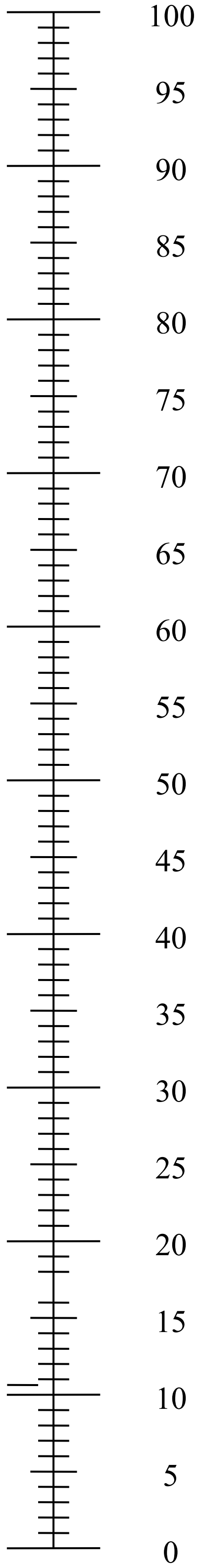

The worst health you can imagine

USA (English) © 2009 EuroQol Group. EQ-5D™ is a trade mark of the EuroQol Group

Appendix M Patient Global Impression of Severity for Heart Failure Symptoms

Patient Global Impression of Severity for Heart Failure Symptoms

Overall, how would you rate the severity of your heart failure symptoms today?

- ☐ No symptoms
- ☐ Very mild
- ☐ Mild
- ☐ Moderate
- ☐ Severe
- ☐ Very Severe

Clinical Study Protocol

Drug Substance	Dapagliflozin
Study Code	D169CC00001
Version	4.0
Date	12th November 2020

An International, Double-blind, Randomised, Placebo-Controlled Phase III Study to Evaluate the Effect of Dapagliflozin on Reducing CV Death or Worsening Heart Failure in Patients with Heart Failure with Preserved Ejection Fraction (HFpEF)

DELIVER - Dapagliflozin Evaluation to Improve the LIVEs of Patients with PReserved Ejection Fraction Heart Failure

Sponsor: AstraZeneca AB, 151 85 Södertälje, Sweden

Regulatory Agency Identifying Number(s):

Eudra CT number: 2018-000802-46

VERSION HISTORY

**Version 1.0, 24th April 2018**				
Initial creation				
**Version 2.0, 09th May 2018**				
	**Section changed**	**Previous Version**	**Current Version**	**Reason for change**
1.	Title page	**Regulatory Agency Identifying Number(s):**	**Regulatory Agency Identifying Number(s):** **Eudra CT number:** 2018-000802-46	Missing information added
2.	Appendix A – Section A3	A subject who is rescreened is **not** required to sign another ICF.	A subject who is rescreened is required to sign another ICF.	Correction of typo error
3.	Appendix D – Section D2 Correction of cross referencing error	**Withdrawal of consent for genetic research:** Subjects may withdraw from this genetic research at any time, independent of any decision concerning participation in other aspects of the main study. Voluntary withdrawal will not prejudice further treatment. Procedures for withdrawal are outlined in Section 7 of the main Clinical Study Protocol.	**Withdrawal of consent for genetic research:** Subjects may withdraw from this genetic research at any time, independent of any decision concerning participation in other aspects of the main study. Voluntary withdrawal will not prejudice further treatment. Procedures for withdrawal are outlined in Section 7 of the main Clinical Study Protocol.	Cross-reference link was updated
4.	Mislabelling of Appendices	Mislabelling of Appendices A, B, C, D, E, G, H, I, J, K, L, M	Correction of mislabelling of Appendices A, B, C, D, E, **F**, G, H, I, J, K, L	Typo error

Version 3.0, 16th December 2019

	Section changed	Summary of change
1.	1.2 Synopsis	CSP synopsis was modified to adjust the study sample size from original 4700 to approximately 6100, based on ongoing blinded monitoring of events accrual. Accordingly, the anticipated recruitment period was increased from 18 months to 22 months.
2.	4.1 Overall design	Overall design was modified to adjust the sample size increase as below:<br>"It is estimated that approximately 11000 patients at approximately 400-500 sites in 20-25 countries will be enrolled to reach approximately 6100 randomised patients."
3	4.2 Scientific rationale for study design	The subacute subgroup definition was modified, such that the recent discharge date from hospitalisation for heart failure was extended from 21 days to 30 days, to be more aligned with clinical practice, i.e.:<br>"…to address a specific need in a period with high risk for events, a proportion of patients will be enrolled and randomised during hospitalisation for heart failure or within 30 days of discharge from hospitalisation for heart failure (subacute subgroup)."
4.	6.3.1.2 Capping	The definition of subacute subgroup (one of the potential capping factors) was modified the same as above.
5.	9.2 Sample size determination	Statistical section 9.2 was updated to reflect the sample size increase in detail. Specifically:<br>"Based on the ongoing blinded monitoring of event accrual (including the percentage of patients from the sub-acute category), the sample size is increased from original 4700 to approximately 6100 randomised patients to provide the required number of 844 patients with a primary event. Accordingly, the recruitment period is anticipated to increase from the original 18 months to 22 months. The study is event driven and the number of patients or duration may further change."

j

Version 4.0, 12th November 2020		
	**Section changed**	**Summary of change**
1.	1.1: Schedule of Activities (SoA)	Concomitant Medication check was added to Visit 1 (Enrolment) to clarify screening eligibility checks.<br>Clarification to BP, pulse, weight and creatinine assessments at Visit 6 and 7 - onwards added: “Assessments to be repeated every 12 months (Visit 6, Visit 9, Visit 12)”.<br>Recording of COVID-19 testing results from Visit 2 onwards added to Safety Events.
2.	1.2 Synopsis	Primary objective and first secondary objective were updated to include analysis of both the full study population and the subpopulation with LVEF <60%.<br>Urgent HF visits were added in addition to hospitalizations for HF as recurrent HF events to be evaluated for the first secondary objective.<br>“To determine whether dapagliflozin is superior to placebo in reducing CV death” added to secondary objectives.<br>“To determine whether dapagliflozin is superior to placebo in reducing the proportion of patients with worsened NYHA class” moved from secondary to exploratory objectives.<br>“To compare the effect of dapagliflozin versus placebo on health status assessed by Patient global impression of severity (PGIS) questionnaires” removed. Table 2 corrected accordingly.<br>Exploratory objective “To compare the effect of dapagliflozin versus placebo on EuroQol five-dimensional five-level questionnaire (EQ- 5D-5L)” was updated to:<br>“To describe health status assessed by EuroQol five-dimensional five-level questionnaire (EQ- 5D-5L)”.<br>Estimated date of last patient completed changed to: Q4 2021.<br>Study duration was prolonged to 39 months.<br>Number of primary endpoint events changed from 844 to 1117.<br>Recruitment period prolonged up to 29 months.<br>Statistical methods section updated to reflect the changes to the primary objective, multiple testing procedure and the increased event target.
3.	2.1 Study Rationale	Section updated to reflect current amendment changes about the two hypotheses, that dapagliflozin is superior to placebo in reducing the composite of CV death and HF events (hospitalisation for HF or urgent HF visit) in patients with HF and preserved systolic function (LVEF >40%), with or without T2D, in (1) the full population and in (2) an LVEF <60% subpopulation.
4.	4.1 Overall Design	Number of primary endpoints updated to 1117.<br>Anticipated total study duration time updated to 39 months.<br>It was added that Study Closure Visit (SCV) which should be hold within 6 weeks of the PACD, can be extended if decided by Global Study Team.
5.	4.2 Scientific rationale for study design	New paragraph added to justify the added testing of the treatment effect in patients with LVEF <60.

6.	8. Study Assessments and Procedures	It was clarified that during Visit 1, the investigator assesses patient's eligibility criteria and reviews concomitant medications, and relevant medications will be recorded.<br>COVID-19 testing was added to Safety events assessment during Visits: 3,4,5,6,7 and onwards, as well as Premature Treatment Discontinuation Visit and Study Closure Visit.<br>It was clarified that starting from Visit 6, vital signs (BP, pulse), and weight assessment as well as blood samples collection for creatinine (for calculation of eGFR) will be repeated every 12 months - on Visit 6, Visit 9 and Visit 12.
7.	8.3 Efficacy Assessments	An alternative phone collection mode solution was implemented for the administration of electronic PROs in settings that are affected by COVID-19 pandemic.
8.	8.4 Safety Assessments	COVID-19 testing results recording was added into Other safety assessments.
9.	8.5 Collection of adverse events	The process of Adjudication of potential DKA events by an independent DKA Committee was implemented (section 8.5.1.2.2.)<br>Requirements for capturing of Major hypoglycaemic events were added as section 8.5.1.4.
10.	9.2 Sample size determination	Section updated to reflect the dual primary hypothesis, changed multiple testing procedure and increased event target.
11.	9.3 Populations for analysis	A subset of the full analysis set consisting of patients with baseline LVEF of <60% (or LVEF <60% subpopulation) will be analysed separately as part of the confirmatory statistical testing procedure added to full analysis set.
12.	9.4 Statistical Analyses	Analysis of the primary variable updated with dual primary analysis.<br>Analysis of the secondary variables updated with regard to analysis of recurrent HF events and CV death for the LVEF < 60 subpopulation, and addition of time to CV death as a secondary endpoint.<br>Methods for multiplicity control updated according to the dual primary hypotheses and updated testing procedure.
13.	9.5 Interim Analyses	Clarification added that the interim analysis testing will be done in the full study population. Futility analysis was removed.

This Clinical Study Protocol has been subject to a peer review according to AstraZeneca Standard procedures. The Clinical Study Protocol is publicly registered and the results are disclosed and/or published according to the AstraZeneca Global Policy on Bioethics and in compliance with prevailing laws and regulations.

TABLE OF CONTENTS

TITLE PAGE ... 1
VERSION HISTORY ... 2
TABLE OF CONTENTS ... 6
1. PROTOCOL SUMMARY ... 11
1.1 Schedule of Activities (SoA) ... 12
1.2 Synopsis ... 15
1.3 Schema ... 19
2. INTRODUCTION ... 20
2.1 Study rationale ... 20
2.2 Background ... 21
2.3 Benefit/risk assessment ... 21
2.3.1 Potential risks to patients ... 22
2.3.1.1 Protection against risks ... 22
2.3.2 Potential benefits to patients ... 23
2.3.4 Conclusion ... 23
3. OBJECTIVES AND ENDPOINTS ... 24
4. STUDY DESIGN ... 26
4.1 Overall design ... 26
4.2 Scientific rationale for study design ... 27
4.3 Justification for dose ... 28
4.4 End of study definition ... 29
5. STUDY POPULATION ... 29
5.1 Inclusion criteria ... 31
5.2 Exclusion criteria ... 32
5.3 Lifestyle restrictions (not applicable) ... 33
5.4 Screen failures ... 33
5.5 Procedures for handling of randomized not eligible patients ... 34
6. STUDY TREATMENTS ... 35
6.1 Treatments administered ... 35
6.2 Preparation/handling/storage/accountability ... 35
6.3 Measures to minimise bias: randomisation and blinding ... 36
6.3.1 Stratification and capping ... 37
6.3.1.1 Stratification ... 37
6.3.1.2 Capping ... 37

6.4 Treatment compliance ... 37
6.5 Concomitant therapy ... 38
6.5.1 Prohibited medication ... 38
6.5.2 Recording of concomitant treatment ... 38
6.5.3 Heart failure background standard of care ... 38
6.5.4 Anti-diabetes treatment ... 38
6.5.4.1 Background ... 38
6.5.4.2 Treatment of patients with established diagnosis of type 2 diabetes ... 38
6.5.5 Other concomitant treatment ... 39
6.6 Dose modification (not applicable) ... 39
6.7 Treatment after the end of the study ... 39
7. DISCONTINUATION OF TREATMENT AND SUBJECT WITHDRAWAL ... 39
7.1 Discontinuation of study treatment ... 39
7.1.1 Temporary discontinuation ... 39
7.1.1.1 Unexpected acute declines in eGFR ... 40
7.1.1.2 Volume depletion/hypotension ... 40
7.1.1.3 Patients at risk of volume depletion ... 40
7.1.2 Procedures for discontinuation of study treatment ... 40
7.2 Lost to follow-up ... 41
7.3 Withdrawal from the study ... 41
8. STUDY ASSESSMENTS AND PROCEDURES ... 42
8.1 Enrolment Period ... 42
8.1.1 Visit 1, Enrolment (Day -21 to Day -1) ... 42
8.2 Treatment period ... 43
8.2.1 Visit 2, Randomisation (Day 1) ... 43
8.2.2 Visit 3 (Day 30; ±7 days): ... 44
8.2.3 Visit 4 (Day 120 ±7 days): ... 44
8.2.4 Visit 5 (Day 240 ±7 days) ... 45
8.2.5 Visit 6 (Day 360 ±7 days) ... 45
8.2.6 Visit 7 and onwards (Day 480 and every 120 days ±14 days) ... 46
8.2.7 Premature Treatment Discontinuation Visit ... 46
8.2.8 Study Closure Visit ... 47
8.2.9 Unscheduled visits ... 47
8.3 Efficacy assessments ... 48
8.3.1 Efficacy event capture ... 48
8.3.2 Efficacy event adjudication ... 48
8.3.3 Clinical Outcome Assessments (COA) ... 48
8.3.3.1 KCCQ ... 48
8.3.3.2 Patient Global Impression of Severity (PGIS) ... 49
8.3.3.3 EuroQoL five-dimensional five-level questionnaire (EQ-5D-5L) ... 49
8.3.3.4 Administration of electronic PROs ... 50
8.4 Safety assessment ... 50
8.4.1 Physical examinations ... 51

8.4.2 Vital Signs ... 51
8.4.3 Electrocardiogram ... 51
8.4.4 Safety laboratory assessments ... 51
8.4.5 Other safety assessments ... 51
8.4.6 Other clinical assessments ... 51
8.4.6.1 Body weight and height ... 51
8.5 Collection of adverse events ... 52
8.5.1 Method of detecting AEs and SAEs ... 52
8.5.1.1 Adverse events (AEs) leading to amputation and potential risk factor AEs for amputations affecting lower limbs (“preceding events”) ... 52
8.5.1.2 Capture of DKA events ... 52
8.5.1.2.1 DKA definition ... 53
8.5.1.2.2 Diabetic Ketoacidosis Adjudication Committee T2D ... 53
8.5.1.3 Capture of cardiac ischaemic events and stroke ... 53
8.5.1.4 Capture of Major hypoglycaemic events ... 53
8.5.1.5 Capture of additional laboratory values ... 54
8.5.2 Time period and frequency for collecting AE and SAE information ... 54
8.5.3 Follow-up of AEs and SAEs ... 54
8.5.4 Adverse event data collection ... 54
8.5.5 Causality collection ... 55
8.5.6 Adverse events based on signs and symptoms ... 55
8.5.7 Adverse events based on examinations and tests ... 55
8.5.8 Disease-under study (DUS) (not applicable) ... 56
8.5.9 Disease progression (not applicable) ... 56
8.6 Safety reporting and medical management ... 56
8.6.1 Reporting of serious adverse events ... 56
8.6.1.1 Reporting of SAEs considered to be potential endpoints ... 56
8.6.2 Pregnancy ... 57
8.6.2.1 Maternal exposure ... 57
8.6.3 Overdose ... 57
8.7 Pharmacokinetics (not applicable) ... 58
8.8 Pharmacodynamics (not applicable) ... 58
8.9 Optional exploratory genetics ... 58
8.10 Biomarkers (not applicable) ... 58
8.11 Health Economics (not applicable) ... 58
9. STATISTICAL CONSIDERATIONS ... 58
9.1 Statistical hypotheses ... 58
9.2 Sample size determination ... 58
9.3 Populations for analyses ... 60
9.4 Statistical analyses ... 60
9.4.1 Efficacy analyses ... 60
9.4.1.1 Analysis of the primary variable ... 60

9.4.1.2 Analysis of the secondary variables ... 61
9.4.1.3 Subgroup analysis ... 61
9.4.1.4 Sensitivity analysis ... 62
9.4.2 Safety analyses ... 62
9.4.3 Methods for multiplicity control ... 62
9.5 Interim analyses ... 62
9.5.1 Data monitoring committee (DMC) ... 63
10. REFERENCES ... 64
11. SUPPORTING DOCUMENTATION AND OPERATIONAL CONSIDERATIONS ... 69

LIST OF TABLES

Table 1 Study of Assessments ... 12
Table 2 Objectives and Endpoints ... 16
Table 3 Study objectives ... 24
Table 4 Study Treatments ... 35
Table 5 Population for analysis ... 60

LIST OF FIGURES

Figure 1 Study design ... 19

LIST OF APPENDICES

Appendix A Regulatory, ethical and study oversight considerations ... 69
Appendix B Adverse event definitions and additional safety information ... 72
Appendix C Cardiovascular related events ... 76
Appendix D Genetics ... 79
Appendix E Handling of Human Biological Samples ... 82
Appendix F Actions required in cases of increases in liver biochemistry and evaluation of Hy's Law (not applicable) ... 84
Appendix G Medical device incidents: definition and procedures for recording, evaluating, follow-up, and reporting (not applicable) ... 84
Appendix H Abbreviations ... 85
Appendix I New York Heart Association (NYHA) Functional Classification ... 86

Appendix J The KC Cardiomyopathy Questionnaire......86
Appendix K EQ-5D-5L Questionnaire......91
Appendix L Patient Global Impression of Severity for Heart Failure Symptoms......95

1. PROTOCOL SUMMARY

1.1 Schedule of Activities (SoA)

Table 1 Study of Assessments

**Visit**	**1 Enrolment**	**2 Randomisation**	**3**	**4**	**5**	**6**	**7 – onwards**	**Premature Treatment Discontinuation Visit**	**Study Closure Visit**	**For details see Section:**
**Day/Month**	**Day -21 to Day -1**	**Day 1**	**Day 30 (±7)**	**Day 120 (±7)**	**Day 240 (±7)**	**Day 360 (±7)**	**Day 480 - onwards (every 120 days ±14 days)**		**≤ 6 weeks after PACD**	
Informed consent	X[6]									5.1, A 3
Inclusion/exclusion criteria	X	X								5.1, 5.2
Demographics	X									5.1
Medical history	X	X								5.1
Concomitant medication	X	X	X	X	X	X	X	X	X	6.5
Cardiac and HF related procedures			X	X	X	X	X	X	X	8.5.1.3
Physical exam	X	X							X	8.4.1
Systolic and diastolic BP	X	X	X			X[3]	X[3]	X	X	5.2, 8.4.2
Pulse	X	X	X			X[3]	X[3]	X	X	5.2, 8.4.2
Weight	X					X[3]	X[3]	X	X	8.4.6.1
Height	X									8.4.6.1
NYHA classification	X	X	X	X	X			X	X	5.1, Appendix I
12-lead ECG	X									8.4.3

**Visit**	**1 Enrolment**	**2 Randomisation**	**3**	**4**	**5**	**6**	**7 – onwards**	**Premature Treatment Discontinuation Visit**	**Study Closure Visit**	**For details see Section:**
**Day/Month**	**Day -21 to Day -1**	**Day 1**	**Day 30 (±7)**	**Day 120 (±7)**	**Day 240 (±7)**	**Day 360 (±7)**	**Day 480 - onwards (every 120 days ±14 days)**		**≤ 6 weeks after PACD**	
C-lab NT-proBNP	X									5.1
C-lab eGFR (creatinine)	X		X	X		X[3]	X[3]			5.2, 8.4.4
C-lab HbA1c	X									6.3.1.1
Sample for genetic research, if applicable[5]		X								Appendix D
KCCQ		X[4]	X[4]	X[4]	X[4]			X[4]	X[4]	8.3.3.1
PGIS		X[4]	X[4]	X[4]	X[4]			X[4]	X[4]	8.3.3.2
EQ-5D-5L		X[4]			X[4]			X[4]	X[4]	8.3.3.3
Local pregnancy test (female patients with childbearing potential only)		X								5.1
Randomisation		X								8.2.1
Dispense investigational product (IP)		X		X	X	X	X			6
Collect unused IP; check IP compliance				X	X	X	X	X	X	6

**Visit**	**1 Enrolment**	**2 Randomisation**	**3**	**4**	**5**	**6**	**7 – onwards**	**Premature Treatment Discontinuation Visit**	**Study Closure Visit**	**For details see Section:**
**Day/Month**	**Day -21 to Day -1**	**Day 1**	**Day 30 (±7)**	**Day 120 (±7)**	**Day 240 (±7)**	**Day 360 (±7)**	**Day 480 - onwards (every 120 days ±14 days)**		**≤ 6 weeks after PACD**	
Efficacy events (death and worsening heart failure) [1]		X[1]	X	X	X	X	X	X	X	8.3
Safety events[2,7]	X	X	X	X	X	X	X	X	X	8.4

AEs Adverse events; DAEs Adverse events leading to discontinuation of investigational product; PACD Primary Analysis Censoring Date; SAEs Serious adverse events; C-lab Central laboratory

[1] Efficacy events are considered as endpoints from time of randomisation and throughout the study. Prior to randomisation, these events are considered as SAEs.

[2] SAEs will be recorded from the time of informed consent. DAEs and Amputations, adverse events (AEs) leading to amputation and potential risk factor AEs for amputations affecting lower limbs will be recorded from Visit 2 onwards.

[3] Assessments to be repeated every 12 months (Visit 6, Visit 9, Visit 12).

[4] Will be administered using a site-based electronic device. It is preferred that PRO questionnaires are completed prior to any other study procedures and before discussion of disease progression to avoid biasing the patient's responses to the questions

[5] Blood sample for future genetic research is optional. The genetic sampling is subject to separate consent by the patient.

[6] The Patient signs the ICF. Patients who agree to the optional sampling of blood for genetic research will provide their consent.

[7] Including recording of COVID-19 testing results from Visit 2 onwards.

1.2 Synopsis

International coordinating Investigator

Scott D. Solomon, MD

The Edward D. Frohlich Distinguished Chair
Professor of Medicine
Harvard Medical School
Senior Physician
Brigham and Women's Hospital
75 Francis Street
Boston, MA 02115
US

Protocol Title:

An International, Double-blind, Randomised Placebo-Controlled Phase III Study to Evaluate the Effect of Dapagliflozin on Reducing CV Death or Worsening Heart Failure in Patients with Heart Failure with Preserved Ejection Fraction (HFpEF).

Rationale:

The prevalence of chronic heart failure (HF) continues to increase globally, and the annual global economic burden (several hundred billion dollars in 2012) will increase as the population ages. Approximately half of all heart failure patients have heart failure with preserved ejection fraction (HFpEF) representing a particularly significant unmet need given that no approved pharmacotherapy exists specifically for this condition. Patients with HFpEF generally receive diuretic treatment for symptom relief, and should receive guideline recommended therapies for concomitant diseases such as hypertension. Recent data from cardiovascular (CV) outcome trials of SGLT2 inhibitors (empagliflozin and canagliflozin) and real world studies (including patients treated with dapagliflozin) indicate that treatment with SGLT2 inhibitors can reduce the risk of CV death and hospitalisation due to HF in patients with Type 2 Diabetes (T2D) overall and in patients with T2D and concomitant HF. Limitations associated with the randomised clinical trials as well as the observational studies are that only patients with T2D were studied, and that the proportion of patients with HFrEF and HFpEF, respectively, is unknown. This study will test the hypothesis that dapagliflozin will reduce the composite of CV death and HF events (hospitalisation for HF or urgent HF visit) in patients with HF and preserved systolic function (LVEF >40%), with or without T2D.

Table 2 Objectives and Endpoints

Primary objective:	Endpoint/variable:
To determine whether dapagliflozin is superior to placebo, when added to standard of care, in reducing the composite of CV death and HF events (hospitalisation for HF or urgent HF visit) in patients with HF and preserved systolic function in<br>• full study population<br>• subpopulation with LVEF <60%	Time to the first occurrence of any of the components of this composite:<br>1. CV death<br>2. Hospitalisation for HF<br>3. Urgent HF visit (e.g., emergency department or outpatients visit)
**Secondary objective:**	**Endpoint/variable:**
To determine whether dapagliflozin is superior to placebo in reducing the total number of HF events (hospitalisation for HF or urgent HF visit) and CV death in<br>• full study population<br>• subpopulation with LVEF <60%	Total number of HF events (first and recurrent) and CV death
To determine whether dapagliflozin is superior to placebo in improving Patient Reported Outcomes measured by KCCQ	Change from baseline in the total symptom score (TSS) of the KCCQ at 8 months
To determine whether dapagliflozin is superior to placebo in reducing CV death	Time to the occurrence of CV death
To determine whether dapagliflozin is superior to placebo in reducing all-cause mortality	Time to the occurrence of death from any cause
**Safety objective:**	
To evaluate the safety and tolerability of dapagliflozin compared to placebo in patients with HFpEF	Serious adverse events (SAEs), adverse events leading to treatment discontinuation (DAEs), amputations, adverse events (AEs) leading to amputation and potential risk factor AEs for amputations affecting lower limbs
**Exploratory Objective:**	
To determine whether dapagliflozin is superior to placebo in reducing all-cause hospitalisation	Time to the first occurrence of hospitalisation from any cause

To determine whether dapagliflozin is superior to placebo in reducing the proportion of patients with worsened NYHA class	Proportion of patients with worsened NYHA class from baseline to 8 months
To describe health status assessed by EuroQol five-dimensional five-level questionnaire (EQ-5D-5L) to support health economic analysis and health technology assessment	Results will be reported separately in a health economic report
To determine whether dapagliflozin compared with placebo will have an effect on systolic BP	Change in systolic BP from baseline
To determine whether dapagliflozin compared with placebo will have an effect on body weight	Change in body weight from baseline
To determine whether dapagliflozin compared with placebo will have an effect on eGFR.	Change in eGFR from baseline
To explore whether dapagliflozin compared to placebo improves KCCQ summary scores, subscores of TSS (Symptom frequency and symptom burden) and domains	Change in Clinical summary score, TSS subscores, Overall summary score, QoL score
To collect and store blood samples for future exploratory genetic research	Not applicable. Results will be reported separately

BP Blood pressure; CV Cardiovascular; EQ-5D-5L EuroQol five-dimensional five-level questionnaire
HF Heart failure; HFrEF Heart failure with reduced ejection fraction; KCCQ Kansas City Cardiomyopathy Questionnaire NYHA New York Heart Association

Overall design:

This is an international, multicentre, parallel-group, event-driven, randomised, double- blind study in patients with HFpEF, evaluating the effect of dapagliflozin 10 mg versus placebo, given once daily in addition to background regional standard of care therapy, including treatments to control co- morbidities, in reducing the composite of CV death and heart failure events (hospitalisations for HF or urgent HF visits). Adult patients aged ≥40 years with HFpEF (LVEF >40% and evidence of structural heart disease) and New York Heart Association (NYHA) class II-IV who are eligible according to the inclusion/exclusion criteria will be randomised in a 1:1 ratio to receive either dapagliflozin 10 mg or placebo. Both out-patients and in-patients hospitalised for heart failure and off intravenous heart failure-therapy for 24 hours can be randomised. It is estimated that approximately 11000 patients at approximately 400-500 sites in 20-25 countries will need to be enrolled to reach approximately 6100 randomised patients.

Study Period:

Estimated date of first patient enrolled: Q3 2018

Estimated date of last patient completed: Q4 2021

Number of randomised Subjects: approximately 6100 patients

Treatments and treatment duration:

Patients will be randomised in a 1:1 ratio to receive either dapagliflozin 10 mg or placebo once daily. The original anticipated average treatment duration was 24 months (range 15 to 33 months). With updated sample size and increased target number of events, the maximum treatment duration is expected to be approximately 39 months.

Data Monitoring Committee:

An independent Data Monitoring Committee (DMC) will review accumulating trial data by treatment group in order to monitor patient safety and efficacy, ensure the validity and integrity of the trial, and make benefit-risk assessment.

Statistical methods

This study is event-driven with a target of 1117 patients with a primary endpoint event. The primary objective of the study is to determine the superiority of dapagliflozin versus placebo, when added to standard of care, in reducing the composite of CV death and HF events (hospitalisation for HF or urgent HF visit). Two hypotheses will be tested simultaneously (i.e, dual primary analyses) for this primary objective: (1) in the full population and in (2) an LVEF <60% subpopulation, with alpha allocated to each test. The final alpha split will be defined in the SAP prior to the interim analysis. It is anticipated that at least 70% of the events (i.e. approximately 780 events) will be available for the LVEF <60% subpopulation. To illustrate, assuming a true hazard ratio (HR) of 0.80 between dapagliflozin and placebo, a two-sided alpha of 1.5% would yield a power of 90% for the full population and a two-sided alpha of 2.4% would yield a power of 80% for the LVEF <60% subpopulation.

Based on above assumption and ongoing blinded monitoring of events accrual, approximately 6100 patients are estimated to provide the required number of primary events in the full population during an anticipated recruitment period up to 29 months and followed until the pre-specified number of primary events has occurred. Randomisation will be stratified by presence or absence of Type 2 Diabetes (T2D).

All patients who have been randomised to study treatment will be included in the Full Analysis Set (FAS) irrespective of their protocol adherence and continued participation in the study. The primary variable is the time to first event included in the primary composite endpoint. The primary analysis will be based on the intention to treat (ITT) principle using the FAS, including events occurring on or prior to the primary analysis censoring date (PACD), confirmed by adjudication.

In the analysis of the primary composite endpoint, treatments (dapagliflozin versus placebo) will be compared using a Cox proportional hazards model with a factor for treatment group, stratified by type 2 diabetes (T2D) status at randomisation. The p-value, hazard ratio and 95% confidence interval will be reported.

Interim analysis is planned to be performed including approximately 67% of target number of adjudicated primary endpoints.

A closed testing procedure including a pre-specified hierarchical ordering of the primary and secondary endpoints will be utilized. No multiplicity control is placed on the exploratory endpoints.

1.3 Schema

The general study design is summarised in Figure 1.

Figure 1 Study design

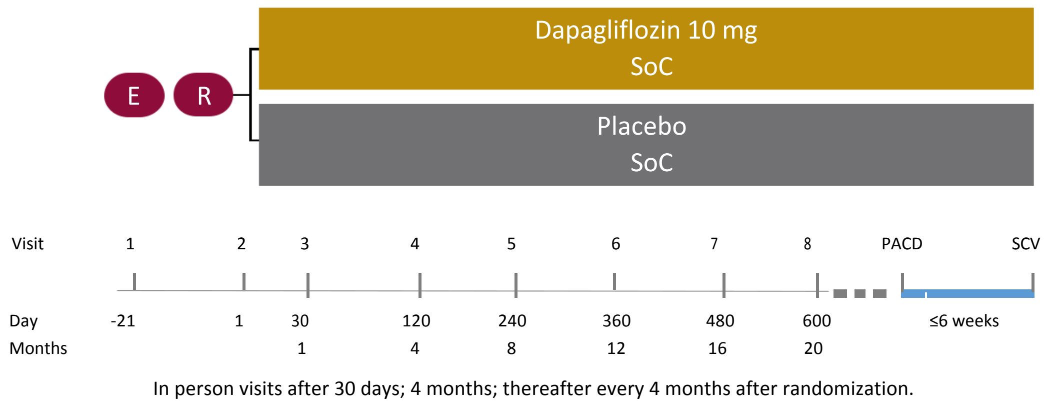

E=Enrolment; R=Randomization; SoC= Standard of Care; PACD=Primary Analysis Censoring Date; SCV=Study Closure Visit; FU=Follow Up

2. INTRODUCTION

2.1 Study rationale

The prevalence of chronic HF continues to increase globally. An estimated 38 million people are affected worldwide (Braunwald 2015), with over 1 million hospitalisations annually in both the United States and Europe (Ambrosy et al 2014). The annual global economic burden in 2012 was estimated to be $108 billion, (Cook et al 2014); this will increase dramatically as the population ages.

Heart failure is a complex syndrome caused by structural and/or functional abnormalities. It is characterised by dyspnoea, fatigue, and pulmonary congestion and/or peripheral oedema due to fluid retention. Patients with signs and symptoms of HF are categorised, based on measurement of left-ventricular ejection fraction (LVEF), as having HF with reduced LVEF (HFrEF) or HF with preserved LVEF (HFpEF).

Approximately half of all heart failure patients have HFpEF (Oktay et al 2013). Risk of death for HFpEF patients is high, with annualised mortality rate up to 15% in community settings (Lam et al 2011). In controlled clinical trials, patients with HFpEF tend to be older and have a higher prevalence of hypertension as compared to patients with HFrEF, although major clinical outcomes are similarly dominated by CV death and HF hospitalisation, the yearly event rates appear to be lower than in HFrEF (Solomon et al 2005). However, patients with HFpEF have a particularly significant unmet medical need given that outcome studies hitherto performed have not resulted in any approved pharmacotherapy specifically for this condition. Conversely, outcome studies have provided evidence for treatments for HFrEF that hence can improve symptoms and haemodynamics as well as reduce hospitalisations for heart failure and mortality. These treatments include diuretics, angiotensin converting enzyme inhibitors, angiotensin II receptor blockers, angiotensin II receptor blocker neprilysin inhibitors, mineralocorticoid receptor antagonists, and beta-blockers (Iwaz et al 2016).

Recent data from cardiovascular (CV) outcome trials of SGLT2 inhibitors (empagliflozin and canagliflozin) indicate that treatment with SGLT2 inhibitors can reduce the risk of CV death and hospitalisation due to HF in patients with T2D overall, and in patients with T2D and concomitant HF (Zinman et al 2015; Fitchett et al 2016; Neal et al 2017; Rådholm et al 2018).

Results from real-world observational studies are broadly consistent with the randomised clinical trials in supporting the benefits of SGLT2 inhibitors in reducing risk of HF hospitalisation and CV death. The CVD-REAL study, consisting of more than 300000 patients with T2D, both with and without established CV disease, across 6 countries found that patients treated with SGLT2 inhibitors compared to patients treated with other glucose lowering drugs was associated with a relative risk reduction in hospitalisation due to HF (39%), all-cause death (51%), and the composite of hospitalisation due to HF or CV death (46%) (Kosiborod et al 2017a).

Limitations associated with the randomised clinical trials as well as the observational studies are that only patients with T2D were studied, and that the proportion of patients with HFrEF and HFpEF, respectively, is unknown.

This study will simultaneously test the two hypotheses that dapagliflozin is superior to placebo in reducing the composite of CV death and HF events (hospitalisation for HF or urgent HF visit) in patients with HF and preserved systolic function (LVEF >40%), with or without T2D, in (1) the full population and in (2) an LVEF <60% subpopulation.

2.2 Background

Dapagliflozin is a potent, highly selective and orally active inhibitor of human renal SGLT2. A detailed description of the chemistry, pharmacology, efficacy, and safety of dapagliflozin is provided in the Investigator's Brochure. Supporting the hypothesis that dapagliflozin may reduce CV Death and HF events in HF patients, irrespective of diabetes status, are observations from the overall dapagliflozin clinical development programme. Dapagliflozin lowers HbA1c with a low risk of inducing hypoglycaemia. In addition, dapagliflozin treatment has also been shown to reduce weight and systolic blood pressure, and to have favourable effect on increased blood uric acid, albuminuria, and arterial elasticity, conditions which are associated with increased CV and renal risk (Shigiyama et al 2017). Dapagliflozin is believed be nephroprotective through non-glycaemic mechanisms (Wanner et al 2016).

The identified blood pressure lowering effects, may reduce the primary outcome in a study population with high prevalence of hypertension, similarly, the observed effects on body weight, may be beneficial to the large part of the study population with obesity. The findings from EMPA-REG, with a similar SGLT2 inhibitor compound, suggests that kidney function is preserved, or improved in this diabetic study population. Furthermore, HFpEF patients are characterized by fluid retention and a change in cardiac metabolism favouring glucose as substrate, both of which has been hypothesised to be positively impacted by SGLT2 inhibitor treatment. Moreover, arterial stiffness, and abnormal ventriculo-arterial coupling, are common in patients with HFpEF, and may be modified by SGLT2 inhibitor treatments.

The clinical studies in healthy subjects at high multiple doses also show that, due to the mechanism of action, dapagliflozin does not induce hypoglycemia in nondiabetic subjects; however, pharmacodynamic effects on glucose, sodium, and urinary volume are observed. Therefore, the changes in these diabetes-independent mechanisms and intrarenal physiology are expected to be similar regardless of underlying disease.

This study is an international, multicentre, parallel-group, event-driven, randomised, double-blind, placebo-controlled study in HFpEF patients, evaluating the effect of dapagliflozin 10 mg, given once daily in addition to background regional standard of care therapy, including treatments to control co-morbidities, in reducing the composite of CV death and heart failure events (hospitalisations for HF or urgent HF visits).

2.3 Benefit/risk assessment

Dapagliflozin has global marketing approval in approximately 90 countries with the most recent estimate of cumulative post-marketing experience totalling over 1.6 million patient-years. Detailed information about the known and expected benefits and risks and reasonably expected adverse events of dapagliflozin appears in the Investigator's Brochure. The following is a summary of benefit-risk considerations relevant to the HFpEF target population.

2.3.1 Potential risks to patients

Dapagliflozin reduces blood volume and blood pressure from its diuretic effect, which could be a concern in patients with HFpEF, but also be important mechanisms of a potential treatment effect. However, in the dapagliflozin type 2 diabetes mellitus (T2D) program, the rate of events related to volume depletion and impaired renal function have been similar between dapagliflozin and placebo. Loop-diuretics are widely used in the target patient population and are also allowed in this study. A pooled analysis of patients with T2D and HF in the dapagliflozin development program, showed no increase of volume depletion events but increase in renal events, mainly creatinine increases, in patients treated with dapagliflozin (n=171) compared with placebo treated patients (n=149). About half of the patients were on loop diuretics (Kosiborod et al 2017b).

An increase in amputations, mostly affecting toes, was observed in a clinical trial (Neal et al 2017) with another SGLT2 inhibitor. There is no indication from the clinical development program that dapagliflozin is associated with an increased risk of amputation (see Section 8.5.1.1 for the detection and capture of amputation events).

Dapagliflozin has not been shown to induce hypoglycaemia in non-diabetes patients. In clinical pharmacology studies, healthy subjects have been treated with single oral doses up to 500 mg and multiple oral doses of 100 mg up to 14 days without any hypoglycaemic events.

There have been post-marketing reports of ketoacidosis, including diabetic ketoacidosis, in patients with T2D taking dapagliflozin and other SGLT2 inhibitors, although a causal relationship has not been established.

Patients treated with dapagliflozin who present with signs and symptoms consistent with ketoacidosis, including nausea, vomiting, abdominal pain, malaise, and shortness of breath, should be assessed for ketoacidosis, even if blood glucose levels are below 14 mmol/L (250 mg/dL). If ketoacidosis is suspected interruption of dapagliflozin treatment should be considered and the patient should be promptly evaluated.

Predisposing factors to ketoacidosis include a low beta-cell function reserve resulting from pancreatic disorders (e.g., T1D, history of pancreatitis, or pancreatic surgery), insulin dose reduction, reduced caloric intake, or increased insulin requirements due to infections, illness or surgery and alcohol abuse. Dapagliflozin should be used with caution in patients in these circumstances. Dapagliflozin is currently not indicated for the treatment of patients with T1D; these patients are excluded from this study.

2.3.1.1 Protection against risks

This study has been designed with appropriate measures in place to monitor and minimise any potential risks to participating patients. Data regarding amputations and adverse events potentially placing the patient at risk for a lower limb amputation will be collected (see Section 8.5.1.1). To ensure the safety of all patients participating in AstraZeneca sponsored studies, reviews of all safety information from all ongoing clinical dapagliflozin studies are conducted as they become available. In addition, an independent Data Monitoring Committee (DMC) will be responsible for safeguarding the interests of the patients by reviewing safety data throughout the study (see Section 9.5.1).

2.3.2 Potential benefits to patients

All patients in the study are expected to be optimally treated according to regional standard of care therapy, including treatments to control co-morbidities, and dapagliflozin or placebo will be administered on top of this treatment.

All patients participating in clinical trials irrespective of whether treated with active treatment or not, generally receive closer medical attention than those in ordinary clinical practice which may be to their advantage.

2.3.3 Conclusion

Considering the non-clinical and clinical experience with dapagliflozin and the precautions included in the study protocol, participation in this study should present a minimal and thus acceptable risk to eligible patients. Although hypothesis-generating data suggest beneficial effects of SGLT2 inhibitors in patients with T2D with heart failure, at the time of writing of this clinical study protocol, no available SGLT2 inhibitor has a treatment indication for patients with HFpEF. The proposed clinical study will test the hypothesis that dapagliflozin reduces the risk of CV death and HF events in patients with HFpEF, with or without T2D, in a rigorous fashion. The results could potentially offer substantial benefit to patients with HFpEF, a patient population with a large medical need for effective treatments.

3. OBJECTIVES AND ENDPOINTS

Table 3 Study objectives

**Primary objective:**	**Endpoint/variable:**
To determine whether dapagliflozin is superior to placebo, when added to standard of care, in reducing the composite of CV death and HF events (hospitalisation for HF or urgent HF visit) in patients with HF and preserved systolic function in<br>• full study population<br>• subpopulation with LVEF <60%	Time to the first occurrence of any of the components of this composite:<br>4. CV death<br>5. Hospitalisation for HF<br>6. Urgent HF visit (e.g., emergency department or outpatients visit)
**Secondary objective:**	**Endpoint/variable:**
To determine whether dapagliflozin is superior to placebo in reducing the total number of HF events (hospitalisation for HF or urgent HF visit) and CV death in<br>• full study population<br>• subpopulation with LVEF <60%	Total number of HF events (first and recurrent) and CV death
To determine whether dapagliflozin is superior to placebo in improving Patient Reported Outcomes measured by KCCQ	Change from baseline in the total symptom score (TSS) of the KCCQ at 8 months
To determine whether dapagliflozin is superior to placebo in reducing CV death	Time to the occurrence of CV death
To determine whether dapagliflozin is superior to placebo in reducing all-cause mortality	Time to the occurrence of death from any cause
**Safety objective:**	
To evaluate the safety and tolerability of dapagliflozin compared to placebo in patients with HFpEF	Serious adverse events (SAEs), adverse events leading to treatment discontinuation (DAEs), amputations, adverse events (AEs) leading to amputation and potential risk factor AEs for amputations affecting lower limbs
**Exploratory Objective:**	
To determine whether dapagliflozin is superior to placebo in reducing all-cause hospitalisation	Time to the first occurrence of hospitalisation from any cause

To determine whether dapagliflozin is superior to placebo in reducing the proportion of patients with worsened NYHA class	Proportion of patients with worsened NYHA class from baseline to 8 months
To describe health status assessed by EuroQol five-dimensional five-level questionnaire (EQ-5D-5L) to support health economic analysis and health technology assessment	Results will be reported separately in a health economic report
To determine whether dapagliflozin compared with placebo will have an effect on systolic BP	Change in systolic BP from baseline
To determine whether dapagliflozin compared with placebo will have an effect on body weight	Change in body weight from baseline
To determine whether dapagliflozin compared with placebo will have an effect on eGFR.	Change in eGFR from baseline
To explore whether dapagliflozin compared to placebo improves KCCQ summary scores, subscores of TSS (Symptom frequency and symptom burden) and domains	Change in Clinical summary score, TSS subscores, Overall summary score, QoL score
To collect and store blood samples for future exploratory genetic research	Not applicable. Results will be reported separately

BP Blood pressure; CV Cardiovascular; EQ-5D-5L EuroQol five-dimensional five-level questionnaire
HF Heart failure; HFrEF Heart failure with reduced ejection fraction; KCCQ Kansas City Cardiomyopathy Questionnaire NYHA New York Heart Association

4. STUDY DESIGN

4.1 Overall design

This is an international, multicentre, parallel-group, event-driven, randomised, double-blind, placebo-controlled study in HFpEF patients, evaluating the effect of dapagliflozin 10 mg versus placebo, given once daily in addition to background regional standard of care therapy, including treatments to control co-morbidities, in reducing the composite of CV death or heart failure events.

For an overview of the study design see Figure 1, Section 1.3. For details on treatments given during the study, see Section 6.1.

For details on what is included in the efficacy and safety endpoints, see Section 3 Objectives and Endpoints.

Adult patients with HFpEF (defined for the purposes of this study as LVEF >40% and evidence of structural heart disease) aged ≥40 years and with NYHA class II- IV who meet the inclusion criteria, and none of the exclusion criteria, will be randomised in a 1:1 ratio to receive either dapagliflozin 10 mg or placebo. Randomised treatment should be started as soon as possible and within 24 hours after randomisation. It is estimated that approximately 11000 patients at approximately 400-500 sites in 20-25 countries will be enrolled to reach approximately 6100 randomised patients (see Section 9.2).

Study closure procedures will be initiated when the predetermined number of primary endpoints are predicted to have occurred (n=1117), i.e. the Primary Analysis Censoring Date (PACD). Patients should be scheduled for a Study Closure Visit (SCV) within 6 weeks of the PACD, which can be extended if decided by Global Study Team. The anticipated total study duration is approximately 39 months dependent on randomisation rate and event rate. The study duration, and the number of patients, may be changed if the randomisation rate or the event rate is different than anticipated. The study may be terminated early if a clear harmful effect of the study treatment is detected during the DMC review, or due to DMC recommendations following pre-specified interim analyses (see Section 9.5).

Data on baseline characteristics, endpoints and AEs will be collected through a validated electronic data capture (EDC) system with electronic case report forms (eCRFs).

4.2 Scientific rationale for study design

This is a randomised, multi-centre, double-blind, parallel-group study. Randomisation and double-blinding will minimise potential bias. The target population includes adult (aged ≥ 40 years) male and female patients with HFpEF, which is defined in this study as individuals with an established diagnosis of heart failure and a LVEF >40% and structural heart disease who meet natriuretic peptide thresholds. The requirement of demonstrated structural heart disease (i.e. left ventricular hypertrophy or left atrial enlargement[1]) and elevated natriuretic peptides aims to support the diagnosis of heart failure, since other common co-morbidities may cause overlapping symptoms. Most randomised patients will be out-patients. However, to address a specific need in a period with high risk for events, a proportion of patients will be enrolled and randomised during hospitalisation for heart failure or within 30 days of discharge from hospitalisation for heart failure (subacute subgroup).

The study population will include patients both with and without T2D, as the beneficial haemodynamic effects of dapagliflozin appear to be independent of the glycaemic effect, and can therefore be expected in both groups. Enrolment in the study may be capped based on the proportion of patients with/without T2D, in certain LVEF categories, in each NYHA class, with/without atrial fibrillation, randomised during or early after HF hospitalisation (subacute subgroup), and geographic region.

The control group will receive placebo; there are no approved pharmacological treatments for HFpEF that could be utilised as a comparator. All patients will be treated according to local guidelines on standard of care treatment for patients with HFpEF, focusing on treatment of HF symptoms (e.g. diuretics) and comorbidities (including treatment for high blood pressure, ischaemic heart disease, atrial fibrillation).

The study population will include patients with eGFR ≥ 25 $ml/min/1.73m^2$. Patients with reduced renal function have a clinical picture with increased intra-glomerular pressure, hypertension, proteinuria and fluid/sodium overload and SGLT2 inhibition can improve all these abnormalities through metabolic-independent mechanisms. Thus, patients with heart failure and reduced renal function could be expected to benefit from treatment with dapagliflozin.

The primary efficacy endpoints of the study are adjudicated CV death and HF events (hospitalisation for HF or urgent HF visit). The rationale for selecting CV death over all-cause death is the expectation that HF treatment will decrease CV death and not all potential causes of death (Zannad et al 2014). Heart failure events include both HF hospitalisations and unplanned HF visits requiring urgent treatment independently of whether the exacerbation of HF results in hospitalisation (according to CDISC definitions; Hicks et al 2014; Hicks et al 2018). These are the same endpoint definitions currently employed in the Sponsor's HFrEF outcome study (Dapa-HF; Study D1699C00001).

[1] Left Atrial Enlargement defined by at least 1 of the following: LA width (diameter) ≥3.8 cm or LA length ≥5.0 cm or LA area ≥20 cm^2 or LA volume ≥55 mL or LA volume index ≥29 mL/m

[2] Left Ventricular Hypertrophy defined by septal thickness or posterior wall thickness ≥1.1 cm

The rationale for including outpatient HF events, in addition to hospital admissions, is that it is the occurrence of worsening of the patient's condition necessitating treatment, and not the place of treatment, that is important. As stated in EMA Guidance 2016, '…patient are often managed for episodes of transient decompensation or worsening HF in outpatient settings (eg, emergency departments, observation units, other outpatient settings). The capture of events of worsening HF without hospitalisation may be warranted as an additional endpoint.' Including only hospital admissions is likely to overlook a modest but significant proportion of episodes of worsening HF (Skali et al 2014, Okumura et al 2016, Greene et al 2018).

While CV death and HF hospitalisations are clearly important to patients and health-care systems, the impact of HF on patients' symptoms and physical/social functioning is also important. In order to evaluate the treatment effects on these aspects of the impact of HF, we will use the Kansas City Cardiomyopathy Questionnaire (KCCQ), a disease-specific patient reported outcomes (PRO) measure developed for patients with chronic HF. The KCCQ has shown to be a valid, reliable and responsive measure for patients with HF (Greene et al 2018, Spertus et al 2005).

There has been a gradual accumulation of data that HF patients with mildly abnormal (or "mid-range") ejection fraction (LVEF 40-50%), although traditionally classified as HFpEF, may potentially benefit from therapies that have been shown to improve outcomes in HFrEF (Nauta et al 2017). During the course of the DELIVER trial, the PARAGON-HF trial was completed, randomizing patients with HFpEF to sacubitril/valsartan or valsartan alone. While the study failed to meet its primary objective (Solomon et al 2019), there appeared to be a differential treatment effect by LVEF with benefit largely seen in patients with LVEF 45-60% (Solomon et al 2020). This new data suggests that HFpEF with a high-normal LVEF may constitute different clinical entities than heart failure with low-normal or mildly reduced LVEF (Lam et al 2020). To account for this emerging information in DELIVER, it was decided to formally investigate the treatment effect in both the subset of patients with LVEF<60% and in the full study population.

4.3 Justification for dose

The 10 mg dose of dapagliflozin has a well-characterised efficacy and safety profile in the T2D clinical development program and is the recommended dose in the majority of countries worldwide.

From a pharmacokinetic perspective, the currently approved dapagliflozin dose of 10 mg once daily is appropriate for use in patients with HFpEF. Slightly higher systemic exposure to dapagliflozin is expected in HFpEF patients when symptomatic, based on the dual renal and hepatic metabolism of dapagliflozin and the lower perfusion of these organs in this patient group. However, the increase in systemic exposure of 10 mg dapagliflozin is not anticipated to warrant dose adjustment in HF patients. Moreover, the anticipated slightly higher systemic exposure to dapagliflozin is likely to be beneficial in HF patients, by compensating for the reduced renal perfusion and consequently lower renal glucose and sodium filtered loads in these patients. Doses lower than 10 mg are therefore unlikely to provide as much benefit to patients with HF as the 10-mg dose. Lastly, no changes in dose of concomitant medications in the HFpEF population are

needed due to a lack of clinically meaningful drug-drug interactions for dapagliflozin with current medications used for treatment of patients with HFpEF, including standard of care medications used to control co-morbidities in this patient group.

In the dapagliflozin clinical program, there are no dose-related SAEs that preclude the use of 10 mg as a preferred dose. Additionally, in a post-hoc analysis of data from 320 patients with a documented history of HF and concomitant T2D in placebo-controlled clinical trials, dapagliflozin 10 mg was found to be well tolerated in this population (Kosiborod et al 2017b).

There are mechanistic reasons for choosing the 10-mg dose as well. One hypothesis of underlying pathophysiology in HFpEF is abnormal pressure coupling between the left ventricle and aorta, and drugs that reduce aortic stiffness may have beneficial effects in patients with HFpEF (Borlaug and Paulus 2011). Studies examining the highest approved dose for empagliflozin have reported improvements in aortic elasticity (Chilton et al 2015,Cherney et al 2014); similar studies are ongoing with dapagliflozin. In a completed placebo-controlled study, treatment with dapagliflozin 10 mg resulted in improvements in parameters associated with arterial remodelling in addition to lowering blood pressure in patients with T2D (Ott et al 2017). This prior work suggests that selecting the 10-mg dose of dapagliflozin is reasonable from a mechanistic perspective to demonstrate a clinical effect.

4.4 End of study definition

The end of study is defined as the last expected visit/contact of the last subject undergoing the study.

The study may be terminated at individual study sites if the study procedures are not being performed according to GCP, or if no patients are recruited. Patients from terminated sites will have the opportunity to be transferred to another site to continue the study. AstraZeneca may also terminate the entire study prematurely if concerns for safety arise within this study or in any other study with dapagliflozin, or due to recommendation by the DMC. Regardless of the reason for termination, all data required by the protocol at the time of discontinuation of follow-up will be collected. In terminating the study, the Sponsor will ensure that adequate consideration is given to the protection of the patients' interests.

See Appendix A 6 for guidelines for the dissemination of study results.

5. STUDY POPULATION

Prospective approval of protocol deviations to recruitment and enrolment criteria, also known as protocol waivers or exemptions, is not permitted.

In this protocol, 'enrolled' patients are defined as those who sign the informed consent form (ICF) and received E-Code. The ICF process is described in Appendix A 3. 'Randomised' patients are defined as those who undergo randomisation and receive a randomisation code.

Patients are eligible to be randomised in the study only if all of the following inclusion criteria and none of the exclusion criteria apply. Enrolled patients who for any reason are not randomised are considered screen failures (see Section 5.4).

5.1 Inclusion criteria

Subjects are eligible to be randomised in the study only if all of the following inclusion criteria and none of the exclusion criteria apply:

1. Provision of signed informed consent prior to any study specific procedures.
2. Male or female patients age ≥40 years.
3. Documented diagnosis of symptomatic heart failure (NYHA class II-IV) at enrolment, and a medical history of typical symptoms/signs[2] of heart failure ≥6 weeks before enrolment with at least intermittent need for diuretic treatment.
4. Left Ventricular Ejection Fraction (LVEF) >40% and evidence of structural heart disease (i.e. left ventricular hypertrophy or left atrial enlargement[3]) documented by the most recent echocardiogram, and/or cardiac MR within the last 12 months prior to enrolment. For patients with prior acute cardiac events or procedures that may reduce LVEF, e.g. as defined in exclusion criterion 6, qualifying cardiac imaging assessment at least 12 weeks following the procedure/event is required.
5. NT-pro BNP ≥300 pg/ml at Visit 1 for patients without ongoing atrial fibrillation/flutter. If ongoing atrial fibrillation/flutter at Visit 1, NT-pro BNP must be ≥600 pg/mL.
6. Patients may be ambulatory, or hospitalized; patients must be off intravenous heart failure therapy (including diuretics) for at least 12 hours prior to enrolment and 24 hours prior to randomisation.

[2] Typical symptoms associated with heart failure: breathlessness, orthopnoea, paroxysmal nocturnal dyspnoea, reduced exercise tolerance, fatigue, tiredness, increased time to recover after exercise, ankle swelling;
Signs associated with Heart Failure:
More specific: elevated jugular venous pressure, hepatojugular reflex, third heart sound (gallop rhythm), laterally displaced apical impulse
Less specific: weight gain (>2 kg/week), weight loss (in advanced HF), tissue wasting (cachexia), cardiac murmur, peripheral oedema (ankle, sacral, scrotal), pulmonary crepitations, reduced air entry and dullness to percussion at lung bases (pleural effusion), tachycardia, irregular pulse, tachypnoea, cheyne stokes respiration, hepatomegaly, ascites, cold extremities, oliguria, narrow pulse pressure

[3] Left Atrial Enlargement defined by at least 1 of the following: LA width (diameter) ≥3.8 cm or LA length ≥5.0 cm or LA area ≥20 cm^2 or LA volume ≥55 mL or LA volume index ≥29 mL/m^2. Left Ventricular Hypertrophy defined by septal thickness or posterior wall thickness ≥1.1 cm

5.2 Exclusion criteria

1. Receiving therapy with an SGLT2 inhibitor within 4 weeks prior to randomisation or previous intolerance to an SGLT2 inhibitor.
2. Type 1 diabetes mellitus (T1D).
3. eGFR <25 mL/min/1.73 m^2 (CKD-EPI formula) at Visit 1.
4. Systolic blood pressure (BP) <95 mmHg on 2 consecutive measurements at 5-minute intervals, at Visit 1 or at Visit 2.
5. Systolic BP≥160 mmHg if not on treatment with ≥3 blood pressure lowering medications or ≥180 mmHg irrespective of treatments, on 2 consecutive measurements at 5-minute intervals, at Visit 1 or at Visit 2.
6. MI, unstable angina, coronary revascularization (percutaneous coronary intervention (PCI) or coronary artery bypass grafting (CABG)), ablation of atrial flutter/fibrillation, valve repair/replacement within 12 weeks prior to enrolment. Before enrolment, these patients must have their qualifying echocardiography and/or cardiac MRI examination at least 12 weeks after the event.
7. Planned coronary revascularization, ablation of atrial flutter/fibrillation and valve repair/replacement.
8. Stroke or transient ischemic attack (TIA) within 12 weeks prior to enrolment.
9. Probable alternative or concomitant diagnoses which in the opinion of the investigator could account for the patient's HF symptoms and signs (e.g. anaemia, hypothyroidism).
10. Body mass index >50 kg/m^2.
11. Primary pulmonary hypertension, chronic pulmonary embolism, severe pulmonary disease including COPD (i.e., requiring home oxygen, chronic nebulizer therapy or chronic oral steroid therapy, or hospitalisation for exacerbation of COPD requiring ventilatory assist within 12 months prior to enrolment).
12. Previous cardiac transplantation, or complex congenital heart disease. Planned cardiac resynchronisation therapy.
13. HF due to any of the following: known infiltrative cardiomyopathy (e.g. amyloid, sarcoid, lymphoma, endomyocardial fibrosis), active myocarditis, constrictive pericarditis, cardiac tamponade, known genetic hypertrophic cardiomyopathy or obstructive hypertrophic cardiomyopathy, arrhythmogenic right ventricular cardiomyopathy/dysplasia (ARVC/D), or uncorrected primary valvular disease.

14. A life expectancy of less than 2 years due to any non-cardiovascular condition, based on investigator's clinical judgement.
15. Inability of the patient, in the opinion of the investigator, to understand and/or comply with study medications, procedures and/or follow-up OR any conditions that, in the opinion of the investigator, may render the patient unable to complete the study.
16. Active malignancy requiring treatment (with the exception of basal cell or squamous cell carcinomas of the skin).
17. Acute or chronic liver disease with severe impairment of liver function (e.g., ascites, oesophageal varices, coagulopathy).
18. Women of child-bearing potential (i.e. those who are not chemically or surgically sterilised or post-menopausal) not willing to use a medically accepted method of contraception considered reliable in the judgment of the investigator OR who have a positive pregnancy test at randomisation OR who are breast-feeding.
19. Involvement in the planning and/or conduct of the study (applies to both AstraZeneca personnel and/or personnel at the study site).
20. Previous randomisation in the present study.
21. Participation in another clinical study with an IP or device during the last month prior to enrolment.

5.3 Lifestyle restrictions (not applicable)

5.4 Screen failures

Enrolled patients who are found not eligible (i.e. not meeting all the inclusion criteria or fulfilling any of the exclusion criteria) must not be randomised or initiated on treatment.

Screen failures are defined as patients who signed the informed consent form to participate in the study but are not subsequently randomised. A minimal set of screen failure information is required to ensure transparent reporting of screen failure patients to meet the Consolidated Standards of Reporting Trials (CONSORT) publishing requirements and to respond to queries from regulatory authorities. Minimal information includes demography, eligibility criteria (reason for screen failure), and any serious adverse event (SAE).

Screen failures may be re-enrolled one time during the study if the Investigator considers that the patient may be eligible for participation in this study at another time point. Re-enrolled patients should be assigned the same enrolment code as for the initial enrolment. All enrolment assessments and procedures, including signing the informed consent form, should be performed again.

5.5 Procedures for handling of randomized not eligible patients

If a patient is randomised and later found not eligible, the Investigator should immediately inform the AstraZeneca representative, who will report the protocol deviation to the AstraZeneca Study Physician.

Study treatment must be discontinued in all cases where continued treatment is deemed to pose a safety risk to the patient. Regardless of whether study treatment is discontinued or not, the patient should continue his/her participation in the study for follow- up of endpoints and other protocol-defined study procedures until the end of the study. Consistent with the intention-to-treat principle, all randomised patients are included in the efficacy analysis according to randomised treatment assignment. The AstraZeneca Study Physician must ensure that the protocol deviation and the rationale for the decision to discontinue or continue study treatment are appropriately documented.

6. STUDY TREATMENTS

Study treatment is defined as any investigational product(s) (including marketed product comparator and placebo) or medical device(s) intended to be administered to a study participant according to the study protocol. Study treatment in this study refers to dapagliflozin or matching placebo.

6.1 Treatments administered

Table 4 Study Treatments

	Dapagliflozin	Placebo
**Investigational Product name**	Dapagliflozin 10 mg	Matching placebo 10 mg
**Dosage formulation**	Green, diamond shaped, film coated tablets 10 mg	Green, diamond shaped, film coated tablets 10 mg placebo
**Route of administration**	Oral	Oral
**Dosing instructions**	Once daily	Once daily
**Packaging and labeling**	Investigational Product will be provided in bottles. Each bottle will be labelled in accordance with Good Manufacturing Practice Annex 13 and per country regulatory requirements	Investigational Product will be provided in bottles. Each bottle will be labelled in accordance with Good Manufacturing Practice Annex 13 and per country regulatory requirements
**Provider**	AstraZeneca	AstraZeneca

The tablets contain lactose, in quantities not likely to cause discomfort in lactose-intolerant individuals.

6.2 Preparation/handling/storage/accountability

The investigator or designee must confirm appropriate temperature conditions have been maintained during transit for all study treatment received and any discrepancies are reported and resolved before use of the study treatment.

All investigational product (IP) should be kept in a secure place under appropriate storage conditions. The label on the IP bottle specifies the appropriate storage.

Only patients randomised in the study may receive IP and only authorised site staff may supply or administer IP. The administration of all investigational products should be recorded in the appropriate sections of the eCRF.

The Investigator is responsible for IP accountability, reconciliation, and record maintenance (i.e. receipt, reconciliation, and final disposition records).

The investigator will retain the returned IP until the AZ representative or delegate collects it, along with any IP not dispensed. The AZ representative or delegate is responsible for confirming the investigator or delegate has recorded the quantities of returned and unused tablets at a patient level before IP is destroyed. The AZ representative or delegate will advise on the appropriate method for destruction of unused IP.

6.3 Measures to minimise bias: randomisation and blinding

All patients will be centrally assigned to randomised IP using an interactive voice/web response system (IxRS). Randomisation to IP will be performed in balanced blocks to ensure approximate balance between the treatment groups (1:1). The randomisation codes will be computer generated and loaded into the IxRS database. Before the study is initiated, the telephone number and call-in directions for the IxRS and/or the log-in information and directions for the IxRS will be provided to each site.

If a randomised patient withdraws from the study, then his/her enrolment/randomisation code cannot be reused. Withdrawn randomised patients will be included in the intention to treat analysis.

The IxRS will provide the Investigator with the kit identification number to be allocated to the patient at each dispensing visit. At all visits where IP is dispensed, site personnel will do a kit verification in IxRS before providing the IP bottle to the patient. Routines for this will be described in the IxRS user manual that will be provided to each centre.

The blinding of treatment is ensured by using a double-blind technique. Individual treatment codes, indicating the randomised treatment for each patient, will be available to the investigator(s) or pharmacists from the IxRS. Instructions for code breaking/unblinding will be described in the IxRS user manual that will be provided to each site.

The randomisation code should not be broken except in medical emergencies when the appropriate management of the patient requires knowledge of the treatment randomisation. The Investigator is to document and report the action to AstraZeneca, without revealing the treatment given to the patient to the AstraZeneca staff.

AstraZeneca retains the right to break the code for SAEs that are unexpected and are suspected to be causally related to an investigational product and that potentially require expedited reporting to regulatory authorities. Randomisation codes will not be broken for the planned analyses of data

until all decisions on the evaluability of the data from each individual patient have been made and documented.

6.3.1 Stratification and capping

The recruitment will be continuously monitored in order to achieve adequate proportions of patient sub-populations.

6.3.1.1 Stratification

Randomisation will be stratified in IxRS based on patients with and without T2D at the time of randomisation in order to ensure approximate balance between treatment groups within each sub-population. Stratification on T2D at the time of randomisation is based on:

- Established diagnosis of T2D

OR

- HbA1c ≥6.5% (48 mmol/mol) shown at central laboratory test at enrolment (Visit 1)

6.3.1.2 Capping

The intent is to enrol a typical cross-section of patients with HFpEF and to include representative proportions of diabetic and non-diabetic patients. The number of randomised patients with and without T2D will be monitored in order to ensure a minimum of 30% in each sub-population. Randomisation may be capped (i.e., no more patients can be randomised in a specific sub-population) if the pre-determined limit is reached.

Randomisation of patients based on geographic region will be monitored to ensure global representation. LVEF value, NYHA class, subacute subgroup (i.e. randomised in- hospital or within 30 days from discharge) and atrial fibrillation status at Visit 1 may be capped in IxRS to avoid over- or under-representation of these patient subgroups.

6.4 Treatment compliance

The administration of all IP should be recorded in the appropriate sections of the eCRF. Any change from the dosing schedule should be recorded in the eCRF.

Patients will be asked to return all unused IP and empty packages to the clinic at the site visit except Visit 3. At each visit, any patient found to be non-compliant will be counselled on the importance of taking their IP as prescribed. The investigator or delegate will enter the number of returned tablets in the eCRF.

The Investigational Product Storage Manager is responsible for managing IP from receipt by the study site until the destruction or return of all unused IP. The Investigator(s) is responsible for ensuring that the patient has returned all unused IP.

6.5 Concomitant therapy

All patients should be treated according to regional standard of care of HFpEF and existing comorbidities (including treatment of hypertension, ischemic heart disease, atrial fibrillation, diabetes, hyperlipidaemia). Background medications should be part of clinical practice and will not be provided by the Sponsor.

6.5.1 Prohibited medication

Concomitant treatment (i.e., treatment in combination with IP) with open label SGLT2 inhibitors e.g., dapagliflozin, empagliflozin, canagliflozin, ertugliflozin, tofogliflozin and luseogliflozin and fix dose combinations containing these drugs should not be used. Also in situations when the patient is not on IP, treatment with open label SGLT2 inhibitors during the study could interfere with the interpretation of study results. If treatment with an SGLT2 inhibitor alone or in combination is deemed essential, IP must be discontinued before that treatment is started.

6.5.2 Recording of concomitant treatment

Recording of relevant concomitant medications in the eCRF will be made according to the schedule of activities (Table 1). These include medications for cardiovascular conditions as well as diabetes mellitus.

6.5.3 Heart failure background standard of care

The patients should be on background standard of care therapies for patients with HFpEF according to local guidelines, including diuretics when needed to control symptoms and volume overload and adequate treatment of co-morbidities such as hypertension and ischaemic heart disease.

6.5.4 Anti-diabetes treatment

6.5.4.1 Background

More than 40% of patients with established HF are estimated to have T2D (Kristensen et al 2016). Therefore, it is expected that a large proportion of patients will have an established T2D diagnosis when included in this study and that some patients will develop T2D during the course of the study. Treatment of diabetes should follow established guidelines, such as according to glycaemic goals as recommended by the American Diabetes Association (ADA) and European Association for the Study of Diabetes (EASD) in their joint Position Statement (Inzucchi et al 2012, Inzucchi et al 2015).

6.5.4.2 Treatment of patients with established diagnosis of type 2 diabetes

Diabetes medications at baseline and during the study will be recorded in the eCRF. Patients with T2D at randomisation will continue their T2D treatment. SGLT2-inhibitors should be avoided (see Section 6.5.1). Patients treated with insulin or insulin secretagogues have a higher risk of experiencing hypoglycaemic events compared with those treated with other antidiabetic agents. If needed, T2D treatments may be adjusted at the discretion of the Investigator or diabetes health care provider.

6.5.5 Other concomitant treatment

Medications other than described above, which is considered necessary for the patient's safety and wellbeing, may be given at the discretion of the Investigator.

6.6 Dose modification (not applicable)

6.7 Treatment after the end of the study

The patients will stop taking IP at the study closure visit (SCV). Remaining IP will be collected at that time. Post-study treatment will not be provided by the Sponsor. Patients should receive standard of care therapy after the SCV, at the discretion of the Investigator.

7. DISCONTINUATION OF TREATMENT AND SUBJECT WITHDRAWAL

7.1 Discontinuation of study treatment

Discontinuation from study treatment is NOT the same thing as a withdrawal from the study. If the patient temporarily or permanently discontinues IP, the patient should remain in the study and it is important that the scheduled study visits and data collection continue according to the study protocol until study closure.

Patients may be discontinued from IP in the following situations:

- Contraindication to further dosing with IP, in the opinion of the Investigator, such as Adverse event or other safety reasons.
- Severe non-compliance with the study protocol.
- Diabetic ketoacidosis (DKA). Consider temporarily interrupting IP if DKA is suspected. If DKA is confirmed, IP should be discontinued permanently.
- Positive pregnancy test (discontinue IP and notify Sponsor representative).
- Patient decision. The patient is at any time free to discontinue treatment, without prejudice to further treatment.

See the Table 1 for data to be collected at the time of treatment discontinuation and follow-up and for any further evaluations that need to be completed.

7.1.1 Temporary discontinuation

Every attempt should be made to maintain patients on IP during the course of the study. If IP has been interrupted, it should be re-introduced as soon as, in the opinion of the Investigator, the patient's condition is stable.

7.1.1.1 Unexpected acute declines in eGFR

If an unexpected, acute decline in kidney function is observed, the patient should be evaluated and temporary interruption of IP should be considered. Volume depletion, hypotension, inter-current medical problems and concomitant drugs may cause increases in blood creatinine. Urinary tract infection and urinary obstruction should be considered (the latter especially in men). Several drugs may cause a decline in kidney function, especially non-steroidal anti-inflammatory drugs (NSAID) and certain antibiotics such as trimethoprim. If any drug is suspected of causing or contributing to worsening kidney function, their use should be re-considered.

7.1.1.2 Volume depletion/hypotension

Patients with clinically relevant symptoms/signs of suspected volume depletion and/or hypotension, should in addition to considering temporary interruption of IP have their regular medication reviewed, and consideration given to reducing the dose of, or stopping concomitant medications, as assessed on an individual basis, including diuretics and drugs that lower blood pressure. The need for conventional diuretics (or the dose of diuretic used) should be re-evaluated in light of the patient's symptoms and signs.

7.1.1.3 Patients at risk of volume depletion

Temporary interruption of IP may be considered in patients thought to be at risk of volume depletion/hypotension, such as patients with an acute medical illness potentially causing volume depletion because of inadequate fluid intake or fluid/blood loss (e.g. gastroenteritis, gastrointestinal haemorrhage), or those undergoing major surgery.

7.1.2 Procedures for discontinuation of study treatment

Investigators should instruct their patients to contact the site before or at the time IP is stopped. A patient that decides to discontinue IP will always be asked about the reason(s) and the presence of any AEs. Generally, AEs, SAEs, and potential endpoint events should not lead to IP discontinuation, unless there is a clear clinical rationale to do so.

The date of last intake of IP should be documented in the eCRF. All IP should be returned by the patient at their next on-site study visit or unscheduled visit. Patients permanently discontinuing IP should be given locally available standard of care therapy, at the discretion of the Investigator.

Discontinuation of IP, for any reason, does not impact on the patient´s participation in the study. The patient should continue attending subsequent study visits and data collection should continue according to the study protocol. If the patient does not agree to continue in-person study visits, a modified follow-up must be arranged to ensure the collection of endpoints and safety information. This could be a telephone contact with the patient, a contact with a relative or treating physician, or information from medical records. The approach taken should be recorded in the medical records. A patient that agrees to modified follow-up is not considered to have withdrawn from the study.

Restart of randomised IP is always encouraged. Even if a premature treatment discontinuation visit (PTDV) was completed due to discontinuation of IP, this should not prevent the patient to return to randomised IP if deemed appropriate.

7.2 Lost to follow-up

A patient will be considered potentially lost to follow-up if he or she fails to return for scheduled visits and it is not possible for the site to get contact with the patient. To optimise the chance of getting in contact with the patient during the study, Investigators should record as much contact information as possible at the start of the study including home phone, mobile phone, holiday home phone, family member phone numbers, email address, and social media contact details.

The following actions must be taken if a patient fails to return to the clinic for a required study visit:

- The site must attempt to contact the patient and reschedule the missed visit as soon as possible and counsel the patient on the importance of maintaining the assigned visit schedule.
- Before a patient is deemed potentially lost to follow up, the Investigator or designee must make every effort to regain contact with the patient or next of kin by, e.g. repeat telephone calls, certified letter to the patient´s last known mailing address or local equivalent methods. These contact attempts should be documented in the patient's medical record.
- Efforts to reach the patient should continue until the end of the study. Information regarding vital status should always be collected if possible.

7.3 Withdrawal from the study

Patients are free to withdraw from the study at any time (IP and assessments), without prejudice to further treatment. Withdrawal of consent should only occur if the patient has received appropriate information about and does not agree to any kind of further assessments or contact, including modified follow up options (see Section 7.1.2). Discontinuation of IP in itself is not considered withdrawal of consent.

Withdrawal of consent must be ascertained and documented in writing by the Investigator who must inform the AZ representative and document the withdrawal of consent in the eCRF and medical records.

A patient who withdraws from the study will always be asked about the reason(s) and the presence of any AE. The Investigator will follow up AEs reported outside of the clinical study.

If a patient withdraws from participation in the study, then his/her enrolment and randomisation codes cannot be reused. Withdrawn patients will not be replaced. If the patient withdraws consent for disclosure of future information, the sponsor may retain and continue to use any data collected before such a withdrawal of consent.

To ensure validity of study data, it is very important to collect as much data as possible throughout the study and especially vital status (dead or alive) at study closure (also for patients who have withdrawn their informed consent). The Investigator will therefore attempt to collect information on all patients' vital status from publicly available sources at study closure, even if informed consent has been withdrawn, in compliance with local privacy laws/practices.

8. STUDY ASSESSMENTS AND PROCEDURES

Study procedures and their timing are summarised in the SoA (see Section 1.1).

An Electronic Data Capture (EDC) system will be used for data collection and query handling. The Investigator will ensure that data are recorded in the eCRFs as specified in the study protocol and in accordance with the eCRF instructions provided.

The Investigator ensures the accuracy, completeness, legibility, and timeliness of the data recorded and of the provision of answers to data queries according to the Clinical Study Agreement. The Investigator will sign the completed eCRFs. A copy of the completed eCRFs will be archived at the study site.

All screening evaluations must be completed and reviewed to confirm that potential patients meet all eligibility criteria. The Investigator will maintain a screening log to record details of all patients screened and to confirm eligibility or record reasons for screening failure, as applicable.

Procedures conducted as part of the patient's routine clinical management (e.g. LVEF assessment) and obtained before signing of the ICF may be utilised for screening or baseline purposes provided the procedures met the protocol-specified criteria.

Adherence to the study design requirements, including those specified in the SoA, is essential and required for study conduct.

8.1 Enrolment Period

8.1.1 Visit 1, Enrolment (Day -21 to Day -1)

Enrolment of hospitalized patients is allowed.

At enrolment the following assessments and procedures will be completed:

- The patient signs the ICF
    - Patients who agree to the optional sampling of blood for genetic research will provide their consent
- The investigator assesses patient's eligibility criteria and reviews concomitant medications, relevant medications will be recorded in the eCRF

- The patient will be enrolled and assigned an E-code in IxRS assuming inclusion/exclusion criteria are met
- Demography and relevant medical history (including prior cardiac imaging assessments) will be recorded
- A physical examination will be conducted
- NYHA Functional Classification will be evaluated and recorded
- 12-lead ECG will be recorded
- Vital signs (BP, pulse), height and weight will be assessed and recorded
- Blood samples will be taken for NT-proBNP, creatinine (for calculation of eGFR) and HbA1c assessment (central laboratory)

8.2 Treatment period

8.2.1 Visit 2, Randomisation (Day 1)

Prior to Visit 2, the investigator will assess eligibility based on the central laboratory assessments from Visit 1. Patients not eligible will be considered screen failures and should not continue to Visit 2.

Randomisation of hospitalized patients is allowed.

At randomisation, the following assessments and procedures will be completed:

- Medical history (including cardiac imaging assessments) will be re-assessed
- A physical examination will be conducted
- A pregnancy test for women of child-bearing potential will be done locally with a dipstick provided by central laboratory with result recorded in the medical record
- Vital signs (BP, pulse) will be assessed and recorded
- NYHA Functional Classification will be evaluated and recorded
- The investigator will re-assess the inclusion and exclusion criteria
- KCCQ, PGIS and EQ-5D-5L questionnaires will be completed
- Review of concomitant medication and recording of relevant medications
- If the patient has experienced any SAEs since last visit, this will be recorded in the eCRF

- Randomisation 1:1 ratio to IP (either dapagliflozin at 10 mg or placebo) will be done in IxRS
- IP will be dispensed via IxRS to the patient. The patient will be instructed to take the IP in accordance with protocol without interruptions, and to bring all dispensed bottles to all study visits
- Patients who have consented to sampling for genetic research, will provide a blood sample

8.2.2 Visit 3 (Day 30; ±7 days):

At Visit 3, the following assessments and procedures will be conducted:

- KCCQ and PGIS questionnaires will be completed
- NYHA Functional Classification will be evaluated and recorded
- Vital signs (BP, pulse) will be assessed and recorded
- Review and updating of concomitant medication and recording of relevant medications
- Review and recording of any cardiac and HF related procedures
- Review of potential efficacy and safety events including COVID-19 testing results
- If the patient has experienced any potential endpoints, SAEs, DAEs and/or amputations, adverse events (AEs) leading to amputation and potential risk factor AEs for amputations affecting lower limbs since the last visit, this will be recorded in the eCRF
- Blood samples will be taken for creatinine (for calculation of eGFR) assessment (central laboratory)

8.2.3 Visit 4 (Day 120 ±7 days):

At Visit 4, the following assessments and procedures will be conducted:

- KCCQ and PGIS questionnaires will be completed
- NYHA Functional Classification will be evaluated and recorded
- Review and updating of concomitant medication and recording of relevant medications
- Review and recording of any cardiac and HF related procedures
- Review of potential efficacy and safety events including COVID-19 testing results

- If the patient has experienced any potential endpoints, SAEs, DAEs and/or amputations, adverse events (AEs) leading to amputation and potential risk factor AEs for amputations affecting lower limbs since the last visit, this will be recorded in the eCRF.
- Blood samples will be taken for creatinine (for calculation of eGFR) assessment (central laboratory)
- IP will be dispensed via IxRS to the patient. Drug accountability of the returned IP will be checked. The patient will be instructed to take the IP in accordance with protocol and without interruptions

8.2.4 Visit 5 (Day 240 ±7 days)

At Visit 5, the following assessments and procedures will be conducted:

- KCCQ, PGIS and EQ-5D-5L questionnaires will be completed
- NYHA Functional Classification will be evaluated and recorded
- Review and updating of concomitant medication and recording of relevant medications
- Review and recording of any cardiac and HF related procedures
- Review of potential efficacy and safety events including COVID-19 testing results
- If the patient has experienced any potential endpoints, SAEs, DAEs and/or amputations, adverse events (AEs) leading to amputation and potential risk factor AEs for amputations affecting lower limbs since the last visit, this will be recorded in the eCRF.
- IP will be dispensed via IxRS to the patient. Drug accountability of the returned IP will be checked. The patient will be instructed to take the IP in accordance with protocol and without interruptions.

8.2.5 Visit 6 (Day 360 ±7 days)

At Visit 6, the following assessments and procedures will be conducted:

- Vital signs (BP, pulse), and weight will be assessed and recorded
- Review and updating of concomitant medication and recording of relevant medications
- Review and recording of any cardiac and HF related procedures
- Review of potential efficacy and safety events including COVID-19 testing results
- If the patient has experienced any potential endpoints, SAEs, DAEs and/or amputations, adverse events (AEs) leading to amputation and potential risk factor AEs for amputations affecting lower limbs since the last visit, this will be recorded in the eCRF.

- IP will be dispensed via IxRS to the patient. Drug accountability of the returned IP will be checked. The patient will be instructed to take the IP in accordance with protocol and without interruptions.
- Blood samples will be taken for creatinine (for calculation of eGFR) assessment (central laboratory)

8.2.6 Visit 7 and onwards (Day 480 and every 120 days ±14 days)

At visit 7 and subsequent visits, the following assessments and procedures will be conducted:

- Review and updating of concomitant medication and recording of relevant medications
- Review and recording of any cardiac and HF related procedures
- Review of potential efficacy and safety events including COVID-19 testing results
- If the patient has experienced any potential endpoints, SAEs, DAEs and/or amputations, adverse events (AEs) leading to amputation and potential risk factor AEs for amputations affecting lower limbs since the last visit, this will be recorded in the eCRF.
- IP will be dispensed via IxRS to the patient. Drug accountability of the returned IP will be checked. The patient will be instructed to take the IP in accordance with protocol and without interruptions.
- Starting from Visit 6, vital signs (BP, pulse), and weight assessment as well as blood samples collection for creatinine (for calculation of eGFR) will be repeated every 12 months - on Visit 6, Visit 9 and Visit 12.

8.2.7 Premature Treatment Discontinuation Visit

Patients who prematurely and permanently discontinue treatment with IP should return for a premature treatment discontinuation visit (PTDV), which will be done as soon as possible after last dose of IP. The following assessments and procedures will be conducted:

- KCCQ, PGIS and EQ-5D-5L questionnaire will be completed
- NYHA Functional Classification will be evaluated
- Vital signs (BP, pulse) and weight will be assessed and recorded
- Review and updating of concomitant medication and recording of relevant medications
- Review and recording of any cardiac and HF related procedures

- Review of potential efficacy and safety events including COVID-19 testing results
- If the patient has experienced any potential endpoints, SAEs, DAEs and/or amputations, adverse events (AEs) leading to amputation and potential risk factor AEs for amputations affecting lower limbs since the last visit, this will be recorded in the eCRF
- Drug accountability of the returned IP will be checked

Patients who discontinue treatment prematurely should attend all study visits according to plan, including the study closure visit (SCV). Patients may re-start treatments if assessed as appropriate by the Investigator. For further details regarding discontinuations from IP, please see Section 7.1.

8.2.8 Study Closure Visit

A primary analysis censoring date (PACD) will be declared based on the rate of accrued endpoints. A study closure visit (SCV) will be scheduled within 6 weeks of the PACD. All patients (including any patients who have discontinued treatment with IP) should return for this visit.

The patient will stop taking IP at the SCV. Remaining IP will be collected at that time and drug accountability will be checked. The following assessments and procedures will be conducted:

- KCCQ, PGIS and EQ-5D-5L questionnaire will be completed
- NYHA Functional Classification will be evaluated
- A physical examination will be conducted
- Vital signs (BP, pulse) and weight will be assessed and recorded.
- Review and updating of concomitant medication and recording of relevant medications
- Review and recording of any cardiac and HF related procedures
- Review of potential efficacy and safety events including COVID-19 testing results
- If the patient has experienced any potential endpoints, SAEs, DAEs and/or amputations, adverse events (AEs) leading to amputation and potential risk factor AEs for amputations affecting lower limbs since the last visit, this will be recorded in the eCRF
- Drug accountability of the returned IP will be checked

8.2.9 Unscheduled visits

An unscheduled on-site or telephone visit may occur in-between scheduled on-site visits (for example assessment of potential endpoint events or safety events).

8.3 Efficacy assessments

8.3.1 Efficacy event capture

Efficacy events (i.e. death, hospitalisation or urgent visits for HF) will be collected by site personnel according to the study visit schedule. All potential efficacy events should be recorded as an AE and on additional event modules in the eCRF. If the potential efficacy event fulfils SAE criteria (see Appendix B 2) the site is to record and report these events to the sponsor or designee within timelines described in Section 8.6.

NYHA classification will be done by the Investigators and recorded in the eCRF. PROs will be collected for all patients throughout the study period via a hand-held electronic device. All-cause hospitalisations will be derived from SAE reports.

8.3.2 Efficacy event adjudication

A Clinical Events Adjudication (CEA) Committee will be established for this trial and adjudicate primary efficacy events in accordance with adjudication criteria detailed in the CEA charter.

Events to be adjudicated include components of the primary efficacy endpoint: deaths, hospitalisation for HF, and urgent HF visits. All deaths will be adjudicated to determine if they are CV or non-CV deaths. All adjudication will be done on an ongoing basis throughout the trial.

8.3.3 Clinical Outcome Assessments (COA)

A COA is any assessment that may be influenced by human choices, judgement, or motivation and may support either direct or indirect evidence of treatment benefit. Patient Reported Outcomes (PROs) is one of the types of COAs. A PRO is any report of the status of a patient's health condition that comes directly from the patient, without interpretation of anyone else. PROs have become a significant endpoint when evaluating benefit/risk of treatments in clinical trials. The following PROs will be collected: KCCQ, PGIS and EQ-5D-5L (see Appendix I, Appendix K, Appendix L). PROs will be collected for all patients throughout the study period via a hand-held electronic device. See study of assessment (See Table1) for the timing of collection. The ePRO devices should be administered prior to first dose at visit 2/randomisation. Site staff should stress that the information is confidential.

8.3.3.1 KCCQ

The Kansas City Cardiomyopathy Questionnaire (KCCQ) is a 23-item, self-administered disease specific instrument and has shown to be a valid, reliable and responsive measure for patients with HF (Greene et al 2018, Spertus et al 2005). The KCCQ was developed to independently measure the patient's perception of their health status, which includes heart failure-related symptoms (frequency, severity and recent change), impact on physical and social function, self- efficacy and knowledge, and how their heart failure impacts their quality of life (QOL). Scores are transformed to a range of 0-100. Higher scores represent a better outcome.

The KCCQ tool quantifies the following six (6) distinct domains and two (2) summary scores:

- KCCQ Symptom Domain quantifies the frequency and burden of clinical symptoms in heart failure, including fatigue, shortness of breath, paroxysmal nocturnal dyspnea and patients' edema/swelling. An overall symptom score is generally used in analyses; subscale scores for both frequency and severity are also available. The total symptom Score incorporates the symptom domains into a single score
- KCCQ Physical Function Domain measures the limitations patients experience, due to their heart failure symptoms, in performing routine activities. Activities are common, gender-neutral, and generalizable across cultures, while also capturing a range of exertional requirements
- KCCQ Quality of Life Domain is designed to reflect patients' assessment of their quality of life, given the current status of their heart failure
- KCCQ Social Limitation Domain quantifies the extent to which heart failure symptoms impair patients' ability to interact in a number of gender-neutral social activities
- KCCQ Self-efficacy Domain quantifies patients' perceptions of how to prevent heart failure exacerbations and manage complications when they arise. This scale is not included in the summary scores
- KCCQ Symptom Stability Domain measures recent changes in patients' symptoms; their shortness of breath, fatigue or swelling. It compares patients frequency of heart failure symptoms at the time of completing the KCCQ with their frequency 2 weeks ago. As a measure of change, it is most interpretable as a baseline assessment of the stability of patients' symptoms at the start of a study and shortly thereafter, as a measure of the acute response to treatment. This domain is not included in the summary scores.
- Clinical Summary Score includes total symptom and physical function scores to correspond with NYHA Classification
- Overall Summary Score includes the total symptom, physical function, social limitations and quality of life scores

8.3.3.2 Patient Global Impression of Severity (PGIS)

The PGIS item is included to assess how a patient perceives his/her overall current severity of heart failure symptoms. Patients will choose from response options from "no symptoms" to "very severe".

8.3.3.3 EuroQoL five-dimensional five-level questionnaire (EQ-5D-5L)

The EQ-5D-5L is a self-reported questionnaire that is used to derive a standardized measure of health status, also referred to as a utility score. EQ-5D-5L utility scores are widely accepted by reimbursement authorities and will be used to support health economic evaluations.

8.3.3.4 Administration of electronic PROs

Each site must allocate the responsibility for the administration of the ePROs to a specific individual and, if possible, assign a backup person to cover if that individual is absent. A key aspect of study success is to have high PRO compliance. Therefore, it is essential to follow SoA and that sites make sure the device is charged and fully functional at all times in order to minimize missing data.

It is important that the site staff explains the value and relevance of PRO data: to hear directly from patients how they feel. The following best practice guidelines should be followed:

- Patient must not receive help from relatives, friends, or site personnel to answer or clarify the PRO questionnaires in order to avoid bias. If a patient uses visual aids (e.g., spectacles or contact lenses) for reading and does not have them at hand, the patient will be exempted from completing the PROs questionnaires on that visit
- Before any other study procedures are conducted at a given visit (except the Visit 2: eligibility confirmation before the KCCQ)
- Before being seen by the investigator
- PRO questionnaires must be completed by the patient in private
- The appointed site personnel should also stress that the information is confidential. Therefore, if the patient has any medical problems, he or she should discuss them with the doctor or research nurse separately from the ePRO assessment
- The appointed site personnel must show patients how to use the ePRO device, in accordance with the instructions provided
- The appointed site personnel should remind patients that there are no right or wrong answers, and the patient should be given sufficient time to complete the PRO questionnaires at his/her own speed

If a site is affected by COVID-19 pandemic and on-site visits are not possible, phone collection of ePRO is an alternative solution to maintain continuity of the assessments. The details of the procedure will be provided in a separate instruction.

If the patient is unable to read the questionnaire (e.g., is blind or illiterate), the patient will be exempted from completing the PRO questionnaires and may still participate in the study. Patients exempted in this regard should be flagged appropriately by the site personnel.

8.4 Safety assessment

Planned time points for all safety assessments are provided in the schedule of activities (Table 1).

8.4.1 Physical examinations

A physical examination will be performed at the time-points specified in Table 1 and include an assessment of the following: general appearance, respiratory and cardiovascular systems (including oedema) and abdomen.

The assessment dates will be recorded in the eCRF.

8.4.2 Vital Signs

- Pulse and BP will be measured twice at all applicable visits, and all measurements will be recorded in the eCRF.
- The measurements should be done before any blood sampling. The measurements will be assessed in a sitting position with a completely automated device. Manual techniques will be used only if an automated device is not available.
- The measurements should be preceded by at least 5 minutes of rest for the subject in a quiet setting without distractions (e.g., television, cell phones).

8.4.3 Electrocardiogram

A 12-lead ECG (standard ECG with a paper speed of 25-50 mm/second covering at least 6 sequential beats) will be recorded at baseline (Visit 1) after the patient has been lying down to rest for at least 5 minutes, to confirm presence or absence of atrial fibrillation/flutter at enrolment. Heart rate and heart rhythm will be reported in the eCRF. The baseline ECG should be stored and be made available upon request for adjudication purposes

8.4.4 Safety laboratory assessments

Serum creatinine will be collected for calculation of eGFR using CKD-EPI equation (Levey et al 2009).

8.4.5 Other safety assessments

If COVID-19 testing was done, the type of test and result (positive/negative) should be recorded in the eCRF.

8.4.6 Other clinical assessments

8.4.6.1 Body weight and height

The patient's body weight will be measured with light clothing and no shoes. If the patient has a prosthetic limb, this should be consistently worn during all weight measurements. The patient's height will be measured at Visit 1, with no shoes. The weight and height will be recorded in the eCRF.

8.5 Collection of adverse events

The Principal Investigator is responsible for ensuring that all staff involved in the study are familiar with the content of this section.

The definitions of an AE or SAE can be found in Appendix B.

AE will be reported by the patient (or, when appropriate, by a caregiver, surrogate, or the patient's legally authorised representative).

The Investigator and any designees are responsible for detecting, documenting, and recording events that meet the definition of a SAEs and DAEs, amputation and events potentially placing the patient at risk for a lower limb amputation (preceding events). For information on how to follow-up AEs see Section 8.5.3.

8.5.1 Method of detecting AEs and SAEs

Care will be taken not to introduce bias when detecting SAEs or DAEs. Open-ended and non-leading verbal questioning of the patient is the preferred method to inquire about AE occurrences.

Safety information on SAEs and DAEs, amputations, adverse events (AEs) leading to amputation and potential risk factor AEs for amputations affecting lower limbs will be collected and entered into eCRFs by site personnel according to the study visit schedule.

If the potential efficacy event fulfils SAE criteria (see Appendix B 2) the site is to record and report these events to the Sponsor or designee within timelines described in Section 8.6.1.

8.5.1.1 Adverse events (AEs) leading to amputation and potential risk factor AEs for amputations affecting lower limbs (“preceding events”)

To ensure that data on amputations is systematically collected, amputations and underlying conditions relevant to amputation will be recorded on a specific eCRF page. The adverse event leading to amputation should be recorded in the eCRF as AE/SAE.

In addition to amputation, non-serious and serious events potentially placing the patient at risk for a lower limb amputation (“preceding events”) should also be recorded in the eCRF as AE/SAE whether or not an amputation has taken place. The lower limb amputation “preceding events” of interest include diabetic foot related conditions, vascular, volume depletion, wounds/injury/trauma, infection and neuropathy. If any of these or other potentially relevant events have occurred, relevant information must be provided (this will be collected on a dedicated eCRF page - for details see eCRF instruction)”.

8.5.1.2 Capture of DKA events

For SAEs or DAEs reported by the Investigator as Diabetic Ketoacidosis (DKA - see definition below) additional information will be recorded on specific eCRF pages in addition to the AE/SAE form. All potential events of DKA will be submitted to an independent DKA Adjudication Committee, see Section 8.5.1.2.2)

8.5.1.2.1 DKA definition

A diagnosis of Diabetic Ketoacidosis should only be made in a clinical setting consistent with DKA (based on patient history, symptoms, and physical exam) and in the absence of more likely alternative diagnoses and causes of acidosis (such as lactic acidosis). The following biochemical data should support diagnosis:

- Ketonaemia ≥3.0 mmol/L and/or significant ketonuria (more than 2+ on standard urine sticks)
- At least one of the following criteria suggesting high anion gap metabolic acidosis:
  - Arterial or Venous pH ≤7.3
  - Serum bicarbonate ≤18 mEq/L
  - Anion gap [Na – (Cl + HCO3)] >10

8.5.1.2.2 Diabetic Ketoacidosis Adjudication Committee T2D

All potential events of DKA will be submitted to an independent DKA Adjudication Committee. The committee will be kept blinded to the treatment codes. A separate DKA Adjudication Manual will define and describe the procedures for the collection of DKA information, handling, adjudication criteria and reporting of these events.

8.5.1.3 Capture of cardiac ischaemic events and stroke

For myocardial infarctions, unstable angina and stroke additional information will be recorded on specific eCRF pages in addition to the AE/SAE form.

The diagnosis of stroke, MI and unstable angina should be made according to standard clinical practice and align with the definition for stroke in the standardised definitions for endpoints (Hicks et al 2018) described in Appendix C.

8.5.1.4 Capture of Major hypoglycaemic events

A major hypoglycemic event is defined as an event that is characterized by altered mental and/or physical status, any symptoms of severe impairment in consciousness or behavior, that require external assistance of another person for treatment of hypoglycemia and recovery, to actively administer carbohydrates, glucagon, or take other corrective actions.

Plasma glucose concentrations may not be available during a hypoglycaemic event, but neurological recovery following the corrective actions is considered sufficient evidence that the event was induced by a low plasma glucose concentration. Major hypoglycaemic episodes will be recorded in the eCRF as an AE and on an additional eCRF page.

8.5.1.5 Capture of additional laboratory values

Any additional safety laboratory assessments during the study period, including creatinine, will be obtained per the Investigator's medical judgment in the course of standard care using local laboratories. Laboratory values would be recorded only on SAE eCRFs as part of narrative information, per the Investigator's judgment.

8.5.2 Time period and frequency for collecting AE and SAE information

Non-serious adverse events as defined per protocol will be collected from randomisation (Visit 2), throughout the treatment period until and including the patient's last visit (the study closure visit). Serious adverse events are recorded from the time of signing of informed consent form throughout the treatment period until and including the patient's last visit.

All SAEs will be recorded and reported to the sponsor or designee within 24 hours, as indicated in Appendix B. The Investigator will submit any updated SAE data to the sponsor within 24 hours of it being available.

The method of recording, evaluating, and assessing causality of AE and SAE and the procedures for completing and transmitting SAE reports are provided in Appendix B.

8.5.3 Follow-up of AEs and SAEs

After the initial AE/SAE report, the Investigator is required to proactively follow each patient at subsequent visits/contacts. All SAE and events of amputation and potential preceding events will be followed until resolution, stabilization, the event is otherwise explained, or the patient is lost to follow-up.

Any AEs that are unresolved at the patient's last visit in the study are followed up by the Investigator for as long as medically indicated, but without further recording in the CRF. AstraZeneca retains the right to request additional information for any patient with ongoing AE(s)/SAE(s) at the end of the study, if judged necessary.

8.5.4 Adverse event data collection

The following variables will be collect for each AE;

- AE (verbatim)
- The date when the AE started and stopped
- Maximum intensity (mild/moderate/severe)
- Whether the AE is serious or not
- Investigator causality rating against the Investigational Product(s) (yes or no)
- Action taken with regard to IP
- Outcome

In addition, the following variables will be collected for SAEs:

- Date AE met criteria for serious AE
- Date Investigator became aware of serious AE
- AE is serious due to
- Date of hospitalisation
- Date of discharge
- Probable cause of death
- Date of death
- Autopsy performed
- Causality assessment in relation to Study procedure(s) and/or other medication
- Description of AE

8.5.5 Causality collection

The Investigator will assess causal relationship between the IP and each AE and answer 'yes' or 'no' to the question 'Do you consider that there is a reasonable possibility that the event may have been caused by the investigational product?'

For SAEs, causal relationship will also be assessed for other medication and study procedures. Note that for SAEs that could be associated with any study procedure the causal relationship is implied as 'yes'.

A guide to the interpretation of the causality question is found in Appendix B to the Clinical Study Protocol.

8.5.6 Adverse events based on signs and symptoms

All AEs spontaneously reported by the patient or reported in response to the open question from the study personnel: 'Have you had any health problems since the previous visit/you were last asked?' or revealed by observation will be collected and recorded in the eCRF if they fulfil the criteria specified in Section 8.5.2. When collecting AEs, the recording of diagnoses is preferred (when possible) to recording a list of signs and symptoms. However, if a diagnosis is known and there are other signs or symptoms that are not generally part of the diagnosis, the diagnosis and each sign or symptom will be recorded separately.

8.5.7 Adverse events based on examinations and tests

The results from the Clinical Study Protocol mandated vital signs and laboratory values will be summarised in the clinical study report. Deterioration as compared with baseline in protocol-mandated vital signs should therefore only be reported as AEs if they fulfil any of the SAE criteria or are the reason for discontinuation of treatment with the IP. If deterioration in a vital sign is associated with clinical signs and symptoms, the sign or symptom will be reported as an AE if they

fulfil any of the SAE criteria or are the reason for discontinuation of treatment of IP, and the associated vital sign will be considered as additional information.

8.5.8 Disease-under study (DUS) (not applicable)

8.5.9 Disease progression (not applicable)

8.6 Safety reporting and medical management

8.6.1 Reporting of serious adverse events

All SAEs have to be reported, whether or not considered causally related to the investigational product, or to the study procedure(s). All SAEs will be recorded in the eCRF.

If any SAE occurs in the course of the study, then Investigators or other site personnel inform the appropriate AstraZeneca representatives within one day i.e., immediately but **no later than 24 hours** of when he or she becomes aware of it.

The designated AstraZeneca representative works with the Investigator to ensure that all the necessary information is provided to the AstraZeneca Patient Safety data entry site **within 1 calendar day** of initial receipt for fatal and life-threatening events **and within 5 calendar days** of initial receipt for all other SAEs.

For fatal or life-threatening adverse events where important or relevant information is missing, active follow-up is undertaken immediately. Investigators or other site personnel inform AstraZeneca representatives of any follow-up information on a previously reported SAE within one calendar day i.e., immediately but **no later than 24 hours** of when he or she becomes aware of it.

Once the Investigators or other site personnel indicate an AE is serious in the EDC system, an automated email alert is sent to the designated AstraZeneca representative.

If the EDC system is not available, then the Investigator or other study site staff reports a SAE to the appropriate AstraZeneca representative by telephone.

The AstraZeneca representative will advise the Investigator/study site staff how to proceed.

Investigators or other site personnel send relevant CRF modules by fax to the designated AstraZeneca representative.

For further guidance on the definition of a SAE, see Appendix B of the Clinical Study Protocol.

8.6.1.1 Reporting of SAEs considered to be potential endpoints

In order to avoid unnecessary unblinding of efficacy endpoint events, certain SAEs which are also potential endpoints (i.e., fatal AEs and HF events) will not be reported to health authorities. Clinical data for the above mentioned events will be recorded as AEs/SAEs as well as on separate event forms in the eCRF. Recording of a suspected endpoint should be done within the same timeframes as defined for SAEs (see Section 8.6.1).

In addition, fatal AEs and potential HF endpoints will be centrally adjudicated by an independent CEA committee (see Section 8.3.1 and 8.3.2). If adjudication confirms the endpoint, the SAE will not be reported to health authorities. However, if it is determined by the CEA committee that a potential endpoint does not meet the endpoint criteria, the event will be reported (according to the timelines specified in Section 8.6.1) to AZ patient safety data entry site and if applicable to the health authorities (note that the clock starts when the adjudication results are available).

8.6.2 Pregnancy

All pregnancies and outcomes of pregnancy should be reported to AstraZeneca except if the pregnancy is discovered before the study patient has received any IP. If a pregnancy is reported, the Investigator should inform the sponsor within 24 hours of learning of the pregnancy. Abnormal pregnancy outcomes (e.g. spontaneous abortion, foetal death, stillbirth, congenital anomalies, ectopic pregnancy) are considered SAEs.

8.6.2.1 Maternal exposure

Women of childbearing potential who are not using contraception as defined in Section 5.2; exclusion criterion number 18 are not allowed to be included in this study. Should a pregnancy still occur, the investigational product should be discontinued immediately and the pregnancy reported to AstraZeneca.

Dapagliflozin must not be used in the second and third trimesters of pregnancy. In the time period corresponding to second and third trimester of pregnancy with respect to human renal maturation, maternal exposure to dapagliflozin in rat studies was associated with increased incidence and/or severity of renal pelvic and tubular dilatations in progeny.

There are no adequate and well-controlled studies of dapagliflozin in pregnant women. When pregnancy is detected, investigational product(s) should be discontinued.

8.6.3 Overdose

Dapagliflozin has been well tolerated at doses of up to 500 mg/day in single dose testing in healthy volunteers and up to 100 mg/day in repeat dose testing for 14 days in healthy volunteers and patients with T2D. Suspected single intake of more than 50 tablets of 10 mg dapagliflozin tablets or repeated intake of more than 10 tablets of 10 mg dapagliflozin tablets should be reported on the eCRF overdose module. If an overdose is suspected, monitoring of vital functions as well as treatment should be performed as appropriate.

For further information regarding overdose, refer to the IB.

- An overdose without associated symptoms is only recorded on the Overdose eCRF module
- An overdose with associated AEs is recorded as the AE diagnosis/symptoms on the relevant AE modules in the eCRF and on the Overdose eCRF module

If an overdose on an AstraZeneca IP occurs in the course of the study, then the investigator or other site personnel inform appropriate AstraZeneca representatives immediately, or no later than 24 hours of when he or she becomes aware of it.

The designated AstraZeneca representative works with the investigator to ensure that all relevant information is provided to the AstraZeneca Patient Safety data entry site.

For overdoses associated with a SAE, the standard reporting timelines apply, see Section 8.5.2. For other overdoses, reporting must occur within 30 days.

8.7 Pharmacokinetics (not applicable)

8.8 Pharmacodynamics (not applicable)

8.9 Optional exploratory genetics

Approximately 6 mL blood sample for DNA isolation will be collected from subjects who have consented to participate in the genetic analysis component of the study. Participation is optional. Subjects who do not wish to participate in the genetic research may still participate in the study.

See Appendix D for Information regarding genetic research. Details on processes for collection and shipment and destruction of these samples can be found in Appendix D.

8.10 Biomarkers (not applicable)

8.11 Health Economics (not applicable)

9. STATISTICAL CONSIDERATIONS

9.1 Statistical hypotheses

For the primary and secondary endpoints, the following hypothesis will be tested at the 4.980 % 2-sided level:

H0: HR [dapagliflozin:placebo] =1

versus

H1: HR [dapagliflozin:placebo] ≠1

9.2 Sample size determination

The primary objective of the study is to determine the superiority of dapagliflozin versus placebo added to standard of care in reducing the composite of CV death and heart failure events (hospitalisation for HF or urgent HF visit). Two hypotheses will be tested simultaneously (i.e., dual primary analyses) for the primary objective: (1) in the full population and in (2) an LVEF <60% subpopulation, with alpha allocated to each test. Originally, assuming a true hazard ratio (HR) of 0.80 between dapagliflozin and placebo, using a two-sided alpha of 5%, 844 primary endpoint

events were targeted in order to provide a statistical power of 90% for the test of the primary composite endpoint. To allow testing for the dual primary analyses, alpha will be allocated to each test to ensure strong control of the overall type I error rate. The target number of patients with a primary endpoint has been increased to 1117 in order to provide adequate statistical power for each test. It is anticipated that at least 70% of the events (i.e., approximately 780 events) will be available for the LVEF <60% subpopulation. To illustrate, assuming a true HR of 0.80, a two-sided alpha of 2.4% allocated to the LVEF <60% subpopulation will result in a power of 80% to detect a treatment difference, whereas an alpha allocation of 1.5% to the full population will result in 90% power. The final alpha split for the dual primary analyses will be specified in the SAP prior to the planned interim analysis. This is based on an overall 1:1 allocation between dapagliflozin and placebo.

The HR 0.80 was originally chosen as a conservative assumption based on the observed HR 0.72 (95% confidence interval 0.50-1.04) for the composite of HF hospitalisation and CV death in patients with HF at baseline in the EMPA- REG OUTCOME trial (Fitchett et al 2016) and HR 0.61 (0.46-0.80) for patients with history of HF in the CANVAS program (Rådholm et al 2018) considering that these were post-hoc analyses in subgroups with limited documentation of baseline HF diagnosis, not characterised by ejection fraction.

The event rate assumptions are based on sub analyses of the TOPCAT and I-PRESERVE studies by geographic region, NT-proBNP levels, prior hospitalisation for HF, and T2D status (Pfeffer et al 2015, Kristensen et al 2015, Kristensen et al 2017). The original sample size calculation (~ 4700 randomized patients) built on the assumption of an annual event rate of 9% in the placebo group for the majority of prevalent HFpEF patients, importantly all with NT -proBNP ≥300 pg/ml by inclusion criterion. Additionally, a subgroup of patients due to be discharged or recently discharged from a HF hospitalisation (here denoted 'subacute' patients) with a higher event rate is planned to be included. Assuming 20% of patients from the subacute category with an annual event rate of 24% during the first year and 9% thereafter for the remainder of the study, the original sample size of 4700 patients was estimated to provide the required number of 844 patients with a primary event during a recruitment period of 18 months and a minimum follow-up period of 15 months.

Based on the ongoing blinded monitoring of event accrual (including the percentage of patients from the subacute category), the sample size is increased from original 4700 to approximately 6100 randomised patients. Accordingly, the recruitment period is anticipated to increase from the original 18 months to 26 months. Recruitment might be marginally prolonged in a few countries to meet local targets. The study is event driven and the number of patients or duration may further change.

With the same event rate assumptions as above, assuming 11% of patients from the subacute category, approximately 6100 patients are estimated to provide the required number of 1117 patients with a primary event during an anticipated recruitment period of 26 months and a minimum follow-up period of 13.5 months (total study duration 39 months).

In addition, the expected number of patients who will be lost to follow-up is expected to be small; hence, these are not considered in the determination of the sample size.

9.3 Populations for analyses

For purposes of analysis, the following populations are defined:

Table 5 Population for analysis

Population	Description
Enrolled	All patients who sign the ICF
Full Analysis Set (FAS)	All patients who have been randomised to study treatment, irrespective of their protocol adherence and continued participation in the study. Patients will be analysed according to their randomised investigational product assignment, irrespective of the treatment actually received. The FAS will be considered the primary analysis set for the intention to treat analysis of primary and secondary variables. A subset of the full analysis set consisting of patients with baseline LVEF of <60% (or LVEF <60% subpopulation) will be analysed separately as part of the confirmatory statistical testing procedure.
Safety analysis set	All patients randomly assigned to Study treatment and who take at least 1 dose of investigational product. Patients will be analysed according to the treatment actually received. The Safety analysis set will be considered the primary analysis set for all safety variables

9.4 Statistical analyses

All personnel involved with the analysis of the study will remain blinded until database lock and Clinical Study Protocol deviations identified.

Analyses will be performed by AstraZeneca or its representatives.

A comprehensive statistical analysis plan (SAP) will be developed prior to first patient randomised and any subsequent amendments will be documented, with final amendments finalised before database lock. This section is a summary of the planned statistical analyses of the primary and secondary endpoints. Any deviations from this plan will be reported in the clinical study report.

9.4.1 Efficacy analyses

9.4.1.1 Analysis of the primary variable

The primary variable is the time from randomisation to first event included in the primary composite endpoint. Two hypotheses will be tested simultaneously (i.e., dual primary endpoint analyses): (1) in the full population and (2) in an LVEF <60% subpopulation, with alpha allocated to each test. The primary analysis will be based on the ITT principle using the FAS, including events occurring on or prior to the PACD, adjudicated by the CEA committee.

In the analysis of the primary composite endpoint, treatments (dapagliflozin versus placebo) will be compared using a Cox proportional hazards model with a factor for treatment group, stratified by T2D status at randomisation. The p-value, HR and 95% confidence interval will be reported.

The contribution of each component of the primary composite endpoint to the overall treatment effect will be examined. Methods similar to those described for the primary analysis will be used to separately analyse the time from randomisation to the first occurrence of each component of the primary composite endpoint. HR and 95% confidence intervals will be reported.

Kaplan-Meier estimates of the cumulative incidence to the first occurrence of any event in the primary endpoint will be calculated and plotted, for overall analysis and for the individual components.

9.4.1.2 Analysis of the secondary variables

The outcome of all HF events (first and recurring) and CV death will be analysed by the semi-parametric proportional rates model (Lin et al 2000) to test the treatment effect and to quantify the treatment difference. The rate ratio and its 95% confidence interval and corresponding two-sided p-value will be presented. This outcome will also be analysed for the LVEF <60% subpopulation within the multiple testing procedure as described in Section 9.2.

The analysis of change from baseline for KCCQ total symptom score at 8 months will be further detailed in the statistical analysis plan, e.g. with consideration of handling of patients who die. In addition to the secondary endpoint, total symptom score, the overall summary score, clinical summary score and domain scores will be analysed. A responder analysis will also be performed (more details presented in the SAP).

The analysis of the endpoints time from randomisation to CV death and time to all-cause mortality will be analysed in the similar manner as the primary variable.

9.4.1.3 Subgroup analysis

Subgroup variables for the primary efficacy endpoint include demography, baseline disease characteristics, baseline concomitant medications and others. Cox proportional hazard model stratified for T2D with factors for treatment group, the subgroup variable and the interaction between treatment and subgroup will be used to examine treatment effects within relevant subgroups separately. A test of interaction between randomised treatment group and the subgroup variable will be performed in each Cox model. The p-values for the subgroup analyses will not be adjusted for multiple comparisons as the tests are exploratory and will be interpreted descriptively. Treatment differences with 95% confidence intervals will be reported for each subgroup. HRs and CIs for overall analysis and subgroups will be presented with forest plots as well. Further details of the subgroup analysis, including the list of subgroup variables, will be provided in the SAP.

9.4.1.4 Sensitivity analysis

Details of the sensitivity analysis for the primary and secondary endpoints will be provided in the SAP.

9.4.2 Safety analyses

All safety analyses will be performed on the Safety analysis set. The number and percent of patients with SAEs, DAEs, amputations, and potential preceding events for lower limb amputations will be summarised by treatment group, and by system organ class and preferred term.

For safety analyses, summaries will be provided using both on treatment observations and using all observations regardless of whether patients are on or off study treatment.

9.4.3 Methods for multiplicity control

A closed testing procedure including a pre-specified hierarchical ordering of the primary and secondary endpoints will be utilised. The Type I error will be controlled at an overall two-sided 5% level for multiplicity across primary and secondary endpoints and in consideration of the planned interim analysis. With one interim analysis at 67% of events (see Section 9.5) the two-sided significance level in final analysis, α, will be 4.980%. Statistical significance will be assessed in two branches in a pre-specified order of the endpoints and populations which is further described in the SAP. The significance level α, will be split for the two primary analyses, denoted α_1 and α_2. If either of the tests of the primary endpoint in the full study population and for LVEF <60% subpopulation is significant at respective levels α_1 and α_2, the next hypothesis in the respective branch sequence will be tested at the same significance level. The exact split of alpha will be documented in an updated SAP before the interim analysis. If all hypotheses in one arm are rejected, the alpha will be recycled to the other branch.

9.5 Interim analyses

An interim analysis is planned to be performed including approximately 67% of the target number of patients with adjudicated primary endpoint events (approximately 748 events). There will in principle be one planned interim analysis for efficacy, with the possibility of the DMC to conduct subsequent interim analysis if they deem necessary. The significance level for final analysis will be determined by the Haybittle-Peto function based on the actual number of interim analyses. The interim analysis will assess superiority of dapagliflozin to placebo. The interim analysis will have a nominal two-sided alpha level of 0.2%. At the interim analysis, the primary composite endpoint will be tested in the full study population at the specified alpha level. If superiority is achieved for the primary endpoint, then the superiority of dapagliflozin to placebo on CV deaths will be tested in the full study population at a two-sided level of 0.2%. If CV death is significant, then an action is triggered whereby the DMC will evaluate the totality of the efficacy data and safety data, to determine if benefit is unequivocal and overwhelming such that the DMC recommends ending the study.

In the initial version of the protocol, there was a planned futility analysis to be performed at the time of interim analysis. This futility analysis was removed, since formal testing was updated to

include both the LVEF <60% subpopulation and full study population, and that this potentially creates complex scenarios related to futility and benefit in one, other or both populations.

9.5.1 Data monitoring committee (DMC)

An independent data monitoring committee (DMC) will be appointed and will report to the Executive Committee. The DMC will be responsible for safeguarding the interests of the patients in the outcome study by assessing the safety of the intervention during the study. The DMC will have access to the individual treatment codes and be able to merge these with the collected study data while the study is ongoing. A charter will be prepared to detail precise roles and responsibilities and procedures of the DMC.

10. REFERENCES

Ambrosy et al 2014
Ambrosy AP, Gheorghiade M, Chioncel O, Mentz RJ, Butler J. Global perspectives in hospitalised heart failure: regional and ethnic variation in patient characteristics, management, and outcomes. Curr Heart Fail Rep 2014 Dec;11(4):416-27.

Borlaug and Paulus 2011
Borlaug BA, Paulus WJ. Heart failure with preserved ejection fraction: pathophysiology, diagnosis, and treatment. Eur Heart J 2011;32:670-9.

Braunwald 2015
Braunwald E. The war against heart failure: the Lancet lecture. Lancet 2015;385:812-24.

Butler et al 2014
Butler J, Fonarow GC, Zile MR, Lam CS, Roessig L, Schelbert EB, et al. Developing therapies for heart failure with preserved ejection fraction: current state and future directions. JACC Heart Fail 2014 Apr;2(2):97-112.

Cherney et al 2014
Cherney DZ, Perkins BA, Soleymanlou N, Har R, Fagan N, Johansen OE, et al. The effect of the empagliflozin on arterial stiffness and heart rate variability in subjects with uncomplicated type 1 diabetes mellitus. Cardiovasc Diabetol 2014;13:28.

Chilton et al 2015
Chilton R, Tikkanen I, Cannon CP, Crowe S, Woerle HJ, Broedl UC, et al. Effects of empagliflozin on blood pressure and markers of arterial stiffness and vascular resistance in patients with type 2 diabetes. Diabetes Obes Metabo 2015;17:1180-93.

Cook et al 2014
Cook C, Cole G, Asaria P, Jabbour R, Francis DP. The annual global economic burden of heart failure. Int J Cardiol 2014 Feb 15;171(3):368-76.

EMA Guidance 2016
EMA 2016; Guideline on clinical investigation of medicinal products for the treatment of chronic heart failure (draft), Committee for Medicinal Products for Human Use (CHMP). EMA/CHMP/392958/2015.

FDA Draft Guidance for Industry 2016
Food and Drug Administration. Use of real-world evidence to support regulatory decision-making for medical devices. Draft Guidance for Industry and Food and Drug Administration Staff. 16 September 2016.

Fitchett et al 2016

Fitchett D, Zinman B, Wanner C, Lachin JM, Hantel S, Salsali A, et al. Heart failure outcomes with empagliflozin in patients with type 2 diabetes at high cardiovascular risk: results of the EMPA-REG OUTCOME® trial. Eur Heart J 2016;14;37(19):1526-34.

Greene et al 2018

Greene SJ, Mentz RJ, Felker GM. Outpatient Worsening Heart Failure as a Target for Therapy: A Review. JAMA Cardiol. 2018 Jan 31. doi:10.1001/jamacardio.2017.5250. [Epub ahead of print] PubMed PMID: 29387880.

Hicks et al 2014

Hicks KA, Hung HMJ, Mahaffey KW, Mehran R, Nissen SE et al, on behalf of the Standardized Data Collection for Cardiovascular Trials Initiative. Standardized Definitions for Cardiovascular and Stroke Endpoint Events in Clinical Trials. Draft Definitions for CDISC August 20, 2014.

Hicks et al 2018

Hicks KA, Mahaffey KW, Mehran R, Nissen SE, et alCardiovascular and Stroke Endpoint Definitions for Clinical Trials. J Am Coll Cardiol 2018;71:1021–34

Inzucchi et al 2012

Inzucchi SE, Bergenstal RM, Buse J.B, Diamant M, Ferrannini E, Nauck M et al. Management of hyperglycaemia in type 2 diabetes: a patient -centered approach. Position statementof the American Diabetes Association (ADA) and the European Association for the Study of Diabetes (EASD). Diabetes Care. 2012 Jun;35(6):1364-79.

Inzucchi et al 2015

Inzucchi Silvio E Bergenstal Richard M, Buse John B, Diamant Michaela, Ferrannini Ele, Nauck Michael, Peters Anne L, Tsapas Apostolos, Wender Richard,and Matthews David R. Management of Hyperglycemia in Type 2 Diabetes, 2015: A Patient-Centered Approach. Update to a Position Statement of the American Diabetes Association and the European Association for the Study of Diabetes. Diabetes Care 2015;38:140–149

Iwaz et al 2016

Iwaz JA, Le E, Aramin H, Romero D, Iqbal N, Kawahara M, et al. New targets in the drug treatment of heart failure. Drugs 2016;79:187-201.

Kosiborod et al 2016

Kosiborod M, Xu J, Sjostrand M, Sjostrom CD Safety and Efficacy of Dapagliflozin in Combination with Potassium-sparing Agents. Abstr, 1094-P ADA, June 2016, New Orleans

Kosiborod et al 2017a

Kosiborod M, Cavender MA, Fu AZ, Wilding JP, Khunti K, Holl RW, et al, on behalf of the CVD-REAL Investigators and Study Group. Lower risk of heart failure and death in patients initiated on SGLT-2 inhibitors versus other glucose-lowering drugs: the CVD-REAL study. Circulation 2017 May 18. pii: CIRCULATIONAHA.117.029190. doi: 10.1161/CIRCULATIONAHA.117.029190.

Kosiborod et al 2017b

Kosiborod M, Gause-Nilsson I, J Xu J, Sonesson C, Johnsson E. Efficacy and safety of and dapagliflozin in patients with type 2 diabetes mellitus concomitant heart failure. J Diabetes Complications 2017; 31 (7):1215-21.

Kristensen et al 2015
Kristensen SL, Jhund PS, Køber L, McKelvie RS, Zile MR, Anand IS, et al. Relative importance of history of heart failure hospitalisation and N-terminal Pro–B-type natriuretic peptide level as predictors of outcomes in patients With heart failure and preserved ejection fraction. JACC Heart Fail 2015 Jun;3(6):478-86.

Kristensen et al 2016
Kristensen SL, Preiss D, Jhund PS, Squire I, Cardoso JS et al. Risk related to pre–diabetes mellitus and diabetes mellitus in heart failure with reduced ejection fraction insights from prospective comparison of ARNI with ACEI to determine impact on global mortality and morbidity in heart failure trial. Circ Heart Fail. 2016;9:e002560.

Kristensen et al 2017
Kristensen SL, Mogensen UM, Jhund PS, Petrie MC, Preiss D1, Win S, et al. Clinical and echocardiographic characteristics and cardiovascular outcomes according to diabetes status in patients with heart failure and preserved ejection fraction: a report from the I-Preserve Trial (Irbesartan in Heart Failure With Preserved Ejection Fraction). Circulation. 201721;135(8):724-35.

Lam et al 2011
Lam CSP, Donal E, Kraigher-Krainer E, Vasan RS. Epidemiology and clinical course of heart failure with preserved ejection fraction. European Journal of Heart Failure 2011; 13: 18–28.

Lam et al 2020
Lam CSP, Voors AA, Ponikowski P, McMurray JJV, Solomon SD. Time to rename the middle child of heart failure: heart failure with mildly reduced ejection fraction. European Heart Journal 2020; 41:2353-55.

Levey at al 2009
Levey AS, Stevens LA, Schmid CH, Zhang YL, Castro AF 3rd, Feldman HI et al. A new equation to estimate glomerular filtration rate. Ann Intern Med. 2009 May 5; 150(9):604-12.

Lin et al 2000
Lin DY, Wei LJ, Yang I, Ying Z. Semiparametric regression for the mean and rate functions of recurrent events. Journal of the Royal Statistical Society: Series B (Statistical Methodology) 2000; 62(4):711–730.

Nauta et al 2017
Nauta JF, Hummel YM, van Melle JP, van der Meer P, Lam CSP, Ponikowski P, Voors AA. What have we learned about heart failure with mid□range ejection fraction one year after its introduction? European Journal of Heart Failure 2017; 19(12): 1569-1573.

Neal et al 2017
Neal B, Perkovic V, Mahaffey KW, de Zeeuw D, Fulcher G, Erondu N, et al, for the CANVAS Program Collaborative Group. Canagliflozin and cardiovascular and renal events in type 2 diabetes. N Engl J Med 2017; 377: 644-657.

Oktay et al 2013
Oktay AA, Rich JD, Shah SJ. The emerging epidemic of heart failure with preserved ejection fraction. Curr Heart Fail Rep 2013;10(4):401-10.

Okumura et al 2016
Okumura N, Jhund PS, Gong J, Lefkowitz MP, Rizkala AR, Rouleau JL, Shi VC, Swedberg K, Zile MR, Solomon SD, Packer M, McMurray JJ; PARADIGM-HF Investigators and Committees*. Importance of Clinical Worsening of Heart Failure Treated in the Outpatient Setting: Evidence From the Prospective Comparison of ARNI With ACEI to Determine Impact on Global Mortality and Morbidity in Heart Failure Trial (PARADIGM-HF). Circulation. 2016 Jun 7;133(23):2254-62. doi:
10.1161/CIRCULATIONAHA.115.020729. Epub 2016 Apr 20. PubMed PMID: 27143684.

Ott et al 2017

Ott C, Jumar A, Striepe K, Friedrich S, Karg MV, Bramlage P, et al. A randomised study of the impact of the SGLT2 inhibitor dapagliflozin on microvascular and macrovascular circulation. Cardiovasc Diabetol 2017;16:26.

Pfeffer et al 2015
Pfeffer MA, Claggett B, Assmann SF, Boineau R, Anand IS, Clausell N, et al. Regional Variation in Patients and Outcomes in the Treatment of Preserved Cardiac Function Heart Failure With an Aldosterone Antagonist (TOPCAT) Trial. Circulation. 2015;131:34-42.

Rådholm et al 2018
Rådholm K, Figtree, Perkovic V, Solomon SD, Mahaffey KW, et al. Canagliflozin and Heart Failure in Type 2 Diabetes Mellitus: Results From the CANVAS Program (Canagliflozin Cardiovascular Assessment Study). Circulation. 2018;137
https://doi.org/10.1161/CIRCULATIONAHA.118.034222

Shigiyama et al 2017
Shigiyama F, Kumashiro N, Miyagi M, Ikehara K, Kanda E, Uchino H, et al. Effectiveness of dapagliflozin on vascular endothelial function and glycemic control in patients with early-stage type 2 diabetes mellitus: DEFENCE study. Cardiovasc Diabetol 2017;16:84. DOI 10.1186/s12933-017-0564-0.

Skali et al 2014
Skali H, Dwyer EM, Goldstein R, Haigney M, Krone R, Kukin M, Lichstein E, McNitt S, Moss AJ, Pfeffer MA, Solomon SD. Prognosis and response to therapy of first inpatient and outpatient heart failure event in a heart failure clinical trial: MADIT-CRT. Eur J Heart Fail. 2014 May;16(5):560-5. doi: 10.1002/ejhf.71. Epub 2014 Feb 27. PubMed PMID: 24578164.

Solomon et al 2005

Solomon SD, Anavekar N, Skali H, McMurray JJ, Swedberg K, Yusuf S, Granger CB, Michelson EL, Wang D, Pocock S, Pfeffer MA; Candesartan in Heart Failure Reduction in Mortality (CHARM) Investigators. Influence of ejection fraction on cardiovascular outcomes in a broad spectrum of heart failure patients. Circulation 2005 Dec 13;112(24):3738-44

Solomon et al 2019

Solomon SD, McMurray JJV, Anand IS, Ge J, Lam CSP, Maggioni AP, Martinez F, Packer M, Pfeffer MA, Pieske B, Redfield MM, Rouleau JL, van Veldhuisen DJ, Zannad F, Zile MR, Desai AS, Claggett B, Jhund PS, Boytsov SA, Comin-Colet J, Cleland J, Düngen HD, Goncalvesova E, Katova T, Saraiva JFK, Lelonek M, Merkely B, Senni M, Shah SJ, Zhou J, Rizkala AR, Gong J, Shi VC, Lefkowitz MP, PARAGON-HF Investigators and Committees; Angiotensin-Neprilysin Inhibition in Heart Failure with Preserved Ejection Fraction. The New England Journal of Medicine 2019; 381:1609-1620.

Solomon et al 2020

Solomon SD, Vaduganathan M, Claggett BL, Packer M, Zile M, Swedberg K, Rouleau JJ, Pfeffer MA, Desai A, Lund LH, Kober L, Anand I, Sweitzer N, Linssen G, Merkely B, Arango JL, Vinereanu D, Chen CH, Senni M, Sibulo A, Boytsov S, Shi V, Rizkala A, Lefkowitz M, McMurray JJV; Sacubitril/Valsartan Across the Spectrum of Ejection Fraction in Heart Failure. Circulation 2020; 141:352-361.

Spertus et al 2005

Spertus J, Peterson E, Conard MW, Heidenreich PA, Krumholz HM, Jones P, et al. Monitoring clinical changes in patients with heart failure: A comparison of methods. Am Heart J 2005;150:707-15.

Thygesen et al 2012

Thygesen Kristian, Alpert Joseph S., Jaffe Allan S., Simoons Maarten L, Chaitman ,White Harvey D. Writing Group on behalf of the Joint ESC/ACCF/AHA/WHF Task Force for the Universal Definition of Myocardial Infarction. Third Universal Definition of Myocardial Infarction. Global Heart Vol: 7 (4), 2012 Page: 275-295

Wanner et al 2016

Wanner Christoph; Inzucchi, Silvio E.; Lachin, John M; Fitchett, David; von Eynatten, Maximilian. Cardio metabolic Risk Factor Changes Observed in Diabetes Prevention Programs in US Settings: A Systematic Review and Meta-analysis. New England Journal of Medicine Vol: 375 (4) 2016 Page: 323 - 334

Zannad et al 2014

Zannad F Garcia AA, Anker SD Armstrong PW Calvo G et al. Clinical outcome endpoints in heart failure trials: a European Society of Cardiology Heart Failure Association consensus document. Eur J Heart Failure 2013;15:1082–94.

Zinman et al 2015

Zinman B, Wanner C, Lachin JM, Fitchett D, Bluhmki E et al. Empagliflozin, cardiovascular outcomes, and mortality in type 2 diabetes. N Engl J Med 2015; 373:2117-2128.

11. SUPPORTING DOCUMENTATION AND OPERATIONAL CONSIDERATIONS

Appendix A Regulatory, ethical and study oversight considerations

A 1 Regulatory and ethical considerations

This study will be conducted in accordance with the protocol and with the following:

- Consensus ethical principles derived from international guidelines including the Declaration of Helsinki and Council for International Organizations of Medical Sciences (CIOMS) International Ethical Guidelines
- Applicable ICH Good Clinical Practice (GCP) Guidelines
- Applicable laws and regulations

The protocol, protocol amendments, ICF, Investigator Brochure, and other relevant documents (e.g. advertisements) must be submitted to an IRB/IEC by the investigator and reviewed and approved by the IRB/IEC before the study is initiated.

Any amendments to the protocol will require IRB/IEC approval before implementation of changes made to the study design, except for changes necessary to eliminate an immediate hazard to study subjects.

The investigator will be responsible for the following:

- Providing written summaries of the status of the study to the IRB/IEC annually or more frequently in accordance with the requirements, policies, and procedures established by the IRB/IEC
- Notifying the IRB/IEC of SAEs or other significant safety findings as required by IRB/IEC procedures
- Providing oversight of the conduct of the study at the site and adherence to requirements of 21 CFR, ICH guidelines, the IRB/IEC, European regulation 536/2014 for clinical studies (if applicable), and all other applicable local regulations

The study will be performed in accordance with the AstraZeneca policy on Bioethics and Human Biological Samples.

A 2 Financial disclosure

Investigators and sub-investigators will provide the sponsor with sufficient, accurate financial information as requested to allow the sponsor to submit complete and accurate financial certification or disclosure statements to the appropriate regulatory authorities. Investigators are

responsible for providing information on financial interests during the course of the study and for 1 year after completion of the study.

A 3 Informed consent process

The Investigator or his/her representative will explain the nature of the study to the subject or his/her legally authorised representative and answer all questions regarding the study.

Subjects must be informed that their participation is voluntary. Subjects or their legally authorised representative will be required to sign a statement of informed consent that meets the requirements of 21 CFR 50, local regulations, ICH guidelines, Health Insurance Portability and Accountability Act (HIPAA) requirements, where applicable, and the IRB/IEC or study centre.

The medical record must include a statement that written informed consent was obtained before the subject was enrolled in the study and the date the written consent was obtained. The authorised person obtaining the informed consent must also sign the ICF.
Subjects must be re-consented to the most current version of the ICF(s) during their participation in the study.

A copy of the ICF(s) must be provided to the subject or the subject’s legally authorised representative.

A subject who is rescreened is required to sign another ICF.

A 4 Data protection

Each subject will be assigned a unique identifier by the sponsor. Any subject records or data sets transferred to the sponsor will contain only the identifier; subject names or any information which would make the subject identifiable will not be transferred.

The subject must be informed that his/her personal study- related data will be used by the sponsor in accordance with local data protection law. The level of disclosure must also be explained to the subject.

The subject must be informed that his/her medical records may be examined by Clinical Quality Assurance auditors or other authorised personnel appointed by the sponsor, by appropriate IRB/IEC members, and by inspectors from regulatory authorities.

A 5 Committees structure

Executive Committee

Together with AZ, the Executive Committee will be responsible for the final overall study design, including the development of the study protocol and eCRF, supervision of the study conduct and progress, development of any protocol amendments needed during the study, liaison with the CEA committee and DMC committee as needed, development of the statistical analysis plan,

interpretation of the final data and reporting (presentations at international congresses and publications in peer reviewed journals) of the study.
The Executive Committee will make recommendations to AstraZeneca with regards to early stopping or modifications of the study based on the information received from the DMC. The Executive Committee will be comprised of designated international academic leaders and nonvoting members of the Sponsor, and will operate under an Executive Committee charter.

National Lead Investigator (NLI) Committee

The National Lead Investigator (NLI) Committee will be comprised of NLIs from each country where the study is conducted and supervised by the Executive Committee. Members of the committee will be responsible for providing clinical guidance on study implementation, recruitment and study conduct in their respective country.

Data Monitoring Committee (DMC)

An independent DMC will be appointed and will report to the Executive Committee. The DMC will be responsible for safeguarding the interests of the patients in the outcome study by assessing the safety of the intervention during the study, and for reviewing the overall conduct of the study. The DMC will have access to the individual treatment codes and be able to merge these with the collected study data while the study is ongoing. A DMC charter will be prepared to detail precise roles and responsibilities and procedures to ensure maintenance of the blinding and integrity of the study in the review of accumulating data and interactions with the Executive Committee.

Clinical Event Adjudication (CEA) Committee

The role of the CEA committee is to independently review, interpret and adjudicate potential endpoints that are experienced by the patients. Endpoints will be identified preliminary by the investigators, and also by AZ personnel or in the CEA process as specified in the CEA charter. The CEA committee members will not have access to individual treatment codes for any patient or clinical efficacy endpoint and safety event. The precise responsibilities and procedures applicable for CEA will be detailed in the CEA charter.

A 6 Dissemination of clinical study data

A description of this clinical trial will be available on http://astrazenecaclinicaltrials.com and http://www.clinicaltrials.gov as will the summary of the main study results when they are available. The clinical trial and/or summary of main study results may also be available on other websites according to the regulations of the countries in which the main study is conducted.

A 7 Data quality assurance

All subject data relating to the study will be recorded on printed or electronic CRF unless transmitted to the sponsor or designee electronically (e.g. laboratory data). The Investigator is responsible for verifying that data entries are accurate and correct by physically or electronically signing the CRF.

The Investigator must maintain accurate documentation (source data) that supports the information entered in the CRF.

The Investigator must permit study-related monitoring, audits, IRB/IEC review, and regulatory agency inspections and provide direct access to source data documents.

The sponsor or designee is responsible for the data management of this study including quality checking of the data.

Study monitors will perform ongoing source data verification to confirm that data entered into the CRF by authorised site personnel are accurate, complete, and verifiable from source documents; that the safety and rights of subjects are being protected; and that the study is being conducted in accordance with the currently approved protocol and any other study agreements, ICH GCP, and all applicable regulatory requirements.

Records and documents, including signed ICFs, pertaining to the conduct of this study must be retained by the Investigator for 15 years after study completion unless local regulations or institutional policies require a longer retention period. No records may be destroyed during the retention period without the written approval of the sponsor. No records may be transferred to another location or party without written notification to the sponsor.

A 8 Source documents

Source documents provide evidence for the existence of the subject and substantiate the integrity of the data collected. Source documents are filed at the Investigator's site.

Data reported on the CRF that are transcribed from source documents must be consistent with the source documents or the discrepancies must be explained. The Investigator may need to request previous medical records or transfer records, depending on the study. Also, current medical records must be available.

A 9 Publication policy

The results of this study may be published or presented at scientific meetings. If this is foreseen, the investigator agrees to submit all manuscripts or abstracts to the sponsor before submission. This allows the sponsor to protect proprietary information and to provide comments.

The sponsor will comply with the requirements for publication of study results. In accordance with standard editorial and ethical practice, the sponsor will generally support publication of multicentre studies only in their entirety and not as individual site data. In this case, a coordinating investigator will be designated by mutual agreement.

Authorship will be determined by mutual agreement and in line with International Committee of Medical Journal Editors authorship requirements.

Appendix B Adverse event definitions and additional safety information

B 1 Definition of adverse events

An adverse event is the development of any untoward medical occurrence in a subject or clinical study subject administered a medicinal product and which does not necessarily have a causal relationship with this treatment. An AE can therefore be any unfavourable and unintended sign (e.g. an abnormal laboratory finding), symptom (for example nausea, chest pain), or disease temporally associated with the use of a medicinal product, whether or not considered related to the medicinal product.

The term AE is used to include both serious and non-serious AEs and can include a deterioration of a pre- existing medical occurrence. An AE may occur at any time, including run-in or washout periods, even if no Study treatment has been administered.

B 2 Definitions of serious adverse event

A serious adverse event is an AE occurring during any study phase (i.e., run-in, treatment, washout, follow-up), that fulfils one or more of the following criteria:

- Results in death
- Is immediately life-threatening
- Requires in-subject hospitalisation or prolongation of existing hospitalisation
- Results in persistent or significant disability or incapacity
- Is a congenital abnormality or birth defect
- Is an important medical event that may jeopardise the subject or may require medical treatment to prevent one of the outcomes listed above.

B 3 Life threatening

'Life-threatening' means that the subject was at immediate risk of death from the AE as it occurred or it is suspected that use or continued use of the product would result in the subject's death. 'Life-threatening' does not mean that had an AE occurred in a more severe form it might have caused death (e.g., hepatitis that resolved without hepatic failure).

B 4 Hospitalisation

Outpatient treatment in an emergency room is not in itself a serious AE, although the reasons for it may be (e.g., bronchospasm, laryngeal oedema). Hospital admissions and/or surgical operations planned before or during a study are not considered AEs if the illness or disease existed before the subject was enrolled in the study, provided that it did not deteriorate in an unexpected way during the study.

B 5 Important medical event or medical treatment

Medical and scientific judgement should be exercised in deciding whether a case is serious in situations where important medical events may not be immediately life threatening or result in death, hospitalisation, disability or incapacity but may jeopardize the subject or may require medical treatment to prevent one or more outcomes listed in the definition of serious. These should usually be considered as serious.

Simply stopping the suspect drug does not mean that it is an important medical event; medical judgement must be used.

- Angioedema not severe enough to require intubation but requiring iv hydrocortisone treatment
- Hepatotoxicity caused by paracetamol (acetaminophen) overdose requiring treatment with N-acetylcysteine
- Intensive treatment in an emergency room or at home for allergic bronchospasm
- Blood dyscrasias (e.g. neutropenia or anaemia requiring blood transfusion, etc.) or convulsions that do not result in hospitalisation
- Development of drug dependency or drug abuse

B 6 Intensity rating scale:

1. mild (awareness of sign or symptom, but easily tolerated)
2. moderate (discomfort sufficient to cause interference with normal activities)
3. severe (incapacitating, with inability to perform normal activities)

It is important to distinguish between serious and severe AEs. Severity is a measure of intensity whereas seriousness is defined by the criteria in Appendix B 2. An AE of severe intensity need not necessarily be considered serious. For example, nausea that persists for several hours may be considered severe nausea, but not a SAE unless it meets the criteria shown in Appendix B 2. On the other hand, a stroke that results in only a limited degree of disability may be considered a mild stroke but would be a SAE when it satisfies the criteria shown in Appendix B 2.

B 7 A Guide to Interpreting the Causality Question

When assessing causality consider the following factors when deciding if there is a ‘reasonable possibility’ that an AE may have been caused by the drug.

- Time Course. Exposure to suspect drug. Has the subject actually received the suspect drug? Did the AE occur in a reasonable temporal relationship to the administration of the suspect drug?
- Consistency with known drug profile. Was the AE consistent with the previous knowledge of the suspect drug (pharmacology and toxicology) or drugs of the same

pharmacological class? Or could the AE be anticipated from its pharmacological properties?

- De-challenge experience. Did the AE resolve or improve on stopping or reducing the dose of the suspect drug?
- No alternative cause. The AE cannot be reasonably explained by another aetiology such as the underlying disease, other drugs, other host or environmental factors.
- Re-challenge experience. Did the AE reoccur if the suspected drug was reintroduced after having been stopped? AstraZeneca would not normally recommend or support a re-challenge.
- Laboratory tests. A specific laboratory investigation (if performed) has confirmed the relationship.

In difficult cases, other factors could be considered such as:

- Is this a recognized feature of overdose of the drug?
- Is there a known mechanism?

Causality of 'related' is made if following a review of the relevant data, there is evidence for a 'reasonable possibility' of a causal relationship for the individual case. The expression 'reasonable possibility' of a causal relationship is meant to convey, in general, that there are facts (evidence) or arguments to suggest a causal relationship.

The causality assessment is performed based on the available data including enough information to make an informed judgment. With limited or insufficient information in the case, it is likely that the event(s) will be assessed as 'not related'.

Causal relationship in cases where the disease under study has deteriorated due to lack of effect should be classified as no reasonable possibility.

B 8 Medication Error

For the purposes of this clinical study a medication error is an unintended failure or mistake in the treatment process for an AstraZeneca investigational product that either causes harm to the participant or has the potential to cause harm to the participant.

A medication error is not lack of efficacy of the drug, but rather a human or process related failure while the drug is in control of the study site staff or participant.

Medication error includes situations where an error:

- Occurred
- Was identified and intercepted before the participant received the drug

- Did not occur, but circumstances were recognized that could have led to an error

Examples of events to be reported in clinical studies as medication errors:

- Drug name confusion
- Dispensing error e.g. medication prepared incorrectly, even if it was not actually given to the participant
- Drug not administered as indicated, for example, wrong route or wrong site of administration
- Drug not taken as indicated e.g. tablet dissolved in water when it should be taken as a solid tablet
- Drug not stored as instructed e.g. kept in the fridge when it should be at room temperature
- Wrong participant received the medication (excluding IxRS errors)
- Wrong drug administered to participant (excluding IxRS errors)

Examples of events that **do not** require reporting as medication errors in clinical studies:

- Errors related to or resulting from IxRS - including those which lead to one of the above listed events that would otherwise have been a medication error
- Participant accidentally missed drug dose(s) e.g. forgot to take medication
- Accidental overdose (will be captured as an overdose)
- Participant failed to return unused medication or empty packaging
- Errors related to background and rescue medication, or standard of care medication in open label studies, even if an AZ product

Medication errors are not regarded as AEs but AEs may occur as a consequence of the medication error.

Appendix C Cardiovascular related events

C 1 Myocardial Infarctions (MI)

MIs are not endpoints in this study but unstable angina and myocardial infarction should be recorded as SAEs if serious criteria are met and additional information be collected in specific eCRF. The diagnoses of unstable angina and MI should adhere to the standardised definitions for endpoints (Hicks et al 2018) described in Appendix C 2

C 2 Diagnosis of MI and Unstable Angina

Myocardial infarction (MI)

The diagnosis of an MI should be made according to standard clinical practice but is expected to align with the criteria from Third Universal Definition of MI, i.e. detection of a rise and/or fall of cardiac biomarkers such as troponin and at least one of the following: typical clinical symptoms, ischaemic ECG findings, imaging evidence of myocardial injury, or detection of an intracoronary thrombus by angiography or autopsy (Thygesen et al 2012).

The diagnosis should be made by, or in consultation with, a cardiologist. The findings supporting the diagnosis should be documented in the description of the SAE in the eCRF.

Unstable Angina (UA)

Unstable Angina (UA) is not an endpoint in this study but should be recorded as SAEs (and DAEs when appropriate). The diagnosis of an UA should be made according to standard clinical practice but is expected to align with the following definition:

The diagnosis of unstable angina will require ischemic chest pain (or equivalent) at rest ≥10 minutes in duration considered to be myocardial ischemia upon final diagnosis and prompting hospitalisation within 24 hours of the most recent symptoms, and without elevation in cardiac biomarkers of necrosis, and the presence of objective evidence of ischemia as defined by at least 1 of the following criteria:

1. New or worsening ST or T wave changes in ≥2 anatomically contiguous leads on a resting ECG (in the absence of LVH and LBBB):

a) transient (<20 minutes) ST elevation at the J point ≥ 0.2 mV in men (> 0.25 mV in men < 40 years old) or ≥ 0.15 mV in women in leads V2-V3 and/or ≥ 0.1 mV in other leads, or

b) horizontal or down-sloping ST depression ≥ 0.10 mV, or

c) T-wave inversion ≥ 0.2 mV

2. Definite evidence of myocardial ischemia on myocardial scintigraphy (clear reversible perfusion defect), stress echocardiography (reversible wall motion abnormality), or MRI (myocardial perfusion deficit under pharmacologic stress) that is believed to be responsible for the myocardial ischemic symptoms/signs.

3. Angiographic evidence of ≥ 70% lesion and/or thrombus in an epicardial coronary artery that is believed to be responsible for the myocardial ischemic symptoms/signs.

C 3 Stroke

Stroke is not an endpoint in this study but should be recorded as SAEs if serious criteria are met, with additional information e.g. classification of stroke type (ischaemic, haemorrhagic, or undetermined) collected in a specific eCRF.

The diagnosis of stroke should be made according to standard clinical practice and align with the definition for stroke in the standardized definitions for endpoints (Hicks et al 2018) described in Appendix C 4 and be differentiated vs Transient Ischaemic Attack (TIA).

C 4 Definition of Stroke and Transient Ischemic Attack

The distinction between an Ischemic Stroke and a Transient Ischemic Attack is the presence of infarction. Persistence of symptoms ≥24 hours or until death[3] is an acceptable indicator of acute infarction in the absence of imaging evidence of infarction.

Transient Ischemic Attack
Transient ischemic attack (TIA) is defined as a transient episode of focal neurological dysfunction caused by brain, spinal cord, or retinal ischemia, *without* acute infarction.

Stroke
Stroke is defined as an acute episode of focal or global neurological dysfunction caused by brain, spinal cord, or retinal vascular injury as a result of haemorrhage or infarction.

Classification:

A. Ischemic Stroke

Ischemic stroke is defined as an acute episode of focal cerebral, spinal, or retinal dysfunction caused by infarction of central nervous system tissue.
Haemorrhage may be a consequence of ischemic stroke. In this situation, the stroke is an ischemic stroke with haemorrhagic transformation and not a haemorrhagic stroke.

B. Haemorrhagic Stroke

Haemorrhagic stroke is defined as an acute episode of focal or global cerebral or spinal dysfunction caused by non-traumatic intraparenchymal, intraventricular, or subarachnoid haemorrhage. NOTE: Subdural hematomas are intracranial haemorrhagic events and not strokes.

C. Undetermined Stroke

Undetermined stroke is defined as an acute episode of focal or global neurological dysfunction caused by presumed brain, spinal cord, or retinal vascular injury as a result of haemorrhage or infarction but with insufficient information to allow categorization as either ischemic or haemorrhagic.

References:

Hicks KA et al. 2017 Cardiovascular and Stroke Endpoint Definitions for Clinical Trials. J Am Coll Cardiol 2018;71:1021–34 https://doi.org/10.1016/j.jacc.2017.12.048

Draft Definitions for CDISC August 20, 2014

Sacco RL, Kasner SE, Broderick JP, Caplan LR, Connors JJ, Culebras A, Elkind MSV, George MG, Hamdan AD, Higashida RT, Hoh BL, Janis LS, Kase CS, Kleindorfer DO, Lee J-M, Moseley ME, Peterson ED, Turan TN, Valderrama AL, Vinters HV; on behalf of the American Heart Association Stroke Council, Council on Cardiovascular Surgery and Anesthesia, Council on Cardiovascular Radiology and Intervention, Council on Cardiovascular and Stroke Nursing, Council on Epidemiology and Prevention, Council on Peripheral Vascular Disease, and Council on Nutrition, Physical Activity and Metabolism. An updated definition of stroke for the 21st century: a statement for healthcare professionals from the American Heart Association/American Stroke Association. Stroke. 2013;44:2064-2089.

Appendix D Genetics

D 1 Use/analysis of DNA

Genetic variation may impact a subject's response to therapy, susceptibility to, and severity and progression of disease. Variable response to therapy may be due to genetic determinants that impact drug absorption, distribution, metabolism, and excretion; mechanism of action of the drug; disease aetiology; and/or molecular subtype of the disease being treated. Therefore, where local regulations and IRB/IEC allow, a blood sample will be collected for DNA analysis from consenting subjects.

AstraZeneca intends to collect and store DNA for genetic research to explore how genetic variations may affect clinical parameters, risk and prognosis of diseases, and the response to medications. Genetic research may lead to better understanding of diseases, better diagnosis of diseases or other improvements in health care and to the discovery of new diagnostics, treatments or medications.

In addition, collection of DNA samples from populations with well described clinical characteristics may lead to improvements in the design and interpretation of clinical trials and, possibly, to genetically guided treatment strategies.

Genetic research may consist of the analysis of the structure of the subject´s DNA, i.e. the entire genome.

The results of genetic analyses may be reported in the clinical study report (CSR) or in a separate study summary.

The sponsor will store the DNA samples in a secure storage space with adequate measures to protect confidentiality.

The samples will be retained while research on heart failure with preserved ejection fraction continues but no longer than 15 years or other period as per local requirements.

D 2 Genetic research plan and procedures

Selection of genetic research population

Study selection record

All subjects will be asked to participate in this genetic research. Participation is voluntary and if a subject decline to participate there will be no penalty or loss of benefit. The subject will not be excluded from any aspect of the main study.

Inclusion criteria

- For inclusion in this genetic research, subjects must fulfil all of the inclusion criteria described in the main body of the Clinical Study Protocol **and** Provide informed consent for the genetic sampling and analyses.

Exclusion criteria

Exclusion from this genetic research may be for any of the exclusion criteria specified in the main study or any of the following:

- Previous allogeneic bone marrow transplant
- Non-leukocyte depleted whole blood transfusion in 120 days of genetic sample collection

Withdrawal of consent for genetic research:

Subjects may withdraw from this genetic research at any time, independent of any decision concerning participation in other aspects of the main study. Voluntary withdrawal will not prejudice further treatment. Procedures for withdrawal are outlined in Section 7 of the main Clinical Study Protocol.

Collection of samples for genetic research

The blood sample for genetic research will be obtained from the subjects at Visit 2. Although DNA is stable, early sample collection is preferred to avoid introducing bias through excluding subjects who may withdraw due to an adverse event (AE), such subjects would be important to include in any genetic analysis. If for any reason the sample is not drawn at Visit 2, it may be taken at any visit until the last study visit. Only one sample should be collected per subject for genetics during the study. Samples will be collected, labelled, stored, and shipped as detailed in the Laboratory Manual.

Coding and storage of DNA samples

The processes adopted for the coding and storage of samples for genetic analysis are important to maintain subject confidentiality. Samples will be stored for a maximum of 15 years, from the date of last subject last visit, after which they will be destroyed. DNA is a finite resource that is used up during analyses. Samples will be stored and used until no further analyses are possible or the maximum storage time has been reached.

An additional second code will be assigned to the blood sample either before or at the time of DNA extraction replacing the information on the sample tube. Thereafter, the sample will be identifiable only by the second, unique number. This number is used to identify the sample and corresponding data at the AstraZeneca genetics laboratories, or at the designated organisation. No personal details identifying the individual will be available to any person (AstraZeneca employee or designated organisations working with the DNA).

The link between the subject enrolment/randomisation code and the second number will be maintained and stored in a secure environment, with restricted access at AstraZeneca or designated organisations. The link will be used to identify the relevant DNA samples for analysis, facilitate correlation of genotypic results with clinical data, allow regulatory audit, and permit tracing of samples for destruction in the case of withdrawal of consent.

Ethical and regulatory requirements

The principles for ethical and regulatory requirements for the study, including this genetics research component, are outlined in Appendix A.

Informed consent

The genetic component of this study is optional and the subject may participate in other components of the main study without participating in the genetic component. To participate in the genetic component of the study the subject must sign and date both the consent form for the main study and the genetic component of the study. Copies of both signed and dated consent forms must be given to the subject and the original filed at the study centre. The Principal Investigator(s) is responsible for ensuring that consent is given freely and that the subject understands that they may freely withdrawal from the genetic aspect of the study at any time.

Subject data protection

AstraZeneca will not provide individual genotype results to subjects, any insurance company, any employer, their family members, general physician unless required to do so by law.

Extra precautions are taken to preserve confidentiality and prevent genetic data being linked to the identity of the subject. In exceptional circumstances, however, certain individuals might see both the genetic data and the personal identifiers of a subject. For example, in the case of a medical emergency, an AstraZeneca Physician or an investigator might know a subject’s identity and also have access to his or her genetic data. In addition, Regulatory authorities may require access to the relevant files, though the subject’s medical information and the genetic files would remain physically separate.

Data management

Any genotype data generated in this study will be stored at a secure system at AstraZeneca and/or designated organizations to analyse the samples.

AstraZeneca and its designated organisations may share summary results (such as genetic differences from groups of individuals with a disease) from this genetic research with other researchers, such as hospitals, academic organisations or health insurance companies. This can be done by placing the results in scientific databases, where they can be combined with the results of similar studies to learn even more about health and disease. The researchers can only use this information for health-related research purposes. Researchers may see summary results but they will not be able to see individual subject data or any personal identifiers.

Some or all of the clinical datasets from the main study may be merged with the genetic data in a suitable secure environment separate from the clinical database.

Statistical methods and determination of sample size

The number of subjects that will agree to participate in the genetic research is unknown. It is therefore not possible to establish whether sufficient data will be collected to allow a formal statistical evaluation or whether only descriptive statistics will be generated. A Statistical Analysis Plan may be prepared where appropriate.

Appendix E Handling of Human Biological Samples

E 1 Chain of custody of biological samples

A full chain of custody is maintained for all samples throughout their lifecycle.

The Investigator at each centre keeps full traceability of collected biological samples from the subjects while in storage at the centre until shipment or disposal (where appropriate).

The sample receiver keeps full traceability of the samples while in storage and during use until used or disposed of or until further shipment and keeps documentation of receipt of arrival.

AstraZeneca will keep oversight of the entire life cycle through internal procedures, monitoring of study sites, auditing or process checks, and contractual requirements of external laboratory providers

Samples retained for further use will be stored in the AZ-assigned biobanks and will be registered by the AstraZeneca Biobank Team during the entire life cycle.

If required, AstraZeneca will ensure that remaining biological samples are returned to the site according to local regulations or at the end of the retention period, whichever is the sooner.

E 2 Withdrawal of Informed Consent for donated biological samples

If a subject withdraws consent to the use of donated biological samples, the samples will be disposed of/destroyed, and the action documented. If samples are already analysed, AstraZeneca is not obliged to destroy the results of this research.

As collection of the biological sample(s) is an integral part of the study, then the subject is withdrawn from further study participation.

The Investigator:

- Ensures subjects' withdrawal of informed consent to the use of donated samples is notified immediately to AstraZeneca
- Ensures that biological samples from that subject, if stored at the study site, are immediately identified, disposed of /destroyed, and the action documented
- Ensures the organization(s) holding the samples is/are informed about the withdrawn consent immediately and that samples are disposed of/destroyed, the action documented and the signed document returned to the study site
- Ensures that the subject and AstraZeneca are informed about the sample disposal.

AstraZeneca ensures the organizations holding the samples is/are informed about the withdrawn consent immediately and that samples are disposed of/destroyed and the action documented and returned to the study site.

E 3 International Airline Transportation Association (IATA) 6.2 Guidance Document

LABELLING AND SHIPMENT OF BIOHAZARD SAMPLES

International Airline Transportation Association (IATA) classifies biohazardous agents into 3 categories (http://www.iata.org/whatwedo/cargo/dangerous_goods/infectious_substances.htm). For transport purposes the classification of infectious substances according to risk groups was removed from the Dangerous Goods Regulations in the 46th edition (2005). Infectious substances are now classified either as Category A, Category B or Exempt. There is no direct relationship between Risk Groups and Categories A and B.

Category A Infectious Substances are infectious substances in a form that, when exposure to it occurs, is capable of causing permanent disability, life-threatening or fatal disease in otherwise healthy humans or animals. Category A pathogens are e.g., Ebola, Lassa fever virus:

- Are to be packed and shipped in accordance with IATA Instruction 602.

Category B Infectious Substances are infectious Substances that do not meet the criteria for inclusion in Category A. Category B pathogens are e.g., Hepatitis A, B, C, D, and E viruses, Human immunodeficiency virus types 1 and 2. They are assigned the following UN number and proper shipping name:

- UN 3373 – Biological Substance, Category B
- Are to be packed in accordance with UN3373 and IATA 650

Exempt - all other materials with minimal risk of containing pathogens

- Clinical trial samples will fall into Category B or exempt under IATA regulations
- Clinical trial samples will routinely be packed and transported at ambient
- Temperature in IATA 650 compliant packaging (http://www.iata.org/whatwedo/cargo/dangerous_goods/infectious_substances.ht m)

- **Biological samples transported in dry ice require additional dangerous goods specification for the dry-ice content**
- IATA compliant courier and packaging materials should be used for packing and transportation and packing should be done by an IATA certified person, as applicable
- Samples routinely transported by road or rail are subject to local regulations which require that they are also packed and transported in a safe and appropriate way to contain any risk of infection or contamination by using approved couriers and packaging/containment materials at all times. The IATA 650 biological sample containment standards are encouraged wherever possible when road or rail transport is used.

Appendix F Actions required in cases of increases in liver biochemistry and evaluation of Hy's Law (not applicable)

Appendix G Medical device incidents: definition and procedures for recording, evaluating, follow-up, and reporting (not applicable)

Appendix H Abbreviations

Abbreviation or special term	Explanation
AE	Adverse Event
BP	Blood Pressure
CEA	Clinical Event Adjudication
COPD	Chronic Obstructive Pulmonary Disease
COVID-19	Coronavirus Disease 2019
CSA	Clinical study Agreement
CV	Cardiovascular
DAE	Adverse Event leading to discontinuation of investigational product
DKA	Diabetic Ketoacidosis
DMC	Data Monitoring Committee
eCRF	Electronic Case Report Form
EC	Ethics Committee, synonymous to Institutional Review Board (IRB) and Independent Ethics Committee (IEC)
ECG	Electrocardiogram
EDC	Electronic Data Capture
EHRs	Electronic Health Records
FAS	Full Analysis Set
GCP	Good Clinical Practice
HF	Heart Failure
HFpEF	Heart Failure with Preserved Ejection Fraction
HR	Hazard Ratio
ICF	Informed Consent Form
ICH	International Conference on Harmonisation
International Co-ordinating Investigator	If a study is conducted in several countries the International Co-ordinating Investigator is the Investigator co-ordinating the Investigators and/or activities internationally.
IxRS	Interactive Voice/Web Response System
KCCQ	Kansas City Cardiomyopathy Questionnaire
LAE	Left Atrial Enlargement
LSLV	Last Subject Last Visit
LVEF	Left Ventricular Ejection Fraction
MI	Myocardial Infarction

NYHA	New York Heart Association
PACD	Primary Analysis Censoring Date
PTDV	Premature Treatment Discontinuation Visit
PI	Principal Investigator
SAE	Serious Adverse Event
SAP	Statistical Analysis Plan
SCV	Study Closure Visit
SoA	Schedule of Activities
T2D	Type 2 Diabetes Mellitus

Appendix I New York Heart Association (NYHA) Functional Classification

**Class**	**Patient symptoms**
I	No limitation of physical activity. Ordinary physical activity does not cause undue fatigue, palpitation, dyspnoea (shortness of breath).
II	Slight limitation of physical activity. Comfortable at rest. Ordinary physical activity results in fatigue, palpitation, dyspnea (shortness of breath).
III	Marked limitation of physical activity. Comfortable at rest. Less than ordinary activity causes fatigue, palpitation, or dyspnea.
IV	Unable to carry on any physical activity without discomfort. Symptoms of heart failure at rest. If any physical activity is undertaken, discomfort increases.

Appendix J The KC Cardiomyopathy Questionnaire

The KC Cardiomyopathy Questionnaire

The following questions refer to your heart failure and how it may affect your life. Please read and complete the following questions. There are no right or wrong answers. Please mark the answer that best applies to you.

1. **Heart failure** affects different people in different ways. Some feel shortness of breath while others feel fatigue. Please indicate how much you are limited by **heart failure** (shortness of breath or fatigue) in your ability to do the following activities <u>over the past 2 weeks.</u>

Place an **X** in one box on each line

Activity	Extremely Limited	Quite a bit Limited	Moderately Limited	Slightly Limited	Not at all Limited	Limited for other reasons or did not do the activity

Dressing yourself	❑	❑	❑	❑	❑	❑
Showering/Bathing	❑	❑	❑	❑	❑	❑
Walking 1 block on level ground	❑	❑	❑	❑	❑	❑
Doing yardwork, housework or carrying groceries	❑	❑	❑	❑	❑	❑
Climbing a flight of stairs without stopping	❑	❑	❑	❑	❑	❑
Hurrying or jogging (as if to catch a bus)	❑	❑	❑	❑	❑	❑

2. <u>Compared with 2 weeks ago</u>, have your symptoms of **heart failure** (shortness of breath, fatigue, or ankle swelling) changed?

My symptoms of **heart failure** have become…

**Much worse**	**Slightly worse**	**Not changed**	**Slightly better**	**Much better**	**I've had no symptoms over the last 2 weeks**
❑	❑	❑	❑	❑	❑

3. Over the <u>past 2 weeks</u>, how many times did you have **swelling** in your feet, ankles or legs when you woke up in the morning?

**Every morning**	**3 or more times a week, but not every day**	**1-2 times a week**	**Less than once a week**	**Never over the past 2 weeks**
❑	❑	❑	❑	❑

4. Over the <u>past 2 weeks</u>, how much has **swelling** in your feet, ankles or legs bothered you?

It has been ...

**Extremely bothersome**	**Quite a bit bothersome**	**Moderately bothersome**	**Slightly bothersome**	**Not at all bothersome**	**I've had no swelling**

❑ ❑ ❑ ❑ ❑ ❑

5. Over the past 2 weeks, on average, how many times has **fatigue** limited your ability to do what you want?

**All of the time**	**Several times per day**	**At least once a day**	**3 or more times per week but not every day**	**1-2 times per week**	**Less than once a week**	**Never over the past 2 weeks**
❑	❑	❑	❑	❑	❑	❑

6. Over the past 2 weeks, how much has your **fatigue** bothered you?

It has been ...

**Extremely bothersome**	**Quite a bit bothersome**	**Moderately bothersome**	**Slightly bothersome**	**Not at all bothersome**	**I've had no fatigue**
❑	❑	❑	❑	❑	❑

7. Over the past 2 weeks, on average, how many times has **shortness of breath** limited your ability to do what you wanted?

**All of the time**	**Several times per day**	**At least once a day**	**3 or more times per week but not everyday**	**1 – 2 times per week**	**Less than once a week**	**Never over the past 2 weeks**
❑	❑	❑	❑	❑	❑	❑

8. Over the past 2 weeks, how much has your **shortness of breath** bothered you?

It has been ...

**Extremely bothersome**	**Quite a bit bothersome**	**Moderately bothersome**	**Slightly bothersome**	**Not at all bothersome**	**I've had no shortness of breath**
❑	❑	❑	❑	❑	❑

9. Over the past 2 weeks, on average, how many times have you been forced to sleep sitting up in a chair or with at least 3 pillows to prop you up because of **shortness of breath**?

Every night	3 or more times per week, but not every day	1-2 times a week	Less than once a week	Never over the past 2 weeks
❑	❑	❑	❑	❑

10. Heart failure symptoms can worsen for a number of reasons. How sure are you that you know what to do, or whom to call, if your **heart failure** gets worse?

Not at all sure	Not very sure	Somewhat sure	Mostly sure	Completely sure
❑	❑	❑	❑	❑

11. How well do you understand what things you are able to do to keep your **heart failure** symptoms from getting worse? (for example, weighing yourself, eating a low salt diet etc.)

Do not understand at all	Do not understand very well	Somewhat understand	Mostly understand	Completely understand
❑	❑	❑	❑	❑

12. Over the past 2 weeks, how much has your **heart failure** limited your enjoyment of life?

It has extremely limited my enjoyment of life	It has limited my enjoyment of life quite a bit	It has moderately limited my enjoyment of life	It has slightly limited my enjoyment of life	It has not limited my enjoyment of life at all
❑	❑	❑	❑	❑

13. If you had to spend the rest of your life with your **heart failure** the way it is right now, how would you feel about this?

Not at all satisfied	Mostly dissatisfied	Somewhat satisfied	Mostly satisfied	Completely satisfied
❑	❑	❑	❑	❑

14. Over the past 2 weeks, how often have you felt discouraged or down in the dumps because of your heart failure?

I felt that way all of the time	I felt that way most of the time	I occasionally felt that way	I rarely felt that way	I never felt that way
❑	❑	❑	❑	❑

15. How much does your heart failure affect your lifestyle? Please indicate how your heart failure may have limited your participation in the following activities over the past 2 weeks. Please place an **X** in one box on each line

**Activity**	**Severely limited**	**Limited quite a bit**	**Moderately limited**	**Slightly limited**	**Did not limit at all**	**Does not apply or did not do for other reasons**
**Hobbies, Recreational activities**	❑	❑	❑	❑	❑	❑
**Working or doing household chores**	❑	❑	❑	❑	❑	❑
**Visiting family or friends out of your home**	❑	❑	❑	❑	❑	❑
**Intimate relationships with loved ones**	❑	❑	❑	❑	❑	❑

Copyright © 1992 –2005 John Spertus, MD, MPH Original US English

Appendix K EQ-5D-5L Questionnaire

Health Questionnaire

Under each heading, please check the ONE box that best describes your health TODAY

MOBILITY

I have no problems walking ❑

I have slight problems walking ❑

I have moderate problems walking ❑

I have severe problems walking ❑

I am unable to walk ❑

SELF-CARE

I have no problems washing or dressing myself ❑

I have slight problems washing or dressing myself ❑

I have moderate problems washing or dressing myself ❑

I have severe problems washing or dressing myself ❑

I am unable to wash or dress myself ❑

USUAL ACTIVITIES *(e.g. work, study, housework, family or leisure activities)*

I have no problems doing my usual activities ☐

I have slight problems doing my usual activities ☐

I have moderate problems doing my usual activities ☐

I have severe problems doing my usual activities ☐

I am unable to do my usual activities ☐

PAIN / DISCOMFORT

I have no pain or discomfort ☐

I have slight pain or discomfort ☐

I have moderate pain or discomfort ☐

I have severe pain or discomfort ☐

I have extreme pain or discomfort ☐

ANXIETY / DEPRESSION

I am not anxious or depressed ☐

I am slightly anxious or depressed ☐

I am moderately anxious or depressed ☐

I am severely anxious or depressed ☐

I am extremely anxious or depressed ☐

- We would like to know how good or bad your health is
  TODAY.
- This scale is numbered from 0 to 100.
- 100 means the best health you can imagine.
  0 means the worst health you can imagine.
- Mark an X on the scale to indicate how your health is TODAY.
- Now, please write the number you marked on the scale in the box below.

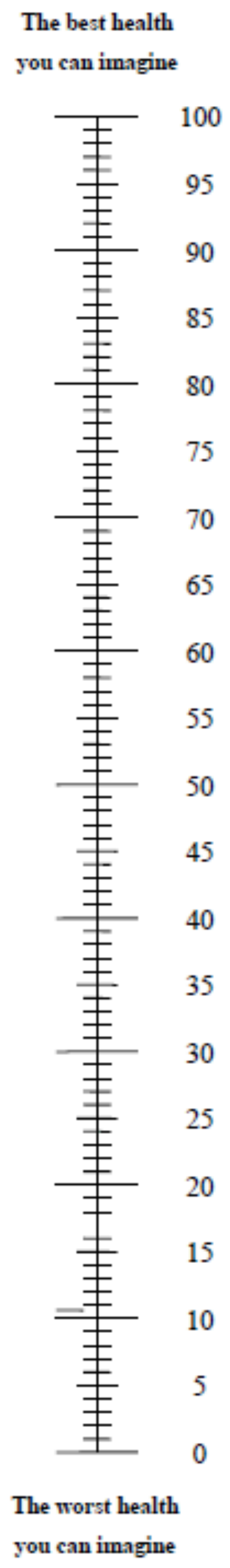

YOUR HEALTH TODAY =

USA (English) © 2009 EuroQol Group. EQ-5D™ is a trade mark of the EuroQol Group

Appendix L Patient Global Impression of Severity for Heart Failure Symptoms

Patient Global Impression of Severity for Heart Failure Symptoms

Overall, how would you rate the severity of your heart failure symptoms today?

☐ No symptoms

☐ Very mild

☐ Mild

☐ Moderate

☐ Severe

☐ Very Severe

SIGNATURE PAGE

This is a representation of an electronic record that was signed electronically and this page is the manifestation of the electronic signature

**Document Name:** d169cc00001-csp-v4		
**Document Title:**	D169CC00001 Clinical Study Protocol version 4	
**Document ID:**	Doc ID-003827083	
**Version Label:**	6.0 CURRENT LATEST APPROVED	
**Server Date (dd-MMM-yyyy HH:mm 'UTC'Z)**	**Signed by**	**Meaning of Signature**
13-Nov-2020 13:23 UTC	Daniel Lindholm	Content Approval
16-Nov-2020 16:15 UTC	Joan Buenconsejo	Content Approval
13-Nov-2020 07:36 UTC	Johan Holmberg	Content Approval
16-Nov-2020 08:56 UTC	Magnus Petersson	Content Approval
13-Nov-2020 08:29 UTC	Magnus Ysander	Qualified Person Approval

Notes: (1) Document details as stored in ANGEL, an AstraZeneca document management system.

Statistical Analysis Plan

Study Code	D169CC00001
Edition Number	1.0
Date	27/August/2018

An International, Double-blind, Randomised, Placebo-Controlled Phase III Study to Evaluate the Effect of Dapagliflozin on Reducing CV Death or Worsening Heart Failure in Patients with Heart Failure with Preserved Ejection Fraction (HFpEF)

DELIVER - Dapagliflozin Evaluation to improve the LIVEs of patients with pReserved ejection fraction heart failure

An International, Double-blind, Randomised, Placebo-Controlled Phase III Study to Evaluate the Effect of Dapagliflozin on Reducing CV Death or Worsening Heart Failure in Patients with Heart Failure with Preserved Ejection Fraction (HFpEF)

DELIVER - Dapagliflozin Evaluation to improve the LIVEs of patients with pReserved ejection fraction heart failure

Study Statistician

	Olof Bengtsson	Date

An International, Double-blind, Randomised, Placebo-Controlled Phase III Study to Evaluate the Effect of Dapagliflozin on Reducing CV Death or Worsening Heart Failure in Patients with Heart Failure with Preserved Ejection Fraction (HFpEF)

DELIVER - Dapagliflozin Evaluation to improve the LIVEs of patients with pReserved ejection fraction heart failure

Biometrics Team Leader

	Hongjian Li	Date

TABLE OF CONTENTS

1 STUDY DETAILS 9
1.1 Study objectives 9
1.1.1 Primary objective 9
1.1.2 Secondary objectives 9
1.1.3 Safety objectives 9
1.1.4 Exploratory objectives 10
1.2 Definitions 10
1.2.1 Primary analysis censoring date 10
1.2.2 Withdrawal of consent 11
1.2.3 Discontinuation from study drug 11
1.2.4 Vital Status 11
1.2.5 Lost to follow-up 11
1.3 Study design 11
1.3.1 Randomisation 12
1.4 Number of subjects 13
2 ANALYSIS SETS 14
2.1 Definition of analysis sets 14
2.1.1 Full analysis set 14
2.1.2 Safety analysis set 14
2.2 Violations and deviations 14
3 PRIMARY AND SECONDARY VARIABLES 14
3.1 Primary variable 15
3.2 Secondary variables 15
3.2.1 Total number of (first and recurrent) hospitalisations for HF and CV death 16
3.2.2 Change from baseline at 8 months in the KCCQ total symptom score 16
3.2.3 Proportion of patients with worsened NYHA class at 8 months 17
3.2.4 Death from any cause 17
3.3 Safety variables 17
3.4 Laboratory values and vital signs 18
3.4.1 Baseline laboratory values and vital signs 18
4 ANALYSIS METHODS 18
4.1 General principles 18
4.1.1 Estimand for primary and secondary outcomes 19
4.1.2 Hypotheses 19
4.1.3 Confirmatory testing procedure 20
4.1.4 Presentation of time-to-event analyses 20
4.1.5 Vital status and follow-up of endpoints 20
4.2 Analysis methods 21
4.2.1 Demographics and baseline characteristics 21
4.2.2 Concomitant and baseline medication 21
4.2.3 Analysis of the primary efficacy variable 22

4.2.3.1 Subgroup analysis of the primary endpoint ... 22
4.2.3.2 Sensitivity analysis of the primary endpoint ... 24
4.2.4 Analysis of the secondary efficacy variables ... 24
4.2.4.1 Analysis of recurrent HF events and CV death ... 24
4.2.4.2 Analysis of change from baseline to 8 months in the KCCQ total symptom score ... 25
4.2.4.3 Analysis of worsened NYHA class from baseline to 8 months ... 29
4.2.4.4 Analysis of all-cause mortality ... 29
4.2.5 Analysis of safety variables ... 29
4.2.5.1 Adverse events ... 30
4.2.5.2 Serious adverse events ... 30
4.2.5.3 Adverse events leading to discontinuation or interruption of IP ... 30
4.2.5.4 Amputations and preceding events ... 31
4.2.5.5 Laboratory evaluation and vital signs ... 31
4.2.6 Analysis of exploratory objectives ... 31
5 INTERIM ANALYSES ... 32
6 CHANGES OF ANALYSIS FROM PROTOCOL ... 33
7 REFERENCES ... 34

LIST OF ABBREVIATIONS

Abbreviation or special term	Explanation
AE	Adverse event
ATC	Anatomical Therapeutic Chemical
BP	Blood pressure
CDF	Cumulative distribution function
CEA	Clinical event adjudication
CKD-EPI	Chronic kidney disease epidemiology collaboration equation
CMH	Cochran-Mantel-Haenszel test
CV	Cardiovascular
DAE	Adverse events leading to discontinuation of investigational product
DKA	Diabetic Ketoacidosis
DMC	Data monitoring committee
eCRF	Electronic case report form
eGFR	Estimated glomerular filtration rate
EQ- 5D-5L	EuroQol five-dimensional five-level questionnaire
FAS	Full analysis set
HF	Heart failure
HFpEF	Heart Failure with Preserved Ejection Fraction
HR	Hazard ratio
IP	Investigational Product (dapagliflozin or matching placebo)
ITT	Intention to treat
IxRS	Interactive Voice/Web Response System
KCCQ	Kansas City Cardiomyopathy Questionnaire
KM	Kaplan-Meier
LTFU	Lost to follow-up
LVEF	Left ventricular ejection fraction
MAR	Missing at random
MedDRA	Medical dictionary for regulatory activities
NYHA	New York Heart Association
PACD	Primary analysis censoring date
PGIS	Patient global impression of severity
PT	MedDRA preferred term
PTDV	Premature treatment discontinuation visit

Abbreviation or special term	Explanation
AE	Adverse event
ATC	Anatomical Therapeutic Chemical
SAE	Serious adverse event
SCV	Study Closure Visit
SOC	MedDRA system organ class
T2D	Type 2 diabetes
TSS	KCCQ total symptom score
WoC	Withdrawal of consent

AMENDMENT HISTORY

Date	Brief description of change
<< >>	<< >>
	N/A

1 STUDY DETAILS

1.1 Study objectives

1.1.1 Primary objective

Primary objective:	Endpoint/variable:
To determine whether dapagliflozin is superior to placebo, when added to standard of care, in reducing the composite of CV death and HF events (hospitalisation for HF or urgent HF visit) in patients with HF and preserved systolic function.	Time to the first occurrence of any of the components of this composite:<br>1. CV death<br>2. Hospitalisation for HF<br>3. Urgent HF visit (e.g., emergency department or outpatient visit)

1.1.2 Secondary objectives

**Secondary objective:**	**Endpoint/variable:**
To determine whether dapagliflozin is superior to placebo in reducing the total number of recurrent HF hospitalisations and CV death	Total number of (first and recurrent) hospitalisations for HF and CV death
To determine whether dapagliflozin is superior to placebo in improving Patient Reported Outcomes measured by KCCQ	Change from baseline in the total symptom score (TSS) of the KCCQ at 8 months
To determine whether dapagliflozin is superior to placebo in reducing the proportion of patients with worsened NYHA class	Proportion of patients with worsened NYHA class from baseline to 8 months
To determine whether dapagliflozin is superior to placebo in reducing all-cause mortality	Time to the occurrence of death from any cause

1.1.3 Safety objectives

**Safety Objective:**	**Outcome Measure :**
To evaluate the safety and tolerability of dapagliflozin compared to placebo in patients with HFpEF	• Serious adverse events (SAEs)<br>• Adverse events leading to treatment discontinuation (DAEs)<br>• Amputations, adverse events (AEs) leading to amputation and potential risk factor AEs for amputations affecting lower limbs

1.1.4 Exploratory objectives

Exploratory Objective:	Endpoint/Variable:
To determine whether dapagliflozin is superior to placebo in reducing all-cause hospitalisation	Time to the first occurrence of hospitalisation from any cause
To compare the effect of dapagliflozin versus placebo on health status assessed by EuroQol five-dimensional five-level questionnaire (EQ- 5D-5L) to support health economic analysis and health technology assessment	Changes in health status measured by EQ-5D-5L
To compare the effect of dapagliflozin versus placebo on health status assessed by Patient global impression of severity (PGIS) questionnaires	Changes in health status measured by PGIS
To determine whether dapagliflozin compared with placebo will have an effect on systolic BP	Change in systolic BP from baseline
To determine whether dapagliflozin compared with placebo will have an effect on body weight	Change in body weight from baseline
To determine whether dapagliflozin compared with placebo will have an effect on eGFR	Change in eGFR from baseline
To explore whether dapagliflozin compared to placebo improves KCCQ summary scores, subscores of TSS (Symptom frequency and symptom burden) and domains	Change in Clinical summary score, TSS sub-scores, Overall summary score, QoL score
To collect and store blood samples for future exploratory genetic research	Not applicable. Results will be reported separately

1.2 Definitions

1.2.1 Primary analysis censoring date

The executive committee and AstraZeneca will monitor the accrual of endpoint events and when appropriate define the primary analysis censoring date (PACD) at which time at least the pre-defined target number of 844 events for the primary composite endpoint is expected to have occurred. The study sites will be instructed to plan for study closure visits to be performed after PACD.

Analyses of efficacy endpoint events will include events with onset on or prior to PACD. Event free patients who have not been prematurely censored due to incomplete information (see Section 3.1) will be censored at PACD. HF events and deaths with onset after PACD will also be adjudicated.

1.2.2 Withdrawal of consent

Withdrawal of consent (WoC) should only occur if the patient has received appropriate information about options for modified study follow-up and does not agree to any kind of further assessment or follow-up. Information regarding vital status (dead or alive) at the end of the study will be collected from public sources, to be included in the analysis of death from any cause as a sole outcome and in patient disposition summaries.

1.2.3 Discontinuation from study drug

Discontinuation from study drug does not mean discontinuation from study follow-up or WoC. Patients who discontinue from study drug should continue study visits according to plan until study closure. If the patient does not agree to this approach, modified follow-up capturing the essential information for the objectives of the study should be arranged. Data will be included in the ITT analyses irrespective of whether the event occurred before or following discontinuation of study drug.

1.2.4 Vital Status

Known vital status at the end of the study will be defined when the patient is dead or has date last know alive on or after the PACD.

For patients who have withdrawn consent, the investigator will attempt to collect vital status from publicly available sources at study closure in compliance with local privacy laws/practices.

1.2.5 Lost to follow-up

The term lost to follow-up (LTFU) will be limited to patients with unknown vital status at the end of the study as defined in section 1.2.4. Other measures will be used to describe completeness of follow-up of the primary endpoint (section 4.1.5)

1.3 Study design

This is an international, multicentre, parallel-group, event-driven, randomised, double-blind, placebo-controlled study in patients with heart failure with preserved ejection fraction (HFpEF), evaluating the effect of dapagliflozin 10 mg versus placebo, given once daily in addition to background regional standard of care therapy, including treatments to control co-morbidities, in reducing the composite of CV death or heart failure events.

HFpEF is defined for the purposes of this study as left ventricular ejection fraction (LVEF) >40% and evidence of structural heart disease. Adult patients with HFpEF, aged ≥40 years and with NYHA class II-IV will be randomised in a 1:1 ratio to receive either dapagliflozin 10 mg or placebo once daily. A proportion of patients, here denoted as the subacute group, will be randomised during hospitalisation for heart failure or within 21 days of discharge from hospitalisation for heart failure.

It is estimated that approximately 8000 patients at approximately 400-500 sites in 20-25 countries will be enrolled to reach the target of approximately 4700 randomised patients.

In this event driven trial, study closure procedures will be initiated when the predetermined number of primary endpoints are predicted to have occurred (n=844), i.e. the PACD (section 1.2.1 and Figure 1). Patients should be scheduled for a Study Closure Visit (SCV) within 6 weeks of the PACD. The anticipated total study duration is approximately 33 months dependent on randomisation rate and event rate. The number of patients randomised, the study duration, or both, may be changed if the randomisation rate or the event rate is different than anticipated.

Figure 1 Study design

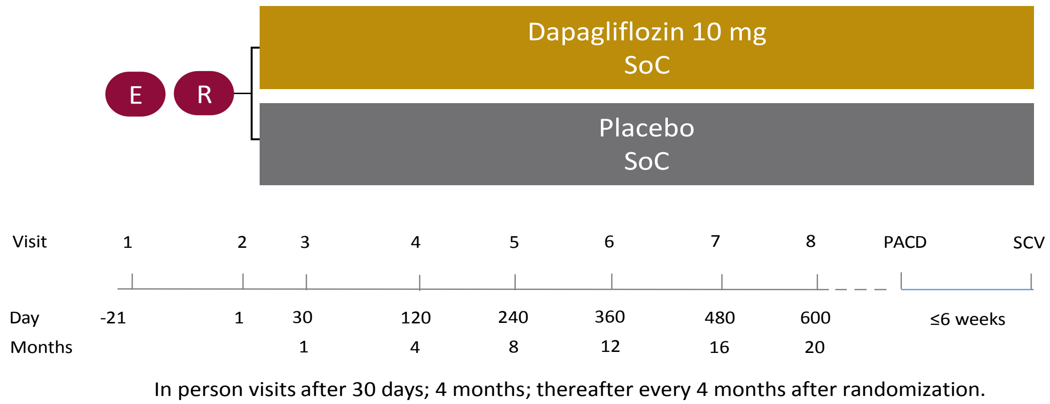

E=Enrolment; R=Randomization; SoC= Standard of Care; PACD=Primary Analysis Censoring Date; SCV=Study Closure Visit; FU=Follow Up

1.3.1 Randomisation

Patients will be randomised 1:1 to either dapagliflozin 10 mg or placebo once daily. The treatment allocation in this study will be double-blind. Randomisation will be stratified by Type 2 diabetes (T2D) status at randomisation (2 levels: with T2D; without T2D). For the purpose of stratification, T2D is defined as established diagnosis of T2D or HbA1c ≥ 6.5% (48 mmol/mol) at enrolment (visit 1; single measure) central laboratory test.

Randomisation will be performed in balanced blocks of fixed size. The randomisation codes will be computer generated and loaded into the IxRS (Interactive Voice/Web Response System) database.

The number of randomised patients with T2D will be monitored in order to ensure a minimum of 30% patients in each group of patients with and without T2D. Randomisation may be capped (i.e., no more patients can be randomised in a specific sub-population) if the pre-determined limit is reached.

Randomisation of patients based on geographic region will be monitored to ensure global representation. LVEF value, NYHA class, subacute/non-subacute group, and atrial fibrillation status at visit 1 may be capped in IxRS to avoid over- or under-representation of these patient subgroups.

1.4 Number of subjects

The primary objective of the study is to determine the superiority of dapagliflozin versus placebo added to standard of care in reducing the composite of CV death and heart failure events (hospitalisation for HF or urgent HF visit). Assuming a true hazard ratio (HR) of 0.80 between dapagliflozin and placebo, using a two-sided alpha of 5%, 844 primary endpoint events will provide a statistical power of 90% for the test of the primary composite endpoint. This is based on an overall 1:1 allocation between dapagliflozin and placebo.

The HR was chosen as a conservative assumption based on the observed HR 0.72 (95% confidence interval 0.50-1.04) for the composite of HF hospitalisation and CV death in patients with HF at baseline in the EMPA-REG OUTCOME trial (Fitchett et al 2016) and HR 0.61 (0.46-0.80) for patients with history of HF in the CANVAS program (Rådholm et al 2018) considering that these were post-hoc analyses in subgroups with limited documentation of baseline HF diagnosis, not characterised by ejection fraction.

The event rate assumptions are based on sub analyses of the TOPCAT and I-PRESERVE studies by geographic region, NT-proBNP levels, prior hospitalisation for HF, and T2D status (Pfeffer et al 2015, Kristensen et al 2015 Kristensen et al 2017). The sample size calculation builds on the assumption of an annual event rate of 9% in the placebo group for the majority of prevalent HFpEF patients, importantly all with NT-proBNP ≥300 pg/ml by inclusion criterion. Additionally, a subgroup of patients due to be discharged or recently discharged from a HF hospitalisation (here denoted 'subacute' patients) with a higher event rate is planned to be included. Assuming 20% of patients from the subacute category with an annual event rate of 24% during the first year and 9% thereafter for the remainder of the study, (corresponding to an annualised rate of approximately 17% for sub-acute patients), approximately 4700 patients are estimated to provide the required number of 844 patients with a primary event during an anticipated recruitment period of 18 months and a minimal follow-up period of 15 months (total study duration 33 months, average follow-up 24 months). The study is event driven and the number of patients or duration may change if the event rate is lower or higher than anticipated.

In addition, the expected number of patients who will be lost to follow-up is expected to be small; hence, these are not considered in the determination of the sample size.

2 ANALYSIS SETS

2.1 Definition of analysis sets

2.1.1 Full analysis set

All patients who have been randomised to study treatment will be included in the full analysis set (FAS) irrespective of their protocol adherence and continued participation in the study. Patients will be analysed according to their randomised IP assignment, irrespective of the treatment actually received. The FAS will be considered the primary analysis set for the intention to treat (ITT) analysis of primary and secondary variables and for the exploratory efficacy variables.

2.1.2 Safety analysis set

All randomised patients who received at least 1 dose of randomised treatment will be included in the safety analysis set. Patients will be analysed according to the treatment actually received. For any patients given incorrect treatment, ie randomised to one of the treatment groups, but actually given the other treatment, the treatment group will be allocated as follows: Patients who got both incorrect and correct treatment will be analysed according to their randomised treatment. Patients who got only the incorrect treatment will be analysed according to that treatment.

The Safety analysis set will be considered the primary analysis set for all safety variables.

2.2 Violations and deviations

The important protocol deviations listed below will be summarised by randomised treatment group

- Patients who were randomised but did not meet inclusion and exclusion criteria
- Patients who received the wrong study treatment at any time during the study.
- Patients who received prohibited concomitant medication, which for this study is limited to open label SGLT2 inhibitors taken in combination with IP.

As the primary analysis is ITT analysis, protocol deviation will not imply exclusion from the primary analysis.

3 PRIMARY AND SECONDARY VARIABLES

Deaths and potential HF events will be adjudicated by an independent clinical event adjudication (CEA) committee. The CEA committee members will not have access to the

treatment codes for any patient. The CEA procedures and event definitions will be described in the CEA charter according to the CDISC definitions (Hicks et al 2018).

Only HF hospitalisations and urgent HF visits confirmed by the CEA will be used in the analysis of the primary and secondary endpoints and their components.

The primary analyses of the endpoints concerning CV deaths, either as a component of a composite or on its own, will include deaths adjudicated as CV cause. Deaths adjudicated as 'cause undetermined' will be considered as non-CV deaths in these analyses.

Adjudicated events occurring from randomisation until WoC or PACD will be included in the analysis of primary and first secondary endpoint. The analysis of all-cause death as a sole outcome will in addition include any deaths (not adjudicated) after WoC, but on or before PACD.

3.1 Primary variable

The primary efficacy variable is time from randomisation to the first occurrence of any event in the composite of CV death, hospitalisation for HF or an urgent HF visit.

Patients who did not have an adjudicated primary endpoint event on or prior to PACD will be censored at the earliest of date of WoC or non-CV death when applicable, and otherwise at the date of the last clinical event assessment or the PACD, whichever occurs first. It is expected that patients alive and under study follow-up will have a clinical event assessment at their SCV after PACD. Last clinical event assessment is defined as the last date when the event assessment question for a potential heart failure event was completed on the eCRF event assessment page.

In analysis of the individual components hospitalisation for HF and urgent HF visit, to examine their contribution to the composite endpoint, date of death from any cause will be an additional point of censoring.

For analysis of time to first event, data will be expressed as two variables:

- A binary variable indicating whether the event in question occurred, or the patient was censored.
- An integer variable for the number of days from randomisation to the first occurrence of an event (start date of the event – randomisation date + 1), or for event free patients, from randomisation to censoring (censoring date – randomisation date + 1).

3.2 Secondary variables

The secondary endpoints are included in a hierarchical testing sequence following the primary endpoint as described in section 4.1.3.

3.2.1 Total number of (first and recurrent) hospitalisations for HF and CV death

The first secondary endpoint is the total number of first and recurrent hospitalisations for HF and CV death, not including urgent HF visit.

For the analysis of recurrent heart failure hospitalisation and CV death, the data will be expressed in counting process style for input to the analysis as described in Section 4.2.4.14.2.4 , as follows. The time from randomisation to end of follow-up/censoring will be split into one or more interval with variables for start of interval, end of interval and a variable indicating if an event occurred at the end of each respective interval, or if the patient was censored.

Patients who did not have the endpoint will be censored by the same rule as for the primary endpoint.

3.2.2 Change from baseline at 8 months in the KCCQ total symptom score

The efficacy variable is the change from baseline at 8 months of the Kansas City Cardiomyopathy Questionnaire (KCCQ) total symptom score (TSS).

The KCCQ is a self-administered disease specific instrument for patients with HF (Green et al 2000, Spertus et al 2005). The KCCQ consists of 23 items measuring HF-related symptoms, physical limitations, social limitations, self-efficacy, and health-related quality of life. The TSS incorporates the symptom burden and symptom frequency domains into a single score. Scores are transformed to a range of 0-100. Higher scores represent better outcomes.

Baseline is defined as the value at randomisation visit (visit 2). Change from baseline at each post-baseline analysis time point will be calculated as the value at the corresponding post-baseline analysis time point minus the baseline value. The KCCQ is assessed by the patient at randomisation, at the visits targeted 1, 4 and 8 months following randomisation and at premature treatment discontinuation visit (PTDV) and SCV. By the ITT principle, the analysis will include all data irrespective of whether the patient has discontinued study drug.

In order to account for patients who die prior to the 8-month assessment and to accommodate non-normal distribution of KCCQ scores, a composite rank-based endpoint will be used. The values of change from baseline to 8 months in TSS of patients who survive to 8 months will be converted to ranks (across both treatment groups combined) with lower ranks attributed to worse outcomes (i.e., lower ranks corresponding to negative or smaller values of change from baseline). Patients who die prior to the 8-month assessment will be assigned the worst rank, i.e., worse than any patient surviving to 8 months. All patients deceased prior to the 8-month assessment will be assigned the same worst rank regardless of the relative timing of their

deaths. This is done to reduce the impact of treatment differences in time to CV death on the assessment of this KCCQ secondary endpoint.

3.2.3 Proportion of patients with worsened NYHA class at 8 months

The efficacy variable is the proportion of patients with worsened NYHA class from baseline to 8 months.

The NYHA classification will be evaluated by the investigator and collected in eCRF at enrolment and randomisation visits, at 1, 4 and 8 months visits, at PTDV and SCV. Baseline is defined as the value at randomisation (visit 2). The analysis will include all data irrespective of whether the patient has discontinued study drug.

For the primary analysis the data will be dichotomised into patients with worsened NYHA class at 8 months (the NYHA class is higher than baseline), including patients who died due to any cause prior to 8 months, versus other patients with improved or unchanged class compared to baseline.

3.2.4 Death from any cause

The efficacy variable is time to from randomisation to death from any cause. All deaths on or prior to PACD, including any deaths after WoC, will be included. Patients who are alive will be censored at the earliest of date last known alive and PACD.

3.3 Safety variables

The safety and tolerability of dapagliflozin in patients with HFpEF will be evaluated from serious adverse events (SAEs), adverse events leading discontinuation of IP (DAEs), adverse events(AE) leading to amputation and AEs reflecting potential risk factors for lower limb amputations (“preceding events”).

In addition to amputation, non-serious and serious events potentially placing the patient at risk for a lower limb amputation, in this document denoted “preceding events”, should also be recorded in the eCRF as AE/SAE, whether or not an amputation has taken place. Preceding events will be defined for analysis by a predefined list of preferred terms. Additional information about amputations with underlying conditions and preceding events will be collected on dedicated eCRF pages.

SAEs will be collected from time of informed consent until and including the patent’s last visit. Non-serious AEs will be collected from randomisation until and including the patient’s last visit. Collection of non-serious AEs is limited to AE leading to amputation, preceding events, AEs leading to a potential endpoint, DAEs and AEs which are the reason for interruption of study drug.

Efficacy endpoints (deaths and potential HF events) will be adjudicated. These events will be recorded as SAEs in the database, but will not be reported as SAEs to health authorities to avoid unnecessary unblinding. However, if it is determined by the CEA committee that a potential endpoint does not meet the endpoint criteria, the event will be reported to AZ patient safety data entry site and if applicable to the health authorities.

For SAEs or DAEs reported by the Investigator as Diabetic Ketoacidosis (DKA) additional information will be recorded on specific eCRF pages in addition to the AE/SAE form.

For myocardial infarctions, unstable angina and stroke additional information will be recorded on specific eCRF pages in addition to the AE/SAE form.

3.4 Laboratory values and vital signs

Blood samples will be taken for central laboratory assessment of creatinine and calculation of eGFR at enrolment visit, at the visits targeted 1, 4 months and 12 months following randomisation, then annually and at PTDV and SCV. eGFR will be calculated (in mL/min/1.73 m^2) using the CKD-EPI formula (Levey at al 2009).

Central laboratory assessment of NT-proBNP and HbA1c will be taken at visit 1.

Systolic blood pressure (SBP), diastolic blood pressure (DBP) and pulse rate will be measured at visit 1, visit 2, at 1 and 12 months visit, then annually and at PTDV and SCV.

Weight will be measured at visit 1, at the 12 months visit, then annually and at PTDV and SCV.

3.4.1 Baseline laboratory values and vital signs

In principle baseline will be defined as the last value on or prior to date of first dose of randomised study drug, or for patients who did not receive treatment, the last value on or prior to date of randomisation. Except for cases of rescreening this will be visit 1 measurement of weight, NT-proBNP, eGFR and HbA1c, and visit 2 measurement of SBP, DBP and pulse rate.

4 ANALYSIS METHODS

4.1 General principles

No multiplicity adjustment will be made to confidence intervals as they will be interpreted descriptively and used as a measure of precision. All p-values will be unadjusted. P-values for variables not included in the confirmatory testing sequence, or following a non-significant test in the sequence will be regarded as nominal.

Primary and secondary analyses of HF events and death include adjudicated events occurring on or prior to PACD.

Stratification of analyses for T2D status will be performed using the stratification values as entered in IxRS to determine the randomisation assignment.

Incomplete dates
If only the year part of a date is available (YY), then the date will be set to YY0701. If only the year and month is available (YYMM), then the date will be set to YYMM15. Additional imputation rules will be defined as appropriate to ensure that eg, dates will not be imputed as prior to randomisation, after death or start date after end date.

Study drug compliance
The percentage of study drug compliance for the overall treatment period will be derived for each patient based on pill counts as the number of pills taken (dispensed – returned), relative to the expected number of pills taken. The expected number of pills taken is defined as 1*(date of last dose – date of first dose +1), excluding days of interruption.

Study drug compliance will be presented descriptively, including mean, median, quartiles and 5% and 95% percentiles.

4.1.1 Estimand for primary and secondary outcomes

The primary and secondary event based objectives will be evaluated under the treatment policy estimand including differences in outcomes over the entire study period until PACD to reflect the effect of the initially assigned randomised study drug, irrespective of exposure to study drug, concomitant treatment as well as subsequent treatment after discontinuation of study drug. The analysis will be performed for the full analysis set including all events that occurred on or prior to PACD, including events following premature discontinuation of study drug. The time-to-first event analysis by Cox proportional hazards regression and the analysis of recurrent events (Section 4.2.4) assume that missing data is at random.

4.1.2 Hypotheses

To control the overall type I error rate at 5% two-sided, the significance level will be adjusted for interim analysis of efficacy performed by the DMC (Section 5) using the Haybittle-Peto function implemented in the software East (Copyright © Cytel Inc) . For one planned interim analysis including 67% of the target number of primary endpoints, the significance level will be 4.980%. The following null hypothesis will be tested for the primary endpoint

H0: HR [dapagliflozin:placebo] =1

versus the alternative hypothesis

H1: HR [dapagliflozin:placebo] ≠1

The secondary endpoints included in confirmatory statistical testing using a closed testing procedure (section 4.1.3) will be based on similar two-sided alternative hypotheses for the respective treatment difference.

4.1.3 Confirmatory testing procedure

A closed testing procedure including a pre-specified hierarchical ordering of the primary and secondary endpoints will be utilised. The Type I error will be controlled at an overall two-sided 5% level for multiplicity across primary and secondary endpoints and in consideration of the planned interim analysis. With one interim analysis at 67% of events the two-sided significance level in final analysis, α, will be 4.980%. Statistical significance will be assessed in the pre-specified order of the endpoints as specified in section 1.1.1 and section 1.1.2 . If the primary endpoint is significant at level α, then the first secondary endpoint, recurrent HF hospitalisations and CV death, will be tested at level α. If the first secondary endpoint is significant, then the α will be split between KCCQ total symptom score and NYHA class. If one of them is significant at level α/2, then the other can be tested at level α. If both KCCQ and NYHA class reach statistical significance, then all-cause mortality will be tested at significance level α.

If the study is stopped in the efficacy interim analysis (section 5), testing of secondary endpoints will be performed with the same testing procedure as described in this section above with a two-sided α= 0.002.

4.1.4 Presentation of time-to-event analyses

In general, summary tables of time-to-event analyses will include the number and percent of patients with event per treatment group, event rate, hazard ratio with 95% confidence interval and p-value. The event rate will be derived as the number of patients with event divided by the total duration of follow-up across all patients in the given group.

Kaplan-Meier (KM) estimates of the cumulative proportion of patients with events will be calculated and plotted per treatment group, with the number of patients at risk indicated below the plot at specific time points. The KM plots will be presented for all time to event analyses, including the individual components of the composite endpoints.

4.1.5 Vital status and follow-up of endpoints

Potential HF endpoints and deaths will be collected and adjudicated from randomisation throughout the study until and including the patient's last visit. The investigator will attempt to collect vital status (dead or alive) at the end of the study for all patients, including vital status from publicly available sources for patients who have withdrawn consent, in compliance with local privacy laws/practices.

Known vital status at the end of the study will be defined when the patient is dead or has date last know alive on or after the PACD. In patient disposition the number of patients who are dead, alive or with unknown vital status will be reported separately for patients who did/did not withdraw consent. The term lost to follow-up (LTFU) will be limited to only patients with unknown vital status.

Follow-up of the primary endpoint will be defined in terms of completion of the event assessment question for a potential HF event as described for censoring in section 3.1. Thus, a patient that is not LTFU, ie with known vital status, may have incomplete follow-up of endpoints.

Complete follow up of the primary endpoint will be defined when the patient had a primary endpoint event, died from non-CV death or had complete event assessment on or after the PACD (ie, the patient was not censored du to incomplete follow-up of endpoints).

In addition to the number and percent of patients with complete follow-up, the proportion of total patient time with complete follow-up will be reported per treatment group.
Patient time with complete follow-up will be defined as time from randomisation until the earliest of first primary endpoint event, death, WoC, censoring where last complete event assessment is prior to PACD or PACD. The denominator, representing maximum complete follow-up, will be the time to first primary endpoint event, death or PACD.

4.2 Analysis methods

4.2.1 Demographics and baseline characteristics

Demographic and baseline characteristics, including medical history, will be summarized, using frequency distributions and summary statistics based on the FAS, for each treatment group as well as for all patients combined. No statistical test will be performed for comparison of any baseline measurement among treatment groups.

4.2.2 Concomitant and baseline medication

Baseline medication is defined as medication with at least one dose taken before date of randomisation and with no stop date before date of randomisation.

Concomitant medication is defined as medications taken post randomisation, irrespective of study drug.

The frequency of baseline and concomitant medication will be presented for the FAS per ATC class and treatment group. Summaries of prohibited medication, in this study limited to SGLT2 inhibitor taken while on IP, will be presented.

4.2.3 Analysis of the primary efficacy variable

The primary variable is the time to first event included in the primary composite endpoint. The primary analysis will be based on the ITT principle using the FAS, including events with onset on or prior to PACD, adjudicated and confirmed by the CEA committee.

In the analysis of the primary composite endpoint, treatments (dapagliflozin versus placebo) will be compared using a Cox proportional hazards model with a factor for treatment group, stratified by T2D status at randomisation. The analysis will use WoC, non-CV death, last clinical event assessment and PACD for censoring of patients without any primary event as described in Section 3.1. The Efron method for ties and p-value based on the score statistic will be used. Event rates, p-value, HR, and 95% confidence interval will be reported.

The contribution of each component of the primary composite endpoint to the overall treatment effect will be examined. In the analysis of the components, all first event of the given type will be included irrespective of any preceding non-fatal composite event of a different type. Consequently, the sum of the number of patients with individual events in the component analysis will be larger than the number of patients with a composite outcome. Methods similar to those described for the primary analysis will be used to separately analyze the time from randomisation to the first occurrence of each component of the primary composite endpoint.

Kaplan-Meier estimates of the cumulative proportion of patients with event will be calculated and plotted, for the composite endpoint and for the individual components.

4.2.3.1 Subgroup analysis of the primary endpoint

Exploratory subgroup analyses of the primary composite endpoint will be performed for the characteristics listed in Table 1. Cox proportional hazard model stratified for T2D with factors for treatment group, the subgroup variable and the interaction between treatment and subgroup will be used to examine treatment effects within relevant subgroups separately. In addition to the number and percent of patients with event, event rate estimate, HR with 95% confidence interval and p-value for each subgroup, the interaction p-value will be presented. HRs with confidence interval will be presented in a forest plot, also including the event rate and interaction p-value. The p-values for the subgroup analyses and interaction will not be adjusted for multiple comparisons as the tests are exploratory and will be interpreted descriptively.

Table 1 Characteristics and categories for sub group analysis of the primary endpoint

**Characteristic**	**Categories**
Age (years)	<= median, > median

Sex	Male, female
Race	White, Black or African, Asian, Other
Geographic region	Asia (China, Japan, Taiwan, Vietnam)<br>Europe and Saudi Arabia (Belgium, Bulgaria, Czech Republic, France, Hungary, Netherlands, Poland, Romania, Russia, Saudi Arabia, Spain)<br>North America (Canada, US)<br>Latin America (Argentina, Brazil, Mexico, Peru)
NYHA class at enrolment	II, III/IV
LVEF at enrollment (%)	41-49, ≥50
NT-proBNP at enrollment (pg/ml)	<= median, > median
Randomised during hospitalisation for HF or within 21 days of discharge.	Yes, No
eGFR at enrolment (ml/min/1.73m^2)	<60, ≥60
BMI at enrolment (kg/m2)	<30, ≥30
Type 2 diabetes at enrolment*	Yes, No
Systolic blood pressure at randomisation	<= median, > median
Atrial fibrillation or flutter at enrolment ECG	Yes, No

* The subgroup analysis by T2D status will be based on eCRF medical history record and exclude T2D as a stratification factor from the model.

The subgroup analyses will be repeated for the CV death component of the primary composite endpoint.

4.2.3.2 Sensitivity analysis of the primary endpoint

Undetermined cause of death

A sensitivity analysis of the primary analysis where deaths adjudicated as 'undetermined' cause are considered as CV deaths and included as endpoint events will be performed.

Missing data and informative censoring

The time-to-event analysis using the Cox regression depends on the assumption of non-informative or ignorable censoring, corresponding to the missing-at-random assumption. The missing data in this context are patients who are prematurely censored due to WoC, LTFU or otherwise incomplete follow-up of endpoints. The amount of missing data will be described eg, in terms of the number of patients and patient time with incomplete follow-up as described in Section 4.1.5.

Patient retention and follow-up are at the forefront of study planning and conduct, and the amount of incomplete follow-up is expected to be small. To assess the impact of missing data and the robustness of the results with regard to the assumption of non-informative censoring, sensitivity analysis will be planned based on the evaluation of the missing follow-up and discussed in relation to the observed efficacy signal. This may include analysis where scenarios in terms of increased risk in censored patients are explored to identify a 'tipping point' where statistical significance would be lost.

4.2.4 Analysis of the secondary efficacy variables

4.2.4.1 Analysis of recurrent HF events and CV death

The composite outcome of recurrent HF hospitalizations andr CV death will be analysed by the semi-parametric proportional rates model (Lin et al 2000; known as the LWYY method) to test the treatment effect and to quantify the treatment difference in terms of the rate ratio with 95% confidence interval and p-value.

In addition, the two components in the composite endpoint (total HF hospitalizations and CV death) will be analysed separately to quantify the respective treatment effects and check the consistency between the composite and the components. For the analysis of total HF hospitalizations component, occurrence of CV death can be regarded as semi-competing risk (informative censoring) and may introduce a bias in the treatment effect estimate for HF hospitalizations (dilution of effect size if the drug has a positive effect on both components). To address this concern and to account for the correlation between the two components, the joint modelling (frailty model) approach (Rogers et al 2016) will be used for the component analyses. Non-parametric estimates of HF hospitalization rates over time allowing for death as terminal event will be provided as well (Ghosh and Lin 2000).

4.2.4.2 Analysis of change from baseline to 8 months in the KCCQ total symptom score

Hypothesis testing

The composite rank-based endpoint representing the patients' vital status at 8 months and the change from baseline to 8 months in TSS in surviving patients, as defined in Section 3.2.2, will be analysed using the rank ANCOVA method (Stokes et al 2012) to test the null hypothesis of no differences in the distributions of ranked outcomes between the two treatment groups. Analysis will be stratified by T2D status at randomisation, and adjusted for the baseline TSS value as follows.

First the change from baseline to 8 months in TSS and vital status at 8 months, as well as values of the baseline TSS covariate will be transformed to standardized ranks within each T2D randomization stratum, using fractional ranks and mean method for ties. Ranking for the composite endpoint will be done so that patients who died prior to the 8-month assessment are assigned the worst ranks within each stratum. This will be implemented by assigning a temporary value of -101 to subjects who died prior to 8-month assessment before deriving fractional ranks. Then, separate regression models will be fit to the ranked data for each randomization stratum using a regression model for the ranked composite variable as dependent variable, adjusting for the ranked baseline covariate. Residuals from this regression model will be captured for further testing of differences between treatment groups. The Cochran-Mantel-Haenszel (CMH) test, stratified for the T2D status at randomization, using the values of the residuals as scores will be used to compare treatment groups.

The p-value from the CMH test of treatment effect at 8 months will be the used for the confirmatory testing of the secondary endpoint in the multiple testing procedure described in section 4.1.3.

Estimation of treatment effect

<u>Win ratio:</u>

For a summary statistic that uses the same ranking as that used in the hypothesis test, but has a clinical interpretation, the win ratio (WR) and the corresponding 95% confidence interval (Wang and Pocock 2016) will be reported. It is noted that the WR differs from the statistic used for hypothesis testing, so that exact consistency is not expected as between these two analyses, e.g. on rare occasions, the confidence interval for WR could exclude unity while the pre-planned hypothesis test could be non-significant, or the hypothesis test could be significant with the confidence interval for WR including unity. Formal inference for the superiority of the treatment over control will be made only from the preplanned hypothesis test.

The win ratio represents the odds of having a more favourable outcome versus a less favourable outcome when assigned to the dapagliflozin 10 mg treatment group as opposed to

placebo. More specifically, each patient in the dapagliflozin group is compared with each patient in the placebo group and each pair is labelled as “winner”, “loser”, or “tie”, depending on whether the patient on dapagliflozin has a more favourable, less favourable, or the same outcome, respectively, with respect to the composite ranked endpoint compared to the patient on placebo. Win ratio is defined as the ratio of the number of “winner” pairs to the number of “looser” pairs for the dapagliflozin arm. If the estimated win ratio is greater than 1 then the treatment effect is in favour of dapagliflozin.

The win ratio statistic adjusted for the randomization stratification factor and baseline TSS will be obtained using the methodology in (Kawaguchi et al 2011) for the stratified Mann-Whitney estimators for the comparison of two treatments with randomization based covariance adjustment. The win ratio statistic will be calculated as Mann-Whitney odds, i.e., WR=MW⁄((1-MW)), where MW is the adjusted Mann-Whitney estimate. The 95% confidence interval for the win ratio will be obtained as

$$\exp\{\ln(WR) \pm 1.96 * SE(\ln(WR))\}$$

where the standard error of the logarithm of WR is obtained as

$$SE(\ln(WR)) = SE(MW)/(MW * (1 - MW))$$

and the $SE(MW)$ is the standard error of the adjusted Mann-Whitney estimate. The adjusted Mann-Whitney estimates and its standard error will be obtained using the “sanon” package in R (Kawaguchi and Koch 2015).

Responder analysis:

Number and percentage of patients in each treatment group will be summarized across the following categories:

5 point improvement from baseline to 8 months in TSS vs no significant improvement:
- Change from baseline in TSS ≥ 5 points, vs
- Death prior to the 8 months assessment or change from baseline in TSS < 5 points.

5 point deterioration from baseline to 8 months in TSS vs no significant deterioration:
- Death prior to the 8 months assessment or change from baseline in TSS ≤ -5 points, vs
- Change from baseline to 8 months in TSS > -5 points.

Cumulative distribution function (CDF) plots will be presented by treatment group to summarize the distribution of change from baseline to 8 months in TSS values, where patients who die prior to the 8-month assessment will be represented with the value of -101 (a value below the worst possible change from baseline).

Handling of missing data

The number of patients with missing vital status at 8 months is expected to be negligible. If some patients are LTFU or patients who withdrew consent have unknown vital status, the main analysis will be done with these patients assigned the worst ranks (same as deaths).

In the context of analysing the composite ranked endpoint as described above, missing data may arise when patients miss the 8-month KCCQ assessment while remaining in the study during the 8-month assessment window, or when patients withdraw consent from the study prior to 8 months. If a patient is known to have died prior to the 8-month assessment, the patient is considered to have a non-missing composite outcome and will be handled as described above (assigned the worst rank). Otherwise, patients who are alive at 8 months and have missing baseline or 8-month KCCQ assessments will have their missing TSS imputed using the multiple imputation (MI) methodology as follows.

Missing TSS values at baseline or at 8 months will be imputed under the Missing at Random (MAR) assumption. The imputation will be done using a predictive mean matching multiple imputation model and a method of Fully Conditional Specification as implemented in the SAS Procedure MI (FCS statement). The predictive mean matching method ensures that the imputed values remain in the permissible range of the TSS values. The imputation model will include the treatment group, T2D randomization stratum, TSS at baseline, month 1, 4, and 8, and three auxiliary binary variables representing occurrences of any HF events in the intervals from randomization to 1 month, from 1 to 4 months, and from 4 to 8 months, respectively. Occurrences of HF events will be determined based on the investigator-reported potential HF events. Auxiliary variables related to HF events are included in the imputation model to improve the imputation accuracy, because the occurrence of HF events is associated with quality of life assessed by KCCQ.

The number of closest observations used to sample an imputed value by the predictive mean matching method will be 5 (SAS default setting).

Each imputed dataset will be analysed using the methods described in the “Hypothesis testing” and “Estimation of treatment effect” sub-sections above. The results from multiple imputed datasets will be combined using Rubin’s rule as implemented in the SAS Procedure MIANALYZE.

- In the analysis of rank ANCOVA, the CMH tests statistic used for the hypothesis test has a chi-square distribution. In order to apply Rubin’s combination rule, which assumes approximate normal distribution of the statistics being combined, a normalizing Wilson-Hilferty transformation will be applied to the CMH test statistics from each imputed dataset (Ratitch et al 2013). The standardized transformed statistic will be computed as follows:

$$st_{wh\_cmh}{}^{(m)} = \frac{\sqrt[3]{\frac{cmh^{(m)}}{df}} - \left(1 - \frac{2}{9 \times df}\right)}{\sqrt[2]{\frac{2}{9 \times df}}}$$

where $cmh^{(m)}$is the CMH statistic from the mth imputed dataset and df is the number of degrees of freedom associated with the statistic (in this case equal 1). The transformed statistics are approximately normally distributed with mean of 0 and variance of 1 and can be combined using Rubin's rule.

- For the estimation of the win ratio, a combined Mann-Whitney estimate (MW) and its standard error ($SE(MW)$) will first be obtained by applying Rubin's rule to the corresponding estimates from multiple imputed datasets. Then the win ratio and its 95% confidence interval will be obtained based on the combined Mann-Whitney estimate and its standard error as previously described.

- For the summaries of number and percentage of subjects in the categories of significant improvement and deterioration from baseline as well as CDF plots, as discussed in the "Estimation of treatment effect" sub-section above, the average number and percent of subjects in each category across all multiple imputed datasets will be reported.

Supportive analyses and sensitivity analyses

The number and percent of patients who die prior to the 8-month assessment will be summarized by treatment group.

Descriptive statistics of scores and change from baseline at 1,4, and 8 months and SVC will be presented for total symptom score, overall summary score, clinical summary score and domains (Physical limitation, symptom stability, symptom frequency, symptom burden, quality of life, self efficacy and social limitation).

The testing and estimation described for change from baseline at 8 months in TSS, will be repeated in an exploratory fashion for change from baseline in TSS at 1 and 4 months, and for the overall summary score and clinical summary scores at 1, 4 and 8 months.

To assess the impact on TSS change from baseline of a treatment effect on mortality, an alternative ranking my be applied where patients who die prior to the 8 months assessment will be assigned worse ranks than any patient surviving to 8 months, but among the deceased the relative ranking will be based on their last value of change from baseline in TSS while alive.

4.2.4.3 Analysis of worsened NYHA class from baseline to 8 months

The proportion of patients with worsened (higher) NYHA class at 8 months compared to baseline, including patients who died prior to 8 months in the worsened category, versus patient with improved or unchanged NYHA class, will be analyzed by logistic regression with treatment group, baseline NYHA class and T2D status randomization as factors. The odds ratio between treatment groups and its 95% confidence interval and corresponding two-sided p-value will be presented. Frequencies of NYHA class and change from baseline as well as the odds ratio for treatment effect will be presented for all post baseline visits with scheduled NYHA class evaluation. The p-value for the test of treatment effect at 8 months will be used for the confirmatory testing of the secondary endpoint in the multiple testing procedure described in section 4.1.3.

Missing NYHA assessments will be handled with the same multiple imputation methodology as described above for the analysis of KCCQ TSS in section 4.2.4.2

To assess the impact of a treatment effect of death, a sensitivity analysis will be performed where the last NYHA assessment prior to death will be carried forward.

4.2.4.4 Analysis of all-cause mortality

The 4th secondary endpoint, time to death from any cause will be analysed using Cox regression in the same manner as the primary composite endpoint, with stratification for T2D status at randomisation. The analysis will include deaths occurring on or prior to PACD. Patients who are alive will be censored at PACD, or for any patients who are LTFU, at last date known to be alive.

4.2.5 Analysis of safety variables

Analysis set

For safety analyses, all summaries will be based on the safety analysis set (Section 2.1.2).

Exposure

The total exposure to study drug will be defined as the length of period on study drug, calculated for each patient as date of last dose – date of first dose +1.

An alternative measure where days of interruption are removed will be calculated and termed actual exposure.

Total and actual exposure will be presented descriptively.

Treatment periods

The summaries for the on-treatment period will include events with an onset date on or after first dose of randomized study drug and on or before 30 days after last dose of study drug. Additional presentations will include all events with onset on or after first dose of study drug

regardless of whether patients are on or off study treatment at the time of the event (the 'on +off ' treatment period.). Patients who complete the study on study drug will discontinue treatment on the SCV. Thus there will in general be no events after completion of the study drug period, and censoring of events for on-treatment analysis affects only patients who prematurely and permanently discontinue study drug.

All summaries of AEs described in Section 4.2.5.1 to 4.2.5.4 below will be presented for the on-treatment period. Additional summaries based on the on+off treatment period will be presented for SAEs, amputations and preceding events as defined in Section 3.3.

4.2.5.1 Adverse events

Summaries of AEs will primarily be based on the on-treatment period.

In addition to SAEs, the collection of AEs that are not serious is limited to DAEs, AEs leading to interruption of IP, amputations and preceding events (see section 3.3) . Thus, summaries of AEs will be limited to these categories and general summaries of all non-serious AEs are not planned.

AEs will be classified according to MedDRA by the medical coding team at AstraZeneca data management centre, using the most current version of MedDRA.

Summaries by system organ class (SOC) and preferred term (PT) will be sorted by international order for SOC and by descending order of PT in the dapagliflozin treatment group.

No statistical tests to compare crude AE frequencies between treatment groups are planned.

A summary table of the total number and percent of patients with SAE, DAE, AE leading to temporary interruption, amputations and preceding events per treatment group will be provided.

4.2.5.2 Serious adverse events

SAEs will be presented as described below both on treatment and on+off treatment.

The number and percent of patients with SAEs will be presented by SOC, PT and treatment group. The most common SAEs will also be presented by PT only.

AEs with outcome death will be presented separately by SOC and PT.

4.2.5.3 Adverse events leading to discontinuation or interruption of IP

The number and percent of patients will be presented by SOC and PT for AEs leading discontinuation of IP and AEs leading to temporary interruption (separately for the two

categories based action taken “Drug Permanently Discontinued” and “Drug Interrupted” respectively, recorded in the CRF AE module).

4.2.5.4 Amputations and preceding events

Amputations and preceding events (see section 3.3) will be presented in summary tables including the number and percent of patients with any event in the AE category, SAE, DAE and AE leading to interruption, and tabulated with frequency by SOC and PT.

In addition to presentations of the number of patients with event, the total number of events counting multiple events per subject will be presented.

In addition to the presentation of on-treatment events, on+off presentations will be provided amputations and preceding events.

4.2.5.5 Laboratory evaluation and vital signs

Summaries of creatinine and calculated eGFR will be based on creatine samples analyzed at the central laboratory.

The result and the change from baseline of creatinine, eGFR and vital signs, will be summarized by treatment group at each visit with scheduled measurement (see section 3.4) using descriptive statistics, including n, mean, SD, median and quartiles.

4.2.6 Analysis of exploratory objectives

Time to the first occurrence of hospitalisation from any cause will be analysed with the same method as the primary endpoint, based on information on the SAE eCRF form.

Change from baseline to each scheduled assessment visit (see section 3.4) for body weight, systolic blood pressure and eGFR will be analysed with a repeated measures model. All non-missing visit data will be used, including measurements after discontinuation of study drug. The model will include terms for treatment group, visit, visit*treatment group and the baseline measurement and T2D stratification factor as covariates. The model will be used to derive a least-squares estimate of the treatment difference with 95% confidence interval and corresponding two-sided p-value. Missing data will not be imputed.

For eGFR, the model above will additionally be used to derive the “total” slopes (between randomisation and eg 1 year and 2 years respectively) and the “chronic” slopes (between a post randomization time point to eg 1 year and 2 years respectively) will be estimated via linear contrasts.

The analysis of change in KCCQ clinical summary score, overall summary score, QoL score and sub-scores is described under ‘Supportive analyses and sensitivity analyses’ in section 4.2.4.2

EQ-5D-5L derived utility score will be summarised by descriptive statistics by visit and treatment group, and will be used to support modelling in a separate health economic report.

Patient global impression of severity (PGIS) will be tabulated by visit and treatment group, and will be used in anchor based analyses to support threshold for clinically important change of KCCQ total symptom score.

5 INTERIM ANALYSES

An interim analysis is planned to be performed including approximately 67% of the target number of patients with adjudicated primary endpoint events. There will in principle be one planned interim analysis for efficacy, with the possibility of the data monitoring committee (DMC) to conduct subsequent interim analysis if they deem necessary. The significance level for final analysis will be determined by the Haybittle-Peto function based on the actual number of interim analyses, using the East software (Copyright © Cytel Inc). The interim analysis will assess superiority of dapagliflozin to placebo. The interim analysis will have a nominal two-sided alpha level of 0.2%. At the interim analysis, the primary composite endpoint will be tested first at the specified alpha level. If superiority is achieved for the primary endpoint, then the superiority of dapagliflozin to placebo on CV deaths will be tested at a two-sided level of 0.2%. If CV death is significant, then an action is triggered whereby the DMC will evaluate the totality of the efficacy data and safety data, to determine if benefit is unequivocal and overwhelming such that the DMC recommends ending the study.

If the interim analysis leads to a decision to terminate the study early based on pre-defined stopping guidelines, the interim analysis database will become the basis of statistical inference for the primary endpoint and CV death. Following such a decision, the executive committee will define a PACD, on or after which study closure visits will commence. Analysis based on the final database will be conducted to support the full reporting of the study. The consistency between the interim analysis database and the subsequently locked database will be assessed.

If the study is stopped in the efficacy interim analysis, testing of secondary endpoints will be performed on the final database with the same testing procedure as described in section 4.1.3 with two-sided significance level 0.002.

A futility analysis is planned to be performed at the same time as the planned interim analysis. The study may be stopped for futility if the observed HR for the primary endpoint is > 0.946, corresponding to a predictive power of 5%. If the futility criterion of the primary endpoint is met, then DMC will evaluate the totality of data, including potential benefits on patient reported outcomes to consider recommending ending the study for futility.

6 CHANGES OF ANALYSIS FROM PROTOCOL

NA

7 REFERENCES

Fitchett et al 2016
Fitchett D, Zinman B, Wanner C, Lachin JM, Hantel S, Salsali A, et al. Heart failure outcomes with empagliflozin in patients with type 2 diabetes at high cardiovascular risk: results of the EMPA-REG OUTCOME® trial. Eur Heart J 2016;14;37(19):1526-34.

Ghosh and Lin 2000
Ghosh D, Lin DY. Nonparametric analysis of recurrent events and death. Biometrics 2000; 56:554–562.

Green et al 2000
C. Patrick Green, MD, Charles B. Porter, MD, FACC, Dennis R. Bresnahan, MD, FACC, John A.Spertus, MD, MPH, FACC Development and Evaluation of the Kansas City Cardiomyopathy Questionnaire: A New Health Status Measure for Heart Failure. JACC, Vol.35 No 5, April 2000:1245-55

Hicks et al 2018
Hicks KA, Mahaffey KW, Mehran R, Nissen SE, et al. Cardiovascular and Stroke Endpoint Definitions for Clinical Trials. J Am Coll Cardiol 2018;71:1021–34

Kawaguchi et al 2011
Kawaguchi A, Koch G, Wang X. Stratified Multivariate Mann-Whitney Estimators for the Comparison of Two Treatments with Randomization Based Covariance Adjustment. Statistics in Biopharmaceutical Research, 2011;3(2), 217–231.

Kawaguchi and Koch 2015
Kawaguchi A, Koch, GG. sanon: An R Package for Stratified Analysis with Nonparametric Covariable Adjustment. Journal of Statistical Software, 2015;67(9).

Kristensen et al 2015
Kristensen SL, Jhund PS, Køber L, McKelvie RS, Zile MR, Anand IS, et al. Relative importance of history of heart failure hospitalisation and N-terminal Pro–B-type natriuretic peptide level as predictors of outcomes in patients With heart failure and preserved ejection fraction. JACC Heart Fail 2015 Jun;3(6):478-86.

Kristensen et al 2017
Kristensen SL, Mogensen UM, Jhund PS, Petrie MC, Preiss D1, Win S, et al. Clinical and echocardiographic characteristics and cardiovascular outcomes according to diabetes status in patients with heart failure and preserved ejection fraction: a report from the I-Preserve Trial (Irbesartan in Heart Failure With Preserved Ejection Fraction). Circulation. 201721;135(8):724-35.

Levey at al 2009
Levey AS, Stevens LA, Schmid CH, Zhang YL, Castro AF 3rd, Feldman HI, Kusek JW, Eggers P, VanLente F, Greene T, Coresh J; CKD-EPI (Chronic Kidney Disease Epidemiology Collaboration). A new equation to estimate glomerular filtration rate. Ann Intern Med. 2009 May 5; 150(9):604-12

Lin et al 2000
Lin DY, Wei LJ, Yang I, Ying Z. Semiparametric regression for the mean and rate functions of recurrent events. Journal of the Royal Statistical Society: Series B (Statistical Methodology) 2000; 62(4):711–730.

Neal et al 2017
Neal B, Perkovic V, Mahaffey KW, de Zeeuw D, Fulcher G, Erondu N, et al, for the CANVAS Program Collaborative Group. Canagliflozin and cardiovascular and renal events in type 2 diabetes. N Engl J Med 2017; 377: 644-657.

Pfeffer et al 2015
Pfeffer MA, Claggett B, Assmann SF, Boineau R, Anand IS, Clausell N, et al. Regional Variation in Patients and Outcomes in the Treatment of Preserved Cardiac Function Heart Failure With an Aldosterone Antagonist (TOPCAT) Trial. Circulation. 2015;131:34-42.

Ratitch et al 2013
Ratitch B, Lipkovich I, O'Kelly M. Combining Analysis Results from Multiply Imputed Categorical Data. PharmaSUG 2013 - Paper SP03

Rogers et al 2016
Jennifer K Rogers, Alex Yaroshinsky, Stuart J Pocock, David Stokar and Janice Pogoda. Analysis of recurrent events with an associated informative dropout time: Application of the joint frailty model. Statist. Med. 2016, 35 2195–2205

Rådholm et al 2018
Rådholm K, Figtree, Perkovic V, Solomon SD, Mahaffey KW, et al. Canagliflozin and Heart Failure in Type 2 Diabetes Mellitus: Results From the CANVAS Program (Canagliflozin Cardiovascular Assessment Study). Circulation. 2018;137
https://doi.org/10.1161/CIRCULATIONAHA.118.034222

Spertus et al 2005
Spertus J, Peterson E, Conard MW, Heidenreich PA, Krumholz HM, Jones P, et al. Monitoring clinical changes in patients with heart failure: A comparison of methods. Am Heart J 2005;150:707-15.

Stokes et al 2012
Stokes ME, Davis CS, Koch GG.Categorical Data Analysis Using the SAS System. Carry, NC: SAS Institute Inc.

Wang and Pocock 2016
Wang D, Pocock S. A win ratio approach to comparing continuous non-normal outcomes in clinical trials. Pharmaceut. Statist. 2016, 15 238–245

**Statistical Analysis Plan**	
Study Code	D169CC00001
Edition Number	5.0
Date	8 December 2021

An International, Double-blind, Randomised, Placebo-Controlled Phase III Study to Evaluate the Effect of Dapagliflozin on Reducing CV Death or Worsening Heart Failure in Patients with Heart Failure with Preserved Ejection Fraction (HFpEF)

DELIVER - Dapagliflozin Evaluation to Improve the LIVEs of Patients with PReserved Ejection Fraction Heart Failure

An International, Double-blind, Randomised, Placebo-Controlled Phase III Study to Evaluate the Effect of Dapagliflozin on Reducing CV Death or Worsening Heart Failure in Patients with Heart Failure with Preserved Ejection Fraction (HFpEF)

DELIVER - Dapagliflozin Evaluation to improve the LIVEs of patients with pReserved ejection fraction heart failure

Study Statistician

Ulrica Wilderäng | Date

An International, Double-blind, Randomised, Placebo-Controlled Phase III Study to Evaluate the Effect of Dapagliflozin on Reducing CV Death or Worsening Heart Failure in Patients with Heart Failure with Preserved Ejection Fraction (HFpEF)

DELIVER - Dapagliflozin Evaluation to improve the LIVEs of patients with pReserved ejection fraction heart failure

Global Product Statistician

	Olof Bengtsson	Date

TABLE OF CONTENTS

TABLE OF CONTENTS 4

AMENDMENT HISTORY 8

1 STUDY DETAILS 16
1.1 Study Objectives 16
1.1.1 Primary Objective 16
1.1.2 Secondary Objectives 16
1.1.3 Safety Objectives 16
1.1.4 Exploratory Objectives 17
1.2 Definitions 17
1.2.1 Primary Analysis Censoring Date 17
1.2.2 Withdrawal of Consent 18
1.2.3 Discontinuation of Investigational Product 18
1.2.4 Vital Status 18
1.2.5 Lost to Follow-up 18
1.3 Study Design 18
1.3.1 Randomisation 19
1.4 Number of Subjects 20

2 ANALYSIS SETS 22
2.1 Definition of Analysis Sets 22
2.1.1 Full Analysis Set 22
2.1.2 Safety Analysis Set 22
2.2 Violations and Deviations 22

3 PRIMARY AND SECONDARY VARIABLES 23
3.1 Primary Variable 23
3.2 Secondary Variables 24
3.2.1 Total Number of Heart Failure Events (First and Recurrent) and Cardiovascular Death 24
3.2.2 Change from Baseline at 8 Months in the KCCQ Total Symptom Score 24
3.2.3 Cardiovascular Death 25
3.2.4 Death from Any Cause 25
3.3 Safety Variables 25
3.4 Laboratory Values and Vital Signs 26
3.4.1 Baseline Laboratory Values and Vital Signs 26

4 ANALYSIS METHODS 26
4.1 General Principles 26
4.1.1 Estimand for Primary and Secondary Outcomes 27
4.1.2 Hypotheses 27
4.1.3 Confirmatory Testing Procedure 28
4.1.4 Presentation of Time-to-Event Analyses 30
4.1.5 Vital Status and Follow-up of Endpoints 31

4.2 Analysis Methods 31
4.2.1 Demographics and Baseline Characteristics 31
4.2.2 Concomitant and Baseline Medication 32
4.2.3 Analysis of the Primary Efficacy Variables 32
4.2.4 Analysis of the Secondary Efficacy Variables 35
4.2.5 Analysis of Safety Variables 41
4.2.6 Analysis of Exploratory Objectives 43

5 INTERIM ANALYSES 44

6 CHANGES OF ANALYSIS FROM PROTOCOL 45

7 REFERENCES 46

Appendix A Estimation of Clinically Meaningful Thresholds for KCCQ Total Symptom Score 49

Appendix B Programming Code for Calculating Significance Level for LVEF <60% Subpopulation 57

LIST OF TABLES

Table 1 Level of α_1 Depending on Proportion of Events in LVEF < 60% Subpopulation 29

Table 2 Characteristics and Categories for Subgroup Analysis of the Primary Endpoint 33

Table A1 Categories of Change from Baseline PGIS in Heart Failure Symptoms at 8 Months 50

Table A2 Distribution of Change from Baseline KCCQ-TSS at 8 months by Change from Baseline PGIS 8 Months 52

Table A3 Distribution-Based Cut-offs for a Minimal Clinically Important Difference in the KCCQ-TSS 56

LIST OF FIGURES

Figure 1 Study Design 19

Figure A1 Empirical Cumulative Distribution Function for Change from Baseline KCCQ-TSS at 8 Months versus Change from Baseline PGIS at 8 Months with 7 Categories 53

Figure A2 Empirical Cumulative Distribution Function for Change from Baseline KCCQ-TSS at 8 Months Versus Change from Baseline PGIS at 8 Months with 5 Categories (Collapsing “Moderate” and “Large”) 54

Figure A3 Empirical Cumulative Distribution Function for Change from Baseline KCCQ-TSS at 8 months Versus Change from Baseline PGIS at 8 Months with 5 Categories (Collapsing “Small” and “Moderate”) 55

LIST OF ABBREVIATIONS

**Abbreviation or special term**	**Explanation**
AE	Adverse event
ATC	Anatomical therapeutic chemical
BP	Blood pressure
CDF	Cumulative distribution function
CEA	Clinical event adjudication
CKD-EPI	Chronic kidney disease epidemiology collaboration equation
CMH	Cochran-Mantel-Haenszel test
CMWPC	Clinically meaningful within-patient change
COVID-19	Corona Virus Disease 2019
CSP	Clinical study protocol
CV	Cardiovascular
DAE	Adverse events leading to discontinuation of investigational product
DBP	Diastolic blood pressure
DKA	Diabetic ketoacidosis
DMC	Data monitoring committee
eCDF	Empirical cumulative distribution function
eCRF	Electronic case report form
eGFR	Estimated glomerular filtration rate
FAS	Full analysis set
HbA1c	Haemoglobin A1c
HF	Heart failure
HFpEF	Heart failure with preserved ejection fraction
HR	Hazard ratio
IP	Investigational product (dapagliflozin or matching placebo)
ITT	Intention to treat
IxRS	Interactive Voice/Web Response System
KCCQ	Kansas City Cardiomyopathy Questionnaire
KM	Kaplan-Meier
LTFU	Lost to follow-up
LVEF	Left ventricular ejection fraction
MCID	Minimal clinically important difference
MedDRA	Medical dictionary for regulatory activities
MMRM	Mixed Model Repeated Measures

Abbreviation or special term	Explanation
MTP	Multiple testing procedure
NT-proBNP	N-terminal pro b-type natriuretic peptide
NYHA	New York Heart Association
PACD	Primary analysis censoring date
PGIS	Patient global impression of severity
PRAC	European Medicines Agency's Pharmacovigilance Risk Assessment Committee
PT	MedDRA preferred term
PTDV	Premature treatment discontinuation visit
QoL	Quality of life
SAE	Serious adverse event
SAS	Safety analysis set
SBP	Systolic blood pressure
SCV	Study closure visit
SD	Standard deviation
SEM	Standard error of measurement
SGLT2	Sodium-glucose co-transporter 2
SOC	MedDRA system organ class
T2D	Type 2 diabetes
TSS	Total symptom score
WHO	World Health Organization
WoC	Withdrawal of consent
WR	Win ratio

AMENDMENT HISTORY

Date	Brief description of change
Version 1<br>27 August 2018	Version 1.0 signed

Version 2<br>6 November 2020	[1.1 Study objectives]<br>Updated primary objective with dual primary analyses:<br>Primary analysis to be analysed in full study population and subpopulation with LVEF < 60%<br>Updated secondary objectives:<br>First secondary to be analysed in full study population and subpopulation with LVEF < 60%. Adding urgent HF visits to total number of HF events (first and recurrent) and CV death.<br>Moved NYHA class from secondary objective to exploratory.<br>Added CV death as secondary objective.<br>Updated exploratory objectives:<br>Added NYHA class objective from secondary objective and removed PGIS objective.<br>Rewording of EQ-5D-5L objective and endpoint.<br>[1.2.1 Primary analysis censoring date]<br>Increased target number of primary endpoint events from 844 to 1117.<br>[1.3 Study design]<br>Updated definition of subacute patients, increasing hospitalisation from within 21 days to within 30 days.<br>Increased number of randomised patients from 4700 to 6100 and number of enrolled patients from 8000 to 11000.<br>Updated target number of primary endpoint events from 844 to 1117.<br>Updated anticipated total study duration from 33 months to 39 months.<br>[1.4 Number of subjects]<br>Updated power, study duration, number of events and proportion of subacute.<br>[2.1.1 Full analysis set]<br>Updated with subpopulation information: "A subset of the full analysis set consisting of patients with baseline LVEF of < 60% (or LVEF < 60% subpopulation) will be analysed separately as part of the confirmatory statistical testing procedure."<br>[3.2 Secondary variables]<br>Updated with dual primary endpoints.<br>Updated with new definition of total number of events, including urgent HF visits.<br>Added Figure 2 with updated multiple testing procedure with dual primary analyses.<br>[3.2.1 Total number of (first and recurrent) hospitalisations for HF and CV death]<br>Updated with definition of total number of events.<br>Updated with information regarding prioritisation, which event to be counted in recurrent event analysis, if HF event and CV death occur at same day.<br>[3.2.2 Change from baseline at 8 months in the KCCQ total symptom score]<br>Added definition regarding ranking.<br>[Previous 3.2.3 Proportion of patients with worsened NYHA class at 8 months]<br>Removed entire paragraph.<br>[3.2.3 Cardiovascular death]<br>Added paragraph with secondary objective concerning CV death.<br>[3.3 Safety variables]

Date	Brief description of change
	Added adjudication of potential DKA events.<br>Added major hypoglycaemic events to list of safety variables.<br>[4.1.1 Estimand for primary and secondary outcomes]<br>Added estimand for KCCQ TSS.<br>[4.1.2 Hypotheses]<br>Added dual primary hypotheses.<br>[4.1.3 Confirmatory testing procedure]<br>Updated with handling of alpha for split primary analyses.<br>Added Figure 2.<br>[4.2.3.2 Sensitivity analysis of the primary endpoint]<br>Updated with information that sensitivity analyses related to impact of COVID-19 will be added at next SAP update prior to interim analysis.<br>[4.2.4.1 Analysis of recurrent HF events and CV death]<br>Updated definition of HF events, including urgent HF visits.<br>Added handling on priority of events occurring on the same day.<br>[4.2.4.2 Analysis of change from baseline to 8 months in the KCCQ total symptom score]<br>Added information on how to handle analysis under COVID-19 pandemic.<br>Added information on ranking.<br>Added information on handling of missing response for reasons other than death.<br>Estimation of treatment effect updated.<br>Added update on handling of ceiling and floor effects.<br>Information on imputation updated.<br>Updated information on TSS responder analyses.<br>[4.2.4.3 NYHA]<br>Section removed and moved to 4.2.6 Analysis of exploratory objectives.<br>[4.2.4.3 CV death]<br>Section on analysis of CV death added.<br>[4.2.5.4 Amputations and preceding events]<br>Section renamed to “Specific adverse events” and paragraphs on DKA, major hypoglycaemic events and genital infections added.<br>[4.2.6 Analysis of exploratory objectives]<br>Section on NYHA added (moved from previous Section 4.2.4.3).<br>Section on PGIS removed.<br>[5 Interim analysis]<br>Removed futility analysis.<br>[Reference]<br>Added references: FDA guidance during COVID-19 2020 and Spiessen and Debois 2010<br>Removed references: Kawaguchi and Koch 2015 and Neal et al 2017

Date	Brief description of change
Version 3.0<br>9 December 2020	[4.2.4.2 Analysis of change from baseline to 8 months in the KCCQ total symptom score]<br>Added information on responder analysis:<br>"Additional responder analysis will be performed in the same way as for 5 points improvement and deterioration described above, using the thresholds of clinically meaningful within-patient change from baseline TSS derived from anchor-based analyses of blinded study data as described in Appendix A, with "ceiling" and "floor" values handled consistently."<br><br>[Reference]<br>Added reference: Coon and Cook 2018.<br>[Appendix]<br>Added Appendix A describing how to estimate clinically meaningful thresholds for KCCQ total symptom score, using PGIS.

Version 4.0<br>20 May 2021	Minor edits done throughout entire document.<br>[1.2.1 Primary analysis censoring date]<br>Updated to be consistent with CSP, that SCV should be performed within 6 weeks after PACD, which can be extended if decided by Global Study Team.<br>Added that patients will stop taking IP at the SCV.<br>[1.4 Number of subjects]<br>Added information that final allocation of alpha and full testing procedure can be found in section 4.1.3. Added text that the power considerations stated in this section are examples for the dual primary analysis.<br>[3.2.1 Total number of HF events (first and recurrent) and CV death]<br>Removed: "Recurrent HF events (hospitalisation for HF or urgent HF visit), CV death and censoring processes all have continuous distributions so that HF events and death cannot happen at the same time."<br>Updated for clarification: "For patients who did not have a HF event or CV death, and following last event in patients with one or more HF events, censoring will follow the same rule as for the primary endpoint."<br>[3.2.3 Cardiovascular death]<br>Added "or died after WoC" for specification on patients to be censored.<br>[3.2.4 Death from any cause]<br>Added "or with unknown vital status" for specification on patients to be censored.<br>[3.3 Safety variables]<br>Updated list of safety variables, adding myocardial infarction, unstable angina, stroke, major hypoglycaemic events, potential diabetic ketoacidosis and amputations.<br>Updated for clarification: "These events will be recorded as **AEs or if they fulfil seriousness criteria as** SAEs in the database, but **SAEs** will not be reported to health authorities to avoid unnecessary unblinding."<br>[4.1 General principles]<br>Added for clarification: "**If the number of tablets dispensed or the number of tablets returned is missing for at least one observation, compliance is not calculated for that patient."** and<br>"IP compliance will be presented descriptively, including mean, **SD,** median, quartiles and 5% and 95% percentiles **for safety analysis set by treatment group**."<br>[4.1.1 Estimand for primary and secondary outcomes]<br>Sentence removed: "The time-to-first-event analysis by Cox proportional hazards regression and the analysis of recurrent events (Section 4.2.4) assume that missing data is at random."<br>[4.1.2 Hypotheses]<br>Removed reference to Haybittle-Peto function as that method will not to be used.<br>Updated alpha level for final analysis and added/removed details for clarification:<br>"**With alpha 0.2% allocated to** one planned interim analysis~~ including 67% of the target number of primary endpoints~~, the significance level **in the final analysis** will be **4.8%**, to be split between the dual hypothesis."<br>[4.1.3 Confirmatory testing procedure]<br>Section updated with details on significance levels.<br>Added table: "Table 1 Level of α_1 depending on proportion of events in LVEF < 60% subpopulation".<br>Updated for clarity: "If the study is stopped at the efficacy interim analysis (Section 5), testing of remaining secondary endpoints will be performed in the full study

	population only, in fixed sequence at two-sided alpha of 0.2% in the order described in the right branch of Figure 2."<br>[4.1.5 Vital status and follow-up of endpoints]<br>Added for clarification: "The denominator, representing maximum complete follow-up, will be the time **from randomisation until the earliest of** first primary endpoint event, death or PACD."<br>[4.2.2 Concomitant and baseline medication]<br>Added for clarification: "The **proportion** of **patients taking** baseline and concomitant medication will be presented for the FAS per ATC class and treatment group."<br>[4.2.3.1 Subgroup analysis of the primary endpoint]<br>Added for clarification: "A test of interaction between randomised treatment group and the subgroup variable will be performed using Cox proportional hazard model stratified **by** T2D **status at randomisation** with factors for treatment group, the subgroup variable and the interaction between treatment and subgroup."<br>Added: "Hazard ratio estimates, confidence intervals and p-values are not presented for subgroups with less than 15 events in total, both arms combined."<br>Table 1 renamed to Table 2<br>Table 2: Updated subgroups for LVEF at enrollment to ≤ 49%, 50% to 59%, ≥ 60%<br>[4.2.3.2 Sensitivity analysis of the primary endpoint]<br>Added information that further sensitivity analyses will be added at a later update: "**We will monitor the blinded study data to assess the impact of COVID-19 on the study and will add supportive and** sensitivity analyses related to the impact of COVID-19 on both primary and secondary endpoints in a SAP update prior to clinical data lock. **Also, additional covariates might be added to analyses, if deemed necessary based on blinded data.**"<br>[4.2.4.1 Analysis of total number of HF events (first and recurrent) and CV death]<br>Added for consistency: "The composite outcome of **total number of** HF events (first and recurrent) and CV death **with onset on or prior to PACD, adjudicated and confirmed by the CEA committee,**"<br>Sentence removed: "Recurrent HF events, CV death and censoring processes all have continuous distributions so that a HF event and death cannot happen at the same time."<br>[4.2.4.2 Analysis of change from baseline at 8 months in the KCCQ total symptom score]<br>Added for clarification: "In the ranking, patients who die prior to the first follow-up visit where KCCQ-KSS is assessed, at 1 month, will be defined as having a zero change from baseline while alive."<br>Added cut-off date to define population to be used in primary KCCQ-TSS analysis: "As a consequence, the main analysis of this endpoint will be done in the population with patients who had a planned visit 5 (8 months) prior to the major COVID-19 outbreak, **defined as 11th March 2020 (the date when WHO declared COVID-19 a pandemic)** thus unaffected by the pandemic's possible impact on health-related quality of life"<br>Removed: "The section regarding these analyses and exact date for data cut-off will be updated prior to the interim analysis."<br>Added that formal inference will be based on Win ratio method.<br>Section on responder analysis updated.<br>Section on handling of missing KCCQ data updated, including numbers from anchor-

Date	Brief description of change
	based analyses.<br>Clarifications made in section on "Handling of missing KCCQ data".<br>[4.2.4.3 Analysis of CV death]<br>Clarifications that CV deaths are confirmed in adjudication and how censoring is handled.<br>[4.2.4.4 Analysis of all-cause mortality]<br>Clarification that analysis includes deaths from any cause.<br>[4.2.5 Analysis of safety variables]<br>Updated that summaries of AEs will be presented both for the on-treatment period and on- and off-treatment period.<br>[4.2.5.1 Adverse events]<br>Updated list of safety variables, adding myocardial infarction, unstable angina, stroke, major hypoglycaemic events, potential DKA and amputations.<br>[4.2.5.4 Specific adverse events]<br>Added: "AEs leading to amputations" to list.<br>Added that event rate will be presented for AEs leading to amputations and preceding events, DKA and major hypoglycaemic events, as well as definition of event rate calculation.<br>Added for clarification: "**Events of genital area infections and necrotising fasciitis potential** of Fournier's gangrene".<br>[4.2.5.5 Laboratory evaluation and vital signs]<br>Removed PTDV and SCV from list of visits and added range to descriptive statistics.<br>[4.2.6 Analysis of exploratory objectives]<br>Added: "Only NYHA assessments made at site or through phone visits with the patients to be used in analyses."<br>Added clarification on exploratory KCCQ analyses.<br>[5 Interim Analyses]<br>Removed reference to Haybittle-Peto function as that method will not to be used.<br>[6 Changes of Analysis from Protocol]<br>Added: "**The alpha for final analysis adjusted for interim analysis at alpha 0.2% will be set to 5% minus 0.2% = 4.8%, rather than 4.98% as determined by the Haybittle-Peto function for 67% of events (sections 9.1 and 9.5 of the protocol).**"<br>[References]<br>Added reference: **Burman et al 2009.**<br>[Appendix A]<br>Earlier Appendix A renamed A1 Methods.<br>[Appendix A2]<br>Added appendix including summary of results of anchor-based analysis on blinded study data.<br>[Appendix B]<br>Added appendix with R code for calculation of significance level.

Date	Brief description of change
Version 5.0<br>08 December 2021	Formatting updated throughout entire document.<br>[3.3 Safety Variables]<br>Minor clarifications added<br>[4.1.3 Confirmatory Testing Procedure]<br>Sentence added:<br>"**For the calculation of α_1, the correlation will be based on the square root of the lower bound of a two-sided 95% confidence interval for the proportion of events in the subpopulation with LVEF◦<◦60%, using a normal approximation confidence interval for the proportion.**"<br>Table 1 updated presenting number of events in LVEF < 60% subpopulation and full population instead of presenting proportion of events in the subpopulation. Confidence intervals added and numbers for α_1 in the different scenarios updated.<br>Last bullet in the list clarified.<br>[4.1.5 Vital Status and Follow-up of Endpoints]<br>Clarified that non-CV death includes undetermined.<br>[4.2.3.1 Subgroup Analysis of the Primary Endpoint]<br>Updated that subgroup analysis will be done both for full population and LVEF < 60% subpopulation.<br>[4.2.3.2 Sensitivity Analysis of the Primary Endpoint]<br>Added description of a sensitivity analysis where patients with premature censoring have imputed time to event information and more detailed information about the planned tipping point analysis.<br>Added sensitivity analysis where patients and events are censored at the onset date of AE associated with COVID-19 infection.<br>[4.2.4.1 Analysis of Total Number of HF Events (First and Recurrent) and CV Death]<br>Added sensitivity analysis where patients and events are censored at the onset date of AE associated with COVID-19 infection.<br>[4.2.4.2 Analysis of Change from Baseline at 8 Months in the KCCQ Total Symptom Score]<br>Added that both **planned and** performed 8 month assessments are to be included in COVID-19 supplementary analysis for KCCQ TSS.<br>[4.2.4.3 Analysis of CV Death]<br>Added sensitivity analysis where patients and events are censored at the onset date of AE associated with COVID-19 infection.<br>[4.2.5.1 Adverse Events]<br>Clarification that on-treatment period will be used for primary analysis of all safety variables, except for amputations and preceding events.<br>Added that MedDRA 24.1 will be used.<br>Information previously in section "4.2.5.4 Specific adverse events" added to this section.<br>[4.2.5.4 Specific Adverse Events]<br>Text moved to be included in Section 4.2.5.1 and section removed.<br>[4.2.6 Analysis of Explorative Objectives]<br>Updated that KCCQ QoL will be reported descriptively only.<br>[Appendix B]<br>Updated to include R and SAS code.

1 STUDY DETAILS

1.1 Study Objectives

1.1.1 Primary Objective

Primary objective	Endpoint/variable
To determine whether dapagliflozin is superior to placebo, when added to standard of care, in reducing the composite of CV death and HF events (hospitalisation for HF or urgent HF visit) in patients with HF and preserved systolic function, in<br>• full study population<br>• subpopulation with LVEF < 60%	Time to the first occurrence of any of the components of this composite:<br>1. CV death<br>2. Hospitalisation for HF<br>3. Urgent HF visit (eg, emergency department or outpatient visit)

1.1.2 Secondary Objectives

Secondary objective	Endpoint/variable
To determine whether dapagliflozin is superior to placebo in reducing the total number of recurrent HF events (hospitalisations for HF or urgent HF visit) and CV death, in<br>• full study population<br>• subpopulation with LVEF < 60%	Total number of HF events (first and recurrent) and CV death
To determine whether dapagliflozin is superior to placebo in improving Patient Reported Outcomes measured by KCCQ	Change from baseline in the TSS of the KCCQ at 8 months
To determine whether dapagliflozin is superior to placebo in reducing CV death	Time to the occurrence of CV death
To determine whether dapagliflozin is superior to placebo in reducing all-cause mortality	Time to the occurrence of death from any cause

1.1.3 Safety Objectives

Safety Objective	Outcome Measure
To evaluate the safety and tolerability of dapagliflozin compared to placebo in patients with HFpEF	• SAEs<br>• DAEs<br>• Amputations, AEs leading to amputation and potential risk factor AEs for amputations affecting lower limbs

1.1.4 Exploratory Objectives

Exploratory Objective	Endpoint/Variable
To determine whether dapagliflozin is superior to placebo in reducing all-cause hospitalisation	Time to the first occurrence of hospitalisation from any cause
To determine whether dapagliflozin is superior to placebo in reducing the proportion of patients with worsened NYHA class	Proportion of patients with worsened NYHA class from baseline to 8 months
To describe health status assessed by EQ-5D-5L to support health economic analysis and health technology assessment	Results will be reported separately in a health economic report
To determine whether dapagliflozin compared with placebo will have an effect on SBP	Change in SBP from baseline
To determine whether dapagliflozin compared with placebo will have an effect on body weight	Change in body weight from baseline
To determine whether dapagliflozin compared with placebo will have an effect on eGFR	Change in eGFR from baseline
To explore whether dapagliflozin compared to placebo improves KCCQ summary scores, sub-scores of TSS (symptom frequency and symptom burden) and domains	Change in Clinical summary score, TSS sub-scores, Overall summary score, QoL score
To collect and store blood samples for future exploratory genetic research	Not applicable. Results will be reported separately

1.2 Definitions

1.2.1 Primary Analysis Censoring Date

The executive committee and AstraZeneca will monitor the accrual of endpoint events and when appropriate define the PACD at which time at least the pre-defined target number of 1117 events for the primary composite endpoint is expected to have occurred. The study sites will be instructed to plan for SCV to be performed within 6 weeks after PACD, which can be extended if decided by the Global Study Team. Patients will stop taking IP at the SCV.

Analyses of efficacy endpoint events will include events with onset on or prior to PACD. Event free patients who have not been prematurely censored due to incomplete information (see Section 3.1) will be censored at PACD. HF events and deaths with onset after PACD will also be adjudicated.

1.2.2 Withdrawal of Consent

Withdrawal of consent should only occur if the patient has received appropriate information about options for modified study follow-up and does not agree to any kind of further assessment or follow-up. Information regarding vital status (dead or alive) at the end of the study will be collected from public sources, to be included in the analysis of death from any cause as a sole outcome and in patient disposition summaries.

1.2.3 Discontinuation of Investigational Product

Discontinuation of IP does not mean discontinuation from study follow-up or WoC. Patients who discontinue from IP should continue study visits according to plan until study closure. If the patient does not agree to this approach, modified follow-up capturing the essential information for the objectives of the study should be arranged. Data will be included in the ITT analyses irrespective of whether the event occurred before or following discontinuation of IP.

1.2.4 Vital Status

Known vital status at the end of the study will be defined when the patient is dead or has date last know alive on or after the PACD.

For patients who have withdrawn consent, the investigator will attempt to collect vital status from publicly available sources at study closure in compliance with local privacy laws/practices.

1.2.5 Lost to Follow-up

The term LTFU will be limited to patients with unknown vital status at the end of the study as defined in Section 1.2.4. Other measures will be used to describe completeness of follow-up of the primary endpoint (Section 4.1.5).

1.3 Study Design

This is an international, multicentre, parallel-group, event-driven, randomised, double-blind, placebo-controlled study in patients with HFpEF, evaluating the effect of dapagliflozin 10 mg versus placebo, given once daily in addition to background regional standard of care therapy, including treatments to control co-morbidities, in reducing the composite of CV death or HF events.

HFpEF is defined for the purposes of this study as LVEF > 40% and evidence of structural heart disease. Adult patients with HFpEF, aged ≥ 40 years and with NYHA class II to IV will be randomised in a 1:1 ratio to receive either dapagliflozin 10 mg or placebo once daily. A proportion of patients, here denoted as the subacute group, will be randomised during hospitalisation for HF or within 30 days of discharge from hospitalisation for HF.

Originally, 4700 patients were planned to be randomised with a study duration of approximately 33 months, when 844 primary events had occurred. Based on the ongoing blinded monitoring of event accrual (including the percentage of patients from the subacute category), the sample size was increased from original 4700 to approximately 6100 patients.

It was estimated that approximately 11000 patients at approximately 400 to 500 sites in 20 to 25 countries will be enrolled to reach the target of approximately 6100 randomised patients.

In this event driven trial, study closure procedures will be initiated when the predetermined number of primary endpoints are predicted to have occurred (n = 1117), ie, the PACD (Section 1.2.1 and Figure Figure 1 Study Design). Patients should be scheduled for a SCV within 6 weeks of the PACD, which can be extended if decided by Global Study Team. The maximum treatment duration is expected to be approximately 39 months, dependent on randomisation rate and event rate. The number of patients randomised, the study duration, or both, may be changed if the randomisation rate or the event rate is different than anticipated.

Figure 1 Study Design

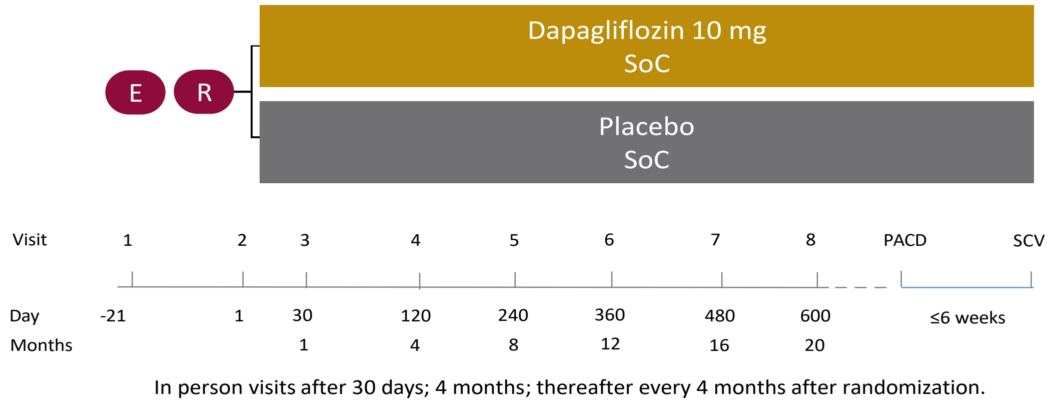

E=Enrolment; R=Randomization; SoC= Standard of Care; PACD=Primary Analysis Censoring Date; SCV=Study Closure Visit; FU=Follow Up

1.3.1 Randomisation

Patients will be randomised 1:1 to either dapagliflozin 10 mg or placebo once daily. The treatment allocation in this study will be double-blind. Randomisation will be stratified by T2D status at randomisation (2 levels: with T2D; without T2D). For the purpose of stratification, T2D is defined as established diagnosis of T2D or HbA1c ≥ 6.5% (48 mmol/mol) at enrolment (Visit 1; single measure) central laboratory test.

Randomisation will be performed in balanced blocks of fixed size. The randomisation codes will be computer generated and loaded into the IxRS database.

The number of randomised patients with T2D will be monitored in order to ensure a minimum of 30% patients in each group of patients with and without T2D. Randomisation may be capped (ie, no more patients can be randomised in a specific sub-population) if the pre-determined limit is reached.

Randomisation of patients based on geographic region will be monitored to ensure global representation. LVEF value, NYHA class, subacute/non-subacute group, and atrial fibrillation status at Visit 1 may be capped in IxRS to avoid over- or under-representation of these patient subgroups.

1.4 Number of Subjects

The primary objective of the study is to determine the superiority of dapagliflozin versus placebo added to standard of care in reducing the composite of CV death and HF events (hospitalisation for HF or urgent HF visit). Two hypotheses will be tested simultaneously (ie, dual primary analyses) for this primary objective: (1) in the full population and (2) in an LVEF < 60% subpopulation, with alpha allocated to each test.

Originally, assuming a true HR of 0.80 between dapagliflozin and placebo, using a two-sided alpha of 5%, 844 primary endpoint events were targeted in order to provide a statistical power of 90% for the test of the primary composite endpoint.

To allow testing for the dual primary analyses, alpha will be allocated to each test to ensure strong control of the overall type I error rate. The target number of patients with a primary endpoint has been increased to 1117 in order to provide adequate statistical power for each test. The power to reject the dual primary hypotheses depends on how alpha is allocated between the two hypotheses and the proportion of primary events in the LVEF < 60% subpopulation. It is anticipated that at least 70% of the primary endpoint events (ie, approximately 780 events) will be available for the LVEF < 60% subpopulation. The final allocation of alpha and full testing procedure is specified in Section 4.1.3 and the alpha levels used in the following text are just examples used to illustrate the power considerations for the dual primary analysis. For illustration, testing the effect on the primary endpoint in the LVEF < 60% subpopulation, a true HR of 0.80 and approximately 1117 primary endpoint events in the full population (at least 780 events in the subpopulation) would then provide at least:

- 80% power for a two-sided nominal alpha of 2.4%
- 85% power for a two-sided nominal alpha of 3.7%

For testing the effect on the primary endpoint in the full study population, a true HR of 0.80 and approximately 1117 primary endpoint events would also provide:

- 90% power for a two-sided nominal alpha of 1.5%

- 93% power for a two-sided nominal alpha of 2.4%.

This is based on an overall 1:1 allocation between dapagliflozin and placebo.

The HR 0.80 was originally chosen as a conservative assumption based on the observed HR 0.72 (95% confidence interval 0.50-1.04) for the composite of HF hospitalisation and CV death in patients with HF at baseline in the EMPA-REG OUTCOME trial (Fitchett et al 2016) and HR 0.61 (0.46-0.80) for patients with history of HF in the CANVAS program (Rådholm et al 2018) considering that these were post-hoc analyses in subgroups with limited documentation of baseline HF diagnosis, not characterised by ejection fraction.

The event rate assumptions are based on sub analyses of the TOPCAT and I-PRESERVE studies by geographic region, NT-proBNP levels, prior hospitalisation for HF, and T2D status (Pfeffer et al 2015, Kristensen et al 2015, Kristensen et al 2017). The sample size calculation builds on the assumption of an annual event rate of 9% in the placebo group for the majority of prevalent HFpEF patients, importantly all with NT-proBNP ≥ 300 pg/ml by inclusion criterion. Additionally, a subgroup of patients due to be discharged or recently discharged from a HF hospitalisation (here denoted 'subacute' patients) with a higher event rate is planned to be included. Assuming 20% of patients from the subacute category with an annual event rate of 24% during the first year and 9% thereafter for the remainder of the study, the original sample size of 4700 was estimated to provide 844 events during a recruitment period of 18 months and a minimum follow-up of 15 months.

Based on the ongoing blinded monitoring of event accrual (including the percentage of patients from the subacute category), the sample size was increased from original 4700 to approximately 6100 randomised patients. Accordingly, the recruitment period was anticipated to increase from the original 18 months to 26 months. Recruitment might be marginally prolonged in a few countries to meet local targets. The study is event driven and the number of patients or duration may further change.

With the same event rate assumptions as above, assuming 11% of patients from the subacute category, approximately 6100 patients were estimated to provide the required number of 1117 patients with a primary event in the full study population, during an anticipated recruitment period of 26 months and a minimum follow-up period of 13.5 months (total study duration 39 months).

In addition, the expected number of patients who will be LTFU is expected to be small; hence, these are not considered in the determination of the sample size.

2 ANALYSIS SETS

2.1 Definition of Analysis Sets

2.1.1 Full Analysis Set

All patients who have been randomised to IP will be included in the FAS irrespective of their protocol adherence and continued participation in the study. Patients will be analysed according to their randomised IP assignment, irrespective of the treatment actually received. The FAS will be considered the primary analysis set for the ITT analysis of primary and secondary variables and for the exploratory efficacy variables. A subset of the FAS consisting of patients with baseline LVEF of < 60% (or LVEF < 60% subpopulation) will be analysed separately as part of the confirmatory statistical testing procedure (see CSP Section 4.2 for justification of testing LVEF < 60% subpopulation).

2.1.2 Safety Analysis Set

All randomised patients who received at least 1 dose of randomised treatment will be included in the SAS. Patients will be analysed according to the treatment actually received. For any patients given incorrect treatment, ie, randomised to one of the treatment groups, but actually given the other treatment, the treatment group will be allocated as follows: Patients who got both incorrect and correct treatment will be analysed according to their randomised treatment. Patients who got only the incorrect treatment will be analysed according to that treatment.

The SAS will be considered the primary analysis set for all safety variables.

2.2 Violations and Deviations

The important protocol deviations listed below will be summarised by randomised treatment group

- Patients who were randomised but did not meet inclusion criteria, or met exclusion criteria
- Patients who received the wrong IP at any time during the study.
- Patients who received prohibited concomitant medication, which for this study is limited to open label SGLT2 inhibitors taken in combination with IP.

As the primary analysis is ITT analysis, protocol deviation will not imply exclusion from the primary analysis.

3 PRIMARY AND SECONDARY VARIABLES

Deaths and potential HF events will be adjudicated by an independent CEA committee. The CEA committee members will not have access to the treatment codes for any patient. The CEA procedures and event definitions will be described in the CEA charter according to the CDISC definitions (Hicks et al 2018).

Only HF hospitalisations and urgent HF visits confirmed by the CEA will be used in the analysis of the primary and secondary endpoints and their components.

The primary analyses of the endpoints concerning CV deaths, either as a component of a composite or on its own, will include deaths adjudicated as CV cause. Deaths adjudicated as "cause undetermined" will be considered as non-CV deaths in these analyses.

Adjudicated events occurring from randomisation until WoC or PACD will be included in the analysis of primary and secondary endpoints. The analysis of all-cause death as a sole outcome will in addition include any deaths (not adjudicated) after WoC, but on or before PACD.

3.1 Primary Variable

The primary efficacy variable is time from randomisation to the first occurrence of any event in the composite of CV death, hospitalisation for HF or an urgent HF visit.

Patients who did not have an adjudicated primary endpoint event on or prior to PACD will be censored at the earliest of date of WoC or non-CV death when applicable, and otherwise at the date of the last clinical event assessment or the PACD, whichever occurs first. It is expected that patients alive and under study follow-up will have a clinical event assessment at their SCV after PACD. Last clinical event assessment is defined as the last date when the event assessment question for a potential HF event was completed on the eCRF event assessment page.

In analysis of the individual components hospitalisation for HF and urgent HF visit, to examine their contribution to the composite endpoint, date of death from any cause will be an additional point of censoring.

For analysis of time to first event, data will be expressed as two variables:

- A binary variable indicating whether the event in question occurred, or the patient was censored.
- An integer variable for the number of days from randomisation to the first occurrence of an event (start date of the event – randomisation date + 1), or for event free patients, from randomisation to censoring (censoring date – randomisation date + 1).

3.2 Secondary Variables

The secondary endpoints are included in hierarchical testing sequences following the dual primary analysis as described in Section 4.1.3 and depicted in Figure 2.

3.2.1 Total Number of Heart Failure Events (First and Recurrent) and Cardiovascular Death

The efficacy variable is the total number of first and recurrent HF events (hospitalisations for HF or urgent HF visits) and CV death.

For the analysis of first and recurrent HF events and CV death, the data will be expressed in counting process style for input to the analysis as described in Section 4.2.4.1, as follows. The time from randomisation to end of follow-up/censoring will be split into one or more interval with variables for start of interval, end of interval and a variable indicating if an event occurred at the end of each respective interval, or if the patient was censored. If a HF event and CV death occurred at the same day, then only the CV death will be counted.

For patients who did not have a HF event or CV death, and following last event in patients with one or more HF events, censoring will follow the same rule as for the primary endpoint.

3.2.2 Change from Baseline at 8 Months in the KCCQ Total Symptom Score

The efficacy variable is the change from baseline at 8 months of the KCCQ-TSS.

The KCCQ is a self-administered disease specific instrument for patients with HF (Green et al 2000, Spertus et al 2005). The KCCQ consists of 23 items measuring HF-related symptoms, physical limitations, social limitations, self-efficacy, and health-related quality of life. The TSS incorporates the symptom burden and symptom frequency domains into a single score. Scores are transformed to a range of 0 to 100. Higher scores represent better outcomes.

Baseline is defined as the value at randomisation visit (Visit 2). Change from baseline at each post-baseline analysis time point will be calculated as the value at the corresponding post-baseline analysis time point minus the baseline value. The KCCQ is assessed by the patient at randomisation, at the visits targeted 1, 4 and 8 months following randomisation and at PTDV and SCV. By the ITT principle, the analysis will include all data irrespective of whether the patient has discontinued IP.

In order to account for patients who die prior to the 8-month assessment and to accommodate non-normal distribution of KCCQ scores, a composite rank-based endpoint will be used. The values of change from baseline at 8 months in TSS of patients who survive to 8 months will be converted to ranks (across both treatment groups combined) with lower ranks attributed to worse outcomes (ie, lower ranks corresponding to negative or smaller values of change from

baseline). Patients who die prior to the 8-month assessment will be assigned the worst rank, ie, worse than any patient surviving to 8 months, but among the deceased the relative ranking will be based on their last value of change from baseline in TSS while alive.

3.2.3 Cardiovascular Death

The efficacy variable is time from randomisation to CV death, confirmed in adjudication. All CV deaths on or prior to PACD will be included. Patients who are alive or died after WoC will be censored at the earliest of date of WoC, last known alive and PACD. Patients who die of any other cause are censored at their date of death.

3.2.4 Death from Any Cause

The efficacy variable is time from randomisation to death from any cause. All deaths on or prior to PACD, including any deaths after WoC, will be included. Patients who are alive or with unknown vital status will be censored at the earliest of date last known alive and PACD.

3.3 Safety Variables

The safety and tolerability of dapagliflozin in patients with HFpEF will be evaluated from SAEs, DAEs, amputations, AEs leading to amputation and AEs reflecting potential risk factors for lower limb amputations (“preceding events”).

In addition to amputation, non-serious and serious events potentially placing the patient at risk for a lower limb amputation, in this document denoted “preceding events”, should also be recorded in the eCRF as AE/SAE, whether or not an amputation has taken place. Preceding events will be defined for analysis by a predefined list of PRAC PTs. Additional information about amputations with underlying conditions and preceding events will be collected on dedicated eCRF pages.

SAEs will be collected from time of informed consent until and including the patient’s last visit. Non-serious AEs will be collected from randomisation until and including the patient’s last visit. Collection of non-serious AEs includes cardiac ischaemic events (myocardial infarction and unstable angina), stroke, major hypoglycaemic events, potential DKA, amputations, AE leading to amputation, and preceding events, AEs leading to a potential endpoint, DAEs and AEs which are the reason for interruption of IP.

Efficacy endpoints (deaths and potential HF events) will be adjudicated. These events will be recorded as AEs or, if they fulfil seriousness criteria, as SAEs in the database, but SAEs will not be reported to health authorities to avoid unnecessary unblinding. However, if it is determined by the CEA committee that a potential endpoint does not meet the endpoint criteria, the event will be reported to AstraZeneca patient safety data entry site and if applicable to the health authorities.

For SAEs or DAEs reported by the Investigator as potential DKA, additional information will be recorded on specific eCRF pages in addition to the AE/SAE form. All potential DKA events will be adjudicated by an independent committee and adjudicated outcomes will be considered the main analysis for DKA events.

For myocardial infarctions, unstable angina, stroke, major hypoglycaemic events and amputations, additional information will be recorded on specific eCRF pages in addition to the AE/SAE form.

3.4 Laboratory Values and Vital Signs

Blood samples will be taken for central laboratory assessment of creatinine and calculation of eGFR at enrolment visit, at the visits targeted 1, 4, and 12 months following randomisation, then annually and at PTDV and SCV. eGFR will be calculated (in mL/min/1.73 m^2) using the CKD-EPI formula (Levey at al 2009).

Central laboratory assessment of NT-proBNP and HbA1c will be taken at Visit 1.

Systolic blood pressure, DBP, and pulse rate will be measured at Visit 1, Visit 2, at 1 and 12 months visit, then annually and at PTDV and SCV.

Weight will be measured at Visit 1, at the 12 months visit, then annually and at PTDV and SCV.

3.4.1 Baseline Laboratory Values and Vital Signs

In principle, baseline will be defined as the last value on or prior to date of first dose of randomised IP, or for patients who did not receive treatment, the last value on or prior to date of randomisation. Except for cases of rescreening this will be Visit 1 measurement of weight, NT-proBNP, eGFR and HbA1c, and Visit 2 measurement of SBP, DBP, and pulse rate.

4 ANALYSIS METHODS

4.1 General Principles

No multiplicity adjustment will be made to confidence intervals as they will be interpreted descriptively and used as a measure of precision. All p-values will be unadjusted. P-values for variables not included in the confirmatory testing sequence, or following a non-significant test in the sequence, will be regarded as nominal.

Primary and secondary analyses of HF events and death include adjudicated events occurring on or prior to PACD.

Stratification of analyses for T2D status will be performed using the stratification values as entered in IxRS to determine the randomisation assignment.

<u>Incomplete dates</u>

If only the year part of a date is available (YY), then the date will be set to YY0701. If only the year and month is available (YYMM), then the date will be set to YYMM15. Additional imputation rules will be defined as appropriate to ensure that eg, dates will not be imputed as prior to randomisation, after death or start date after end date.

<u>IP compliance</u>

The percentage of IP compliance for the overall treatment period will be derived for each patient based on pill counts as the number of pills taken (dispensed – returned), relative to the expected number of pills taken. The expected number of pills taken is defined as 1 × (date of last dose – date of first dose + 1), excluding days of interruption. If the number of tablets dispensed or the number of tablets returned is missing for at least 1 observation, compliance is not calculated for that patient.

IP compliance will be presented descriptively, including mean, SD, median, quartiles and 5% and 95% percentiles for SAS by treatment group.

4.1.1 Estimand for Primary and Secondary Outcomes

The primary and secondary event-based objectives will be evaluated under the treatment policy estimand including differences in outcomes over the entire study period until PACD to reflect the effect of the initially assigned randomised IP, irrespective of exposure to IP, concomitant treatment as well as subsequent treatment after discontinuation of IP. The analysis will be performed for the FAS including all events that occurred on or prior to PACD, including events following premature discontinuation of IP.

The estimand for the change from baseline in KCCQ-TSS at 8 months will employ a combination of a treatment policy strategy and a composite strategy. For the intercurrent event of death (due to any cause) prior to the KCCQ assessment at 8 months, a composite strategy will be used, where death will be considered unfavorable and represented by a lowest (worst) rank of a combined outcome variable as described in Section 3.2.2. For all other types of intercurrent events, including but not limited to a premature discontinuation of randomised treatment, a treatment policy strategy will be used.

4.1.2 Hypotheses

The primary endpoint will be tested twice, simultaneously: (1) in the full study population, and (2) in the LVEF < 60% subpopulation.

To control the overall type I error rate at 5% two-sided, the significance level will be adjusted for interim analysis of efficacy performed by the DMC (Section 5). With alpha 0.2% allocated to one planned interim analysis, the significance level in the final analysis will be 4.8%, to be

split between the dual hypotheses. The following null hypothesis will be tested for both the dual analyses of the primary endpoint

H0: HR [dapagliflozin:placebo] = 1

versus the alternative hypothesis

H1: HR [dapagliflozin:placebo] ≠ 1

The secondary endpoints included in confirmatory statistical testing using a closed testing procedure (Section 4.1.3) will be based on similar two-sided alternative hypotheses for the respective treatment difference.

4.1.3 Confirmatory Testing Procedure

A closed testing procedure including a pre-specified hierarchical ordering of the primary and secondary endpoints will be utilised, with recycling of alpha following the framework of Burman et al 2009. The Type I error will be controlled at an overall two-sided 5% level across primary and secondary endpoints and in consideration of the planned interim analysis. Two-sided nominal p-values will be reported for each hypothesis. Statistical significance for a given hypothesis will be declared if the point estimate is in favour of the dapagliflozin arm, in addition to the two-sided p-value meeting the corresponding p-value threshold.

At the final analysis, statistical significance will be assessed in two branches in the pre-specified order of the endpoints and populations as specified in Figure 2. The total significance level, alpha, will be split for the two primary analyses of the primary endpoint, allocating α_1 to test the subpopulation and α_2 to test the full population.

For derivation of the two-sided nominal p-value thresholds α_1 and α_2, in the first step of the MTP, a two-sided alpha of 0.2% will be allocated to the interim analysis and 4.8% to the final analysis. The significance level α_2 (for the primary analysis in the full population at the final analysis) will be fixed at 2.4% two-sided. The inherent correlation structure between the full population and the LVEF < 60% subpopulation, where the corresponding test statistics for the primary endpoint are bivariate normal with correlation equal to the proportion of events in the LVEF < 60% subpopulation, will be taken into account when calculating α_1 (Spiessen and Debois 2010). For the calculation of α_1, the correlation will be based on the square root of the lower bound of a two-sided 95% confidence interval for the proportion of events in the subpopulation with LVEF < 60%, using a normal approximation confidence interval for the proportion. The threshold α_1 will be such that for $\alpha_2 = 2.4\%$ two-sided; the two-sided probability of rejecting at least one true null hypothesis at the final analysis will be 4.8%. It then follows that if the primary endpoint in the full population at interim analysis is assessed versus a two-sided p-value of 0.2%, the two-sided probability of rejecting at least one true primary null hypothesis at any analysis can be no larger than 5%. Table 2 shows how the two-

sided nominal p-value threshold α_1 depends on the proportion of events in the LVEF < 60% subpopulation at the final analysis. R and SAS code for calculating α_1 is provided in Appendix B.

Table 2 Level of α_1 Depending on Proportion of Events in LVEF < 60% Subpopulation

Patients with event (LVEF < 60% / overall)	Proportion (95% CI)	Correlation = sqrt of lower confidence limit	Two-sided alpha (%) for primary endpoint		
			Interim analysis<br>Full population	Final analysis (α_2)<br>Full population	Final analysis (α_1)<br>Subpopulation LVEF < 60%
780/1117	0.698 (0.671, 0.725)	0.819	0.2	2.4	3.647
790/1117	0.707 (0.681, 0.734)	0.825	0.2	2.4	3.674
800/1117	0.716 (0.690, 0.743)	0.831	0.2	2.4	3.701
810/1117	0.725 (0.699, 0.751)	0.836	0.2	2.4	3.730
820/1117	0.734 (0.708, 0.760)	0.842	0.2	2.4	3.758
830/1117	0.743 (0.717, 0.769)	0.847	0.2	2.4	3.788

CI, Confidence interval; LVEF, left ventricular ejection fraction; sqrt square root.

- If both the primary null hypotheses can be rejected, the following hypotheses in each branch will be tested at 2.4%, in the order described in Figure 2.
- The following will apply if only one of the tests of the primary endpoint can be rejected at respective levels 2.4% (in the full population) and α_1 (in the LVEF < 60% subpopulation): the remaining hypotheses in the branch where the primary hypothesis was rejected will be tested in fixed sequence at the following two-sided significance levels
  - 4.8% – 2.4% = 2.4% in the left branch only (in case the primary endpoint in the subpopulation was significant at level α_1 but not in the full population at level 2.4%)
  - 4.8% – α_1 in the right branch only (in case the primary endpoint in the full population was significant at level 2.4% but not in the subpopulation at level α_1)
- If all hypotheses in one branch are rejected, alpha will be recycled to the other branch, where remaining unrejected hypotheses can be tested at full alpha adjusted for interim analysis (ie, 4.8%) in the order described in Figure 2.
- If the first secondary hypothesis (recurrent HF events and CV death) in full study population is rejected in one of the branches, it does not have to be re-tested in the other branch. If the primary hypothesis is rejected in both branches and the first secondary

hypothesis (recurrent events) is rejected in the LVEF < 60% subpopulation, then the first secondary hypothesis in full population can be tested at full alpha adjusted for interim analysis (4.8%).

If the study is stopped at the efficacy interim analysis (Section 5), testing of remaining secondary endpoints will be performed in the full study population only, in fixed sequence at two-sided alpha of 0.2% in the order described in the right branch of Figure 2.

Figure 2 Testing Procedure

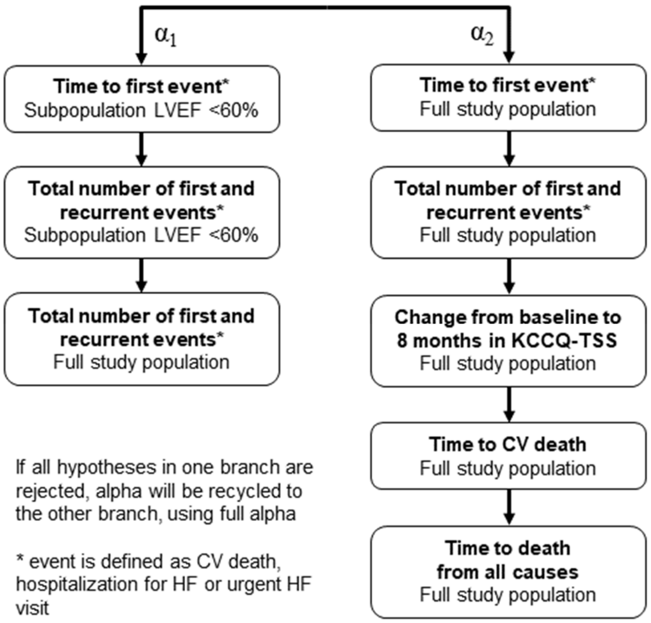

CV, cardiovascular; HF, heart failure; KCCQ, Kansas city cardiomyopathy questionnaire; LVEF, left ventricular ejection fraction; TSS, total symptom score

4.1.4 Presentation of Time-to-Event Analyses

In general, summary tables of time-to-event analyses will include the number and percent of patients with event per treatment group, event rate, HR with 95% confidence interval and p-value. The event rate will be derived as the number of patients with event divided by the total duration of follow-up across all patients in the given group.

Kaplan-Meier estimates of the cumulative proportion of patients with events will be calculated and plotted per treatment group, with the number of patients at risk indicated below the plot at specific time points. The KM plots will be presented for all time to event analyses, including the individual components of the composite endpoints.

4.1.5 Vital Status and Follow-up of Endpoints

Potential HF endpoints and deaths will be collected and adjudicated from randomisation throughout the study until and including the patient's last visit. The investigator will attempt to collect vital status (dead or alive) at the end of the study for all patients, including vital status from publicly available sources for patients who have withdrawn consent, in compliance with local privacy laws/practices.

Known vital status at the end of the study will be defined when the patient is dead or has date last know alive on or after the PACD. In patient disposition the number of patients who are dead, alive or with unknown vital status will be reported separately for patients who did/did not withdraw consent. The term LTFU will be limited to only patients with unknown vital status.

Follow-up of the primary endpoint will be defined in terms of completion of the event assessment question for a potential HF event as described for censoring in Section 3.1. Thus, a patient that is not LTFU, ie, with known vital status, may have incomplete follow-up of endpoints.

Complete follow up of the primary endpoint will be defined when the patient had a primary endpoint event, died from non-CV death (including undetermined death) or had complete event assessment on or after the PACD (ie, the patient was not censored due to incomplete follow-up of endpoints).

In addition to the number and percent of patients with complete follow-up, the proportion of total patient time with complete follow-up will be reported per treatment group.

Patient time with complete follow-up will be defined as time from randomisation until the earliest of first primary endpoint event, death, WoC, censoring where last complete event assessment is prior to PACD or PACD. The denominator, representing maximum complete follow-up, will be the time from randomisation until the earliest of first primary endpoint event, death or PACD.

4.2 Analysis Methods

4.2.1 Demographics and Baseline Characteristics

Demographic and baseline characteristics, including medical history, will be summarized, using frequency distributions and summary statistics based on the FAS, for each treatment group as well as for all patients combined. No statistical test will be performed for comparison of any baseline measurement among treatment groups.

4.2.2 Concomitant and Baseline Medication

Baseline medication is defined as medication with at least one dose taken before date of randomisation and with no stop date before date of randomisation.

Concomitant medication is defined as medications taken post randomisation, irrespective of IP.

The proportion of patients taking baseline and concomitant medication will be presented for the FAS per ATC class and treatment group. Summaries of prohibited medication, in this study limited to open label SGLT2 inhibitor taken while on IP, will be presented.

4.2.3 Analysis of the Primary Efficacy Variables

Dual primary analyses will be performed simultaneously for the primary composite endpoint, (1) in the full population based on the FAS as well as (2) in the LVEF < 60% subpopulation. The same procedure described below will be used for both of these analyses.

The primary variable is the time to first event included in the primary composite endpoint. The primary analysis will be based on the ITT principle using the FAS, including events with onset on or prior to PACD, adjudicated and confirmed by the CEA committee.

In the analysis of the primary composite endpoint, treatments (dapagliflozin versus placebo) will be compared using a Cox proportional hazards model with a factor for treatment group, stratified by T2D status at randomisation. The analysis will use WoC, non-CV death, last clinical event assessment and PACD for censoring of patients without any primary event as described in Section 3.1. The Efron method for ties and p-value based on the Wald statistic will be used. Event rates, p-value, HR, and 95% confidence interval will be reported.

The contribution of each component of the primary composite endpoint to the overall treatment effect will be examined. In the analysis of the components, all first event of the given type will be included irrespective of any preceding non-fatal composite event of a different type. Consequently, the sum of the number of patients with individual events in the component analysis will be larger than the number of patients with a composite outcome. Methods similar to those described for the primary analysis will be used to separately analyse the time from randomisation to the first occurrence of each component of the primary composite endpoint.

Kaplan-Meier estimates of the cumulative proportion of patients with event will be calculated and plotted, for the composite endpoint and for the individual components.

4.2.3.1 Subgroup Analysis of the Primary Endpoint

Exploratory subgroup analyses of the primary composite endpoint will be performed for the characteristics listed in Table 3 for both full population and LVEF < 60% subpopulation. A

test of interaction between randomised treatment group and the subgroup variable will be performed using Cox proportional hazard model stratified by T2D status at randomisation with factors for treatment group, the subgroup variable and the interaction between treatment and subgroup. In addition to the number and percent of patients with event, event rate estimate, HR with 95% confidence interval and p-value for each subgroup, the interaction p-value will be presented. Hazard ratio estimates, confidence intervals and p-values are not presented for subgroups with less than 15 events in total, both arms combined. HRs with confidence interval will be presented in a forest plot, including number of patients with event and interaction p-value. The p-values for the subgroup analyses and interaction will not be adjusted for multiple comparisons as the tests are exploratory and will be interpreted descriptively.

Table 3 Characteristics and Categories for Subgroup Analysis of the Primary Endpoint

Characteristic	Categories
Age at enrolment (years)	≤ median, > median
Sex	Male, Female
Race	White, Black or African American, Asian, Other
Geographic region	Asia (China, Japan, Taiwan, Vietnam)<br>Europe and Saudi Arabia (Belgium, Bulgaria, Czech Republic, France, Hungary, Netherlands, Poland, Romania, Russia, Saudi Arabia, Spain)<br>North America (Canada, US)<br>Latin America (Argentina, Brazil, Mexico, Peru)
NYHA class at enrolment	II, III/IV
LVEF at enrollment (%)	≤ 49, 50 to 59, ≥ 60
NT-proBNP at enrollment (pg/mL)	≤ median, > median
Randomised during hospitalisation for HF or within 30 days of discharge.	Yes, No
eGFR at enrolment (mL/min/1.73m^2)	< 60, ≥ 60
BMI at enrolment (kg/m2)	< 30, ≥ 30
T2D at enrolment [a]	Yes, No
SBP at randomisation	≤ median, > median
Atrial fibrillation or flutter at enrolment ECG	Yes, No

[a] The subgroup analysis by T2D status will be based on eCRF medical history record and exclude T2D as a stratification factor from the model

BMI, body mass index; ECG, electrocardiogram; eGFR, estimated glomerular filtration rate; HF, heart failure; LVEF, left ventricular ejection fraction; NT-proBNP, N-terminal pro b-type natriuretic peptide; NYHA, New York Heart Association; SBP, systolic blood pressure; T2D, type 2 diabetes

The subgroup analyses will be repeated for CV death and the HF event (hospitalisation for HF and urgent HF visit) component of the primary composite endpoint.

4.2.3.2 Sensitivity Analysis of the Primary Endpoint

Undetermined cause of death

A sensitivity analysis of the primary analysis where deaths adjudicated as 'undetermined' cause are considered as CV deaths and included as endpoint events will be performed.

Missing data and informative censoring

The time-to-event analysis using the Cox regression depends on the assumption of non-informative or ignorable censoring, corresponding to the missing-at-random assumption. The missing data in this context are patients who are prematurely censored due to WoC, LTFU or otherwise incomplete follow-up of endpoints. The amount of missing data will be described eg, in terms of the number of patients and patient time with incomplete follow-up as described in Section 4.1.5.

Patient retention and follow-up are at the forefront of study planning and conduct, and the amount of incomplete follow-up is expected to be small.

To assess the effect of incomplete follow up of the primary endpoint, a sensitivity analysis may be performed where time to event information is imputed for patients with premature censoring (censored before PACD due to WoC or incomplete primary event assessment). Event rates will be estimated separately in the two T2DM strata by an exponential distribution with constant hazard rate over time. Using the hazard ratio from the primary analysis, the event rates will be calculated for the dapagliflozin group, separately for the T2DM strata (by multiplying the corresponding placebo group rates by the hazard ratio estimated in the primary analysis). Using the estimated event rates, new event times will be simulated for patients with premature censoring from the exponential distribution. If the simulated time is in the interval from the censoring date to PACD (or death date, whichever came first), a new event will be imputed at the resulting event time. Otherwise, if the simulated time is outside the interval from the original censoring to PACD or death, the patient will be considered censored at PACD or death. The primary analysis will thereafter be conducted again, supplemented by the simulated time-to-event information. The process is to be repeated 1000 times and the resulting hazard ratios and standard errors will be combined using the Rubin's rule.

A tipping point analysis may be conducted to assess the robustness of the statistical significance of the primary analysis. While keeping the placebo event rates constant at the estimated values, the event rates in the dapagliflozin group will gradually be increased by increasing the hazard ratio from the primary analysis until the test of the primary endpoint no longer is statistically significant.

COVID-19

Subjects affected by COVID-19 infection will be defined by pre-specified preferred terms for adverse events associated with COVID-19 infection. A COVID-19 sensitivity analysis of the primary endpoint (and components) will be performed where the main analysis of the primary endpoint will be done, where patients and events are censored at the onset date of AE associated with COVID-19 infection. In this setting, onset of COVID-19 can be assumed to be unrelated to randomised treatment and as such should not introduce informative censoring while accounting for impact of COVID-19 infection in the main analysis.

4.2.4 Analysis of the Secondary Efficacy Variables

4.2.4.1 Analysis of Total Number of HF Events (First and Recurrent) and CV Death

The composite outcome of total number of HF events (first and recurrent) and CV death with onset on or prior to PACD, adjudicated and confirmed by the CEA committee, will be analysed by the semi-parametric proportional rates model (Lin et al 2000; known as the LWYY method) to test the treatment effect and to quantify the treatment difference in terms of the rate ratio with 95% confidence interval and p-value. If a HF event and CV death occurred at the same day, then only CV death will be counted.

In addition, the two components in the composite endpoint (total number of HF events and CV death) will be analysed separately to quantify the respective treatment effects and check the consistency between the composite and the components. For the analysis of total number of HF events component, occurrence of CV death can be regarded as semi-competing risk (informative censoring) and may introduce a bias in the treatment effect estimate for HF events (dilution of effect size if the drug has a positive effect on both components). To address this concern and to account for the correlation between the two components, the joint modelling (frailty model) approach (Rogers et al 2016) will be used for the component analyses. Non-parametric estimates of HF event rates over time allowing for death as terminal event will be provided as well (Ghosh and Lin 2000).

COVID-19

A COVID-19 sensitivity analysis of the first secondary endpoint (and components) will be performed where the main analysis LWYY will be applied and where patients and events are censored at the onset date of AE associated with COVID-19 infection. In this setting, onset of COVID-19 can be assumed to be unrelated to randomised treatment and as such should not introduce informative censoring while accounting for impact of COVID-19 infection in the main analysis.

4.2.4.2 Analysis of Change from Baseline at 8 Months in the KCCQ Total Symptom Score

Hypothesis testing

The composite rank-based endpoint representing the patients' vital status at 8 months and the change from baseline at 8 months in KCCQ-TSS in surviving patients, as defined in Section 3.2.2, will be analysed using the rank ANCOVA method (Stokes et al 2012) to test the null hypothesis of no difference in the distributions of ranked outcomes between the two treatment groups. Analysis will be stratified by T2D status at randomisation, and adjusted for the baseline KCCQ-TSS value as follows.

First the change from baseline at 8 months in KCCQ-TSS and vital status at 8 months, as well as values of the baseline KCCQ-TSS covariate will be transformed to standardised ranks within each T2D randomisation stratum, using fractional ranks and mean method for ties. Ranking for the composite endpoint will be done so that patients who died prior to the 8-month assessment are assigned the worst ranks within each stratum. Among the deceased, the relative ranking will be based on their last value of change from baseline in KCCQ-TSS while alive before deriving fractional ranks. In the ranking, patients who die prior to the first follow-up visit where KCCQ-TSS is assessed, at 1 month, will be defined as having a zero change from baseline while alive. Then, separate regression models will be fit to the ranked data for each randomisation stratum using a regression model for the ranked composite variable as dependent variable, adjusting for the ranked baseline covariate. Residuals from this regression model will be captured for testing of differences between treatment groups. The CMH test, stratified by T2D status at randomisation, using the values of the residuals as scores will be used to compare treatment groups.

KCCQ data missing for reasons other than death will be imputed as described in Section "Handling of missing KCCQ data".

The p-value from the CMH test of treatment effect at 8 months will be the used for the confirmatory testing of the secondary endpoint in the MTP described in Section 4.1.3.

COVID-19

Due to COVID-19 pandemic, on-site assessments could not be performed in a substantial number of sites, where some were done remotely and some cancelled. Furthermore, it could be assumed that lock-downs and other measures could impact PRO assessments. As a consequence, the main analysis of this endpoint includes the population with patients who had a planned or performed 8 month assessment (Visit 5) prior to the major COVID-19 outbreak, defined as 11th March 2020 (the date when WHO declared COVID-19 a pandemic) thus unaffected by the pandemic's possible impact on health-related quality of life (FDA 2020). The KCCQ-TSS in the presence of COVID-19 pandemic will be described.

Estimation of treatment effect

Win ratio

For a summary statistic that uses the same ranking as that used in the hypothesis test, but has a clinical interpretation, the WR and the corresponding 95% confidence interval (Wang and Pocock 2016) will be reported. It is noted that the WR differs from the statistic used for hypothesis testing, so that exact consistency is not expected between these two analyses, eg on rare occasions, the 95% confidence interval for WR could exclude unity while the p-value for the pre-planned hypothesis test could be > 0.05, or the hypothesis test could be < 0.05 with the confidence interval for WR including unity. Formal inference for the superiority of the treatment over control will be made only from the pre-planned hypothesis test based on the WR.

The win ratio represents the odds of having a more favourable outcome versus a less favourable outcome when assigned to the dapagliflozin 10 mg treatment group as opposed to placebo. More specifically, each patient in the dapagliflozin group is compared with each patient in the placebo group and each pair is labelled as “winner”, “loser”, or “tie”, depending on whether the patient on dapagliflozin has a more favourable, less favourable, or the same outcome, respectively, with respect to the composite ranked endpoint compared to the patient on placebo. Win ratio is defined as the ratio of the number of “winner” pairs to the number of “loser” pairs for the dapagliflozin arm. If the estimated win ratio is greater than 1 then the treatment effect is in favour of dapagliflozin.

The win ratio statistic adjusted for the randomisation stratification factor and baseline KCCQ-TSS will be obtained using the methodology in (Koch et al 1998, Kawaguchi et al 2011) for the stratified Mann-Whitney estimators for the comparison of two treatments with randomisation based covariance adjustment. The win ratio statistic will be calculated as Mann-Whitney odds, ie, $WR = MW / (1 - MW)$, where MW is the adjusted Mann-Whitney estimate. This transformation is monotonous in the domain of the Mann-Whitney estimate. The 95% confidence interval for the win ratio will be obtained by transforming the bounds of the confidence interval (Koch et al 1998) for the Mann-Whitney estimate, using the same transformation as for the win ratio.

Responder analysis

Number and percentage of patients in each treatment group will be summarised across the following categories, where change from baseline is defined as KCCQ-TSS at 8 months minus KCCQ-TSS at baseline:

Thirteen point improvement from baseline at 8 months in KCCQ-TSS, identified as a clinically meaningful improvement in anchor-based analyses (see Appendix A), vs no clinically meaningful improvement:

- Change from baseline in KCCQ-TSS ≥ 13 points, vs
- Death prior to the 8 months assessment or change from baseline in KCCQ-TSS < 13 points.

Five point deterioration from baseline at 8 months in KCCQ-TSS, identified as a clinically meaningful deterioration in anchor-based analyses (see Appendix A), vs no clinically meaningful deterioration:

- Death prior to the 8 months assessment or a negative change from baseline in KCCQ-TSS ≥ 5 points, vs
- Change from baseline at 8 months in KCCQ-TSS that is positive or, if negative, is smaller than 5 points.

Patients who had a baseline value of KCCQ-TSS ≥ 100 – 13 = 87 points (ie, too close to the "ceiling" to have a clinically meaningful improvement based on the instrument), will be defined as having achieved "responder status" for improvement only if the following conditions are both met: KCCQ-TSS remains ≥ 87 points at 8 months and KCCQ-TSS ≥ baseline at 8 months (ie, they had no deterioration from their baseline score). Similarly, for clinically meaningful deterioration, patients who had a baseline value of KCCQ-TSS ≤ 5 points (ie, too close to the "floor" to have a clinically meaningful deterioration based on the instrument), will be defined as having achieved "responder status" for deterioration only if KCCQ-TSS remains ≤ 5 points at 8 months and KCCQ-TSS ≤ baseline at 8 months (ie, they had no improvement from their baseline score).

The proportion of patients in the different KCCQ-TSS responder categories will be compared between treatment groups using a logistic regression model including treatment group, stratification variable (T2D at randomisation) and baseline KCCQ-TSS value. The observed number and proportion of KCCQ-TSS responders, odds ratio between treatment groups, its corresponding 2-sided 95% confidence interval and p-value estimated from each imputed dataset will be combined using Rubin's rule, and the combined results will be presented.

Additional responder analysis will be performed in the same way as described above, for 17 points improvement ("large improvement") and 14 points deterioration ("large deterioration"). These thresholds of clinically meaningful change from baseline KCCQ-TSS were derived from anchor-based analyses of blinded study data as described in Appendix A. In these analyses, "ceiling" and "floor" values are handled in an analogous way as for the analysis of 13 points improvement and 5 points deterioration.

Empirical cumulative distribution function plots will be presented by treatment group to summarize the distribution of change from baseline at 8 months in KCCQ-TSS values, where patients who die prior to the 8-month assessment will be represented with the value of -101 (a value below the worst possible change from baseline).

Handling of missing KCCQ data

The number of patients with missing vital status at 8 months is expected to be negligible. If some patients are LTFU or withdrew consent and have unknown vital status, the main analysis will be done with these patients assigned the worst ranks (same as deaths, described below).

In the context of analysing the composite ranked endpoint as described above, missing data may arise when patients miss the 8-month KCCQ assessment while remaining in the study during the 8-month assessment window (+/- 14 days will be used), or when patients withdraw consent from the study prior to 8 months. If a patient is known to have died prior to the 8-month assessment, the patient is considered to have a non-missing composite outcome and will be handled as described above (assigned the worst rank). Otherwise, patients who are alive at 8 months and have missing baseline or 8-month KCCQ assessments will have their missing KCCQ-TSS imputed using the multiple imputation methodology as follows.

Missing KCCQ-TSS values at baseline or at 8 months will be imputed under the Missing at Random assumption. The imputation will be done using a predictive mean matching multiple imputation model and a method of Fully Conditional Specification as implemented in the SAS Procedure MI (FCS statement). The predictive mean matching method ensures that the imputed values remain in the permissible range of the KCCQ-TSS values. Imputation will be done sequentially, ie, imputing each time point in their chronological order and the imputations at a given time point will be informed by preceding imputed time points. The imputation model will include the treatment group, T2D randomisation stratum, prior KCCQ-TSS (at baseline, month 1 and month 4), and three categorical variables representing the number of HF events (categorised as 0, 1 or ≥ 2) in the intervals from randomisation to 1 month, from 1 to 4 months, and from 4 to 8 months, respectively, depending on the time point being imputed. Occurrences of HF events will be determined based on the investigator-reported potential HF events. Auxiliary variables related to HF events are included in the imputation model to improve the imputation accuracy, because the occurrence of HF events is expected to be associated with HF symptoms as assessed by KCCQ-TSS.

The number of closest observations used to sample an imputed value by the predictive mean matching method will be 5 (SAS default setting).

Each imputed dataset will be analysed using the methods described in the "Hypothesis testing" and "Estimation of treatment effect" sub-sections above. The results from multiple imputed datasets will be combined using Rubin's rule as implemented in the SAS Procedure MIANALYZE.

- In the analysis of rank ANCOVA, the CMH tests statistic used for the hypothesis test has a chi-square distribution. In order to apply Rubin's combination rule, which assumes approximate normal distribution of the statistics being combined, a normalising Wilson-

Hilferty transformation will be applied to the CMH test statistics from each imputed dataset (Ratitch et al 2013). The standardized transformed statistic will be computed as follows:

$$st_{wh\_cmh}{}^{(m)} = \frac{\sqrt[3]{\frac{cmh^{(m)}}{df}} - \left(1 - \frac{2}{9 \times df}\right)}{\sqrt[2]{\frac{2}{9 \times df}}}$$

where $cmh^{(m)}$ is the CMH statistic from the mth imputed dataset and df is the number of degrees of freedom associated with the statistic (in this case equal 1). The transformed statistics are approximately normally distributed with mean of 0 and variance of 1 and can be combined using Rubin's rule.

- For the estimation of the win ratio, a combined Mann-Whitney estimate and its standard error will first be obtained by applying Rubin's rule to the corresponding estimates from multiple imputed datasets. Then the win ratio and its 95% confidence interval will be obtained based on the combined Mann-Whitney estimate and its standard error as previously described.
- For the summaries of number and percentage of subjects in the categories of significant improvement and deterioration from baseline, the number and percent of subjects with actual observed improvement and observed deterioration/death respectively will be reported. The estimation of odds ratio and confidence intervals for the KCCQ-TSS responder analyses will use the imputation datasets created for the main analysis. Therefore, deaths will be defined as non-responders, and responder status will be determined based on the imputed KCCQ-TSS values for the patients who have missing KCCQ-TSS due to reasons other than death.

Supportive analyses and sensitivity analyses for KCCQ

The number and percent of patients who die prior to the 8-month assessment will be summarized by treatment group.

Descriptive statistics of scores and change from baseline at 1, 4 and 8 months will be presented for TSS, overall summary score, clinical summary score and domains (physical limitation, symptom stability, symptom frequency, symptom burden, quality of life, self-efficacy and social limitation).

The testing and estimation described for change from baseline at 8 months in KCCQ-TSS, will be repeated in an exploratory fashion for change from baseline in KCCQ-TSS at 1 and 4 months, and for the overall summary score and clinical summary scores at 1, 4 and 8 months.

4.2.4.3 Analysis of CV death

Time to CV death will be analysed using Cox regression in the same manner as the primary composite endpoint, with stratification for T2D status at randomisation. The analysis will include CV deaths, confirmed in adjudication, occurring on or prior to PACD. Patients who did not die from CV death, will be censored at the earliest of death due to other cause, WoC, PACD, or for any patients who are LTFU, at last date known to be alive.

COVID-19

As part of the COVID-19 related sensitivity analysis of the primary endpoint, the component CV death will be reported (Section 4.2.3.2).

4.2.4.4 Analysis of All-Cause Mortality

Time to death from any cause will be analysed using Cox regression in the same manner as the primary composite endpoint, with stratification for T2D status at randomisation. The analysis will include deaths from any cause occurring on or prior to PACD. Patients who are alive will be censored at PACD, or for any patients who are LTFU, at last date known to be alive.

4.2.5 Analysis of Safety Variables

Analysis set

For safety analyses, all summaries will be based on the SAS (Section 2.1.2).

Exposure

The total exposure to IP will be defined as the length of period on IP, calculated for each patient as date of last dose – date of first dose +1.

An alternative measure where days of interruption are removed will be calculated and termed actual exposure.

Total and actual exposure will be presented descriptively.

Treatment periods

The summaries for the on-treatment period will include events with an onset date on or after first dose of randomised IP and on or before 30 days after last dose of IP. Additional presentations will include all events with onset on or after first dose of IP regardless of whether patients are on or off IP at the time of the event (the "on- and off-" treatment period.). Patients who complete the study on IP will discontinue treatment on the SCV. Thus, there will in general be no events after completion of the IP period, and censoring of events for on-treatment analysis affects only patients who prematurely and permanently discontinue IP.

All summaries of AEs described in Section 4.2.5.1 to 4.2.5.4 below will be presented for the on-treatment period and on- and off- treatment period.

4.2.5.1 Adverse Events

The on-treatment period was used for primary analysis of all safety variables, except for amputations and preceding events, for which the on- and off-treatment period was considered the primary approach.

In addition to SAEs, the collection of AEs that are not serious includes myocardial infarction, unstable angina, stroke, major hypoglycaemic events, potential DKAs, amputations, AEs leading to amputation, and preceding events, AEs leading to a potential endpoint, DAEs, and AEs which are the reason for interruption of IP (see Section 3.3). Thus, summaries of AEs will be limited to these categories and general summaries of all non-serious AEs are not planned.

AEs will be classified according to MedDRA by the medical coding team at AstraZeneca data management center, using MedDRA 24.1.

Summaries by SOC and PT will be sorted by international order for SOC and by descending order of PT in the dapagliflozin treatment group.

No statistical tests to compare crude AE frequencies between treatment groups are planned. A summary table of the total number and percent of patients with AE with outcome death, AEs of definite or probable DKA, any major hypoglycemic event, SAE, DAE, AE leading to temporary interruption of IP, AEs possibly related to IP, amputations and preceding events per treatment group will be provided.

Amputations, AEs leading to amputations, and preceding events (see Section 3.3) will be presented in summary tables including the number and percent of patients with any event in the AE category, SAE, DAE and AE leading to interruption, and tabulated with frequency by PT.

In addition to presentations of the number of patients with event, the total number of events counting multiple events per subject will be presented.

All potential events of DKA will be submitted to an independent DKA Adjudication Committee. The adjudicated outcome, definite or probable, will be considered the main analysis for DKA.

For major hypoglycaemic events a summary table including the total number of subjects with events, the number and percent of patients with event in the AE intensity category, SAE, DAE, AE leading to interruption, possible relation to IP will be presented. The presentation of on-treatment events, on- and off-treatment presentations will be provided for all major hypoglycaemic events.

For AEs leading to amputations and preceding events, DKA and major hypoglycaemic events, event rate per 100 subject years will also be presented, calculated as 100 times the number of patients with event divided by the total duration of treatment (including 30 days after last dose) in the given group for the on-treatment presentation, and total duration of follow-up in the given group for on and off treatment.

Events of genital area infections and necrotising fasciitis to be medically assessed in a blinded fashion prior to clinical data lock as potential events of Fournier's gangrene will be presented in a summary table including the number and percent of patients with any event in the SAE or DAE category, and tabulated with frequency by PT.

4.2.5.2 Serious Adverse Events

SAEs will be presented as described below both on treatment and on and off treatment.

The number and percent of patients with SAEs will be presented by SOC, PT and treatment group. The most common SAEs will also be presented by PT only.

AEs with outcome death will be presented separately by SOC and PT.

4.2.5.3 Adverse Events Leading to Discontinuation or Interruption of Investigational Product

The number and percent of patients with event will be presented by SOC and PT for AEs leading to discontinuation of IP and AEs leading to temporary interruption (separately for the two categories based on action taken "Drug Permanently Discontinued" and "Drug Interrupted" respectively, recorded in the CRF AE module).

4.2.5.4 Laboratory Evaluation and Vital Signs

Summaries of creatinine and calculated eGFR will be based on creatine samples analysed at the central laboratory.

The result and the change from baseline of creatinine, eGFR and vital signs, will be summarized by treatment group at each visit (excluding PTDV and SCV) with scheduled measurement (see Section 3.4) using descriptive statistics, including n, mean, SD, range, median, and quartiles.

4.2.6 Analysis of Exploratory Objectives

Time to the first occurrence of hospitalisation from any cause will be analysed with the same method as the primary endpoint, based on information on the SAE eCRF form.

The proportion of patients with worsened (higher) NYHA class at 8 months compared to baseline, including patients who died prior to 8 months in the worsened category, versus patient with improved or unchanged NYHA class, will be analyzed by logistic regression with

treatment group, baseline NYHA class and T2D status at randomisation as factors, presented as an odds ratio with corresponding 95% confidence interval. Only NYHA assessments made at site or through phone visits with the patient to be used in analyses.

Change from baseline to each scheduled assessment visit (see Section 3.4) for body weight, SBP and eGFR will be analysed with a MMRM. All non-missing visit data will be used, including measurements after discontinuation of IP. The model will include terms for treatment group, visit, visit by treatment group interaction, the baseline measurement and T2D stratification status at randomisation as covariates. The model will be used to derive a least-squares estimate of the treatment difference with 95% confidence interval and corresponding two-sided p-value. Missing data will not be imputed.

For eGFR, the MMRM model above will additionally be used to derive the "total" slopes (between randomisation and eg, 1 year and 2 years respectively) and the "chronic" slopes (between a post randomisation time point to eg, 1 year and 2 years respectively) will be estimated via linear contrasts.

The analysis of change from baseline in KCCQ clinical summary score, overall summary score and KCCQ-TSS sub-scores (symptom burden and symptom frequency) will follow the analysis of KCCQ-TSS in Section 4.2.4.2. QoL score will be summarised using descriptive statistics.

EQ-5D-5L derived utility score will be summarised by descriptive statistics, and used for health economic modelling and reported in a separate health economic report.

5 INTERIM ANALYSES

An interim analysis is planned to be performed including approximately 67% of the target number of patients with adjudicated primary endpoint events (approximately 748 events). There will in principle be one planned interim analysis for efficacy, with the possibility of the DMC to conduct subsequent interim analysis if they deem necessary. The significance level for final analysis will be based on the actual number of interim analyses. The interim analysis will assess superiority of dapagliflozin to placebo. The interim analysis will have a nominal two-sided alpha level of 0.2%. At the interim analysis, the primary composite endpoint will be tested in the full study population at the specified alpha level. If superiority is achieved for the primary endpoint, then the superiority of dapagliflozin to placebo on CV deaths will be tested in the full study population at a two-sided level of 0.2%. If CV death is significant, then an action is triggered whereby the DMC will evaluate the totality of the efficacy data and safety data, to determine if benefit is unequivocal and overwhelming such that the DMC recommends ending the study.

If the interim analysis leads to a decision to terminate the study early based on pre-defined stopping guidelines, the executive committee will define a PACD, on or after which SCVs will commence. The study report will be based on all events occurring on prior to the PACD.

If the study is stopped at the efficacy interim analysis, testing of remaining secondary endpoints will be performed on the final database in the full population only, in fixed sequence described in the right branch of Figure 2 (Section 4.1.3) at two-sided significance level 0.2%.

6 CHANGES OF ANALYSIS FROM PROTOCOL

The alpha for final analysis adjusted for interim analysis at alpha 0.2% will be set to 5% minus 0.2% = 4.8%, rather than 4.98% as determined by the Haybittle-Peto function for 67% of events (Sections 9.1 and 9.5 of the CSP).

7 REFERENCES

Burman et al 2009
Burman CF, Sonesson C, Guilbaud O. KF. A recycling framework for the construction of Bonferroni-based multiple tests. Stat Med. 2009;28:739–61.

Coon and Cook 2018
Coon CD, Cook KF. Moving from significance to real-world meaning: methods for interpreting change in clinical outcome assessment scores. Qual Life Res 2018 Jan;27(1):33–40.

FDA 2020
Statistical considerations for clinical trials during the COVID-19 public health emergency. Guidance for industry. June 2020. US Department of Health and Human Services. Food and Drug Administration.

Fitchett et al 2016
Fitchett D, Zinman B, Wanner C, Lachin JM, Hantel S, Salsali A, et al. Heart failure outcomes with empagliflozin in patients with type 2 diabetes at high cardiovascular risk: results of the EMPA-REG OUTCOME® trial. Eur Heart J 2016;14;37(19):1526-34.

Ghosh and Lin 2000
Ghosh D, Lin DY. Nonparametric analysis of recurrent events and death. Biometrics 2000; 56:554–562.

Green et al 2000
Green CP, Porter CB, Bresnahan DR, Spertus JA. Development and Evaluation of the Kansas City Cardiomyopathy Questionnaire: A New Health Status Measure for Heart Failure. JACC, 2000 Apr;35(5):1245–55.

Hicks et al 2018
Hicks KA, Mahaffey KW, Mehran R, Nissen SE, et al. Cardiovascular and Stroke Endpoint Definitions for Clinical Trials. J Am Coll Cardiol 2018;71:1021–34.

Kawaguchi et al 2011
Kawaguchi A, Koch G, Wang X. Stratified Multivariate Mann-Whitney Estimators for the Comparison of Two Treatments with Randomization Based Covariance Adjustment. Statistics in Biopharmaceutical Research, 2011;3(2):217–231.

Koch et al 1998
Koch GG, Tangen CM, Jung JW, Amara IA. Issues for covariance analysis of dichotomous and ordered categorical data from randomized clinical trials and non-parametric strategies for addressing them. Statist. Med 1998;17(15-16):1863–1892.

Kristensen et al 2015
Kristensen SL, Jhund PS, Køber L, McKelvie RS, Zile MR, Anand IS, et al. Relative importance of history of heart failure hospitalisation and N-terminal Pro–B-type natriuretic peptide level as predictors of outcomes in patients With heart failure and preserved ejection fraction. JACC Heart Fail 2015 Jun;3(6):478–86.

Kristensen et al 2017
Kristensen SL, Mogensen UM, Jhund PS, Petrie MC, Preiss D, Win S, et al. Clinical and echocardiographic characteristics and cardiovascular outcomes according to diabetes status in patients with heart failure and preserved ejection fraction: a report from the I-Preserve Trial (Irbesartan in Heart Failure With Preserved Ejection Fraction). Circulation. 2017;135(8):724–35.

Levey at al 2009
Levey AS, Stevens LA, Schmid CH, Zhang YL, Castro AF 3rd, Feldman HI, Kusek JW, Eggers P, VanLente F, Greene T, Coresh J; CKD-EPI (Chronic Kidney Disease Epidemiology Collaboration). A new equation to estimate glomerular filtration rate. Ann Intern Med. 2009 May 5;150(9):604–12.

Lin et al 2000
Lin DY, Wei LJ, Yang I, Ying Z. Semiparametric regression for the mean and rate functions of recurrent events. Journal of the Royal Statistical Society: Series B (Statistical Methodology) 2000;62(4):711–730.

Pfeffer et al 2015
Pfeffer MA, Claggett B, Assmann SF, Boineau R, Anand IS, Clausell N, et al. Regional Variation in Patients and Outcomes in the Treatment of Preserved Cardiac Function Heart Failure With an Aldosterone Antagonist (TOPCAT) Trial. Circulation. 2015;131:34–42.

Ratitch et al 2013
Ratitch B, Lipkovich I, O’Kelly M. Combining Analysis Results from Multiply Imputed Categorical Data. PharmaSUG 2013 – Paper SP03

Rogers et al 2016
Jennifer K Rogers, Alex Yaroshinsky, Stuart J Pocock, David Stokar and Janice Pogoda. Analysis of recurrent events with an associated informative dropout time: Application of the joint frailty model. Statist. Med. 2016;35:2195–2205.

Rådholm et al 2018
Rådholm K, Figtree, Perkovic V, Solomon SD, Mahaffey KW, et al. Canagliflozin and Heart Failure in Type 2 Diabetes Mellitus: Results From the CANVAS Program (Canagliflozin Cardiovascular Assessment Study). Circulation. 2018;138:458–468. https://doi.org/10.1161/CIRCULATIONAHA.118.034222

Spertus et al 2005
Spertus J, Peterson E, Conard MW, Heidenreich PA, Krumholz HM, Jones P, et al. Monitoring clinical changes in patients with heart failure: A comparison of methods. Am Heart J 2005;150:707–15.

Spiessen and Debois 2010
Spiessen B, Debois M. Adjusted significance levels for subgroup analyses in clinical trials. Cont Clin Trials 2010; 31:647–656.

Stokes et al 2012
Stokes ME, Davis CS, Koch GG. Categorical Data Analysis Using the SAS System. Cary, NC: SAS Institute Inc.

Wang and Pocock 2016
Wang D, Pocock S. A win ratio approach to comparing continuous non-normal outcomes in clinical trials. Pharmaceut. Statist. 2016;15:238–245.

Appendix A Estimation of Clinically Meaningful Thresholds for KCCQ Total Symptom Score

A 1 Methods

Thresholds for CMWPC will be estimated according to predefined algorithms using an anchor-based approach, supplemented with graphical visualisations of the distribution across anchor categories. Clinically meaningful thresholds will be estimated for change from baseline KCCQ-TSS at 8 months.

This appendix describes the methods which were applied to blinded study data prior to database lock and unblinding of the study, with results and derived thresholds presented in this SAP prior to the interim analysis. The threshold analyses were performed on the FAS population used in the main analysis for KCCQ (the population with patients who had a planned Visit 5, ie, at 8 months, prior to the major COVID-19 outbreak; see 4.2.4.2), on blinded study data across both treatment arms only including patients with complete data at baseline and 8 months.

<u>Anchor-based approaches</u>

Anchor-based approaches estimate a threshold by 'anchoring' the results on a separate variable, often a patient-reported outcome. The anchor-based analysis will employ the PGIS in HF symptoms. Meaningful change will be evaluated using observed scores according to a predefined algorithm. The responses to PGIS at baseline and 8 months will be used in the analysis.

<u>Categorisation of anchors</u>

The change from baseline PGIS at 8 months will be categorized and categories will be collapsed in different ways, to provide a clearer distinction between patients who have and have not experienced a meaningful change according to this anchor.

The ordinal responses to PGIS at baseline and 8 months will be assigned the following numeric values:

- 1 ('no symptoms')
- 2 ('very mild')
- 3 ('mild')
- 4 ('moderate')
- 5 ('severe')
- 6 ('very severe')

Change from baseline PGIS at 8 months will be categorized as small, moderate or large improvement/deterioration or stable as defined in Table A1.

Table A1 Categories of Change from Baseline PGIS in Heart Failure Symptoms at 8 Months

PGIS at baseline		PGIS at 8 months					
		No symptoms	Very mild	Mild	Moderate	Severe	Very Severe
		1	2	3	4	5	6
**No symptoms**	1	0<br>Stable	+1<br>SD	+2<br>MD	+3<br>LD	+4<br>LD	+5<br>LD
**Very mild**	2	-1<br>SI	0<br>Stable	+1<br>SD	+2<br>MD	+3<br>LD	+4<br>LD
**Mild**	3	-2<br>MI	-1<br>SI	0<br>Stable	+1<br>SD	+2<br>MD	+3<br>LD
**Moderate**	4	-3<br>LI	-2<br>MI	-1<br>SI	0<br>Stable	+1<br>SD	+2<br>MD
**Severe**	5	-4<br>LI	-3<br>LI	-2<br>MI	-1<br>SI	0<br>Stable	+1<br>SD
**Very severe**	6	-5<br>LI	-4<br>LI	-3<br>LI	-2<br>MI	-1<br>SI	0<br>Stable

LD, large deterioration; LI, large improvement; MD, moderate deterioration; MI, moderate improvement; SD, small deterioration; SI, small improvement

The categories in Table A1 will be further collapsed as

- ‘moderate or large deterioration’ in the categorisation with 5 categories (version A)
- ‘small or moderate deterioration’ in the categorisation with 5 categories (version B)
- ‘small or moderate improvement’ in the categorisation with 5 categories (version B)
- ‘moderate or large improvement’ in the categorisation with 5 categories (version A)

The change from baseline KCCQ-TSS at 8 months, will be used repeatedly in the anchor-based analyses. To explore the adequateness of each anchor categorisation, the Spearman correlation coefficient between change from baseline KCCQ-TSS and change from baseline PGIS at 8 months will be assessed.

The larger the correlation coefficient between an anchor and the endpoint, the greater the confidence in the classifications. An anchor is considered adequate if it has a correlation coefficient of 0.3 or greater (Coon and Cook 2018).

Descriptive statistics (mean, SD, median, quartiles, minimum and maximum) and an eCDF is presented for each categorisation in Section A 2. The eCDF curves display a continuous plot

of the change from baseline on the horizontal axis, and the cumulative proportion of patients experiencing changes from baseline up to that level, on the vertical axis. If the eCDF curves show very poor distinction between categories, they may be complemented with curves illustrating the probability density function for that categorisation.

Establishing the clinically meaningful threshold

The various estimates from the different streams of evidence (tables and plots of the distribution) will be examined for convergence in an effort to triangulate onto a single threshold value which represents CMWPC (for improvement and deterioration, respectively) and the KCCQ-TSS responder analysis will be performed for this threshold. However, if the values are too disparate, a range of clinically relevant thresholds may be identified. CMWPC thresholds identified will be indicated in the eCDF for change from baseline KCCQ-TSS by treatment, in the unblinded results, and responder analysis will be performed for the thresholds.

A 2 Summary of Results of Anchor-Based Analysis on Blinded Study Data

The anchor-based analysis of change from baseline KCCQ-TSS at 8 months in different categories of change from baseline PGIS at 8 months, is presented in Table A2. As this analysis is done on blinded study data and only includes patients with observed values for both KCCQ-TSS and PGIS at 8 months (patients who died and all other patients with missing data are excluded), the “mean” is selected as a representation of the average of a group. This anchor-based analysis indicates that small or moderate improvement corresponds to a mean increase in KCCQ-TSS of 13 points. A large improvement in PGIS corresponds to a mean increase in KCCQ-TSS of about 17 points. A large deterioration in PGIS corresponds to a mean decrease in KCCQ-TSS of about 14 points, whereas a moderate deterioration in PGIS corresponds to a mean decrease in KCCQ-TSS of 5 points. It is important to note that the group of patients who were categorized as being “stable” in terms of their HF symptoms at 8 months had a mean increase in KCCQ-TSS of almost 5 points.

In the responder analysis of the third secondary efficacy endpoint, change from baseline measured at 8 months in the TSS of the KCCQ (Section 4.2.4.2), an increase of 13 points or more in KCCQ-TSS will be considered a clinically meaningful improvement and a decrease of 5 points or more will be considered a clinically meaningful deterioration. The anchor-based analysis and the distribution curves indicate that a “small” improvement cannot be distinguished from a “moderate” improvement, while they are both clearly separated from the “stable” category. Likewise, the anchor-based analysis and distribution curves indicate that a “small” deterioration cannot be distinguished from the “stable” category. The Spearman’s correlation coefficient between change from baseline at 8 months in KCCQ-TSS and PGIS was around 0.29-0.30, where a correlation of 0.3 or greater between an anchor and the anchored scale is considered adequate (Coon and Cook 2018).

Table A2 Distribution of Change from Baseline KCCQ-TSS at 8 months by Change from Baseline PGIS 8 Months

	N	(%)	Mean	SD	Min	Q1	Median	Q3	Max	Correlation [a]
PGIS at 8 Months: 7										0.29
Large Improvement	120	(6)	17.4	22.51	-54.2	0.5	15.1	32.8	70.8	
Moderate Improvement	275	(13)	12.9	20.13	-76.0	0.0	12.5	25.0	72.9	
Small Improvement	453	(21)	13.0	19.63	-47.9	0.0	11.5	24.0	85.4	
Stable	811	(38)	4.5	19.01	-64.6	-5.2	2.1	16.7	66.7	
Small Deterioration	277	(13)	1.7	17.32	-37.5	-8.3	0.0	11.5	55.2	
Moderate Deterioration	111	(5)	-4.7	20.43	-59.4	-16.7	-4.2	6.3	58.3	
Large Deterioration	64	(3)	-13.7	27.85	-91.7	-30.2	-7.8	4.2	29.2	
PGIS at 8 Months: 5 Categories (collapsing “moderate” and “large”)										0.29
Moderate or Large	395	(19)	14.3	20.96	-76.0	0.0	12.5	27.1	72.9	
Small Improvement	453	(21)	13.0	19.63	-47.9	0.0	11.5	24.0	85.4	
Stable	811	(38)	4.5	19.01	-64.6	-5.2	2.1	16.7	66.7	
Small Deterioration	277	(13)	1.7	17.32	-37.5	-8.3	0.0	11.5	55.2	
Moderate or Large	175	(8)	-8.0	23.74	-91.7	-20.8	-4.2	5.2	58.3	
PGIS at 8 Months: 5 Categories (collapsing “small” and “moderate”)										0.30
Large Improvement	120	(6)	17.4	22.51	-54.2	0.5	15.1	32.8	70.8	
Small or Moderate	728	(34)	13.0	19.81	-76.0	0.0	11.5	25.0	85.4	
Stable	811	(38)	4.5	19.01	-64.6	-5.2	2.1	16.7	66.7	
Small or Moderate	388	(18)	-0.1	18.46	-59.4	-10.4	0.0	10.4	58.3	
Large Deterioration	64	(3)	-13.7	27.85	-91.7	-30.2	-7.8	4.2	29.2	

[a] Absolute value of the Spearman correlation coefficient for change from baseline KCCQ-TSS at 8 months and change from baseline PGIS at 8 months with each categorisation.

Categories of change from baseline PGIS at 8 months as defined in Table A1.

KCCQ, Kansas City Cardiomyopathy Questionnaire; PGIS, patient global impression of severity; TSS, total symptom score

The eCDF curves in Figure A1 demonstrate a clear separation between all categories of improvement and the “stable” category, in the interval between 5 and 40 points increase in KCCQ-TSS at 8 months, where separation is expected for these curves. However, the separation is less distinct between the categories of “small” and “moderate” improvement. For deterioration, the “large” and “moderate” deterioration categories are clearly separated from the “small” and the “stable” category, in the interval between 5 and 40 points decrease in KCCQ-TSS at 8 months, where separation is expected for these curves. The combined “moderate or large” categories of deterioration and improvement in Figure A2 are separated

from the "stable" category. This is also observed for combined "small or moderate" categories of deterioration and improvement in Figure A3.

Figure A1 Empirical Cumulative Distribution Function for Change from Baseline KCCQ-TSS at 8 Months versus Change from Baseline PGIS at 8 Months with 7 Categories

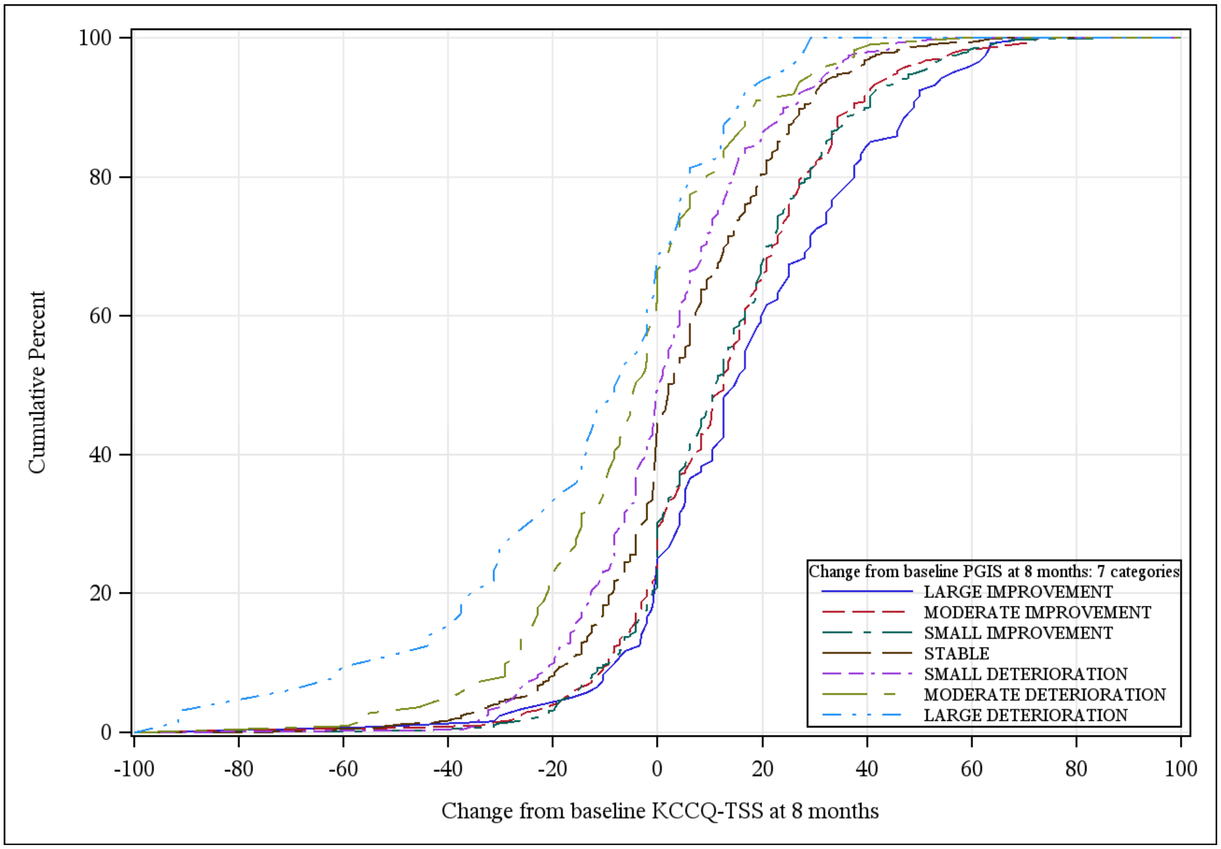

KCCQ, Kansas City Cardiomyopathy Questionnaire; PGIS, patient global impression of severity; TSS, total symptom score

Figure A2 Empirical Cumulative Distribution Function for Change from Baseline KCCQ-TSS at 8 Months Versus Change from Baseline PGIS at 8 Months with 5 Categories (Collapsing "Moderate" and "Large")

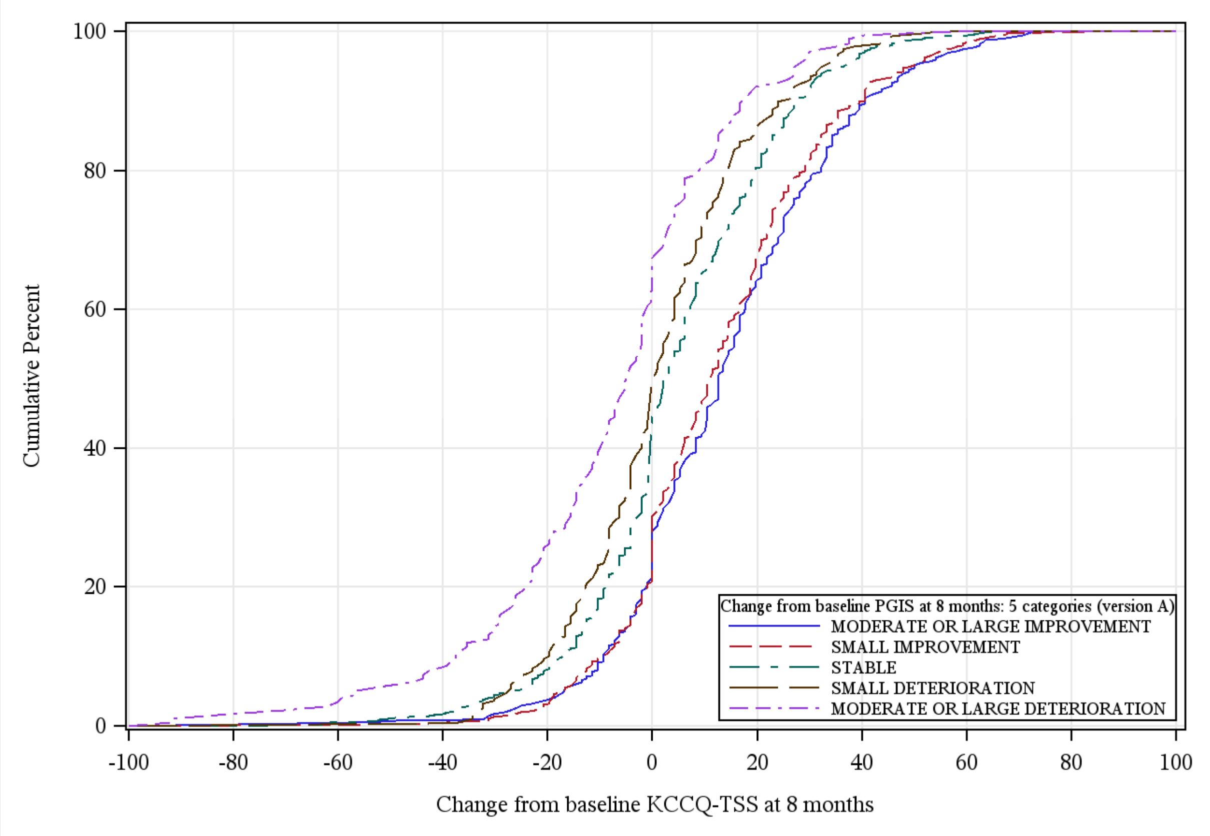

KCCQ, Kansas City Cardiomyopathy Questionnaire; PGIS, patient global impression of severity; TSS, total symptom score

Figure A3 Empirical Cumulative Distribution Function for Change from Baseline KCCQ-TSS at 8 months Versus Change from Baseline PGIS at 8 Months with 5 Categories (Collapsing "Small" and "Moderate")

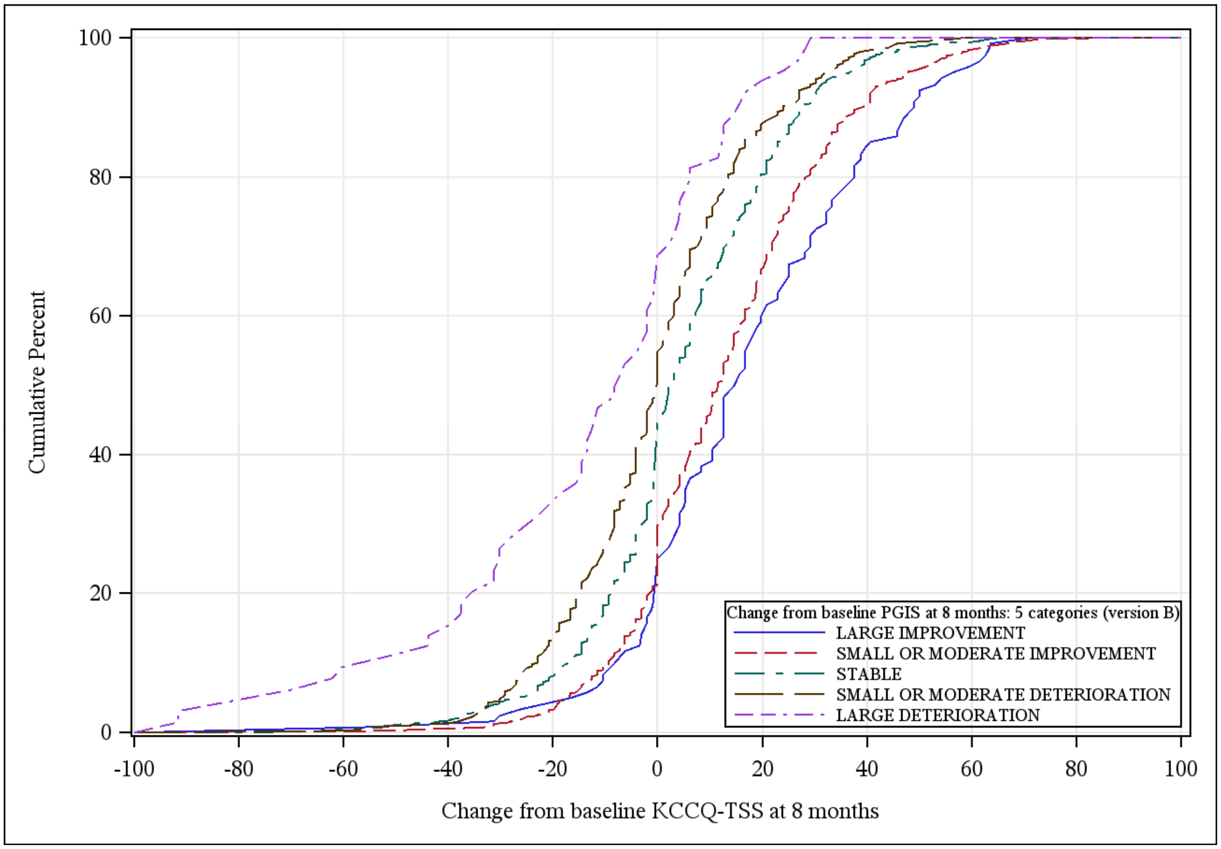

KCCQ, Kansas City Cardiomyopathy Questionnaire; PGIS, patient global impression of severity; TSS, total symptom score

A 3 Summary of Results of Distribution-Based Analysis on Blinded Baseline Study Data

Distribution-based methods (0.5 SD and 1 SEM) were used to explore the MCID in the KCCQ-TSS, in patients with HFpEF. The MCID is a value to which between-group differences in average change from baseline are compared, to assess clinical relevance of the difference between treatment groups.

The SEM was calculated as $SEM = \sigma_x * \sqrt{1 - r_{xx}}$, where σ_x is the SD at baseline and r_{xx} is the reliability (internal consistency) of the scale at baseline. The internal consistency of the KCCQ-TSS was assessed by Cronbach's alpha. The distribution-based analyses indicated that 0.5 SD (based on Cohen's "medium" effect size) of the baseline KCCQ-TSS score was equal to 11.0 and that 1 SEM was equal to 8.6 (Table A3). Based on these distribution-based analyses, a rounded mid-point between these values of 10 points is expected to represent a

MCID for KCCQ-TSS in patients with HFpEF. The MCID will not be used to inform responder analyses, as the MCID is not based on within-patient change and is therefore not appropriate for assessing an individual's "response".

Table A3 Distribution-Based Cut-offs for a Minimal Clinically Important Difference in the KCCQ-TSS

	N_1	One-half SD	N_2	1 SEM
**Baseline KCCQ-TSS**	4730	11.0	4562	8.6

N_1 The SD is based on the number of patients with an observed baseline KCCQ-TSS.

N_2 The SEM is based on the number of patients with an observed scorable responses to each of the items in the KCCQ-TSS.

KCCQ, Kansas City Cardiomyopathy Questionnaire; N, number of patients in treatment group; SD, standard deviation, SEM, standard error of measurement; TSS, total symptom score

Academic Statistical Analysis Plan	
Study Code	D169CC00001
Edition Number	1.3
Date	April 15, 2022

DELIVER: Academic Statistical Analysis Plan

Dapagliflozin Evaluation to Improve the LIVEs of Patients with PReserved Ejection Fraction Heart Failure

An International, Double-blind, Randomised, Placebo-Controlled Phase III Study to Evaluate the Effect of Dapagliflozin on Reducing CV Death or Worsening Heart Failure in Patients with Heart Failure with Preserved Ejection Fraction (HFpEF)

DELIVER: Academic Statistical Analysis Plan

Dapagliflozin Evaluation to Improve the LIVEs of Patients with PReserved Ejection Fraction Heart Failure

An International, Double-blind, Randomised, Placebo-Controlled Phase III Study to Evaluate the Effect of Dapagliflozin on Reducing CV Death or Worsening Heart Failure in Patients with Heart Failure with Preserved Ejection Fraction (HFpEF)

DELIVER CO-CHAIRS, STEERING COMMITTEE

Scott D. Solomon, MD
Brigham and Women's Hospital

Scott Solomon (Apr 15, 2022 15:38 EDT) Apr 15, 2022

Signature Date

John J.V. McMurray, MD
University of Glasgow

Apr 15, 2022

Signature Date

Table of Contents

1. INTRODUCTION 4
2. CLINICAL ENDPOINTS OF INTEREST 4
3. LABORATORY-BASED ENDPOINTS OF INTEREST 5
4. BREAKDOWN OF ENDPOINTS 6
5. SUBGROUPS 6
5. ALTERNATIVE ANALYTIC APPROACHES 7
6. COVID-19 7
7. META-ANALYSES 9

1. INTRODUCTION

DELIVER is an international, multicentre, parallel group, event-driven, randomized, double-blind trial in patients with chronic heart failure and left ventricular ejection fraction (LVEF) >40%, comparing the effect of dapagliflozin 10 mg once daily, vs. placebo, in addition to standard of care. Patients with or without diabetes, with signs and symptoms of heart failure, a LVEF >40%, elevation in natriuretic peptides and evidence of structural heart disease are eligible. The primary endpoint is time-to-first cardiovascular death or worsening heart failure event (heart failure hospitalization or urgent heart failure visit), and will be assessed in dual primary analyses – the full population and in those with LVEF <60%. The study is event-driven and will target 1117 primary events. A total of 6,263 patients have been randomized.

The DELIVER executive committee has developed this academic statistical analysis plan (ASAP) that describes pre-specified analyses that were not described in the DELIVER regulatory SAP (rSAP). General principles outlined in the regulatory SAP will be followed unless specified otherwise here. This document is meant to supplement and complement the regulatory SAP and delineate all analyses that were pre-specified prior to database lock. When relevant, analyses will be conducted based on the pooled DAPA-HF and DELIVER dataset to examine the effects of dapagliflozin in a broad range of patients with HF.

2. CLINICAL ENDPOINTS OF INTEREST

In addition to the efficacy and safety variables listed in the rSAP, the effect of dapagliflozin on the following endpoints will be explored. These events that are imbalanced between arms may be analyzed as time-to-event to better understand the time course. All endpoints will be assessed in the full cohort and in the LVEF < 60% subgroup. These include:

- Days alive and out of the hospital
- Quality of life-adjusted days alive and out of the hospital
- Investigator reported vs. CEC-adjudicated endpoints
- Time to onset of benefit of dapagliflozin
- New diuretic initiation, discontinuation, and dose changes
- New onset atrial fibrillation
- In the T2D subgroup, new glucose lowering therapy initiation and changes in insulin dose (in those on insulin at baseline)
- In the non-T2D subgroup, new diagnosis of diabetes
- Signs and symptoms of HF
- Patient Global Impression of Severity
- Target risk factor control (for blood pressure, smoking, antiplatelet/anticoagulant therapy)
- Cardiac ischemic events including myocardial infarction, unstable angina, unplanned coronary revascularization, and stroke
- Hyperkalemia as a reported adverse event and initiation of new potassium-lowering therapy
- Acute kidney injury as a reported adverse event and initiation of dialysis
- Anemia and requirement for blood transfusion as reported adverse events
- Gout as an adverse event and initiation of new uric acid-lowering therapy
- KCCQ Overall Summary Score at 1, 4 and 8 months

- KCCQ Clinical Summary Score at 1, 4 and 8 months
- KCCQ Physical Limitations Score at 1, 4 and 8 months
- KCCQ Social Limitations Score at 1, 4 and 8 months
- Proportion of patients with clinically meaningful deterioration (5 point or greater worsening), and small (≥5 point), moderate (≥10 point) and large (≥20 point) improvement in KCCQ-TSS, CSS, OSS, PL, QoL and Social Limitations Scores.

COVID-19 Related Endpoints

In addition, the following COVID-19 related endpoints will be evaluated:

- Occurrence of COVID-19 infection (documented as AE or SAE)
- Occurrence of COVID-19 related hospitalizations (overall and among patients with COVID-19 infection)
- Occurrence of COVID-19 related hospitalizations requiring ICU admission (overall and among patients with Covid-19 infection)
- Occurrence of COVID-19 related deaths (overall and among patients with Covid-19 infection)
- Acute kidney injury and initiation of dialysis reported as an adverse event during hospitalization for COVID-19
- Requirement for mechanical ventilation reported as an adverse event during COVID-19 hospitalization
- Requirement for vasopressor support reported as an adverse event during COVID-19 hospitalization
- Sudden cardiac death/cardiac arrest requiring resuscitation during COVID-19 hospitalization
- Worsening heart failure reported during or following COVID-19 hospitalization
- Use of systemic corticosteroids for COVID-19
- Diabetic ketoacidosis reported as an adverse event during or following COVID-19 hospitalization
- Among patients with documented COVID-19 infection, total events of COVID-10 related hospitalizations and COVID-19 related deaths

3. LABORATORY-BASED ENDPOINTS OF INTEREST

In addition, the following laboratory-based endpoints will be assessed:

- eGFR-based
    - Composite of confirmed sustained decline in eGFR, ESRD, and/or renal death. Sustained decline in eGFR will be defined as ≥40%, ≥50%, ≥57% decline from baseline
    - Acute, chronic, and total eGFR slope analysis, including with blanking period to account for acute, expected eGFR changes
    - Focused examination of the “eGFR dip”, the acute changes in eGFR in the days-to-weeks after randomization
    - Recalculation of eGFR based on variable calculators (including the 2009 CKD-EPI Equation and 2021 CKD-EPI Equation)

4. BREAKDOWN OF ENDPOINTS

- Mode of death including focused examination of sudden death (as a composite with ventricular arrhythmias reported as adverse events)
- Reasons for hospitalization (total all-cause hospitalization, non-CV hospitalization, HF-related hospitalization, and other CV hospitalizations)
- 30-day readmission (all-cause and HF-related)
- Breakdown of worsening HF events (including urgent visits / Emergency Department stays / oral loop diuretic escalation)

Unknown deaths will not be included as a component of CV deaths in the primary analysis as outlined in the rSAP. In a prespecified exploratory analysis, we will apply a probabilistic model (predetermined prior to database lock) to better distinguish unknown deaths as either CV or non-CV in etiology. This probabilistic model will be built based on known clinical factors that differentially predict adjudicated known cases of CV vs. non-CV deaths.

5. SUBGROUPS

In addition to the subgroups listed in the rSAP, the following subgroups of interest will be explored to examine event rates and for consistency of efficacy and safety of dapagliflozin. All subgroups will be identified based on randomization or pre-randomization data unless otherwise specified. For each subgroup, we will assess the treatment effect and interaction with treatment for the primary endpoint and each of the secondary endpoints, including components of the primary endpoint, measures of quality of life (KCCQ), NYHA class, and the renal composite endpoint. In addition, all subgroups will be assessed in the LVEF < 60% subgroup.

- Improved/recovered LVEF (those who had LVEF ≤40% at any time prior to randomization)
- LVEF subgroups in the rSAP are specified according the following cutpoints (≤ 49%, 50 to 59%, ≥60%). Additional LVEF subgroups to limit digit preference will be considered and treatment effects will be examined across LVEF as a continuous function. In addition, the two-way interaction between sex and LVEF will be examined.
- Age subgroups in the rSAP are specified according to the following cutpoints (median age). Specific evaluation of older age categories will be considered and treatment effects will be examined across age as a continuous function
- BMI subgroups in the rSAP are specified according to the following cutpoints (30kg/m^2). BMI categories will additionally be evaluated according to the full WHO classification and treatment effects will be examined across BMI as a continuous function
- Other anthropometric indices e.g., waist-to-height ratio using quantiles and recognized cutpoints
- eGFR subgroups in the rSAP are specified according to the following cutpoints (60mL/min/1.73m^2). eGFR categories will additionally be evaluated according the full KDIGO classification and treatment effects will be examined across eGFR as a continuous function
- Focused examination of Stage IV CKD (if eGFR was less than 30mL/min/1.73m^2 at randomization or at any post-randomization measurement)
- Further breakdown of glycemic categories into no diabetes, prediabetes, and T2D and examination of treatment effects across HbA1c as a continuous measure
- Time from prior HF hospitalization

- Time from index HF diagnosis
- Background HF therapies including focused examination of patients on various combinations of therapies (including the Heart Failure Collaboratory score) and on/off MRA and on/off ARNI at randomization
- In T2D subgroup, background anti-hyperglycemic therapies including focused examination of patients on various combinations of therapies
- Patients with COPD
- Patients with OSA
- Patients with history of coronary artery disease / prior MI
- Patients with metabolic syndrome (using standard definitions)
- Subgroups based on baseline use and dosing of diuretics
- Patients with multimorbidity and frailty
- Patients with baseline risk as determined by the MAGGIC and other risk scores
- Subgroups based on baseline evidence of congestion and congestion scores
- Regional subgroups based on socioeconomic differences based on the GINI coefficient
- Subgroups based on KCCQ-TSS and other KCCQ domains at baseline.

5. ALTERNATIVE ANALYTIC APPROACHES

Unless otherwise specified, these alternative approaches will be considered for the primary endpoint and each of the secondary endpoints, including components of the primary endpoint, measures of quality of life (KCCQ), NYHA class, and the renal composite endpoint.

- Win ratio using different clinically relevant hierarchies e.g., death, heart failure hospitalization, urgent heart failure visit requiring IV therapy, outpatient therapy for worsening HF, quality of life, and kidney endpoints
- Multi-state modeling of changes in transitional states (ranging from alive and well to death)
- Estimation of time to first statistically significant benefit
- Forecasting lifetime benefit of dapagliflozin if treatment effects were assumed to be maintained long-term
- Absolute risk reductions and NNT calculation overall and across key subgroups
- Cost effectiveness based on US perspective, European perspective, and Other Regions of the World perspective
- Assessment of DELIVER trial and label eligibility in the GWTG-HF registry and other "real-world" datasets
- "Real world" application of the DELIVER trial findings to the GWTG-HF registry and other datasets to estimate projected benefit if dapagliflozin was implemented in usual care

6. COVID-19 META-ANALYSES

- Together with the subset of patients in DELIVER with COVID-19, a meta-analysis will be performed using available phase 3/4 published trials of sodium–glucose cotransporter 2 inhibitor therapies in COVID-19 (*including but not limited to DARE-19*)
- Analyses evaluating outcomes after post-randomization COVID-19 diagnosis will be performed (for instance, increase in mortality or HF event risk after COVID-19 diagnosis)

**Systemic Search**	To ensure trials beyond DARE-19 and DELIVER were not missed, a systemic search via PubMed and EMBASE will be conducted of<br>• Randomized, placebo-controlled trials of SGLT2 inhibitors in COVID-19<br>• Published between March 1st, 2020 to August 1, 2022
**Rationale**	DARE-19 randomized non-critically ill patients with one or more cardiometabolic risk factors (including T2D, HTN, ASCVD, HF or CKD) hospitalized with COVID-19 to dapagliflozin versus placebo, with one of the primary outcomes being respiratory/ cardiovascular/ kidney organ failure or death from any cause. DELIVER randomized patients with HF and LVEF above 40% to dapagliflozin or placebo, and due to the time course of the trial had many patients experiencing COVID-19 related hospitalizations and deaths. Neither trial was adequately powered to assess the effects of dapagliflozin on all-cause mortality, and specific end-organ complications. This pre-specified meta-analysis will allow for greater power to evaluate the effects of dapagliflozin on a range of COVID-19 related clinical endpoints.
**Overall Aim**	Using study-level published data from DARE-19 and participant-level data from DELIVER, we aim to estimate the effect of SGLT2 inhibitors on all-cause mortality and specific end-organ complications overall, and in clinically-relevant subgroups
**Primary Endpoint**	COVID-19 related death (this includes COVID-19 related deaths in DELIVER and all deaths in DARE-19)
**Secondary Endpoints**	• Acute kidney injury and initiation of dialysis during or following hospitalization for COVID-19<br>• Requirement for mechanical ventilation during COVID-19 hospitalization<br>• Requirement for vasopressor support during COVID-19 hospitalization<br>• Sudden cardiac death/resuscitated cardiac arrest requiring resuscitation during COVID-19 hospitalization<br>• Worsening heart failure during or following COVID-19 hospitalization<br>• Composite of COVID-19 related death and organ failure (acute kidney injury, initiation of dialysis, mechanical ventilation, vasopressor support, cardiac death/ resuscitated cardiac arrest, worsening heart failure)<br>• Composite of COVID-19 related death, acute kidney injury and initiation of dialysis.<br>• Diabetic ketoacidosis during or following COVID-19 hospitalization
**Subgroups**	• With or without diabetes<br>• With or without ASCVD<br>• With or without CKD (eGFR < 60)<br>• With or without HTN<br>• Age, sex, race, BMI, geographic region
**Statistical Analysis**	• Intention-to-treat analyses from both trials will be considered and include all randomized participants<br>• All effect sizes will be extracted as point estimates (95% CI).<br>• Statistical heterogeneity will be assessed between trials
**Risk of Bias**	Study quality will be evaluated using the Cochrane Risk of Bias Tool
**Reporting**	This planned meta-analysis will be conducted and reported in accordance with the Preferred Reporting Items for Systematic Reviews and Meta-Analysis statement
**Registration**	This meta-analysis will be registered on PROSPERO

7. META-ANALYSIS OF SGLT2 INHIBITOR HFPEF TRIALS AND OTHER SGLT2 INHIBITOR TRIALS

A meta-analysis will be performed using available phase 3/4 published trials of other sodium–glucose cotransporter 2 inhibitor therapies in HFpEF, including but not limited to EMPEROR-Preserved.

**Systemic Search**	To ensure trials beyond EMPEROR-Preserved and DELIVER were not missed, a systemic search via PubMed and EMBASE will be conducted of<br>• Randomized, placebo-controlled CV and kidney outcomes trials of SGLT2 inhibitors<br>• Published between January 1, 2015 to July 1, 2022<br>• Only studies including >1,000 patients with HF and LVEF >40%
**Rationale**	Both EMPEROR-Preserved and DELIVER were similarly designed in evaluating patients with HF, an LVEF above 40%, and elevated natriuretic peptides. Neither trial was powered for mortality or kidney disease outcomes. This pre-specified meta-analysis of the 2 largest trials of HFmrEF and HFpEF will allow for greater power to evaluate a broad range of clinical endpoints and within subgroups of interest than either trial could provide alone.
**Overall Aim**	Using study-level published data from EMPEROR-Preserved and participant-level data from DELIVER, we aim to estimate the effect of SGLT2 inhibitors on cardiovascular events, kidney events, and mortality outcomes overall, and in clinically-relevant subgroups
**Primary Endpoint**	Time from randomization to the occurrence of the composite of death adjudicated as CV cause or unplanned HF hospitalization
**Secondary Endpoints**	• Time from randomization to the occurrence of the composite of death adjudicated as CV cause or a worsening HF event (including either unplanned hospitalization or urgent HF visit requiring IV therapy)<br>• Total number of worsening HF events and cardiovascular death<br>• Time from randomization to the occurrence of deaths adjudicated as CV cause<br>• Time from randomization to death from any cause<br>• Time from randomization to renal composite outcome (50% or higher sustained decline in eGFR, end stage kidney disease, or renal death)<br>• Proportion of patients with clinically meaningful deterioration (5 point or greater worsening), and small (≥5 point), moderate (≥10 point), and large (≥15 point) improvement in KCCQ-TSS, CSS, OSS
**Subgroups**	• LVEF (<50%, ≥50 to <60%, ≥60%)<br>• With or without diabetes<br>• Use of no use of ACEi/ARB/ARNI at baseline<br>• Use and no use of MRA at baseline<br>• Age (≥70 and <70 years), sex (male, female), race (White, Black, Asian, Other), BMI (<30 and ≥30 kg/m^2), eGFR (≥60 and <60mL/min/1.73m^2), systolic blood pressure, history of AF/AFL, hospitalization for HF within 12 months, NYHA class (II and III/IV)
**Statistical Analysis**	• Fixed effects model<br>• Only intention-to-treat analyses from both trials will be considered and include all randomized participants<br>• All effect sizes will be extracted as point estimates (95% CI). For the time-to-first event endpoints, Cox proportional hazards models will be used for hazard ratio (HR) and 95% CI. Recurrent event analyses will be based on the Lin-Wei-Yang-Ying model and summarized as

	rate ratio (RR) and 95% CI. Responder analyses for KCCQ changes will be based on logistic regression analyses summarized as odds ratios with 95% CIs.<br>• The continuous association between LVEF and treatment effects on the primary endpoint will be assessed with restricted cubic spline analyses. Data from these published splines in the EMPEROR program will be digitized using a validated, semiautomatic tool (DigitizeIt software https://www.digitizeit.xyz/).<br>• Statistical heterogeneity will be assessed between trials
**Risk of Bias**	Study quality will be evaluated using the Cochrane Risk of Bias Tool
**Reporting**	This planned meta-analysis will be conducted and reported in accordance with the Preferred Reporting Items for Systematic Reviews and Meta-Analysis statement
**Registration**	This meta-analysis will be registered on PROSPERO

Meta-analyses will also be performed using available phase 3/4 published trials of other sodium–glucose cotransporter 2 inhibitor therapies in different disease states to provide a comprehensive assessment of the value of SGLT2 inhibitors across the disease spectrum.

Appendix B Programming Code for Calculating Significance Level for LVEF <60% Subpopulation

Let Z_1 and Z_2 denote the standardized test statistic for testing the hypothesis of treatment effect in the LVEF < 60% subgroup and the full population respectively. Z_1 and Z_2 are bivariate normal with correlation equal to the proportion of events in the LVEF < 60% subpopulation (Spiessen and Debois 2010). To control the familywise error rate below α, for a pre-specified significance level α_2 for the full population, we need to define α_1 for the subgroup such that, under the null hypothesis,

(1) $P\left(Z_1 > z_{\alpha_1} \text{ OR } Z_2 > z_{\alpha_2}\right) = \alpha$

where z_{α_1} and z_{α_2} are the corresponding critical values from the standard normal distribution.

Equation (1) can be rewritten as

$P\left(Z_2 > z_{\alpha_2}\right) + P\left(Z_1 > z_{\alpha_1}, Z_2 \leq z_{\alpha_2}\right)$

$= \alpha_2 + P\left(Z_1 > z_{\alpha_1}, Z_2 \leq z_{\alpha_2}\right)$

$= \alpha$

Thus we need to find α_1 such that

$P\left(Z_1 > z_{\alpha_1}, Z_2 \leq z_{\alpha_2}\right) = \alpha - \alpha_2$

As noted by Spiessen and Debois 2010, this corresponds to error spending for group sequential methods where Z_2 is the test statistic at interim analysis and Z_1 is the test statistic at the final analysis. Accordingly, standard software for group sequential designs can be used to calculate the significance level α_1 as shown below using the R package gsDesign or the SAS procedure SEQDESIGN

For the proportion of events in the LVEF <60% subgroup we use the lower bound of a 95% confidence interval for the estimated proportion calculated using normal approximation as

$$p - z_{2.5}\sqrt{p(1-p)/e}$$

where $z_{2.5}$ is the upper 2.5% percentile of the standard normal distribution and $p = e_{60}/e$ for e_{60} events in the subgroup and e events in total.

In an example of 810 (72.5%) in the subgroup out of a total of 1117 event, the lower confidence limit for the proportion is 0.699, which will be used in the example R and SAS code below.

R gsDesign package

Example with lower proportion 0.699 (lower confidence limit) events in the subgroup:

```
gsd <-
   gsDesign(k=2, timing=c(0.699,1),
      # 69.9% events at interim, corresponding to proportion in subgroup
   test.type=1, sfu=sfLinear,
   sfupar=c(0.699,1,0.5,1),
      # proportion 0.5 of total alpha spent at interim, corresponding to
      # alpha2 = 0.5*0.048=0.024 two-sided set for the full population
    alpha=0.024)
      #total one-sided alpha 0.048/2
                       #
(alpha1 <- 2*100*(1-pnorm(gsd$upper$bound[2])))
       # alpha1 now holds the two-sided significance level for final
       # analysis, corresponding to alpha1 for the subgroup
```

SAS proc SEQDESIGN

Example with lower proportion 0.699 (lower confidence limit) events in the subgroup:

```
proc seqdesign bscale=pvalue;
     design nstages=2 info=cum(69.9 100)
     /* 69.9% events at interim, corresponding to proportion in subgroup */
     method=peto(pvalue=0.012)
     /* alpha 0.012 one-sided spent at interim, corresponding to
        0.024 two-sided set for the full population */
         stop=reject
         alt=lower alpha=0.024;
     /*   0.048/2 total one-sided alpha  */
     ods output boundary = bound;
  run;

  data alpha1;
    set bound;
    if _stage_=2;
    alpha1=100*2*bound_la;
  run;

   /* alpha1 now holds the two-sided significance level for final analysis,
      corresponding to alpha1 for the subgroup */
```

SIGNATURE PAGE

This is a representation of an electronic record that was signed electronically and this page is the manifestation of the electronic signature

**Document Name:** d169cc00001-statistical-analysis-plan-v5		
**Document Title:**	Statistical Analysis Plan Version 5	
**Document ID:**	Doc ID-003872995	
**Version Label:**	5.0 CURRENT LATEST APPROVED	
**Server Date** **(dd-MMM-yyyy HH:mm 'UTC'Z)**	**Signed by**	**Meaning of Signature**
15-Dec-2021 13:51 UTC	Olof Bengtsson	Content Approval
15-Dec-2021 12:21 UTC	Ulrica Wilderäng	Content Approval

Notes: (1) Document details as stored in ANGEL, an AstraZeneca document management system.

Academic Statistical Analysis Plan	
Study Code	D169CC00001
Edition Number	1.3
Date	April 15, 2022

DELIVER: Academic Statistical Analysis Plan

Dapagliflozin Evaluation to Improve the LIVEs of Patients with PReserved Ejection Fraction Heart Failure

An International, Double-blind, Randomised, Placebo-Controlled Phase III Study to Evaluate the Effect of Dapagliflozin on Reducing CV Death or Worsening Heart Failure in Patients with Heart Failure with Preserved Ejection Fraction (HFpEF)

DELIVER: Academic Statistical Analysis Plan

Dapagliflozin Evaluation to Improve the LIVEs of Patients with PReserved Ejection Fraction Heart Failure

An International, Double-blind, Randomised, Placebo-Controlled Phase III Study to Evaluate the Effect of Dapagliflozin on Reducing CV Death or Worsening Heart Failure in Patients with Heart Failure with Preserved Ejection Fraction (HFpEF)

DELIVER CO-CHAIRS, STEERING COMMITTEE

Scott D. Solomon, MD
Brigham and Women's Hospital

Scott Solomon (Apr 15, 2022 15:38 EDT) Apr 15, 2022

Signature Date

John J.V. McMurray, MD
University of Glasgow

Apr 15, 2022

Signature Date

Table of Contents

1. INTRODUCTION 4
2. CLINICAL ENDPOINTS OF INTEREST 4
3. LABORATORY-BASED ENDPOINTS OF INTEREST 5
4. BREAKDOWN OF ENDPOINTS 6
5. SUBGROUPS 6
5. ALTERNATIVE ANALYTIC APPROACHES 7
6. COVID-19 7
7. META-ANALYSES 9

1. INTRODUCTION

DELIVER is an international, multicentre, parallel group, event-driven, randomized, double-blind trial in patients with chronic heart failure and left ventricular ejection fraction (LVEF) >40%, comparing the effect of dapagliflozin 10 mg once daily, vs. placebo, in addition to standard of care. Patients with or without diabetes, with signs and symptoms of heart failure, a LVEF >40%, elevation in natriuretic peptides and evidence of structural heart disease are eligible. The primary endpoint is time-to-first cardiovascular death or worsening heart failure event (heart failure hospitalization or urgent heart failure visit), and will be assessed in dual primary analyses – the full population and in those with LVEF <60%. The study is event-driven and will target 1117 primary events. A total of 6,263 patients have been randomized.

The DELIVER executive committee has developed this academic statistical analysis plan (ASAP) that describes pre-specified analyses that were not described in the DELIVER regulatory SAP (rSAP). General principles outlined in the regulatory SAP will be followed unless specified otherwise here. This document is meant to supplement and complement the regulatory SAP and delineate all analyses that were pre-specified prior to database lock. When relevant, analyses will be conducted based on the pooled DAPA-HF and DELIVER dataset to examine the effects of dapagliflozin in a broad range of patients with HF.

2. CLINICAL ENDPOINTS OF INTEREST

In addition to the efficacy and safety variables listed in the rSAP, the effect of dapagliflozin on the following endpoints will be explored. These events that are imbalanced between arms may be analyzed as time-to-event to better understand the time course. All endpoints will be assessed in the full cohort and in the LVEF < 60% subgroup. These include:

- Days alive and out of the hospital
- Quality of life-adjusted days alive and out of the hospital
- Investigator reported vs. CEC-adjudicated endpoints
- Time to onset of benefit of dapagliflozin
- New diuretic initiation, discontinuation, and dose changes
- New onset atrial fibrillation
- In the T2D subgroup, new glucose lowering therapy initiation and changes in insulin dose (in those on insulin at baseline)
- In the non-T2D subgroup, new diagnosis of diabetes
- Signs and symptoms of HF
- Patient Global Impression of Severity
- Target risk factor control (for blood pressure, smoking, antiplatelet/anticoagulant therapy)
- Cardiac ischemic events including myocardial infarction, unstable angina, unplanned coronary revascularization, and stroke
- Hyperkalemia as a reported adverse event and initiation of new potassium-lowering therapy
- Acute kidney injury as a reported adverse event and initiation of dialysis
- Anemia and requirement for blood transfusion as reported adverse events
- Gout as an adverse event and initiation of new uric acid-lowering therapy
- KCCQ Overall Summary Score at 1, 4 and 8 months

- KCCQ Clinical Summary Score at 1, 4 and 8 months
- KCCQ Physical Limitations Score at 1, 4 and 8 months
- KCCQ Social Limitations Score at 1, 4 and 8 months
- Proportion of patients with clinically meaningful deterioration (5 point or greater worsening), and small (≥5 point), moderate (≥10 point) and large (≥20 point) improvement in KCCQ-TSS, CSS, OSS, PL, QoL and Social Limitations Scores.

COVID-19 Related Endpoints

In addition, the following COVID-19 related endpoints will be evaluated:

- Occurrence of COVID-19 infection (documented as AE or SAE)
- Occurrence of COVID-19 related hospitalizations (overall and among patients with COVID-19 infection)
- Occurrence of COVID-19 related hospitalizations requiring ICU admission (overall and among patients with Covid-19 infection)
- Occurrence of COVID-19 related deaths (overall and among patients with Covid-19 infection)
- Acute kidney injury and initiation of dialysis reported as an adverse event during hospitalization for COVID-19
- Requirement for mechanical ventilation reported as an adverse event during COVID-19 hospitalization
- Requirement for vasopressor support reported as an adverse event during COVID-19 hospitalization
- Sudden cardiac death/cardiac arrest requiring resuscitation during COVID-19 hospitalization
- Worsening heart failure reported during or following COVID-19 hospitalization
- Use of systemic corticosteroids for COVID-19
- Diabetic ketoacidosis reported as an adverse event during or following COVID-19 hospitalization
- Among patients with documented COVID-19 infection, total events of COVID-10 related hospitalizations and COVID-19 related deaths

3. LABORATORY-BASED ENDPOINTS OF INTEREST

In addition, the following laboratory-based endpoints will be assessed:

- eGFR-based
  - Composite of confirmed sustained decline in eGFR, ESRD, and/or renal death. Sustained decline in eGFR will be defined as ≥40%, ≥50%, ≥57% decline from baseline
  - Acute, chronic, and total eGFR slope analysis, including with blanking period to account for acute, expected eGFR changes
  - Focused examination of the “eGFR dip”, the acute changes in eGFR in the days-to-weeks after randomization
  - Recalculation of eGFR based on variable calculators (including the 2009 CKD-EPI Equation and 2021 CKD-EPI Equation)

4. BREAKDOWN OF ENDPOINTS

- Mode of death including focused examination of sudden death (as a composite with ventricular arrhythmias reported as adverse events)
- Reasons for hospitalization (total all-cause hospitalization, non-CV hospitalization, HF-related hospitalization, and other CV hospitalizations)
- 30-day readmission (all-cause and HF-related)
- Breakdown of worsening HF events (including urgent visits / Emergency Department stays / oral loop diuretic escalation)

Unknown deaths will not be included as a component of CV deaths in the primary analysis as outlined in the rSAP. In a prespecified exploratory analysis, we will apply a probabilistic model (predetermined prior to database lock) to better distinguish unknown deaths as either CV or non-CV in etiology. This probabilistic model will be built based on known clinical factors that differentially predict adjudicated known cases of CV vs. non-CV deaths.

5. SUBGROUPS

In addition to the subgroups listed in the rSAP, the following subgroups of interest will be explored to examine event rates and for consistency of efficacy and safety of dapagliflozin. All subgroups will be identified based on randomization or pre-randomization data unless otherwise specified. For each subgroup, we will assess the treatment effect and interaction with treatment for the primary endpoint and each of the secondary endpoints, including components of the primary endpoint, measures of quality of life (KCCQ), NYHA class, and the renal composite endpoint. In addition, all subgroups will be assessed in the LVEF < 60% subgroup.

- Improved/recovered LVEF (those who had LVEF ≤40% at any time prior to randomization)
- LVEF subgroups in the rSAP are specified according the following cutpoints (≤ 49%, 50 to 59%, ≥60%). Additional LVEF subgroups to limit digit preference will be considered and treatment effects will be examined across LVEF as a continuous function. In addition, the two-way interaction between sex and LVEF will be examined.
- Age subgroups in the rSAP are specified according to the following cutpoints (median age). Specific evaluation of older age categories will be considered and treatment effects will be examined across age as a continuous function
- BMI subgroups in the rSAP are specified according to the following cutpoints ($30kg/m^2$). BMI categories will additionally be evaluated according to the full WHO classification and treatment effects will be examined across BMI as a continuous function
- Other anthropometric indices e.g., waist-to-height ratio using quantiles and recognized cutpoints
- eGFR subgroups in the rSAP are specified according to the following cutpoints ($60mL/min/1.73m^2$). eGFR categories will additionally be evaluated according the full KDIGO classification and treatment effects will be examined across eGFR as a continuous function
- Focused examination of Stage IV CKD (if eGFR was less than $30mL/min/1.73m^2$ at randomization or at any post-randomization measurement)
- Further breakdown of glycemic categories into no diabetes, prediabetes, and T2D and examination of treatment effects across HbA1c as a continuous measure
- Time from prior HF hospitalization

- Time from index HF diagnosis
- Background HF therapies including focused examination of patients on various combinations of therapies (including the Heart Failure Collaboratory score) and on/off MRA and on/off ARNI at randomization
- In T2D subgroup, background anti-hyperglycemic therapies including focused examination of patients on various combinations of therapies
- Patients with COPD
- Patients with OSA
- Patients with history of coronary artery disease / prior MI
- Patients with metabolic syndrome (using standard definitions)
- Subgroups based on baseline use and dosing of diuretics
- Patients with multimorbidity and frailty
- Patients with baseline risk as determined by the MAGGIC and other risk scores
- Subgroups based on baseline evidence of congestion and congestion scores
- Regional subgroups based on socioeconomic differences based on the GINI coefficient
- Subgroups based on KCCQ-TSS and other KCCQ domains at baseline.

5. ALTERNATIVE ANALYTIC APPROACHES

Unless otherwise specified, these alternative approaches will be considered for the primary endpoint and each of the secondary endpoints, including components of the primary endpoint, measures of quality of life (KCCQ), NYHA class, and the renal composite endpoint.

- Win ratio using different clinically relevant hierarchies e.g., death, heart failure hospitalization, urgent heart failure visit requiring IV therapy, outpatient therapy for worsening HF, quality of life, and kidney endpoints
- Multi-state modeling of changes in transitional states (ranging from alive and well to death)
- Estimation of time to first statistically significant benefit
- Forecasting lifetime benefit of dapagliflozin if treatment effects were assumed to be maintained long-term
- Absolute risk reductions and NNT calculation overall and across key subgroups
- Cost effectiveness based on US perspective, European perspective, and Other Regions of the World perspective
- Assessment of DELIVER trial and label eligibility in the GWTG-HF registry and other “real-world” datasets
- “Real world” application of the DELIVER trial findings to the GWTG-HF registry and other datasets to estimate projected benefit if dapagliflozin was implemented in usual care

6. COVID-19 META-ANALYSES

- Together with the subset of patients in DELIVER with COVID-19, a meta-analysis will be performed using available phase 3/4 published trials of sodium–glucose cotransporter 2 inhibitor therapies in COVID-19 (*including but not limited to DARE-19*)
- Analyses evaluating outcomes after post-randomization COVID-19 diagnosis will be performed (for instance, increase in mortality or HF event risk after COVID-19 diagnosis)

**Systemic Search**	To ensure trials beyond DARE-19 and DELIVER were not missed, a systemic search via PubMed and EMBASE will be conducted of<br>• Randomized, placebo-controlled trials of SGLT2 inhibitors in COVID-19<br>• Published between March 1st, 2020 to August 1, 2022
**Rationale**	DARE-19 randomized non-critically ill patients with one or more cardiometabolic risk factors (including T2D, HTN, ASCVD, HF or CKD) hospitalized with COVID-19 to dapagliflozin versus placebo, with one of the primary outcomes being respiratory/ cardiovascular/ kidney organ failure or death from any cause. DELIVER randomized patients with HF and LVEF above 40% to dapagliflozin or placebo, and due to the time course of the trial had many patients experiencing COVID-19 related hospitalizations and deaths. Neither trial was adequately powered to assess the effects of dapagliflozin on all-cause mortality, and specific end-organ complications. This pre-specified meta-analysis will allow for greater power to evaluate the effects of dapagliflozin on a range of COVID-19 related clinical endpoints.
**Overall Aim**	Using study-level published data from DARE-19 and participant-level data from DELIVER, we aim to estimate the effect of SGLT2 inhibitors on all-cause mortality and specific end-organ complications overall, and in clinically-relevant subgroups
**Primary Endpoint**	COVID-19 related death (this includes COVID-19 related deaths in DELIVER and all deaths in DARE-19)
**Secondary Endpoints**	• Acute kidney injury and initiation of dialysis during or following hospitalization for COVID-19<br>• Requirement for mechanical ventilation during COVID-19 hospitalization<br>• Requirement for vasopressor support during COVID-19 hospitalization<br>• Sudden cardiac death/resuscitated cardiac arrest requiring resuscitation during COVID-19 hospitalization<br>• Worsening heart failure during or following COVID-19 hospitalization<br>• Composite of COVID-19 related death and organ failure (acute kidney injury, initiation of dialysis, mechanical ventilation, vasopressor support, cardiac death/ resuscitated cardiac arrest, worsening heart failure)<br>• Composite of COVID-19 related death, acute kidney injury and initiation of dialysis.<br>• Diabetic ketoacidosis during or following COVID-19 hospitalization
**Subgroups**	• With or without diabetes<br>• With or without ASCVD<br>• With or without CKD (eGFR < 60)<br>• With or without HTN<br>• Age, sex, race, BMI, geographic region
**Statistical Analysis**	• Intention-to-treat analyses from both trials will be considered and include all randomized participants<br>• All effect sizes will be extracted as point estimates (95% CI).<br>• Statistical heterogeneity will be assessed between trials
**Risk of Bias**	Study quality will be evaluated using the Cochrane Risk of Bias Tool
**Reporting**	This planned meta-analysis will be conducted and reported in accordance with the Preferred Reporting Items for Systematic Reviews and Meta-Analysis statement
**Registration**	This meta-analysis will be registered on PROSPERO

7. META-ANALYSIS OF SGLT2 INHIBITOR HFPEF TRIALS AND OTHER SGLT2 INHIBITOR TRIALS

A meta-analysis will be performed using available phase 3/4 published trials of other sodium–glucose cotransporter 2 inhibitor therapies in HFpEF, including but not limited to EMPEROR-Preserved.

**Systemic Search**	To ensure trials beyond EMPEROR-Preserved and DELIVER were not missed, a systemic search via PubMed and EMBASE will be conducted of<br>• Randomized, placebo-controlled CV and kidney outcomes trials of SGLT2 inhibitors<br>• Published between January 1, 2015 to July 1, 2022<br>• Only studies including >1,000 patients with HF and LVEF >40%
**Rationale**	Both EMPEROR-Preserved and DELIVER were similarly designed in evaluating patients with HF, an LVEF above 40%, and elevated natriuretic peptides. Neither trial was powered for mortality or kidney disease outcomes. This pre-specified meta-analysis of the 2 largest trials of HFmrEF and HFpEF will allow for greater power to evaluate a broad range of clinical endpoints and within subgroups of interest than either trial could provide alone.
**Overall Aim**	Using study-level published data from EMPEROR-Preserved and participant-level data from DELIVER, we aim to estimate the effect of SGLT2 inhibitors on cardiovascular events, kidney events, and mortality outcomes overall, and in clinically-relevant subgroups
**Primary Endpoint**	Time from randomization to the occurrence of the composite of death adjudicated as CV cause or unplanned HF hospitalization
**Secondary Endpoints**	• Time from randomization to the occurrence of the composite of death adjudicated as CV cause or a worsening HF event (including either unplanned hospitalization or urgent HF visit requiring IV therapy)<br>• Total number of worsening HF events and cardiovascular death<br>• Time from randomization to the occurrence of deaths adjudicated as CV cause<br>• Time from randomization to death from any cause<br>• Time from randomization to renal composite outcome (50% or higher sustained decline in eGFR, end stage kidney disease, or renal death)<br>• Proportion of patients with clinically meaningful deterioration (5 point or greater worsening), and small (≥5 point), moderate (≥10 point), and large (≥15 point) improvement in KCCQ-TSS, CSS, OSS
**Subgroups**	• LVEF (<50%, ≥50 to <60%, ≥60%)<br>• With or without diabetes<br>• Use of no use of ACEi/ARB/ARNI at baseline<br>• Use and no use of MRA at baseline<br>• Age (≥70 and <70 years), sex (male, female), race (White, Black, Asian, Other), BMI (<30 and ≥30 kg/m^2), eGFR (≥60 and <60mL/min/1.73m^2), systolic blood pressure, history of AF/AFL, hospitalization for HF within 12 months, NYHA class (II and III/IV)
**Statistical Analysis**	• Fixed effects model<br>• Only intention-to-treat analyses from both trials will be considered and include all randomized participants<br>• All effect sizes will be extracted as point estimates (95% CI). For the time-to-first event endpoints, Cox proportional hazards models will be used for hazard ratio (HR) and 95% CI. Recurrent event analyses will be based on the Lin-Wei-Yang-Ying model and summarized as

	rate ratio (RR) and 95% CI. Responder analyses for KCCQ changes will be based on logistic regression analyses summarized as odds ratios with 95% CIs.<br>• The continuous association between LVEF and treatment effects on the primary endpoint will be assessed with restricted cubic spline analyses. Data from these published splines in the EMPEROR program will be digitized using a validated, semiautomatic tool (DigitizeIt software https://www.digitizeit.xyz/).<br>• Statistical heterogeneity will be assessed between trials
**Risk of Bias**	Study quality will be evaluated using the Cochrane Risk of Bias Tool
**Reporting**	This planned meta-analysis will be conducted and reported in accordance with the Preferred Reporting Items for Systematic Reviews and Meta-Analysis statement
**Registration**	This meta-analysis will be registered on PROSPERO

Meta-analyses will also be performed using available phase 3/4 published trials of other sodium–glucose cotransporter 2 inhibitor therapies in different disease states to provide a comprehensive assessment of the value of SGLT2 inhibitors across the disease spectrum.
