# Supplementary material for: Dapagliflozin in heart failure with improved ejection fraction: a prespecified analysis of the DELIVER trial
Source: Nat Med. 2022 Dec 15;28(12):2504–11. doi: 10.1038/s41591-022-02102-9 (PMC9800271; doi:10.1038/s41591-022-02102-9)
Supplement: Supplementary file 2 — Reporting Summary [file 41591_2022_2102_MOESM2_ESM.pdf]

nature portfolio

Corresponding author(s): Scott Solomon & Orly Vardeny

Last updated by author(s): Oct 12, 2022

# Reporting Summary

Nature Portfolio wishes to improve the reproducibility of the work that we publish. This form provides structure for consistency and transparency in reporting. For further information on Nature Portfolio policies, see our Editorial Policies and the Editorial Policy Checklist.

## Statistics

For all statistical analyses, confirm that the following items are present in the figure legend, table legend, main text, or Methods section.

| n/a | Confirmed | |
|---|---|---|
| ☐ | ☒ | The exact sample size (*n*) for each experimental group/condition, given as a discrete number and unit of measurement |
| ☐ | ☒ | A statement on whether measurements were taken from distinct samples or whether the same sample was measured repeatedly |
| ☐ | ☒ | The statistical test(s) used AND whether they are one- or two-sided<br>*Only common tests should be described solely by name; describe more complex techniques in the Methods section.* |
| ☐ | ☒ | A description of all covariates tested |
| ☐ | ☒ | A description of any assumptions or corrections, such as tests of normality and adjustment for multiple comparisons |
| ☐ | ☒ | A full description of the statistical parameters including central tendency (e.g. means) or other basic estimates (e.g. regression coefficient) AND variation (e.g. standard deviation) or associated estimates of uncertainty (e.g. confidence intervals) |
| ☐ | ☒ | For null hypothesis testing, the test statistic (e.g. *F*, *t*, *r*) with confidence intervals, effect sizes, degrees of freedom and *P* value noted<br>*Give P values as exact values whenever suitable.* |
| ☒ | ☐ | For Bayesian analysis, information on the choice of priors and Markov chain Monte Carlo settings |
| ☒ | ☐ | For hierarchical and complex designs, identification of the appropriate level for tests and full reporting of outcomes |
| ☒ | ☐ | Estimates of effect sizes (e.g. Cohen's *d*, Pearson's *r*), indicating how they were calculated |

*Our web collection on statistics for biologists contains articles on many of the points above.*

## Software and code

Policy information about availability of computer code

| | |
|---|---|
| Data collection | N/A |
| Data analysis | Stata version 17.0 (College Station, TX, USA) |

For manuscripts utilizing custom algorithms or software that are central to the research but not yet described in published literature, software must be made available to editors and reviewers. We strongly encourage code deposition in a community repository (e.g. GitHub). See the Nature Portfolio guidelines for submitting code & software for further information.

## Data

Policy information about availability of data

All manuscripts must include a data availability statement. This statement should provide the following information, where applicable:

- Accession codes, unique identifiers, or web links for publicly available datasets
- A description of any restrictions on data availability
- For clinical datasets or third party data, please ensure that the statement adheres to our policy

Data underlying the findings described in this manuscript may be obtained following AstraZeneca's data sharing policy described at https://astrazenecagrouptrials.pharmacm.com/ST/Submission/Disclosure.

## Human research participants

Policy information about studies involving human research participants and Sex and Gender in Research.

| | |
|---|---|
| Reporting on sex and gender | The study included ~44% women, which was determined based on self-report. The primary study results were analyzed by sex and shown in figure 3, these analyses were planned a priori and are described in the statistical analysis plan (uploaded with the manuscript). |
| Population characteristics | Patients enrolled in the DELIVER trial were age 40 or older with symptomatic heart failure (New York Heart Association [NYHA] functional class II-IV) and a left ventricular ejection fraction >40% (within 12 months of enrollment), elevated levels of natriuretic peptides (N-terminal pro-B-type natriuretic peptide [NT-proBNP] of at least 300pg/ml in those without atrial fibrillation or flutter, or at least 600pg/ml in those in atrial fibrillation or flutter) and evidence of structural heart disease (left atrial enlargement or left ventricular hypertrophy). Patients with or without type 2 diabetes mellitus, either an outpatient or hospitalized for worsening heart failure were eligible for enrollment. Patients were also eligible if they previously had LVEF ≤ 40% but had LVEF > 40% on their qualifying echocardiogram at enrollment. The main exclusion criteria were the use of SGLT2i within 4 weeks of randomization, prior intolerance to SGLT2i, type 1 diabetes mellitus, estimated glomerular filtration rate (eGFR) <25ml/min1.73m2 at screening, and systolic blood pressure ≥160mmHg if not using 3 or more blood pressure lowering medications or ≥180mmHg regardless of the number of antihypertensives. Patients were also excluded if they had diagnoses that could alternatively account for their HF symptoms (e.g. anemia, primary pulmonary hypertension). |
| Recruitment | Participants were recruited from 353 performance sites across 20 countries. Participants were enrolled as part of an outpatient visit or during a hospitalization for heart failure. All participants had to fulfill inclusion and exclusion criteria and there was no self-selection. The effects of any selection bias on the outcomes were minimal given the randomized design. |
| Ethics oversight | The protocol was approved by local ethics committees at each participating site, and each patient provided written informed consent in accordance with established guidelines. The complete list of investigators and institutions is listed in the supplementary appendix. |

Note that full information on the approval of the study protocol must also be provided in the manuscript.

# Field-specific reporting

Please select the one below that is the best fit for your research. If you are not sure, read the appropriate sections before making your selection.

☒ Life sciences ☐ Behavioural & social sciences ☐ Ecological, evolutionary & environmental sciences

For a reference copy of the document with all sections, see nature.com/documents/nr-reporting-summary-flat.pdf

# Life sciences study design

All studies must disclose on these points even when the disclosure is negative.

| | |
|---|---|
| Sample size | Sample size was 6263 participants. The sample size and power calculations are described in the following:<br>1: Solomon SD, de Boer RA, DeMets D, Hernandez AF, Inzucchi SE, Kosiborod MN, Lam CSP, Martinez F, Shah SJ, Lindholm D, Wilderäng U, Öhrn F, Claggett B, Langkilde AM, Petersson M, McMurray JJV. Dapagliflozin in heart failure with preserved and mildly reduced ejection fraction: rationale and design of the DELIVER trial. Eur J Heart Fail. 2021 Jul;23(7):1217-1225. doi: 10.1002/ejhf.2249. |
| Data exclusions | No data were excluded |
| Replication | This is a prospectively designed clinical trial. The data analyses were performed independently by the sponsor and by the academic statistician at Brigham and Women's Hospital. |
| Randomization | The study was randomized, double blind, and placebo controlled |
| Blinding | The trial was double blind |

# Reporting for specific materials, systems and methods

We require information from authors about some types of materials, experimental systems and methods used in many studies. Here, indicate whether each material, system or method listed is relevant to your study. If you are not sure if a list item applies to your research, read the appropriate section before selecting a response.

## Materials & experimental systems

| n/a | Involved in the study |
|---|---|
| ☒ | ☐ Antibodies |
| ☒ | ☐ Eukaryotic cell lines |
| ☒ | ☐ Palaeontology and archaeology |
| ☒ | ☐ Animals and other organisms |
| ☐ | ☒ Clinical data |
| ☒ | ☐ Dual use research of concern |

## Methods

| n/a | Involved in the study |
|---|---|
| ☒ | ☐ ChIP-seq |
| ☒ | ☐ Flow cytometry |
| ☒ | ☐ MRI-based neuroimaging |

# Clinical data

Policy information about clinical studies

All manuscripts should comply with the ICMJE guidelines for publication of clinical research and a completed CONSORT checklist must be included with all submissions.

Clinical trial registration

clinicaltrials.gov NCT03619213

Study protocol

The study protocol was submitted with the manuscript in the supplementary material and is also available at https://www.nejm.org/doi/suppl/10.1056/NEJMoa2206286/suppl_file/nejmoa2206286_protocol.pdf,

Data collection

Data were collected at study sites by investigators and by AstraZeneca during the trial, but were analyzed independently at the Brigham and Women's Hospital. Enrollment in DELIVER began on 27 August 2018 and the last patient was randomized on 18 January 2021, with patients enrolled at 353 sites, in 20 countries . See also:
1: Solomon SD, de Boer RA, DeMets D, Hernandez AF, Inzucchi SE, Kosiborod MN, Lam CSP, Martinez F, Shah SJ, Lindholm D, Wilderäng U, Öhrn F, Claggett B, Langkilde AM, Petersson M, McMurray JJV. Dapagliflozin in heart failure with preserved and mildly reduced ejection fraction: rationale and design of the DELIVER trial. Eur J Heart Fail. 2021 Jul;23(7):1217-1225. doi: 10.1002/ejhf.2249.

Outcomes

The DELIVER trial was event-driven and the primary endpoint was a composite of the time to the first occurrence of worsening heart failure or death from a cardiovascular cause. Worsening heart failure was defined as unplanned hospital admission for heart failure or an urgent visit for worsening heart failure resulting in the administration of an intravenous diuretic.
An independent Cardiovascular Endpoint Committee (CEC), blinded to treatment allocation, adjudicated all deaths and non-fatal cardiovascular events submitted by investigators (or otherwise identified) as possible endpoints using a charter reflecting the 2017 Cardiovascular and Stroke Endpoint Definitions for Clinical Trials developed by the Standardized Data Collection for Cardiovascular Trials Initiative:
1.Hicks KA, Mahaffey KW, Mehran R, Nissen SE, Wiviott SD, Dunn B, Solomon SD, Marler JR, Teerlink JR, Farb A, Morrow DA, Targum SL, Sila CA, Hai MT, Jaff MR, Joffe HV, Cutlip DE, Desai AS, Lewis EF, Gibson CM, Landray MJ, Lincoff AM, White CJ, Brooks SS, Rosenfield K, Domanski MJ, Lansky AJ, McMurray JJ, Tcheng JE, Steinhubl SR, Burton P, Mauri L, O'Connor CM, Pfeffer MA, Hung HM, Stockbridge NL, Chaitman BR, Temple RJ; Standardized Data Collection for Cardiovascular Trials Initiative (SCTI) . 2017 Cardiovascular and stroke endpoint definitions for clinical trials. Circulation 2018;137:961–972
Please see statistical plans submitted.
